# Functional requirements driving the gene duplication in 12 *Drosophila* species

**DOI:** 10.1186/1471-2164-14-555

**Published:** 2013-08-15

**Authors:** Yan Zhong, Yanxiao Jia, Yang Gao, Dacheng Tian, Sihai Yang, Xiaohui Zhang

**Affiliations:** 1 State Key Laboratory of Pharmaceutical Biotechnology, School of Life Sciences, Nanjing University, 22 Hankou Rd., Nanjing 210093, China

**Keywords:** Young duplication, Environmental factor, Convergent evolution, Adaptive evolution

## Abstract

**Background:**

Gene duplication supplies the raw materials for novel gene functions and many gene families arisen from duplication experience adaptive evolution. Most studies of young duplicates have focused on mammals, especially humans, whereas reports describing their genome-wide evolutionary patterns across the closely related *Drosophila* species are rare. The sequenced 12 *Drosophila* genomes provide the opportunity to address this issue.

**Results:**

In our study, 3,647 young duplicate gene families were identified across the 12 *Drosophila* species and three types of expansions, species-specific, lineage-specific and complex expansions, were detected in these gene families. Our data showed that the species-specific young duplicate genes predominated (86.6%) over the other two types. Interestingly, many independent species-specific expansions in the same gene family have been observed in many species, even including 11 or 12 *Drosophila* species. Our data also showed that the functional bias observed in these young duplicate genes was mainly related to responses to environmental stimuli and biotic stresses.

**Conclusions:**

This study reveals the evolutionary patterns of young duplicates across 12 *Drosophila* species on a genomic scale. Our results suggest that convergent evolution acts on young duplicate genes after the species differentiation and adaptive evolution may play an important role in duplicate genes for adaption to ecological factors and environmental changes in *Drosophila*.

## Background

Gene duplication is one of the dominant driving forces in adaptive evolution of genome and genetic systems [1]. Duplicate genes are considered to be the raw materials and the primary mechanism for generation of novel gene functions [2]. At least 15% of genes in human genome and 8% to 20% of *Drosophila melanogaster*, *Caenorhabditis elegans*, and *Saccharomyces cervisiae* genomes are thought to arise from gene duplications [3].

Young duplicate genes will ultimately suffer one of three long-term fates: (i) one copy may lose gene function by nonfunctionalization/pseudogenization; (ii) one copy may evolve a new beneficial function by means of neofunctionalization and the other retain the old; or (iii) both duplicated copies may stably be maintained with daughter copy partitioning of ancestral gene function by subfunctionalization [3-7]. Many models have been proposed that pseudogenization could be the most common fate of duplicated genes [8-10]. In addition, evidences for adaptive evolution of pseudogenes have been reported in many organisms, such as pseudogenes in 80 *Arabidopsis* accessions [11] and the *rcsA* gene in *Yersinia pestis*[12]. Similarly, the preservation of duplicated genes might be a by-product of neutral evolution [1,9,13], or might be adaptive substitutions during or after fixation of duplicates [1], indicating that selection for neofunctionalization is the mechanism to keep them [14,15].

Previous studies conducted in many organisms have widely reported that duplicate genes undergo adaptive evolution. At the genome-wide level, the signatures of adaptive natural selection of young gene duplicates are found with high frequency in the human, macaque, mouse and rat genomes [16]. Furthermore, gene duplicates from *Drosophila pseudoobscura* neo-X chromosome [17] and a group of digestive protease encoding genes that are associated with recent, lineage-specific duplications in *Drosophila Arizonae*[18] are detected under adaptive evolution. Researches have been focused almost either on recent duplication events occurring in humans or other mammals involved in human diseases [19,20] or on the duplication and adaptive evolution of single gene families, such as *chalcone synthase* genes and *MADS-box* genes in plants [21,22], fatty acid biosynthesis genes in bacteria [23], or *Toll-like receptor* genes in *Drosophila*[24]. Although gene gain and loss is estimated with a *Drosophila*-wide perspective [25], a systematic investigation of the genetic character and evolutionary pattern of young duplicate genes across the closely related *Drosophila* species has not been reported.

Sequencing of the genomes of the 12 worldwide *Drosophila* species (*Drosophila* 12 Genomes Consortium 2007) [26] provides the opportunity to reveal the evolutionary genetics of recent duplications. These species capture a range of evolutionary distances: closely related sister-species, such as *D. simulans* and *D. sechellia* or *D. pseudoobscura* and *D. persimilis*; distantly related species classified into different subgenera, such as *Sophophora* and *Drosophila*. There are also cosmopolitan species such as *D. melanogaster* and *D. simulans* or highly restricted species such as *D. sechellia,* distributed in some specific geographic ranges [27]. Additionally, the diverse host preferences provide a way to connect recent duplications with ecological and environmental factors. In this work, we conducted a genome-wide investigation of young duplicate genes across 12 *Drosophila* species to uncover their evolutionary patterns.

## Results

### Young duplicate genes in 12 *Drosophila* genomes

Across the 12 *Drosophila* genomes, a total of 22,488 gene families were detected, including 3,647 young duplicate gene families (see Methods; Table 1), suggesting that approximately 16.2% of the total gene families included young duplicates. In these young duplicate gene families, three types were defined based on their expansion patterns: species-specific expansions, lineage-specific expansions and complex expansions. The species-specific young duplicate gene families clearly predominated (3159/3647 = 86.6%) over the other two types of expansions. On the other hand, uneven distribution of the species-specific young duplicate genes among different species, ranging from 54 to 794, was also observed. For example, *D. melanogaster* had the least family number (54), while the highest three values were found in *D. willistoni* (318), *D. yakuba* (569) and *D. grimshawi* (794), respectively (Table 1). This uneven distribution of the young duplicate genes was also found in the lineage-specific expansions and the complex expansions. For example, 114 duplicate gene families were detected in lineages of *D. persimilis* and *D. pseudobsura*, which is approximately 11.9- and 4.9-fold greater than that in lineages of *D. erecta* and *D. yakuba* or *D. sechellia* and *D. simulans*, respectively. Also as expected, if there are more species (e.g. > 3) in a group of lineage-specific and complex expansions, fewer duplicate gene families were detected.

**Table 1:** Number of young duplicate gene families for three types of expansion

**Species**	**Species-specific expansions**	**Lineage-specific expansions**						**Complex expansions**																				**Total**
		**2**	**2**	**2**	**3**	**5**	**6**	**2**		**3**		**4**		**5**		**6**	**6**	**7**	**8**		**9**		**10**	**11**		**11**	**12**	
*D. simulans*	139	+^a^	-	-	+	+	+	-	…^c^	-	…	+	…	+	…	-	+	+	+	…	+		+	…	+	+	+	
*D. sechellia*	289	+	-	-	+	+	+	-		-		+		+		-	-	-	+		+		-		+	+	+	
*D. melanogaster*	54	- ^b^	-	-	+	+	+	-		-		+		+		+	+	+	+		+		+		+	+	+	
*D. yakuba*	569	-	+	-	-	+	+	+		-		+		-		+	+	+	+		+		+		+	+	+	
*D. erecta*	89	-	+	-	-	+	+	-		-		-		+		+	+	+	+		+		+		+	+	+	
*D. ananassae*	150	-	-	-	-	-	+	-		-		-		+		-	-	-	+		+	…	+		+	+	+	
*D. pseudobsura*	247	-	-	+	-	-	-	-		-		-		-		-	+	+	+		+		+		+	-	+	
*D. persimilis*	232	-	-	+	-	-	-	-		-		-		-		-	+	+	+		+		+		+	+	+	
*D. willistoni*	318	-	-	-	-	-	-	-		-		-		-		+	-	-	-		+		+		+	+	+	
*D. virilis*	118	-	-	-	-	-	-	-		+		-		-		+	-	+	-		-		+		+	+	+	
*D. mojavensis*	160	-	-	-	-	-	-	-		+		-		-		-	-	-	-		-		+		-	+	+	
*D. grimshawi*	794	-	-	-	-	-	-	+		+		-		-		+	-	-	-		-		-		+	+	+	
		29	12	143	11	5	1	21	177	5	44	3	9	1	6	1	1	1	1	3	2	3	1	2	1	2	3	
**Total**	**3159**	**201**						**287**																				**3647**

^a^ Corresponding species is involved in the relevant expansion events.

^b^ Corresponding species is not involved in the relevant expansion events.

^c^ Not all expansion events are showed because of space limitation.

Interestingly, three young duplicate gene families were detected respectively in complex expansions occurring in 11 and 12 species (Table 1). Although the six families were classified as the complex expansion type, species-specific duplication events also were found in all of these families (Additional file 1: Figure S1), especially independent duplications after the species differentiation in many species. For example, 15, 16 and 91 species-specific duplicate clades across all of the 12 species were detected in family 2,419, 7,827 and 8,177, respectively. In the other 3 families, 8, 11 and 145 species-specific duplicate clades were distributed in 8, 11 and 11 species, respectively. In addition, some lineage-specific duplications were also found in these families. All these results suggested that these duplicate gene families were likely to have been shaped by convergent evolution due to independent expansions in many species after the species differentiation.

### Distribution of young duplicate genes on chromosomes

**Figure 1:**
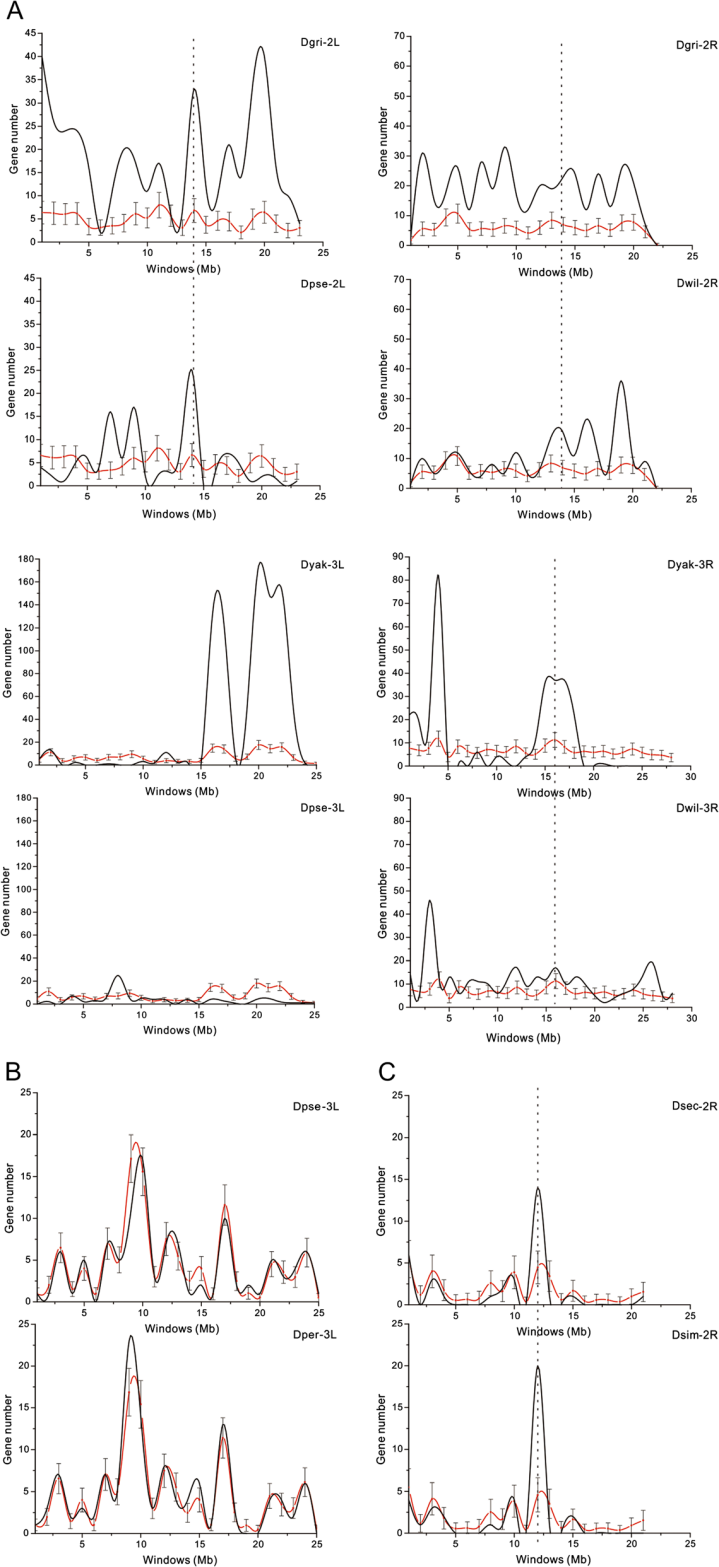
**Distribution of young duplicate genes on chromosomes. (A)** Species-specific duplicates on chromosome 2 and 3. **(B)** Lineage-specific duplicates of *D. pseudoobscura*-*D. persimilis* pair on chromosome 3. **(C)** Duplicates of complex expansions occurring in 11 species on chromosome 2. Black lines represent observations, while black bands and red lines (red dots) show confidence intervals and average numbers of genes in corresponding windows. Dashed lines indicate the shared hotspot regions between the species. Dgri: *D. grimshawi*, Dpse: *D. pseudoobscura*, Dper: *D. persimilis*, Dsec: *D. sechellia*, Dsim: *D. simulans*, Dwil: *D. willistoni*, Dyak: *D. yakuba*; 2L: chromosome 2L, 2R: chromosome 2R, 3L: chromosome 3L, 3R: chromosome 3R.

To explore the distribution of the young duplicate genes on the chromosomes, stochastic simulations were implemented using the observed gene numbers with 10,000 times random repeats. The chromosomal distribution was significantly non-random (*P* < 0.05); for example, chromosome 2 (2L & 2R), 3 (3L & 3R) and X contained a mass of young duplicate genes (Additional file 2: Figure S2). Figure 1 shows graphs representing these simulation results. Furthermore, the windows in Figure 1 with the observed number larger than the upper level of the confidence intervals correspond to hotspot regions on the chromosomes for the young duplications.

As shown in Figure 1, hotspot regions were found in all three types of young duplications, especially in species-specific expansions (Figure 1A), e.g. on chromosome 2 in *D. grimshawi* and on chromosome 3 in *D. yakuba*. In contrast, few hotspot regions were found in species such as in *D. ananassae* and *D. melanogaster*. Interestingly, some duplication hotspot regions were shared by more than one species in the species-specific expansions (marked by dash lines in Figure 1A), also suggesting convergent evolution of these genes among different species. However, none of shared hotspot region were detected in lineage-specific duplications, although the two species had similar trend lines which were generated by the observations and simulation numbers (Figure 1B). In complex expansions, few hotspot regions were detected along the chromosomes (Figure 1C).

### Functional preference of young duplicate genes

**Figure 2:**
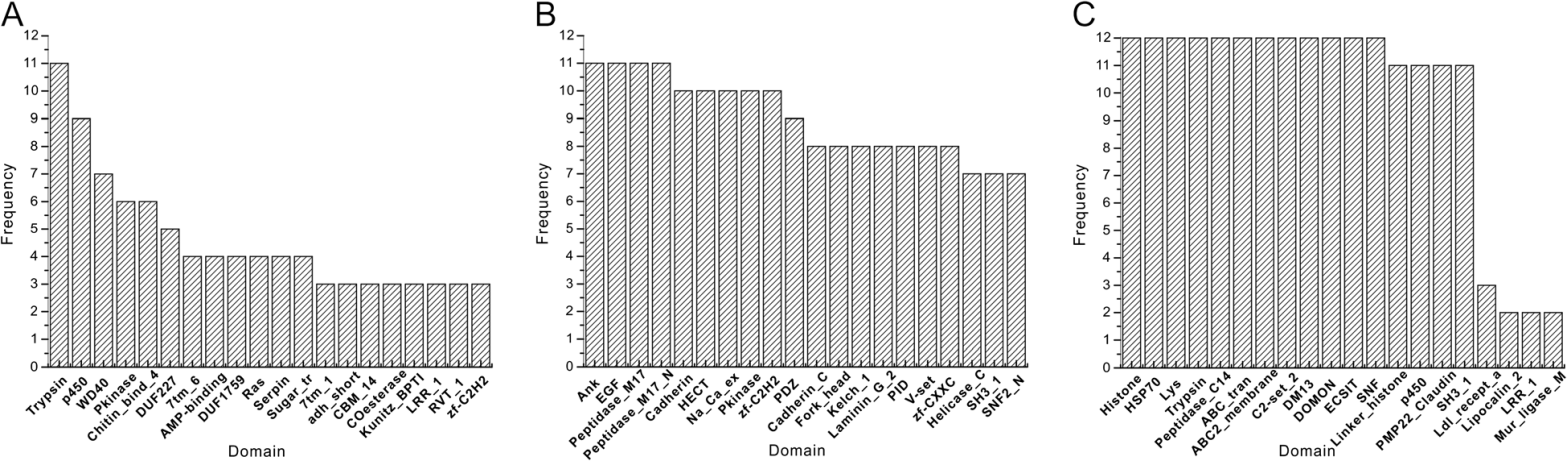
**Occurrence-frequency of the top 20 domains in two types of expansions. (A)** The frequency for species-specific expansions. **(B)** and **(C)** The frequency for complex expansions occurring in 11 and 12 species, respectively.

To further reveal the genetic characteristics of the young duplications, the domains of the duplicates were detected using Pfam searches. Subsequently, the protein domains were counted in each species. For species-specific expansions, a total of 1,277 different domains were found in 12 species, averaging 106 protein domains in each species (Additional file 3: Table S1). Interestingly, approximately 84% of protein domains occurred only once or twice, suggesting that most domains were unique. However, the frequency of some protein domains, such as DUF1676 in *D. willistoni*, annexin and dynein_IC2 in *D. melanogaster*, inositol_P and PAP2 in *D. yakuba*, were high in one species but low (0 or 1) across the other 11 species, suggesting that these species-specific duplicate proteins might be driven by adaptive evolution in each species. Furthermore, some protein domains occurred in a lineage-specific manner, although they were detected in the species-specific expansion events. For example, the expansion of domains Gb3_synth and Gly_transf_sug shared by *D. mojavensis* and *D. virilis*, were greatly expanded only in these two species. A similar situation was also observed in the alpha-amylase domain, which occurred in two closely related *Drosophila* species, *D. sechellia* and *D. simulans*. Although different types and numbers of protein domains were examined in each species, we still found that approximately 4% of the domains appeared simultaneously in ≥ 6 species. Prominent examples of these protein domains were trypsin, p450 and WD40, which were detected in 12, 11 and 11 species, respectively (Additional file 3: Table S1). These proteins are all important in response to environmental stimuli [28,29]. To investigate whether the high-frequency domains also occupied in large numbers in each species or vice versa, we examined the occurrence-frequency of the top 20 domains in 12 genomes. Interestingly, these high-frequency domains also had a large number of copies in the related species (Figure 2A), suggesting that these high-frequency duplicated proteins play an important role in the evolution of these species.

An identical approach was also used for the gene families of lineage-specific expansions and complex expansions. Our results showed that most gene families contained limited protein domains, although the number of the same domain was always different. However, some protein domains were still undergoing rapid expansion independently in many species, e.g. the six shared duplicate gene families in complex expansions occurring in 11 and 12 species (Table 1; Figure 2B and C). Furthermore, Ank, EGF, Peptidase_M17 and Peptidase_M17_N, which were all conserved and widespread domains in organisms for survival, exhibited high frequencies in 11 species (Figure 2B). In the shared expansion events of 12 species (Figure 2C), 12 of the top 20 protein domains such as histones, HSP70, Lys, co-occurred in 12 species. Numerous previous studies have shown that these protein domains are closely related to stress responses and pathogens in the environment, for example, histones are involved in stress responses [30], HSP70 protects cells from stress [31], and Lys (lysozyme) acts as a bacteriolytic enzyme by hydrolyzing bacterial cell walls [32], suggesting that these shared duplications play an important role in adaption to ecological factors and environmental changes in *Drosophila*.

### Nonsynonymous and synonymous substitution between paralogs and orthologs

The ratio of nonsynonymous to synonymous nucleotide substitution (*Ka*/*Ks*) is considered as an important parameter indicating the strength of functional constraints. The smaller the *Ka*/*Ks* ratio is, the stronger the functional constraints are. The 12 *Drosophila* whole-genome data offer us unprecedented opportunity to explore the different selection pressure between paralogs and orthologs. Therefore, we examined *Ka*/*Ks* ratios for paralogs and orthologs in each duplicate gene family.

**Figure 3:**
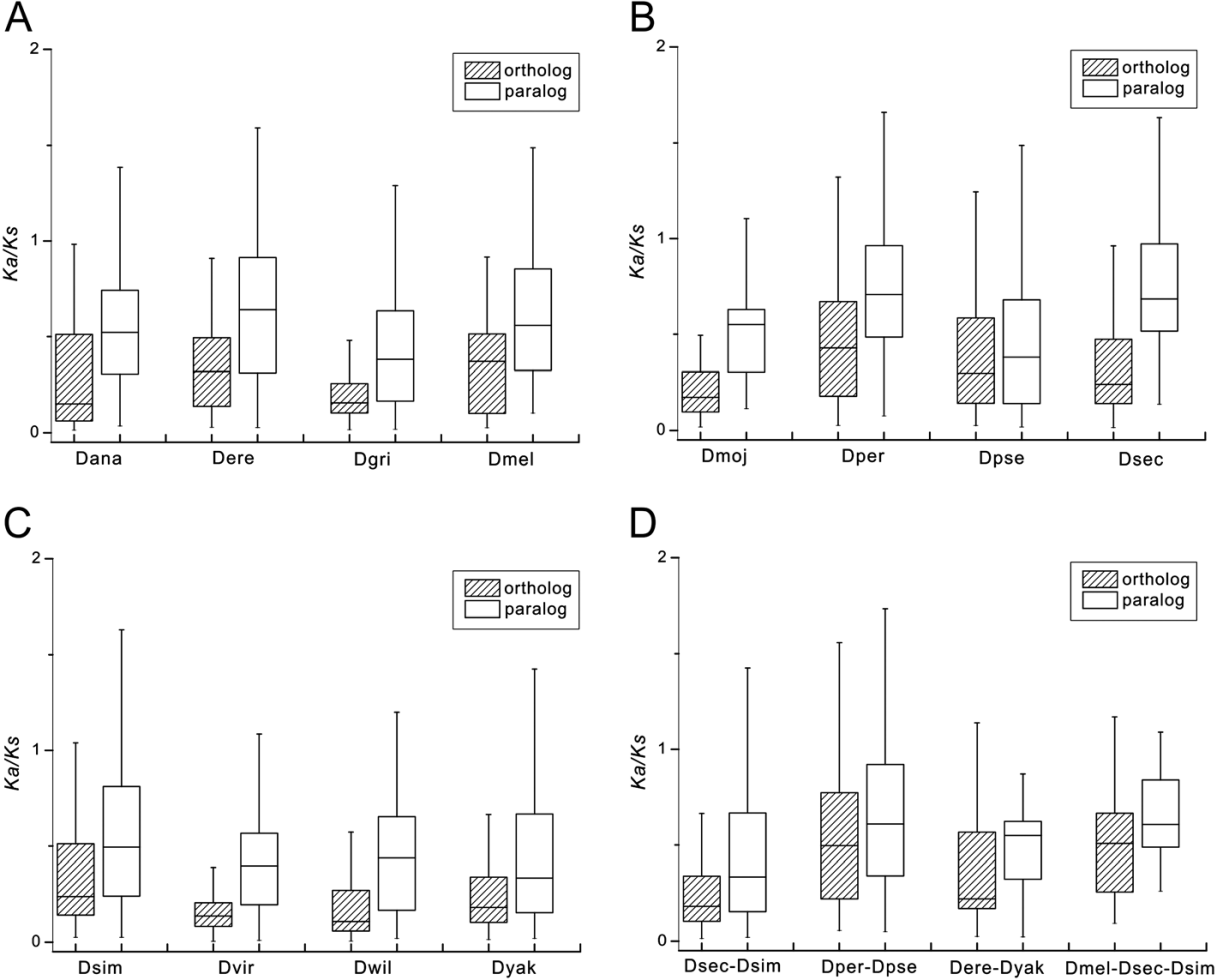
**Box plot comparing the mean *Ka/Ks *ratios of paralog gene pairs and ortholog gene pairs. (A)**, **(B)** and **(C)***Ka/Ks* ratios of species-specific duplicates. **(D)***Ka/Ks* ratios of lineage-specific duplicates. The top bar is maximum observation, the lower bar is minimum observation, the top of box is third quartile, the bottom of box is lower or first quartile, the middle bar in box is median value.

The average *Ka/Ks* between paralog gene pairs or ortholog gene pairs in these young duplicate gene families were 0.626 and 0.445, respectively, which was significantly (*P* < 0.05) larger than the genome-wide *Ka/Ks* (0.218) between ortholog pairs, suggesting relaxation of the functional constraints in the young duplicate gene families. Figure 3 shows that most of the gene pairs (91.2%), including paralogs and orthologs, had *Ka*/*Ks* ratios less than 1, demonstrating that most young duplicate genes were under purifying selection. However, there were still 229 and 82 gene pairs with *Ka*/*Ks* ratios greater than 1 for paralogs and orthologs, respectively, indicating that some young duplicate genes are driven by positive selection. However, in the gene pairs with *Ka*/*Ks* values exceeding 1, many values were just slightly greater than 1 and only few pairs were detected to have *Ka*/*Ks* ratios significantly greater than 1.

Based on the strengths of boxes and whisker lines in species-specific expansions in Figure 3, it was clear that *Ka/Ks* between paralogs had a broader dispersed distribution, larger median and quartile values than orthologs, indicating that paralogs had higher *Ka/Ks* than orthologs. Similar results were also obtained in lineage-specific expansions (Figure 3D), with the exception of *D. sechellia* vs. *D. simulans* and *D. persimilis* vs. *D. pseudoobscura*. To further ensure that the *Ka/Ks* of paralogs were significantly greater than those of orthologs, we conducted paired *t*-tests. Apart from four pairs, the other *Ka/Ks* ratios of paralogs and orthologs exhibited highly significant (*P* < 0.01) or significant (*P* < 0.05) differences (Additional file 4: Table S2). All the results showed that paralogs had significantly higher *Ka/Ks* than orthologs and indicated that paralogs are subject to weaker functional constraints and faster evolutionary processes than orthologs.

Linear analog was also performed between the mean *Ka/Ks* of paralogs and orhtologs (Additional file 5: Figure S3). In the same family, the dot above the trend lines (slope = 1) indicated that paralogs have higher evolutionary rates than orthologs. Interestingly, it was clear that some dots lay far away the trend lines. Detection of the protein domains of these dots (Additional file 4: Table S2) showed that most of the domains detected in the genes of upper dots, such as Coesterase [33,34], Turandot [35] and MIP [36] were involved in stress responses. These results also suggested that the young duplicates result from adaption to the environment both in species-specific and lineage-specific expansions.

### Evolutionary analysis of young duplicate genes across 12 *Drosophila* species

To detect the timing of recent duplication in each species, the *Ks* values were calculated. We adopted the common assumption that *Drosophila* species experienced about 10 generations/year and that the single nucleotide mutation rate was 5.8 ×10^-9^ mutations per generation [37]. Furthermore, only *Ks* values lower than 1.0 were kept to avoid the saturation of nucleotide substitutions.

On the whole, the young duplication events occurred over a short span of time (0.082-5.282 MYA). The duplication time of species-specific expansions fell in a range from 1.238 MYA (*D. simulans*) to 3.573 MYA (*D. melanogaster*) (standard deviation, 0.712) (Table 2), indicating that all the species-specific expansions occurred within a short time. Moreover, most of our estimates of duplication time were less than the species divergence time reported by Tamura [38]. However, the divergence time of several closely related species previously reported, including *D. simulans* vs. *D. sechellia* (< 0.93 ± 0.49 MYA) and *D. pseudoobscura* vs. *D. persimilis* (0.85 ± 0.29 MYA) was slightly lower than their respective family duplication times (1.238, 2.313 and 1.327, 1.573 MYA). Similarly, higher duplication times in the four species were also found in lineage-specific expansions and complex expansions. Moreover, the lowest standard deviations of the duplication time were detected between these lineage species in lineage-specific expansions, which suggested that closely related species duplicated in close periods, especially the species pair *D. persimilis* vs. *D. pseudoobscura* (2.341 and 2.401 MYA). In the six lineage species, there was a group of duplication times with more compact distribution and smaller values than those in species-specific expansions, which indicated that the expansion occurred over a more concentrated and closer period in lineage-specific expansions than in species-specific expansions. It was clear that less species and closer relationships caused such results. Finally, although the highest standard deviations were found in complex expansions, especially in 11 species with a broader range for duplication time (0.765–5.282 MYA) and a larger standard deviation (1.686) than those in others, their distributions of duplication time were still in relative compact period. This demonstrated that these duplicated genes in complex expansions might appear at relatively scattered time compared with duplicates in the other two types. Therefore, we might infer that *Drosophila* species have consistently duplicated to adapt to environmental changes.

**Table 2:** Duplication time for species-specific expansions, lineage-specific expansions and complex expansions

**Species**	**Species-specific expansions**	**Lineage-specific expansions**						**Complex expansions**		
*D. simulans*	1.238	1.561	-	-	1.492	1.916	0.981	2.763	1.531	1.655
*D. sechellia*	2.313	2.249	-	-	1.281	1.575	0.084	2.576	0.765	0.525
*D. melanogaster*	3.573	-	-	-	1.022	0.769	0.998	0.674	1.169	3.794
*D. yakuba*	1.613	-	3.526	-	-	0.641	0.564	4.843	1.644	2.549
*D. erecta*	1.874	-	2.775	-	-	0.539	0.082	5.131	1.287	0.760
*D. ananassae*	2.572	-	-	-	-	-	0.582	3.372	5.282	1.244
*D. pseudoobscura*	1.327	-	-	2.401	-	-	-	4.108	-	3.790
*D. persimilis*	1.573	-	-	2.341	-	-	-	3.268	1.017	4.490
*D. willistoni*	2.085	-	-	-	-	-	-	2.419	2.560	1.473
*D. virilis*	2.513	-	-	-	-	-	-	3.509	5.153	1.003
*D. mojavensis*	3.388	-	-	-	-	-	-	-	1.332	1.023
*D. grimshawi*	2.160	-	-	-	-	-	-	2.162	4.755	0.449
Standard deviation	0.712	0.344	0.375	0.030	0.192	0.553	0.371	1.207	1.686	1.349

## Discussion

### Convergent evolution of young duplicate genes across the 12 *Drosophila* species

Convergent evolution plays an important role in biological adaptation, by which distantly related organisms independently evolve similar structures or functions in order to adapt to similar environments or ecological niches [39], such as, the specialized oxygen transport function of oxygen transport hemoglobins in jawed and jawless vertebrates [40] and the similar substrate of apolipoprotein (a) in humans and hedgehogs [41]. Although there are many other theories could explain the evolutionary process of young duplicates, such as genomic drift proposed by Nei [42,43], convergent evolution might be more convincible for two evidences detected in our study.

In our study, the phylogenetic trees (Additional file 1: Figure S1) and the chromosomal distributions (Figure 1) of young duplicate genes also provide evidence of convergent evolution. Six young duplicate families were found in complex expansions occurring in 11 or 12 species with many species-specific duplication clades across these 11 or 12 species. Interestingly, the phenomenon that the independent duplicates with similar function preference are under convergent evolution has also been previously reported both in animals and plants. For example, histone proteins are highly alkaline proteins in eukaryotic genomes which package DNA into nucleosomes [44] and independent convergent evolution has produced striking similarities between plant and animal histones [45]. Another example of similar genetic characteristics shared by distant species is the digestion function of lysozymes (Lys domain) in animals. Lysozymes are usually present in tears, saliva and other bodily fluids and have independently been recruited to the stomach and play important roles in enzyme functions across vertebrates [46]. Furthermore, some duplication hotspot regions were shared by more than one species across their chromosomes in species-specific expansions. Interestingly, conserved duplication hotspots have also been previously detected between *D. melanogaster* and *D. simulans*[47]. Similar function preference and identical hotspot regions arising from independent duplications suggest that the young duplicate genes have undergone convergent evolution which appears to have played an important role in the independent evolution of adaptive traits in 12 *Drosophila* species.

### Adaptive evolution supported by functional bias of young duplicates

It is well-known that duplicate genes face three possible fates: pseudogenization, subfunctionalization and neofunctionalization. Pseudogenization is considered as the most common fate of duplicate genes [8-10], but more evidence support the models of subfunctionalization or neofunctionalization, as the mechanisms for the preservation of duplicate genes under adaptive selection [6,15,48,49]. Many previous studies have shown that the duplicated genes could adapt to various conditions, in particular, genes encoding membrane or secreted proteins which are always involved in ecological stimuli or stress. For example, adaptive gene duplications have been found in response to biotic stress [50], antibiotics [51,52], weedicides [53] or pesticides [54,55], drugs or toxins [56], extreme temperatures [57,58], nutrient limitation [59,60] and symbiosis between host and parasite [61].

In this study, it was shown that the protein domains of trypsin, p450, WD40 and Pkinase in species-specific expansions, Ank, EGF, histone, HSP70 and Lys in complex expansions occurred with high frequency across the 12 *Drosophila* species. Interestingly, these young duplicates were also involved in different aspects of interactions with the environment. Trypsin is one of the largest families of secreted serine proteases found in the digestive system of vertebrates and invertebrates. Although it participates in many basic physiological processes [62-64], it is predominantly involved in diet and digestion. The high frequency of trypsin across 12 *Drosophila* species indicated that consistent and independent duplication for adaptation to specific dietary habits was due to their diverse ecosystems [27,29]. For example, investigations of trypsin family conducted in various genomes, such as fruit fly [65], mosquitoes [66] and leaf-eating monkey [67], have all indicated that adaption occurs in response to specific diets. In particular, researches into the rapid diversification of trypsin genes in 12 *Drosophila* species have provided insights into the ecological forces driving the adaptive evolution by comparing the relationship between duplications and host preference shifts [65].

Another protein domain shared between 11 *Drosophila* species detected in this study was cytochrome p450 (CYP). P450 comprise a superfamily of enzymes that occurs with a high degree of diversity in all organisms [68]. Among the various biological functions of p450, we focused on the oxidation of xenobiotic compounds, which facilitates their excretion from the organism [69,70]. Abuse of insecticides has forced adaptive evolution in *Drosophila* over an extremely short period. A single p450 gene, *Cyp6g1*, is sufficient and necessary for DDT resistance [28] and its cross-resistance to a wide range of other insecticides has also been identified in *Drosophila*[71,72]. Furthermore, functional divergence and positive selection detected in mammalian CYP genes, provide insights into the adaptive selection of CYPs in response to high diversity of xenobiotics [73].

Other expanded domains were also identified with roles in adaption to various ecological factors, especially stress. For example, some SAPK (stress-active protein kinases, Pkinase) mediate cellular responses to toxins and physical stress [74] and TAK1 (transforming growth factor-β-activate kinase, Pkinase) is a key regulator in response to diverse stimuli in adaptive immunity [75], ankyrin proteins (Ank) play a role in stress responses and disease resistance both in animals [76] and plants [77], histones [30] and HSP70 proteins protect cells from stress [31], and Lys proteins act as bacteriolytic enzymes by hydrolyzing cell bacterial walls [32].

These observations indicate that shared young duplications reflect adaptive evolution of the *Drosophila* species to global ecological pressures.

### Adaptive evolution contributes to specific functional preference

In this study, although most paralogs and orthologs of these young duplicate gene families had *Ka/Ks* ratios lower than 1, some *Ka/Ks* ratios greater than 1 were also found both in species-specific and lineage-specific expansions (Figure 3), demonstrating that they were under adaptive selection. Furthermore, paralog gene pairs had higher *Ka/Ks* ratios than ortholog gene pairs across 12 *Drosophila* species. It can be concluded that the paralogs have higher frequency of adaptive evolution than the orthologs [48]. Previous research has indicated that many genes families in *Drosophila* are driven by adaptive selection, such as, elastase/chymotrypsin, trypsin and astacin, which are all involved in digestive processes in *D. arizonae*[26], two immunity-related gene families, Toll-like receptors and lysozyme in *D. melanogaster*[24,78], and *metallothionein* genes involved in metal tolerance [79,80]. Moreover, positive selection is a major force driving the evolution of male-specific recent duplicates on neo-X chromosome in *D. pseudoobscura*[17] and segmentally duplicated seminal fluid genes in *D. melanogaster*[81].

Functional analysis of those gene families in which paralogs had higher *Ka/Ks* ratios than orthologs with ratios exceeding 1 (Figure 3) showed that adaptive evolution leads to species-specific and lineage-specific functional preference for each *Drosophila* (Additional file 3: Table S1). Examples include the PDZ-domain containing gene family in *D. sechellia*, stress-inducible humoral factor Turandot (Turandot domain) in *D. yakuba* and the Pam16 family (Pam16 domain) in the *D. pseudoobscura* and *D. persimilis* pair. Combining the functional preference with the host preference of corresponding *Drosophila* species [27], it seems reasonable to infer that *Drosophila* evolve for adaption to a given environment [82].

Interestingly, we found that *D. sechellia* only distribute in the Seychelle Islands in the Indian Ocean and prefer inhabiting *Morinda citrifolia*, the fruit of which contain nutrients such as alkaloids. Alkaloids are widely known that play an important role in inhibiting tumors by reducing microtubules disruption during mitosis [83]. Coincidently, PDZ proteins are involved in the interaction between syntrophin-associated serine/threonine kinase and microtubule-associated serine/threonine kinases and are recognized as tumor suppressors [84,85]. Therefore, we speculated that duplication of PDZ in *D. sechellia* is closely associated with adaption to this unique habitat.

The *D. yakuba* species in Africa exhibited propagation of Turandot proteins which are adaptively resistant to high temperature, dehydration and UV irradiation [86,87]. In contrast, the Pam16 proteins of *D. pseudoobscura* and *D. persimilis* play important regulatory roles in recruiting heat shock protein partners and responding to cold hardening [88-90], indicating superior adaptation of these species to their specific habitats situated in the regions of higher latitude in the Northern Hemisphere compared with other species.

Furthermore, other evidence of adaptive evolution in a single species or lineage species pairs was also detected in domain analysis (Additional file 3: Table S1). For example, annexin and dynein_IC2 in *D. melanogaster*, which are sperm-specific proteins (annexin X and cytoplasmic dynein intermediate chain) and absent in other species of the *melanogaster* species subgroup, support the hypothesis that male reproductive functions are driven by selective sweep and rapid molecular evolution [91]. Another example is alpha-amylase (Amy), a digestive enzyme of the two closely related species of the 12 *Drosophila*, *D. sechellia* and *D. simulans*, for which genetic variation of duplicated amylase genes has been reported revealing adaptive evolution in *Drosophila*[92].

Consequently, adaptive evolution of *Drosophila* species leads to young duplicate genes exhibiting specific function preference in response to ecological factors and environmental changes.

## Conclusions

We identified 3,647 young duplicate gene families across 12 *Drosophila* species and detected three types of expansions in these gene families: species-specific, lineage-specific and complex expansions. We found that the species-specific young duplicate genes predominated (86.6%) over the other two types. Furthermore, we observed that, in the same gene family, independent species-specific expansions occurred in many species, even including 11 or 12 *Drosophila* species, suggesting that these young duplicate genes were under convergent evolution after the *Drosophila* species differentiation. We also found that the functional preference of the young duplicate genes was mainly related to responses to environmental stimuli and biotic stresses, suggesting that adaptive evolution may play an important role in duplicate genes for adaption to ecological factors in *Drosophila* species. This work may help us to better understand the evolutionary patterns of young duplicate genes across 12 *Drosophila* species.

## Methods

### Identification of young duplicate genes

The 12 *Drosophila* genome sequences and annotations were downloaded from the Flybase Datebase [http://ftp.flybase.net/genomes/] [93], and the exact version for each species is shown in Table 1 (Additional file 4). An all-versus-all Blastn search with E-value (1.0e-40) was processed across all nucleotide coding sequences (CDSs) in 12 *Drosophila* species, then coverage of > 60% was used to divide the genes into gene families. Subsequently, Clustalw2.0 [94] was used for the pairwise alignment of the nucleotide sequences of genes in one family and the nucleotide diversity (π) was estimated with the Jukes and Cantor correction by MEGA v5.0 [95]. Young duplicate gene families were defined based on the following two conditions: (1) the number of family members was larger than the number of corresponding species in each family; (2) the highest identity of the paralogs in each family exceeded 90%.

To further analyze young duplicate gene families, three types of expansions were defined: species-specific expansions, lineage-specific expansions and complex expansions. Here, the species-specific expansions were denoted as young duplications occurring only in one species, while other species comprised ≤ 1 member or > 2 members but with less than 90% identity between paralogs. We also defined the latter two types as the young duplications of a gene family in species with a close (lineage-specific expansion) or distant (complex expansion) genetic relationship based on the species tree of the 12 *Drosophila* species [27]. Based on this principle, species-specific expansions were easily identified corresponding to each species. Furthermore, we defined the following six lineage-specific expansions: *D. sechellia*-*D. simulans*, *D. yakuba-D. erecta*, *D. pseudoobscura-D. persimilis*, *D. melanogaster-D. sechellia*-*D. simulans*, *D. melanogaster-D. sechellia*-*D. simulans-D. yakuba-D. erecta* and *D. melanogaster-D. sechellia*-*D. simulans-D. yakuba-D. erecta-D. ananassae*, and 14 complex expansions (Table 1 & Additional file 6: Table S4).

### Sequence alignment and phylogenetic analysis

The amino acid sequences of the duplicate genes in each family were first aligned using the MUSCLE program with default options and then manually corrected using MEGA v5.0 [95]. The alignments were used to construct phylogenetic trees with 1,000 replicates using MEGA v5.0 based on the neighbor-joining (NJ) method [96]. NJ analysis was conducted using pairwise deletion of gaps and kimura-2 model (family3093, family9588 and family7827) or p-distance (family8177). Additionally, for the two families (family21 and family2419) with numerous members, NJ trees were constructed with default parameters and 1,000 bootstrap replicates in Clustalw2.0 [94].

### Physical location and structural domains of the young duplicate genes

The hotspot regions for duplication events were examined by identifying the exact physical positions of young duplicate genes across the chromosomes. Detailed genome annotation information was available for the genes of *D. melanogaster* only. Thus, we performed Blast analysis using the CDSs of young duplicate genes in the other 11 genomes against the genome sequences of *D. melanogaster* to gain the position information.

We utilized the position information to process stochastic simulations with 10,000 random repeats by PERL script. Each chromosome was divided into a number of windows (1Mb/1 Window). We incorporated all genes of corresponding species into the given window of each chromosome for the species-specific duplicate gene families, and an identical approach was taken for the families of lineage-specific expansions and complex expansions. All young duplicate genes identified in this study were further examined for structural domains using the Pfam database [Pfam database 26.0, http://pfam.sanger.ac.uk/] [97] with E-value 1.0.

### Calculation of nonsynonymous to synonymous ratio and estimation of duplication time of paralogs

CDSs in each young duplicate gene family were aligned according to alignments of protein sequences in Clustalw2.0 [94]. Subsequently, the nonsynonymous substitutions (*Ka*), synonymous substitutions (*Ks*) and ratio of nonsynonymous to synonymous substitutions (*Ka*/*Ks*) were calculated for paralog and ortholog pairs in each duplicate gene family using MEGA v5.0 [95].

The mean *Ks* values were calculated for paralog pairs in each species-specific duplicated gene family and then used to estimate the timing of duplications. The calculations were performed using a single nucleotide mutation rate of 5.8 × 10^-9^ mutations per generation and it was assumed that *Drosophila* species experienced approximately 10 generations/year [37].

## Abbreviations

CDS: Nucleotide coding sequences; MYA: Million years ago.

## Competing interests

The authors declare that they have no competing interests.

## Authors’ contributions

DT, SY and YZ designed the study. YZ and YJ contributed extensively to the bioinformatic analyses. YZ, XZ and YG wrote the manuscript. SY, XZ and YZ prepared and revised the manuscript. All authors read and approved the final manuscript.

## Supplementary Material

Additional file 1: Figure S1 Phylogenetic trees of the six gene families of complex expansions occurring in 11 and 12 species. Dana: *D. ananassae*, Dere: *D. erecta*, Dgri: *D. grimshawi*, Dmoj: *D. mojavensis*, Dper: *D. persimilis*, Dpse: *D. pseudoobscura*, Dsec: *D. sechellia*, Dsim: *D. simulans*, Dvir: *D. virilis*, Dwil: *D. willistoni*, Dyak: *D. yakuba*. Genes without abbreviative species name are from *D. melanogaster*. Click here for file

Additional file 2: Figure S2 Distribution of young duplicate genes on chromosomes. **(A)**-**(E)** Species-specific duplicates on chromosome 2L, 2R, 3L, 3R and X, respectively. (F) Lineage-specific duplicates of *D. pseudoobscura-D. persimilis* pair. **(G)**-**(K)** Duplicates of complex expansions occurring in 11 species on chromosome 2L, 2R, 3L, 3R and X, respectively. Black lines represent observations, while black bands and red lines (red dots) show confidence intervals and average numbers of genes in corresponding windows. Dmel: *D. melanogaster*. 2L: chromosome 2L, 2R: chromosome 2R, 3L: chromosome 3L, 3R: chromosome 3R. Click here for file

Additional file 3: Table S1 Number of protein domains for young duplicate genes in species-specific expansions. Click here for file

Additional file 4: Table S2 Structural domains detected in families corresponding to the dots excessively deviating from trend lines. **Table S3.** Versions of genome sequences and annotations for each species. Click here for file

Additional file 5: Figure S3 Mean *Ka/Ks* ratios of paralog gene pairs vs. ortholog gene pairs. **(A) ***Ka/Ks* ratios of species-specific duplicates. **(B) ***Ka/Ks* ratios of lineage-specific duplicates. Black lines means the trend line of black dots and red lines represents trend lines with slope = 1. Click here for file

Additional file 6: Table S4 Number of young duplicate gene families for three types of expansion. Click here for file
