# Supplementary material for: Functional requirements driving the gene duplication in 12 Drosophila species
Source: BMC Genomics. 2013 Aug 15;14:555. doi: 10.1186/1471-2164-14-555 (PMC3751352; doi:10.1186/1471-2164-14-555)
Supplement: Additional file 1: Figure S1 — Phylogenetic trees of the six gene families of complex expansions occurring in 11 and 12 species. Dana: D. ananassae, Dere: D. erecta, Dgri: D. grimshawi, Dmoj: D. mojavensis, Dper: D. persimilis, Dpse: D. pseudoobscura, Dsec: D. sechellia, Dsim: D. simulans, Dvir: D. virilis, Dwil: D. willistoni, Dyak: D. yakuba. Genes without abbreviative species name are from D. melanogaster. [file 1471-2164-14-555-S1.pdf]

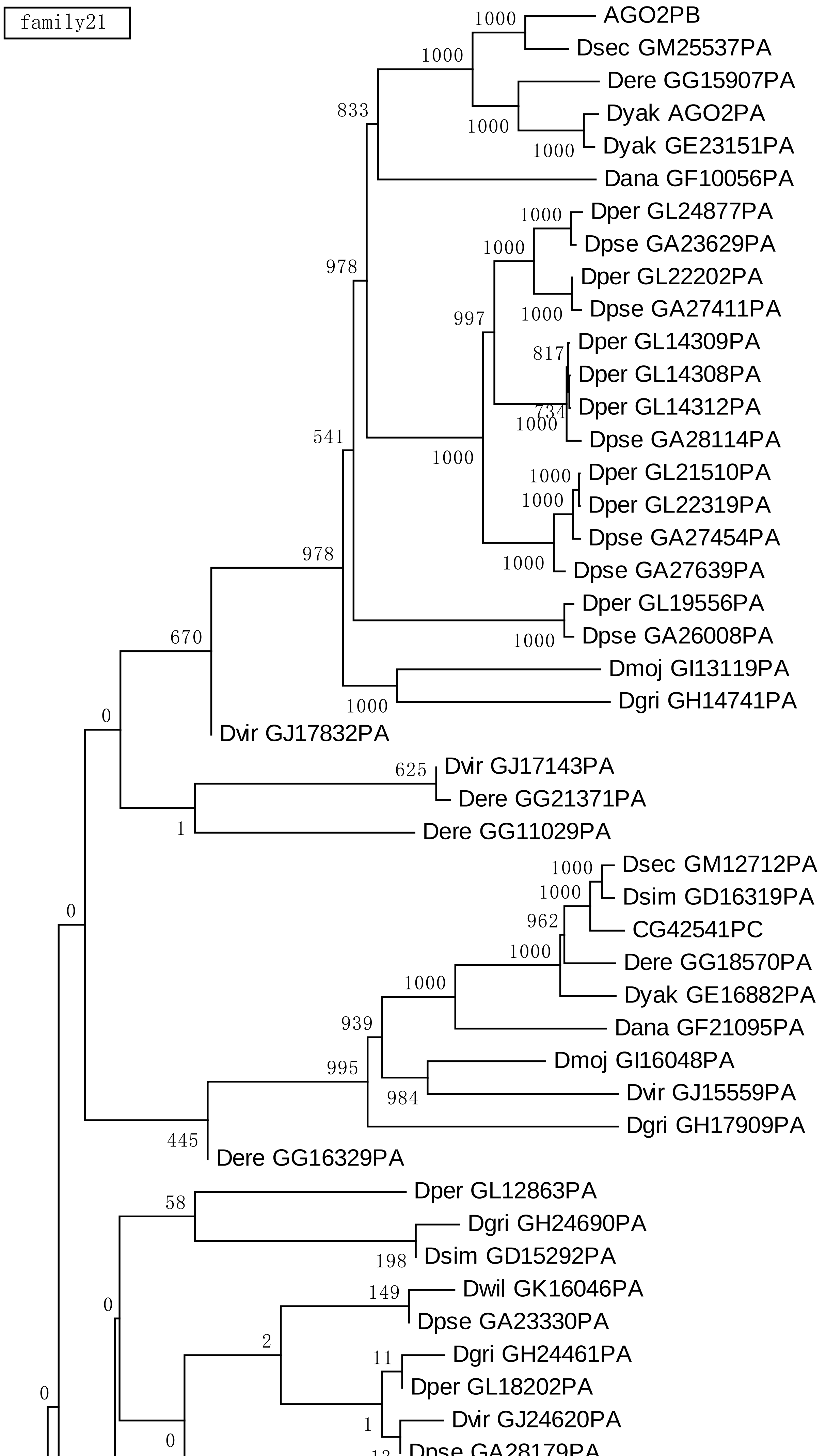
family21
AGO2PB
Dsec GM25537PA
Dere GG15907PA
Dyak AGO2PA
Dyak GE23151PA
Dana GF10056PA
Dper GL24877PA
Dpse GA23629PA
Dper GL22202PA
Dpse GA27411PA
Dper GL14309PA
Dper GL14308PA
Dper GL14312PA
Dpse GA28114PA
Dper GL21510PA
Dper GL22319PA
Dpse GA27454PA
Dpse GA27639PA
Dper GL19556PA
Dpse GA26008PA
Dmoj GI13119PA
Dgri GH14741PA
Dvir GJ17832PA
Dvir GJ17143PA
Dere GG21371PA
Dere GG11029PA
Dsec GM12712PA
Dsim GD16319PA
CG42541PC
Dere GG18570PA
Dyak GE16882PA
Dana GF21095PA
Dmoj GI16048PA
Dvir GJ15559PA
Dgri GH17909PA
Dere GG16329PA
Dper GL12863PA
Dgri GH24690PA
Dsim GD15292PA
Dwil GK16046PA
Dpse GA23330PA
Dgri GH24461PA
Dper GL18202PA
Dvir GJ24620PA
1000
1000
833
1000
1000
978
1000
1000
997
1000
817
734
1000
541
1000
1000
1000
978
1000
670
1000
1000
0
625
1
1000
1000
962
0
1000
1000
939
995
984
445
58
198
0
149
2
11
0
1
0

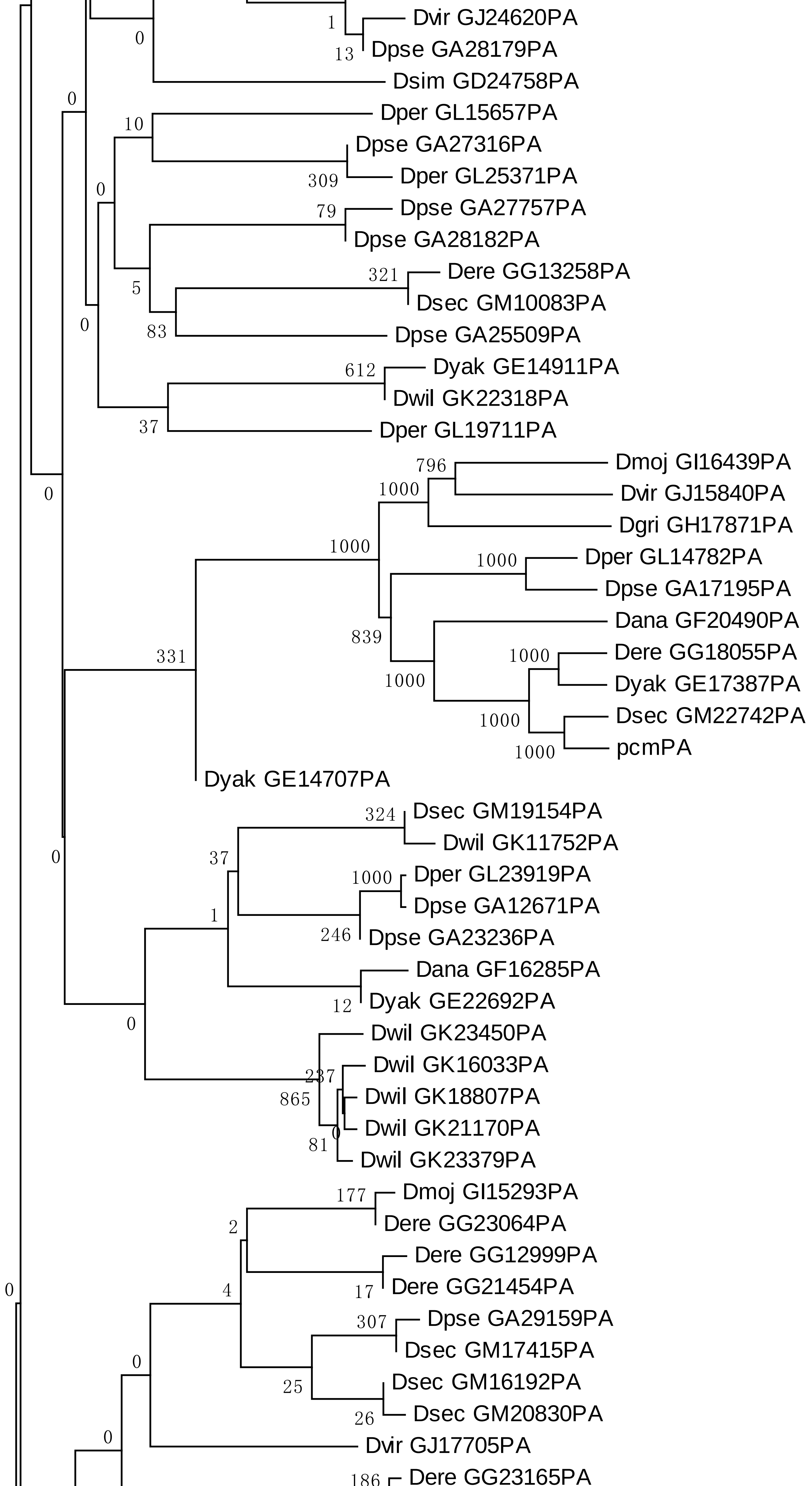

Dvir GJ24620PA
Dpse GA28179PA
Dsim GD24758PA
Dper GL15657PA
Dpse GA27316PA
Dper GL25371PA
Dpse GA27757PA
Dpse GA28182PA
Dere GG13258PA
Dsec GM10083PA
Dpse GA25509PA
Dyak GE14911PA
Dwil GK22318PA
Dper GL19711PA
Dmoj GI16439PA
Dvir GJ15840PA
Dgri GH17871PA
Dper GL14782PA
Dpse GA17195PA
Dana GF20490PA
Dere GG18055PA
Dyak GE17387PA
Dsec GM22742PA
pcmPA
Dyak GE14707PA
Dsec GM19154PA
Dwil GK11752PA
Dper GL23919PA
Dpse GA12671PA
Dpse GA23236PA
Dana GF16285PA
Dyak GE22692PA
Dwil GK23450PA
Dwil GK16033PA
Dwil GK18807PA
Dwil GK21170PA
Dwil GK23379PA
Dmoj GI15293PA
Dere GG23064PA
Dere GG12999PA
Dere GG21454PA
Dpse GA29159PA
Dsec GM17415PA
Dsec GM16192PA
Dsec GM20830PA
Dvir GJ17705PA
Dere GG23165PA

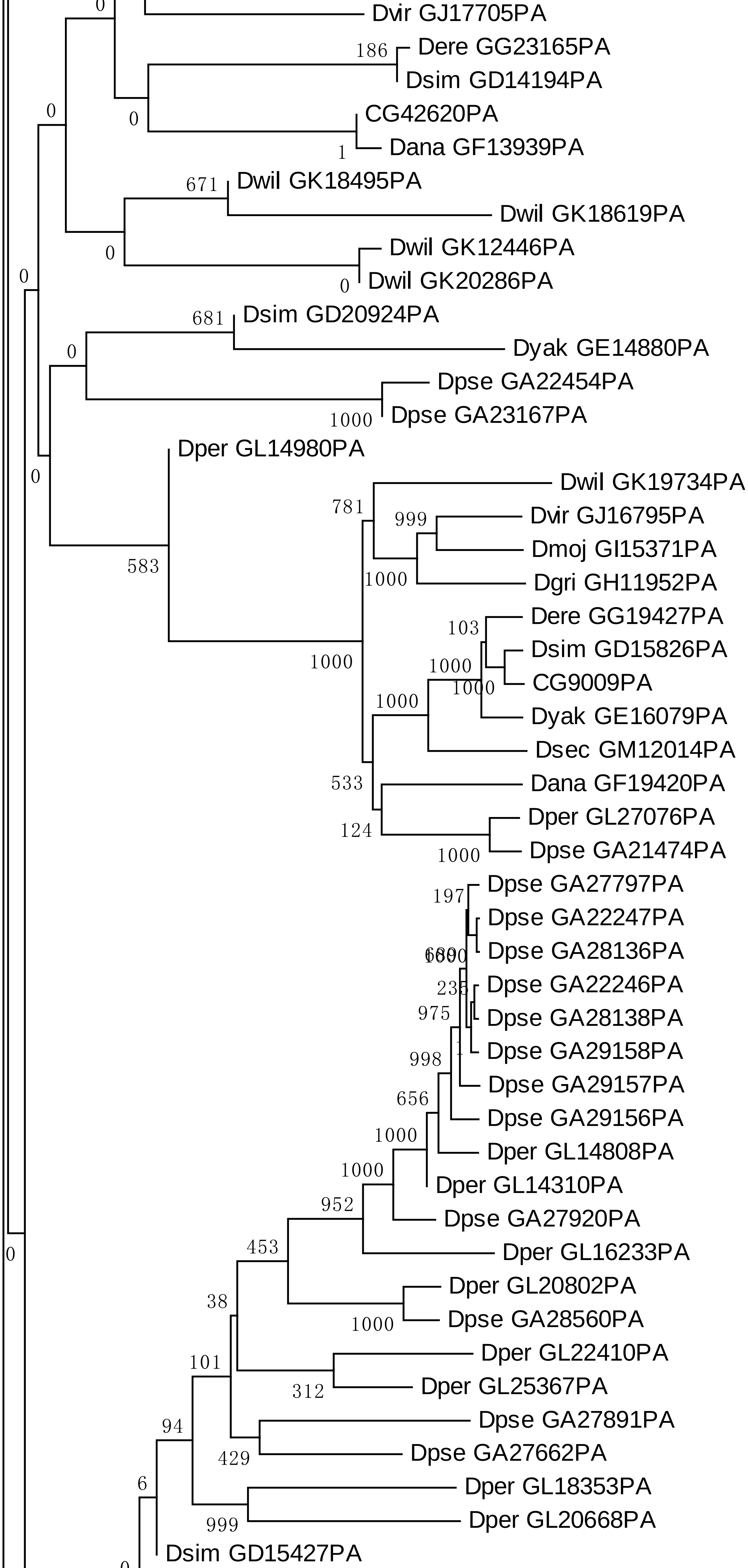

Dvir GJ17705PA
Dere GG23165PA
Dsim GD14194PA
CG42620PA
Dana GF13939PA
Dwil GK18495PA
Dwil GK18619PA
Dwil GK12446PA
Dwil GK20286PA
Dsim GD20924PA
Dyak GE14880PA
Dpse GA22454PA
Dpse GA23167PA
Dper GL14980PA
Dwil GK19734PA
Dvir GJ16795PA
Dmoj GI15371PA
Dgri GH11952PA
Dere GG19427PA
Dsim GD15826PA
CG9009PA
Dyak GE16079PA
Dsec GM12014PA
Dana GF19420PA
Dper GL27076PA
Dpse GA21474PA
Dpse GA27797PA
Dpse GA22247PA
Dpse GA28136PA
Dpse GA22246PA
Dpse GA28138PA
Dpse GA29158PA
Dpse GA29157PA
Dpse GA29156PA
Dper GL14808PA
Dper GL14310PA
Dpse GA27920PA
Dper GL16233PA
Dper GL20802PA
Dpse GA28560PA
Dper GL22410PA
Dper GL25367PA
Dpse GA27891PA
Dpse GA27662PA
Dper GL18353PA
Dper GL20668PA
Dsim GD15427PA

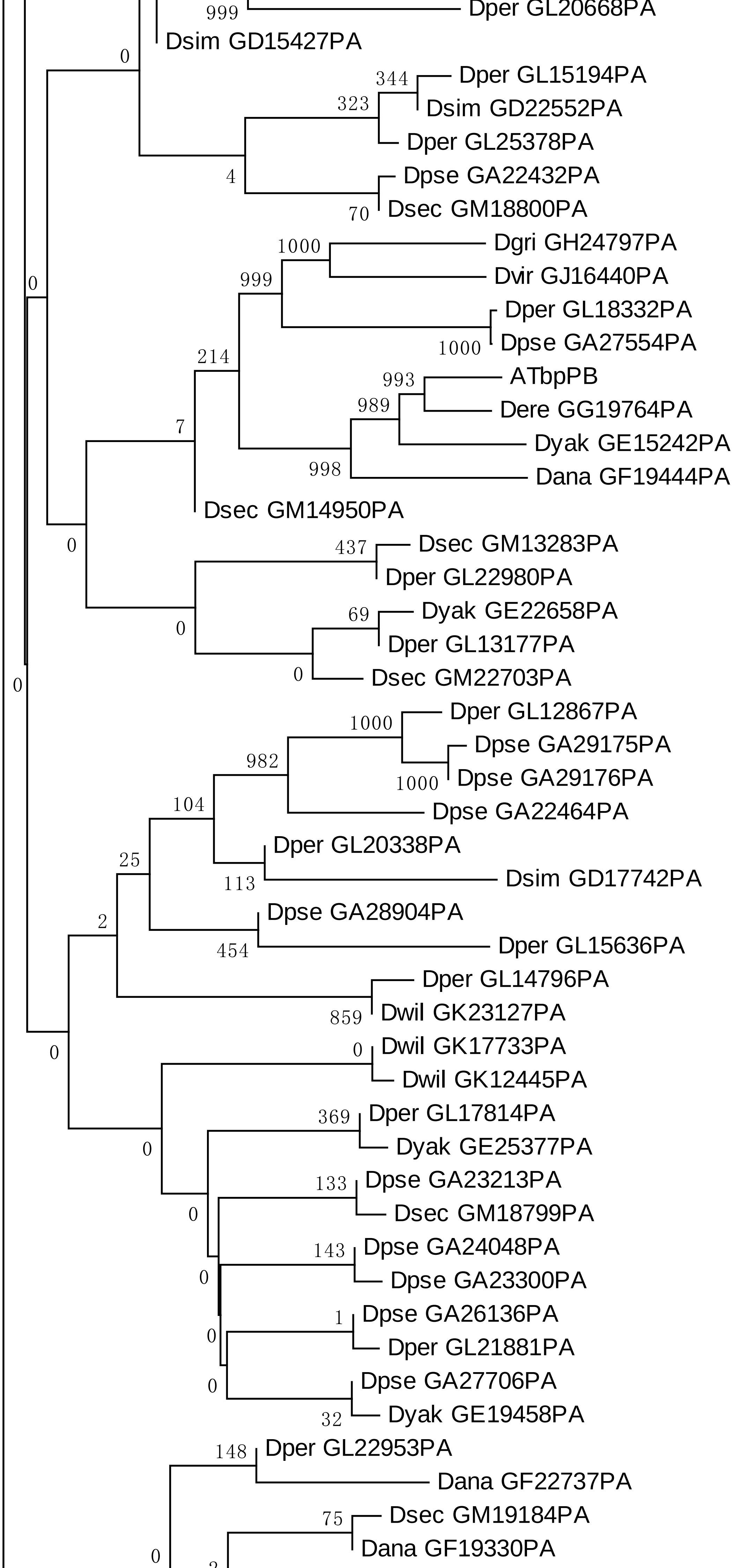

999
Dper GL20668PA
Dsim GD15427PA
0
344
Dper GL15194PA
323
Dsim GD22552PA
Dper GL25378PA
4
Dpse GA22432PA
Dsec GM18800PA
70
1000
Dgri GH24797PA
999
Dvir GJ16440PA
0
Dper GL18332PA
1000
Dpse GA27554PA
214
993
ATbpPB
989
Dere GG19764PA
7
Dyak GE15242PA
998
Dana GF19444PA
Dsec GM14950PA
0
437
Dsec GM13283PA
Dper GL22980PA
69
Dyak GE22658PA
0
Dper GL13177PA
0
Dsec GM22703PA
0
1000
Dper GL12867PA
Dpse GA29175PA
982
1000
Dpse GA29176PA
104
Dpse GA22464PA
Dper GL20338PA
25
113
Dsim GD17742PA
Dpse GA28904PA
2
454
Dper GL15636PA
Dper GL14796PA
859
Dwil GK23127PA
0
0
Dwil GK17733PA
Dwil GK12445PA
369
Dper GL17814PA
0
Dyak GE25377PA
133
Dpse GA23213PA
0
Dsec GM18799PA
143
Dpse GA24048PA
0
Dpse GA23300PA
1
Dpse GA26136PA
0
Dper GL21881PA
0
Dpse GA27706PA
32
Dyak GE19458PA
148
Dper GL22953PA
Dana GF22737PA
75
Dsec GM19184PA
0
Dana GF19330PA

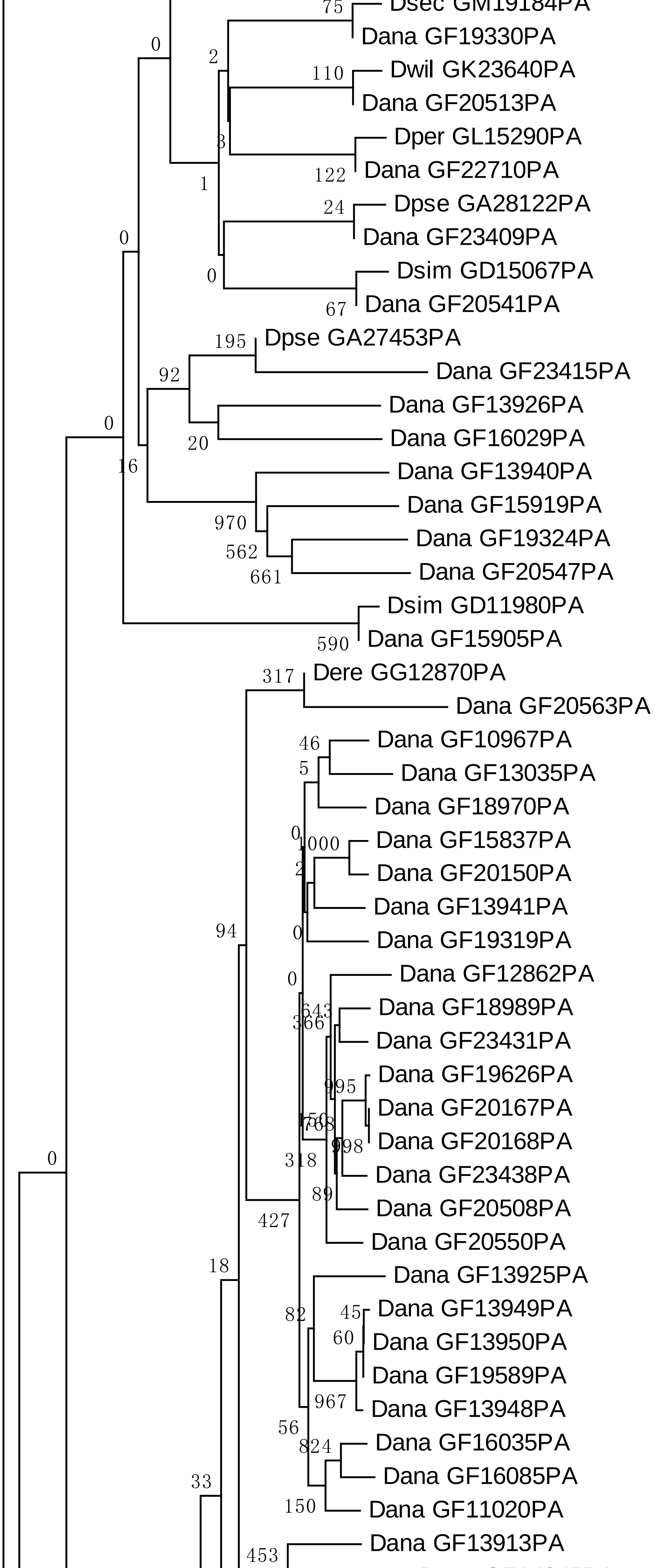

Dana GF19330PA
Dwil GK23640PA
Dana GF20513PA
Dper GL15290PA
Dana GF22710PA
Dpse GA28122PA
Dana GF23409PA
Dsim GD15067PA
Dana GF20541PA
Dpse GA27453PA
Dana GF23415PA
Dana GF13926PA
Dana GF16029PA
Dana GF13940PA
Dana GF15919PA
Dana GF19324PA
Dana GF20547PA
Dsim GD11980PA
Dana GF15905PA
Dere GG12870PA
Dana GF20563PA
Dana GF10967PA
Dana GF13035PA
Dana GF18970PA
Dana GF15837PA
Dana GF20150PA
Dana GF13941PA
Dana GF19319PA
Dana GF12862PA
Dana GF18989PA
Dana GF23431PA
Dana GF19626PA
Dana GF20167PA
Dana GF20168PA
Dana GF23438PA
Dana GF20508PA
Dana GF20550PA
Dana GF13925PA
Dana GF13949PA
Dana GF13950PA
Dana GF19589PA
Dana GF13948PA
Dana GF16035PA
Dana GF16085PA
Dana GF11020PA
Dana GF13913PA

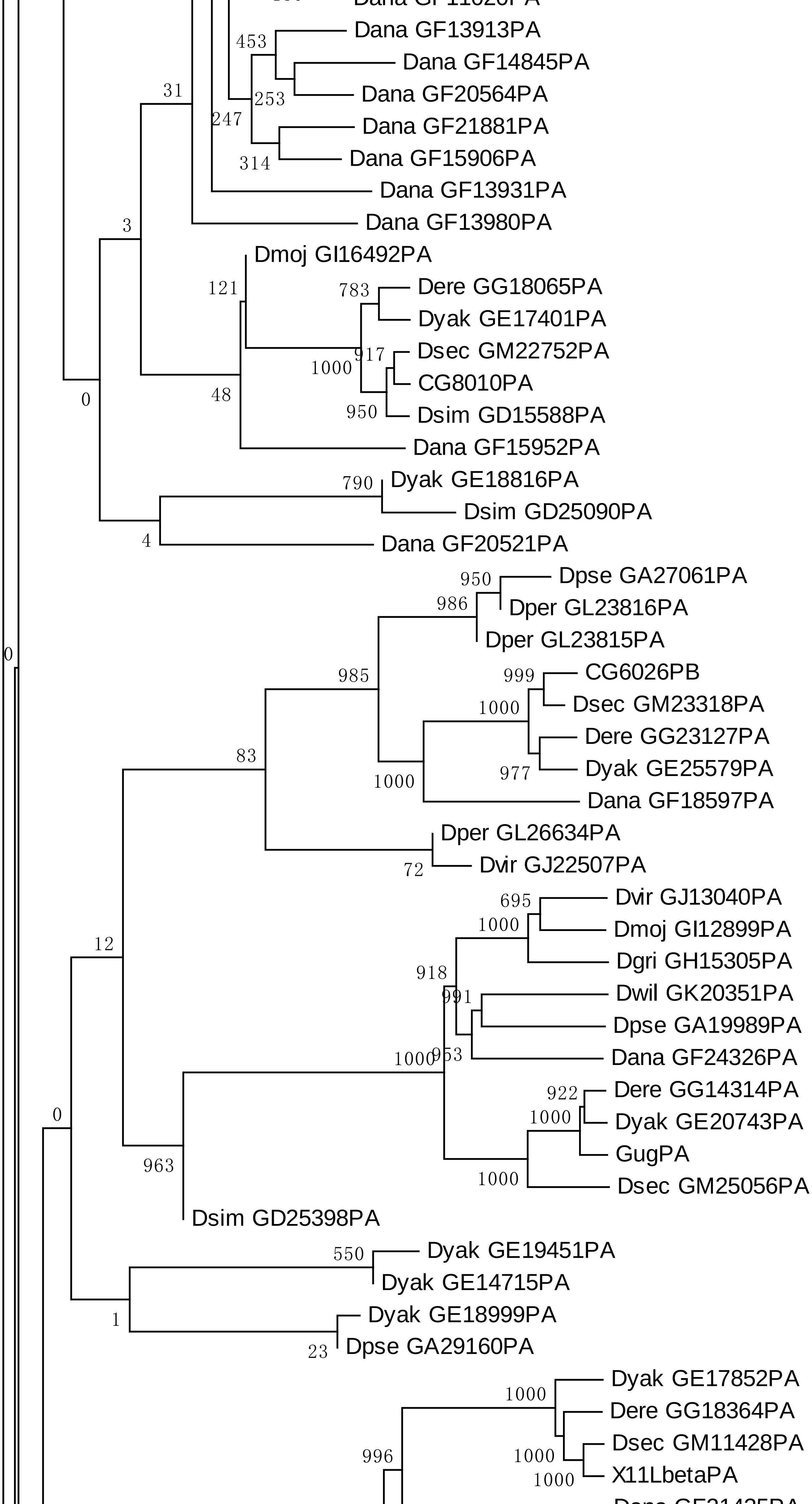

Dana GF13913PA
Dana GF14845PA
Dana GF20564PA
Dana GF21881PA
Dana GF15906PA
Dana GF13931PA
Dana GF13980PA
Dmoj GI16492PA
Dere GG18065PA
Dyak GE17401PA
Dsec GM22752PA
CG8010PA
Dsim GD15588PA
Dana GF15952PA
Dyak GE18816PA
Dsim GD25090PA
Dana GF20521PA
Dpse GA27061PA
Dper GL23816PA
Dper GL23815PA
CG6026PB
Dsec GM23318PA
Dere GG23127PA
Dyak GE25579PA
Dana GF18597PA
Dper GL26634PA
Dvir GJ22507PA
Dvir GJ13040PA
Dmoj GI12899PA
Dgri GH15305PA
Dwil GK20351PA
Dpse GA19989PA
Dana GF24326PA
Dere GG14314PA
Dyak GE20743PA
GugPA
Dsec GM25056PA
Dsim GD25398PA
Dyak GE19451PA
Dyak GE14715PA
Dyak GE18999PA
Dpse GA29160PA
Dyak GE17852PA
Dere GG18364PA
Dsec GM11428PA
X11LbetaPA
453
253
247
314
31
3
121
783
917
1000
950
48
0
790
4
950
986
985
999
1000
977
1000
83
72
695
1000
918
991
1000
953
922
1000
1000
12
963
0
550
1
23
1000
996
1000
1000
0

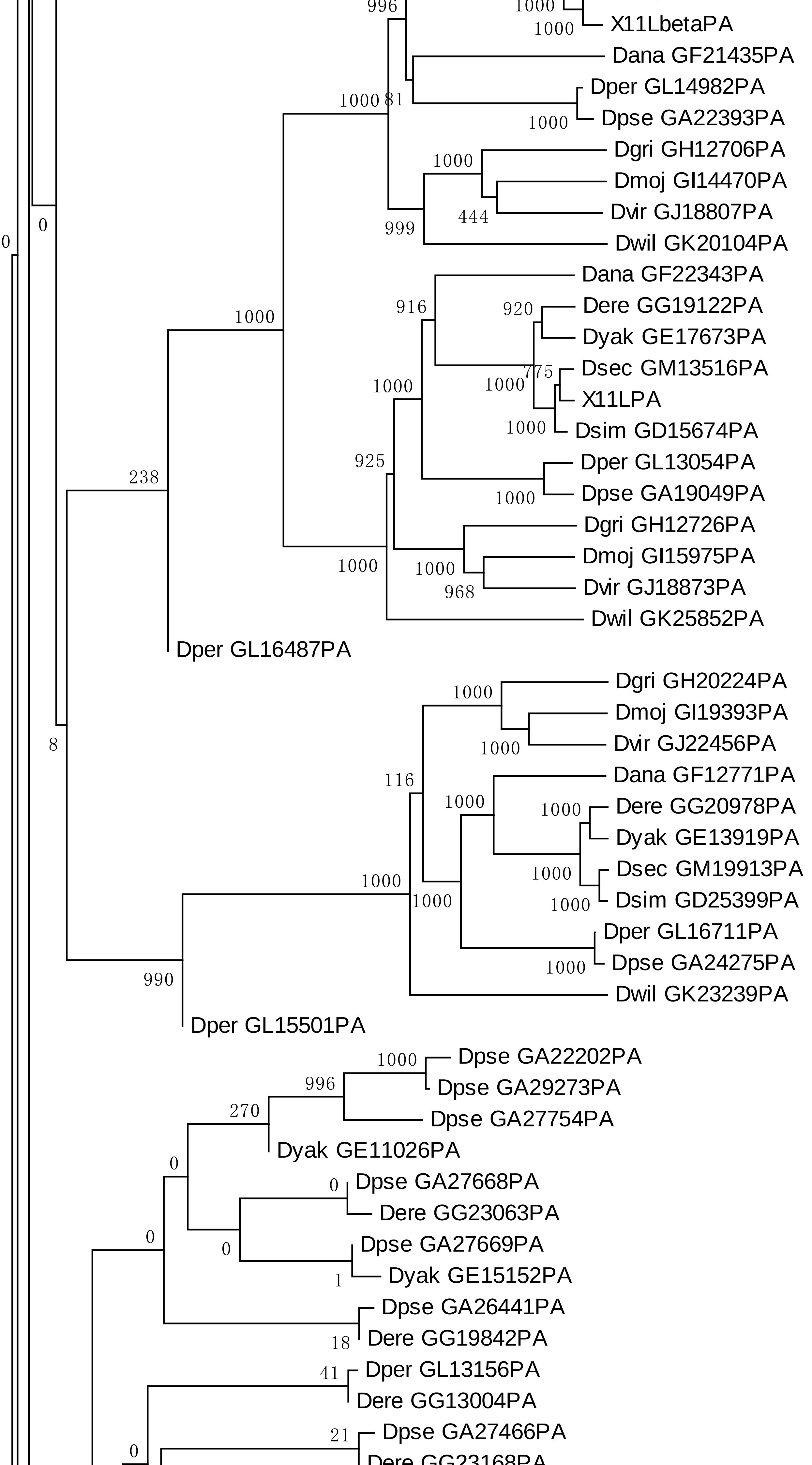
996
1000
1000
X11LbetaPA
Dana GF21435PA
1000
81
Dper GL14982PA
1000
Dpse GA22393PA
1000
Dgri GH12706PA
Dmoj GI14470PA
444
999
Dvir GJ18807PA
0
Dwil GK20104PA
0
Dana GF22343PA
1000
916
920
Dere GG19122PA
Dyak GE17673PA
775
Dsec GM13516PA
1000
1000
X11LPA
1000
Dsim GD15674PA
925
Dper GL13054PA
238
1000
Dpse GA19049PA
Dgri GH12726PA
1000
1000
Dmoj GI15975PA
968
Dvir GJ18873PA
Dwil GK25852PA
Dper GL16487PA
Dgri GH20224PA
1000
Dmoj GI19393PA
1000
Dvir GJ22456PA
8
116
Dana GF12771PA
1000
1000
Dere GG20978PA
Dyak GE13919PA
1000
Dsec GM19913PA
1000
1000
1000
Dsim GD25399PA
Dper GL16711PA
1000
Dpse GA24275PA
990
Dwil GK23239PA
Dper GL15501PA
1000
Dpse GA22202PA
996
Dpse GA29273PA
270
Dpse GA27754PA
Dyak GE11026PA
0
0
Dpse GA27668PA
Dere GG23063PA
0
0
Dpse GA27669PA
1
Dyak GE15152PA
Dpse GA26441PA
18
Dere GG19842PA
41
Dper GL13156PA
Dere GG13004PA
21
Dpse GA27466PA
0

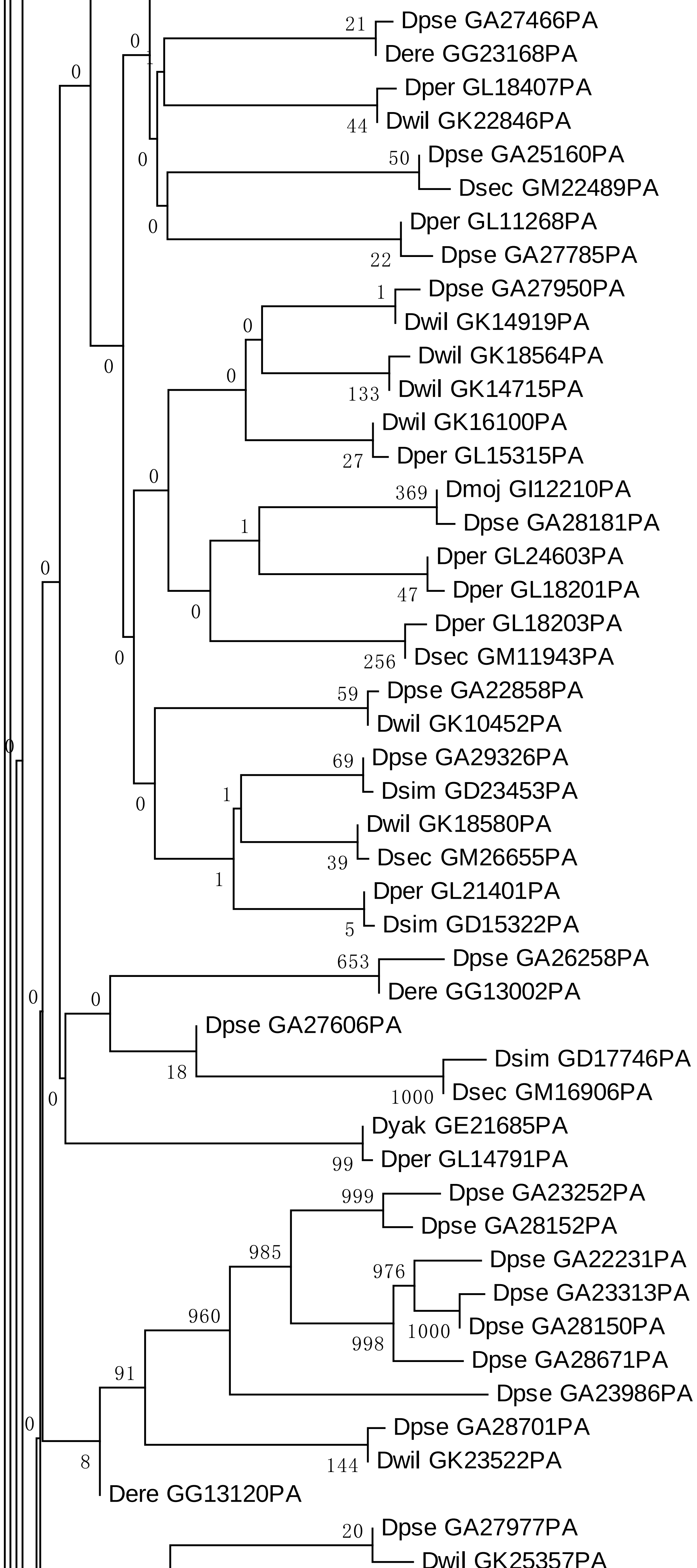

21
Dpse GA27466PA
0
Dere GG23168PA
0
Dper GL18407PA
44
Dwil GK22846PA
0
50
Dpse GA25160PA
Dsec GM22489PA
0
Dper GL11268PA
22
Dpse GA27785PA
1
Dpse GA27950PA
0
Dwil GK14919PA
0
Dwil GK18564PA
0
133
Dwil GK14715PA
Dwil GK16100PA
27
Dper GL15315PA
0
369
Dmoj GI12210PA
1
Dpse GA28181PA
0
Dper GL24603PA
47
Dper GL18201PA
0
Dper GL18203PA
0
256
Dsec GM11943PA
59
Dpse GA22858PA
Dwil GK10452PA
0
69
Dpse GA29326PA
1
Dsim GD23453PA
Dwil GK18580PA
39
Dsec GM26655PA
1
Dper GL21401PA
5
Dsim GD15322PA
653
Dpse GA26258PA
0
0
Dere GG13002PA
Dpse GA27606PA
Dsim GD17746PA
18
1000
Dsec GM16906PA
0
Dyak GE21685PA
99
Dper GL14791PA
999
Dpse GA23252PA
Dpse GA28152PA
985
Dpse GA22231PA
976
Dpse GA23313PA
960
1000
Dpse GA28150PA
998
Dpse GA28671PA
91
Dpse GA23986PA
Dpse GA28701PA
0
144
Dwil GK23522PA
8
Dere GG13120PA
20
Dpse GA27977PA
Dwil GK25357PA

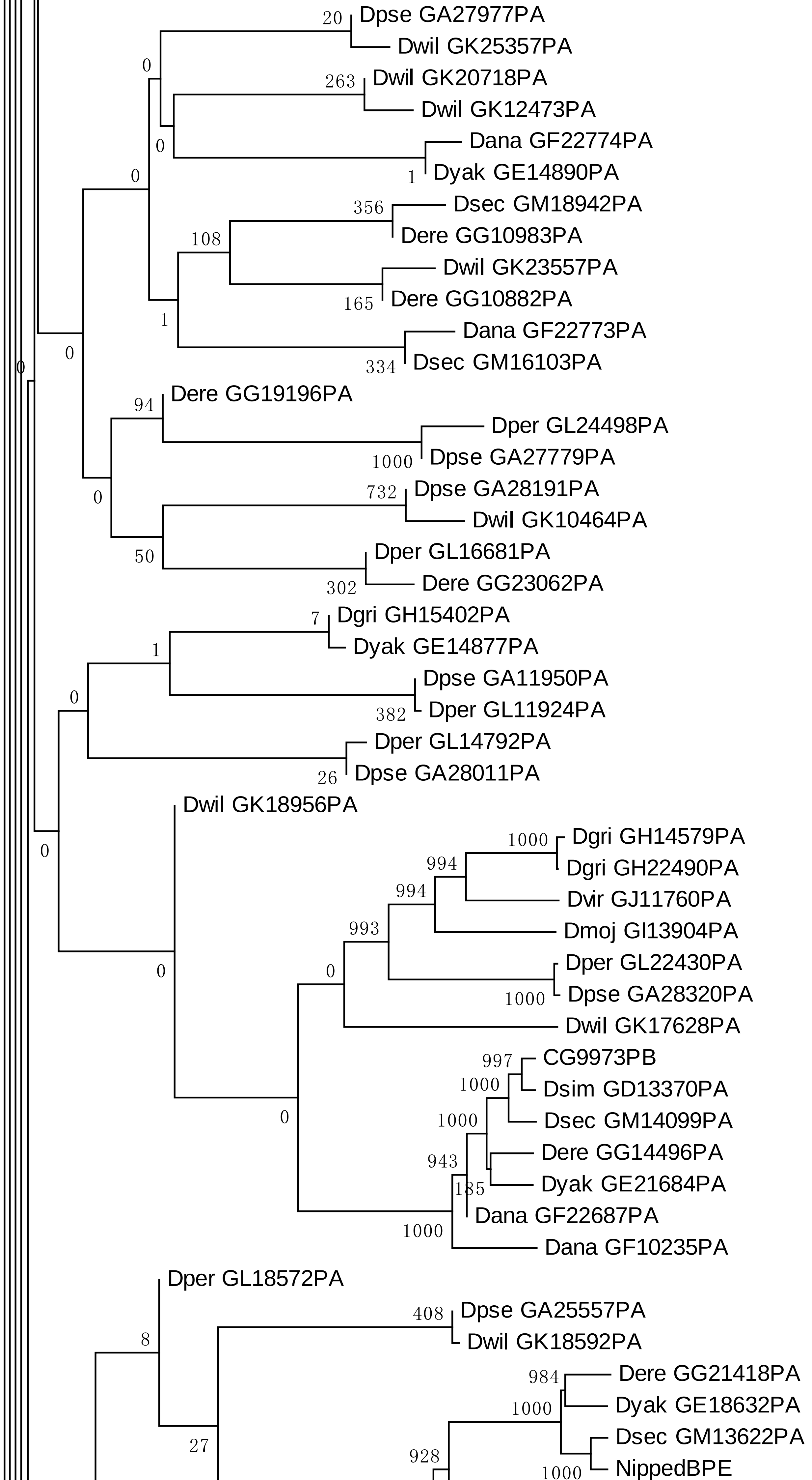

20
Dpse GA27977PA
Dwil GK25357PA
0
263
Dwil GK20718PA
Dwil GK12473PA
0
Dana GF22774PA
1
Dyak GE14890PA
0
356
Dsec GM18942PA
108
Dere GG10983PA
Dwil GK23557PA
165
Dere GG10882PA
1
Dana GF22773PA
0
334
Dsec GM16103PA
0
94
Dere GG19196PA
Dper GL24498PA
1000
Dpse GA27779PA
732
Dpse GA28191PA
0
Dwil GK10464PA
50
Dper GL16681PA
302
Dere GG23062PA
7
Dgri GH15402PA
1
Dyak GE14877PA
0
Dpse GA11950PA
382
Dper GL11924PA
Dper GL14792PA
26
Dpse GA28011PA
Dwil GK18956PA
0
1000
Dgri GH14579PA
994
Dgri GH22490PA
994
Dvir GJ11760PA
993
Dmoj GI13904PA
0
0
Dper GL22430PA
1000
Dpse GA28320PA
Dwil GK17628PA
997
CG9973PB
1000
Dsim GD13370PA
0
1000
Dsec GM14099PA
Dere GG14496PA
943
185
Dyak GE21684PA
Dana GF22687PA
1000
Dana GF10235PA
Dper GL18572PA
408
Dpse GA25557PA
8
Dwil GK18592PA
984
Dere GG21418PA
1000
Dyak GE18632PA
27
Dsec GM13622PA
928
1000
NippedBPE

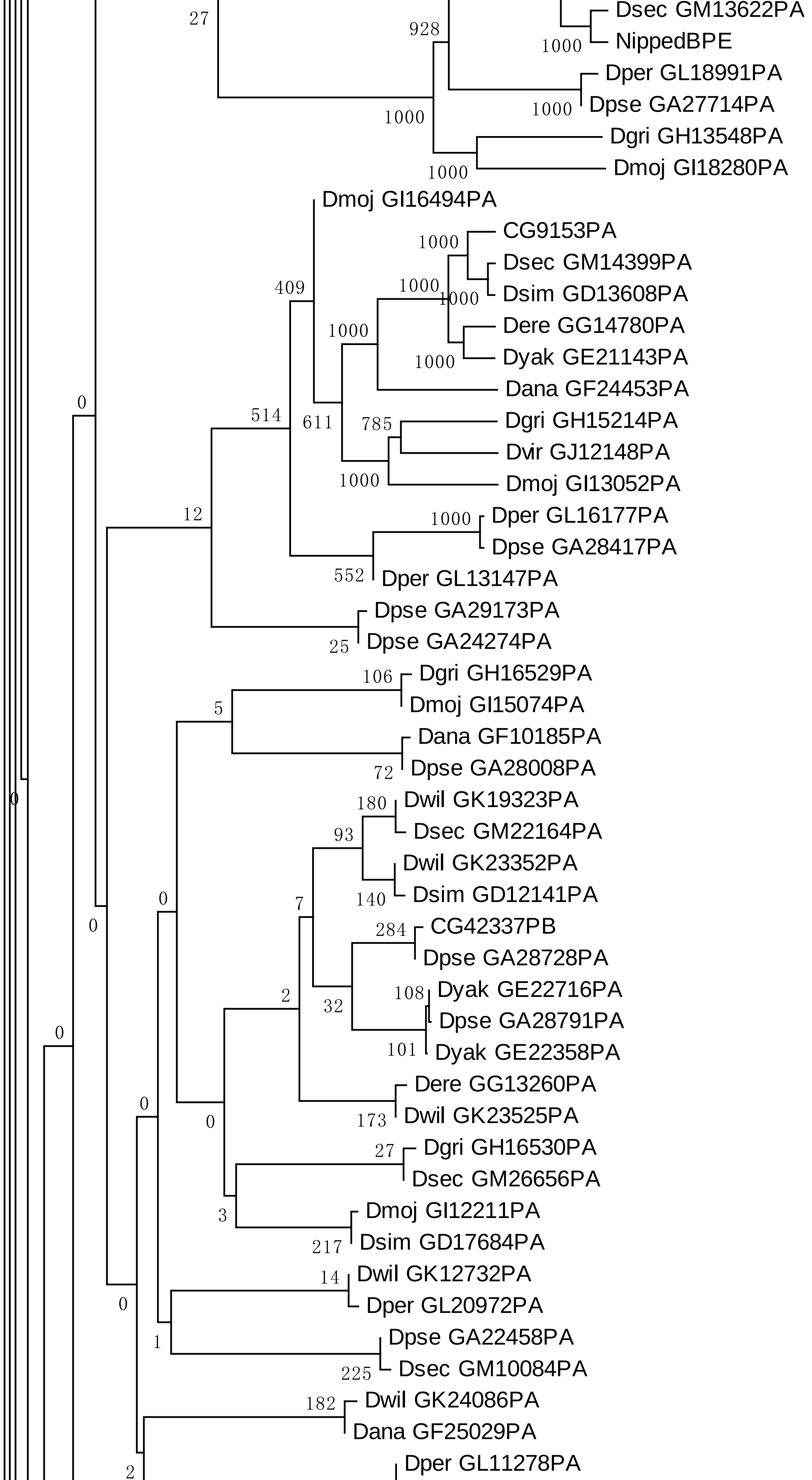

Dsec GM13622PA
NippedBPE
Dper GL18991PA
Dpse GA27714PA
Dgri GH13548PA
Dmoj GI18280PA
Dmoj GI16494PA
CG9153PA
Dsec GM14399PA
Dsim GD13608PA
Dere GG14780PA
Dyak GE21143PA
Dana GF24453PA
Dgri GH15214PA
Dvir GJ12148PA
Dmoj GI13052PA
Dper GL16177PA
Dpse GA28417PA
Dper GL13147PA
Dpse GA29173PA
Dpse GA24274PA
Dgri GH16529PA
Dmoj GI15074PA
Dana GF10185PA
Dpse GA28008PA
Dwil GK19323PA
Dsec GM22164PA
Dwil GK23352PA
Dsim GD12141PA
CG42337PB
Dpse GA28728PA
Dyak GE22716PA
Dpse GA28791PA
Dyak GE22358PA
Dere GG13260PA
Dwil GK23525PA
Dgri GH16530PA
Dsec GM26656PA
Dmoj GI12211PA
Dsim GD17684PA
Dwil GK12732PA
Dper GL20972PA
Dpse GA22458PA
Dsec GM10084PA
Dwil GK24086PA
Dana GF25029PA
Dper GL11278PA

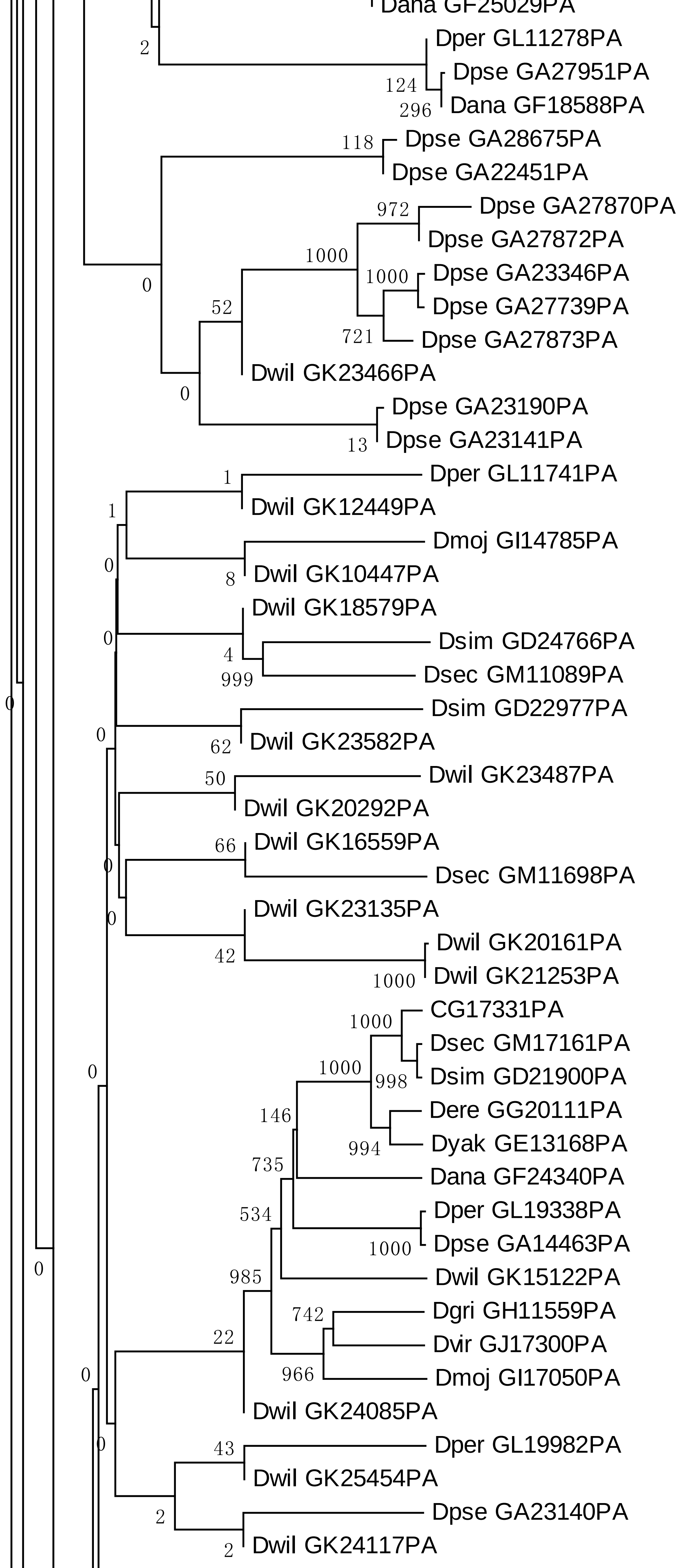

Dana GF25029PA
Dper GL11278PA
Dpse GA27951PA
Dana GF18588PA
Dpse GA28675PA
Dpse GA22451PA
Dpse GA27870PA
Dpse GA27872PA
Dpse GA23346PA
Dpse GA27739PA
Dpse GA27873PA
Dwil GK23466PA
Dpse GA23190PA
Dpse GA23141PA
Dper GL11741PA
Dwil GK12449PA
Dmoj GI14785PA
Dwil GK10447PA
Dwil GK18579PA
Dsim GD24766PA
Dsec GM11089PA
Dsim GD22977PA
Dwil GK23582PA
Dwil GK23487PA
Dwil GK20292PA
Dwil GK16559PA
Dsec GM11698PA
Dwil GK23135PA
Dwil GK20161PA
Dwil GK21253PA
CG17331PA
Dsec GM17161PA
Dsim GD21900PA
Dere GG20111PA
Dyak GE13168PA
Dana GF24340PA
Dper GL19338PA
Dpse GA14463PA
Dwil GK15122PA
Dgri GH11559PA
Dvir GJ17300PA
Dmoj GI17050PA
Dwil GK24085PA
Dper GL19982PA
Dwil GK25454PA
Dpse GA23140PA
Dwil GK24117PA

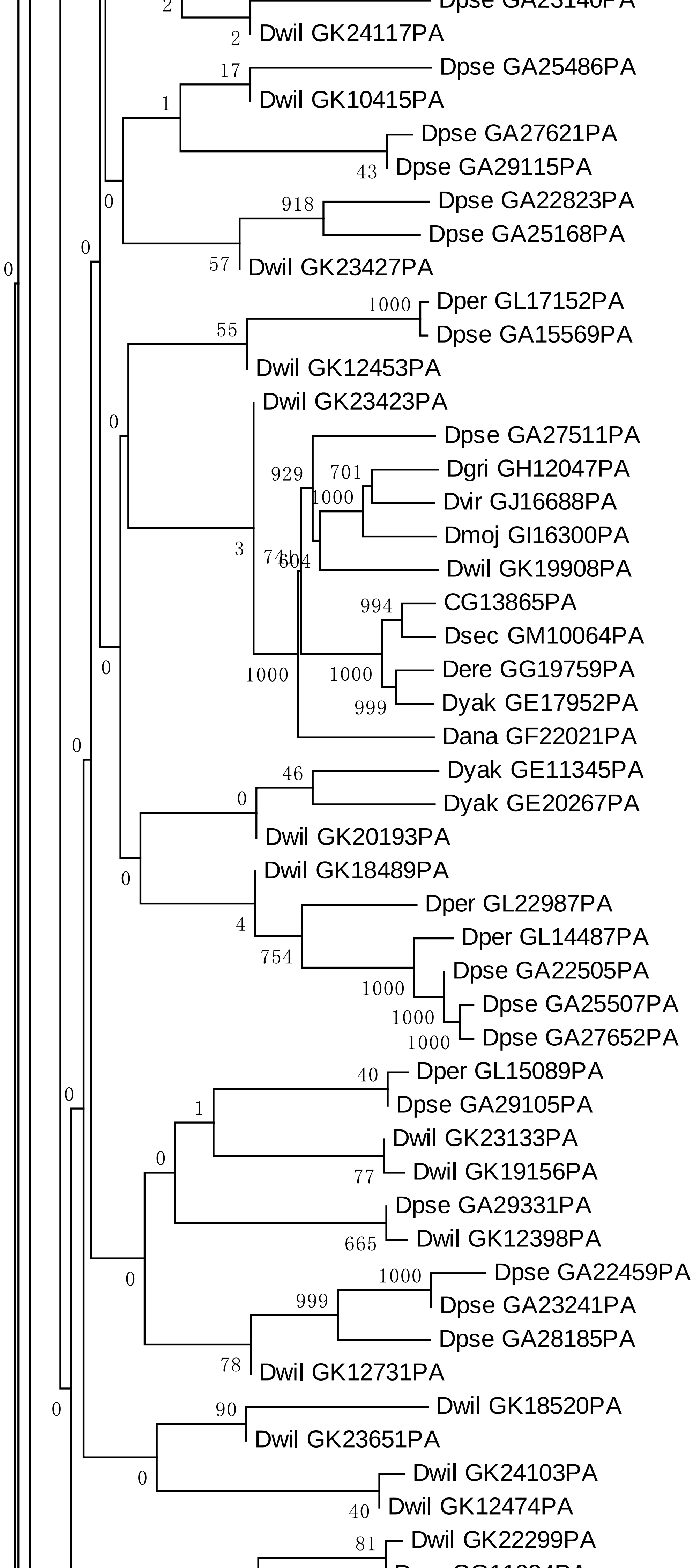

Dwil GK24117PA
Dpse GA25486PA
Dwil GK10415PA
Dpse GA27621PA
Dpse GA29115PA
Dpse GA22823PA
Dpse GA25168PA
Dwil GK23427PA
Dper GL17152PA
Dpse GA15569PA
Dwil GK12453PA
Dwil GK23423PA
Dpse GA27511PA
Dgri GH12047PA
Dvir GJ16688PA
Dmoj GI16300PA
Dwil GK19908PA
CG13865PA
Dsec GM10064PA
Dere GG19759PA
Dyak GE17952PA
Dana GF22021PA
Dyak GE11345PA
Dyak GE20267PA
Dwil GK20193PA
Dwil GK18489PA
Dper GL22987PA
Dper GL14487PA
Dpse GA22505PA
Dpse GA25507PA
Dpse GA27652PA
Dper GL15089PA
Dpse GA29105PA
Dwil GK23133PA
Dwil GK19156PA
Dpse GA29331PA
Dwil GK12398PA
Dpse GA22459PA
Dpse GA23241PA
Dpse GA28185PA
Dwil GK12731PA
Dwil GK18520PA
Dwil GK23651PA
Dwil GK24103PA
Dwil GK12474PA
Dwil GK22299PA

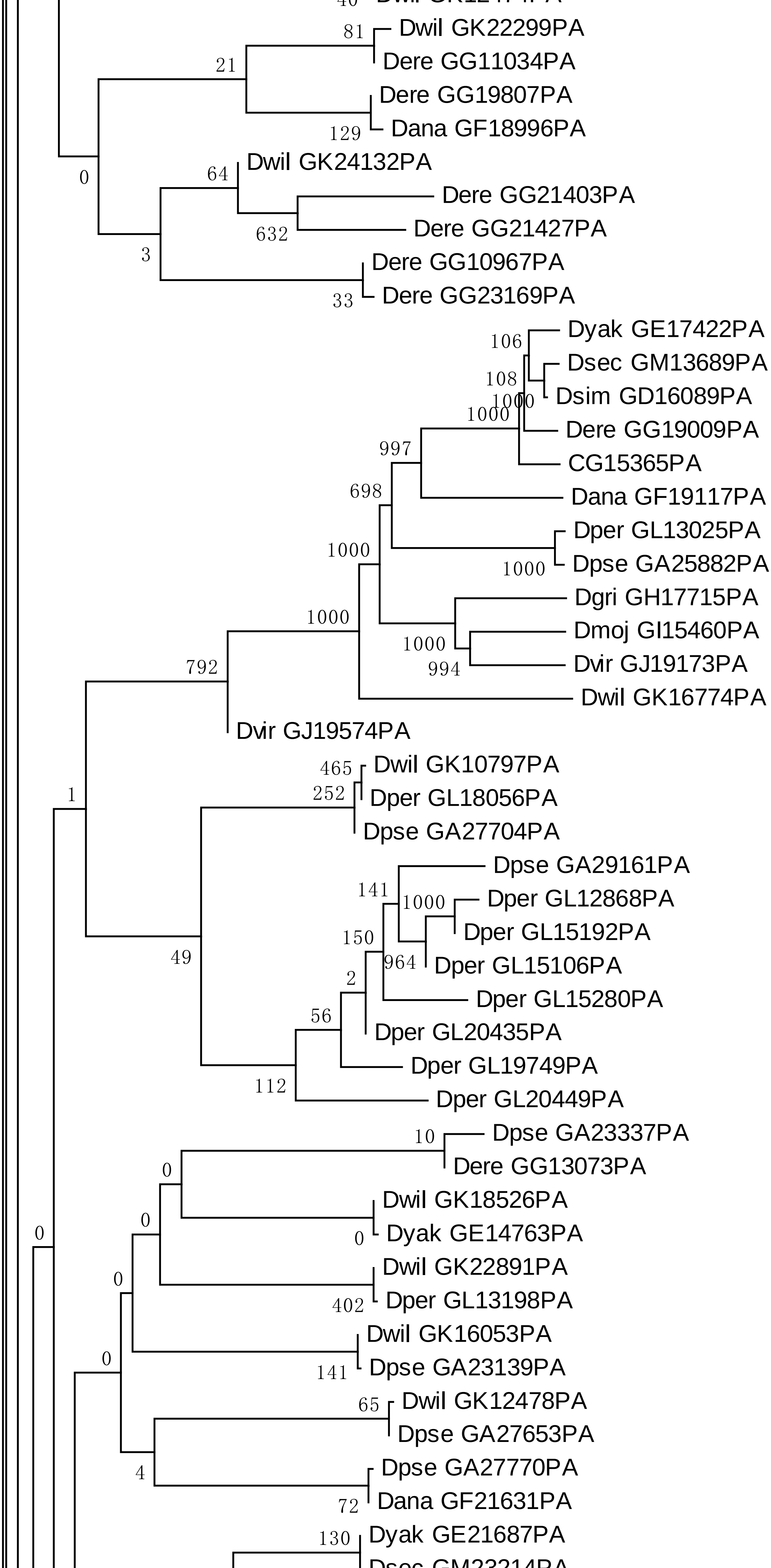
Dwil GK22299PA
Dere GG11034PA
Dere GG19807PA
Dana GF18996PA
Dwil GK24132PA
Dere GG21403PA
Dere GG21427PA
Dere GG10967PA
Dere GG23169PA
Dyak GE17422PA
Dsec GM13689PA
Dsim GD16089PA
Dere GG19009PA
CG15365PA
Dana GF19117PA
Dper GL13025PA
Dpse GA25882PA
Dgri GH17715PA
Dmoj GI15460PA
Dvir GJ19173PA
Dwil GK16774PA
Dvir GJ19574PA
Dwil GK10797PA
Dper GL18056PA
Dpse GA27704PA
Dpse GA29161PA
Dper GL12868PA
Dper GL15192PA
Dper GL15106PA
Dper GL15280PA
Dper GL20435PA
Dper GL19749PA
Dper GL20449PA
Dpse GA23337PA
Dere GG13073PA
Dwil GK18526PA
Dyak GE14763PA
Dwil GK22891PA
Dper GL13198PA
Dwil GK16053PA
Dpse GA23139PA
Dwil GK12478PA
Dpse GA27653PA
Dpse GA27770PA
Dana GF21631PA
Dyak GE21687PA

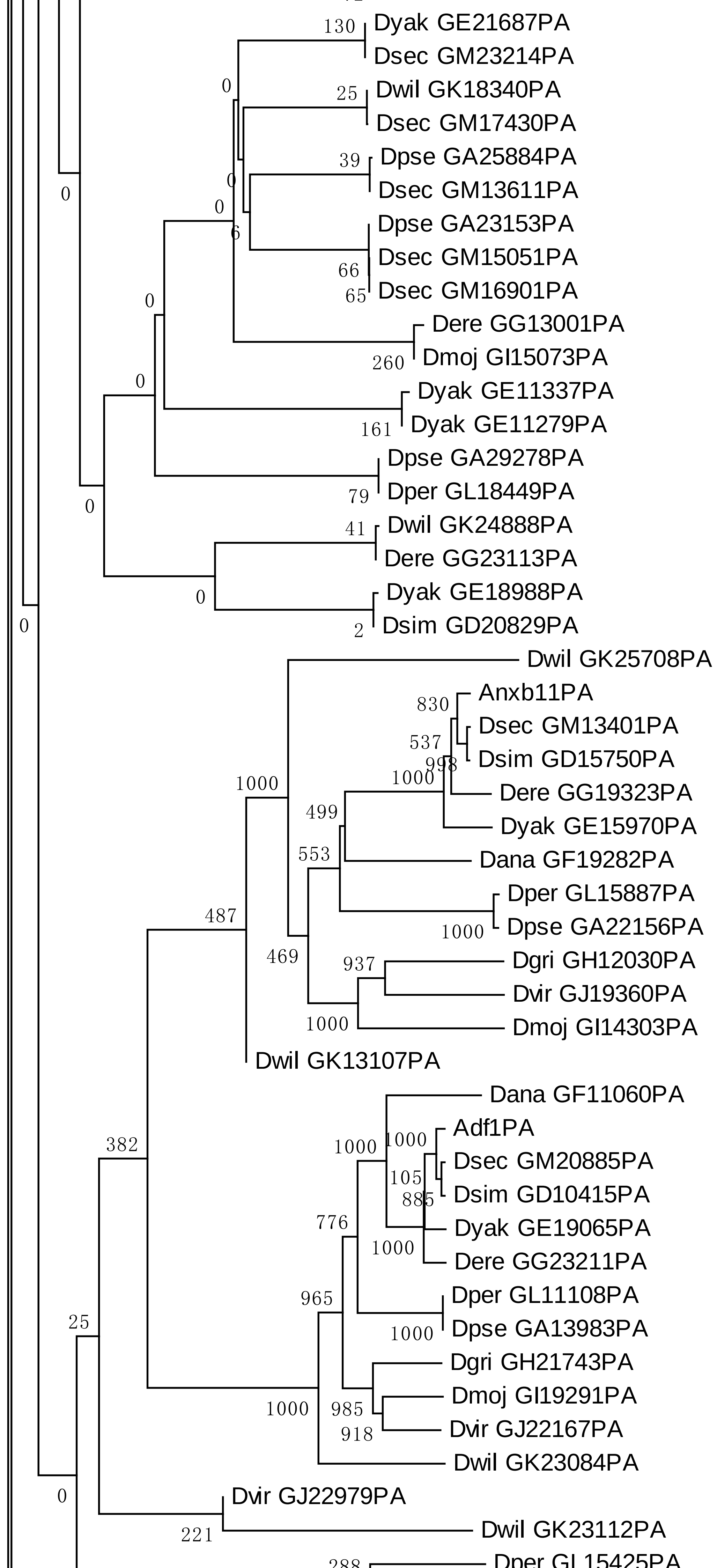
Dyak GE21687PA
Dsec GM23214PA
Dwil GK18340PA
Dsec GM17430PA
Dpse GA25884PA
Dsec GM13611PA
Dpse GA23153PA
Dsec GM15051PA
Dsec GM16901PA
Dere GG13001PA
Dmoj GI15073PA
Dyak GE11337PA
Dyak GE11279PA
Dpse GA29278PA
Dper GL18449PA
Dwil GK24888PA
Dere GG23113PA
Dyak GE18988PA
Dsim GD20829PA
Dwil GK25708PA
Anxb11PA
Dsec GM13401PA
Dsim GD15750PA
Dere GG19323PA
Dyak GE15970PA
Dana GF19282PA
Dper GL15887PA
Dpse GA22156PA
Dgri GH12030PA
Dvir GJ19360PA
Dmoj GI14303PA
Dwil GK13107PA
Dana GF11060PA
Adf1PA
Dsec GM20885PA
Dsim GD10415PA
Dyak GE19065PA
Dere GG23211PA
Dper GL11108PA
Dpse GA13983PA
Dgri GH21743PA
Dmoj GI19291PA
Dvir GJ22167PA
Dwil GK23084PA
Dvir GJ22979PA
Dwil GK23112PA

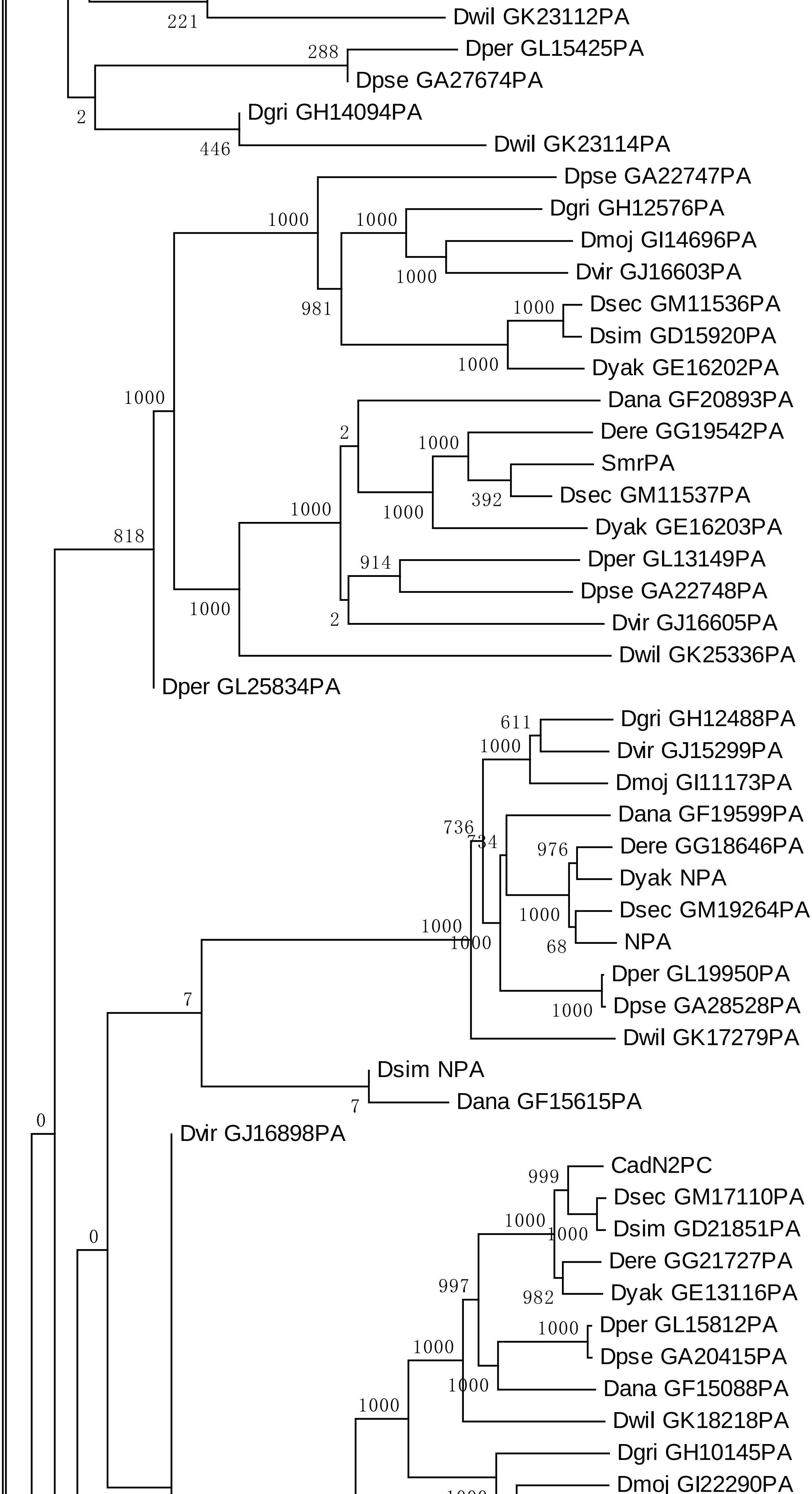
221
Dwil GK23112PA
288
Dper GL15425PA
Dpse GA27674PA
2
Dgri GH14094PA
446
Dwil GK23114PA
Dpse GA22747PA
1000
1000
Dgri GH12576PA
Dmoj GI14696PA
1000
Dvir GJ16603PA
981
1000
Dsec GM11536PA
Dsim GD15920PA
1000
Dyak GE16202PA
1000
Dana GF20893PA
2
Dere GG19542PA
1000
SmrPA
392
Dsec GM11537PA
1000
1000
Dyak GE16203PA
818
914
Dper GL13149PA
Dpse GA22748PA
1000
2
Dvir GJ16605PA
Dwil GK25336PA
Dper GL25834PA
611
Dgri GH12488PA
1000
Dvir GJ15299PA
Dmoj GI11173PA
Dana GF19599PA
736
734
976
Dere GG18646PA
Dyak NPA
1000
Dsec GM19264PA
1000
1000
68
NPA
Dper GL19950PA
1000
Dpse GA28528PA
7
Dwil GK17279PA
Dsim NPA
7
Dana GF15615PA
0
Dvir GJ16898PA
CadN2PC
999
Dsec GM17110PA
1000
1000
Dsim GD21851PA
0
Dere GG21727PA
997
982
Dyak GE13116PA
1000
Dper GL15812PA
1000
Dpse GA20415PA
1000
Dana GF15088PA
1000
Dwil GK18218PA
Dgri GH10145PA
Dmoj GI22290PA

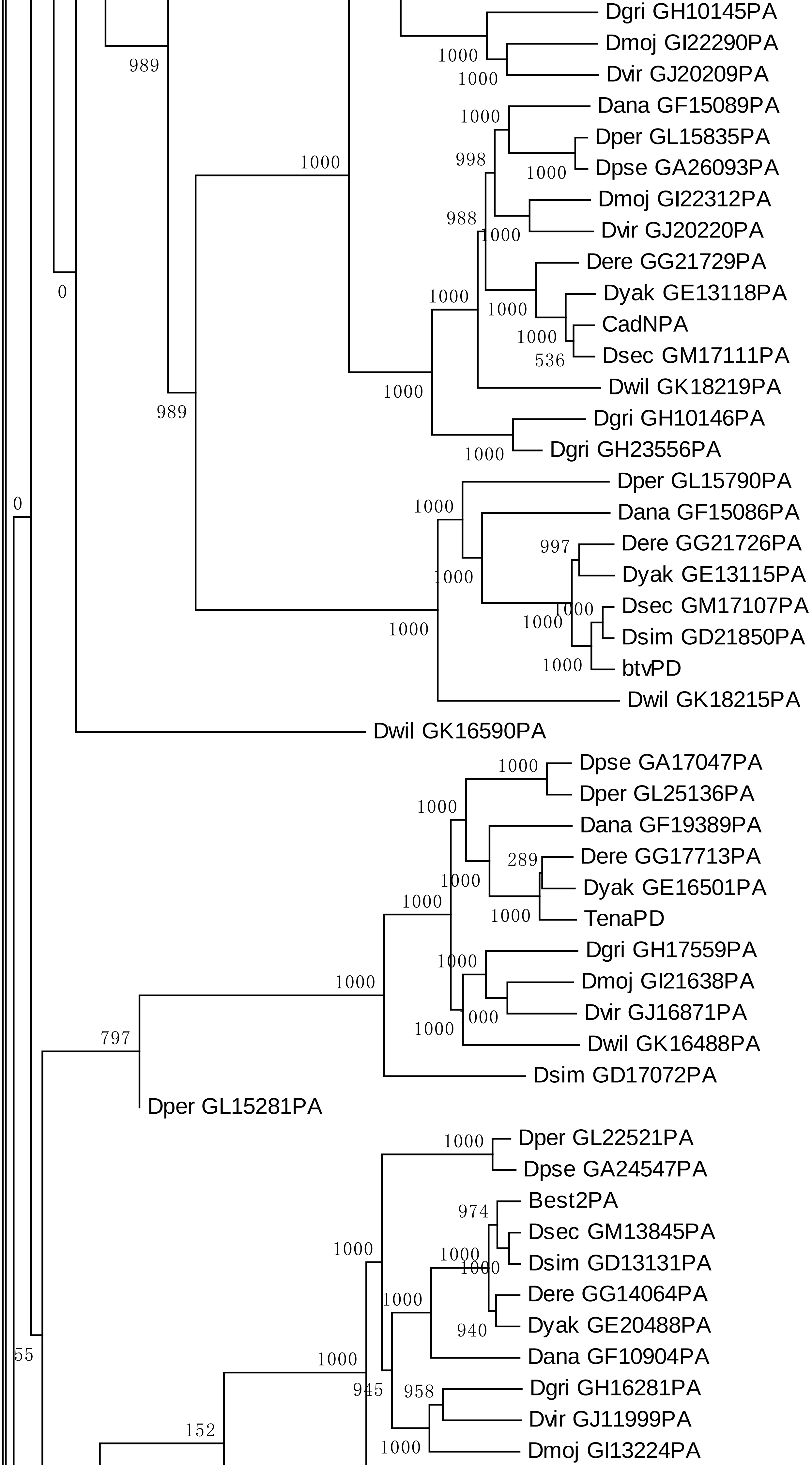
Dgri GH10145PA
Dmoj GI22290PA
Dvir GJ20209PA
Dana GF15089PA
Dper GL15835PA
Dpse GA26093PA
Dmoj GI22312PA
Dvir GJ20220PA
Dere GG21729PA
Dyak GE13118PA
CadNPA
Dsec GM17111PA
Dwil GK18219PA
Dgri GH10146PA
Dgri GH23556PA
Dper GL15790PA
Dana GF15086PA
Dere GG21726PA
Dyak GE13115PA
Dsec GM17107PA
Dsim GD21850PA
btvPD
Dwil GK18215PA
Dwil GK16590PA
Dpse GA17047PA
Dper GL25136PA
Dana GF19389PA
Dere GG17713PA
Dyak GE16501PA
TenaPD
Dgri GH17559PA
Dmoj GI21638PA
Dvir GJ16871PA
Dwil GK16488PA
Dsim GD17072PA
Dper GL15281PA
Dper GL22521PA
Dpse GA24547PA
Best2PA
Dsec GM13845PA
Dsim GD13131PA
Dere GG14064PA
Dyak GE20488PA
Dana GF10904PA
Dgri GH16281PA
Dvir GJ11999PA
Dmoj GI13224PA
989
1000
1000
1000
1000
998
1000
988
1000
0
1000
1000
1000
536
1000
989
1000
1000
1000
997
1000
1000
1000
1000
1000
0
1000
1000
289
1000
1000
1000
1000
1000
1000
1000
797
1000
1000
974
1000
1000
1000
940
55
1000
945
958
152
1000

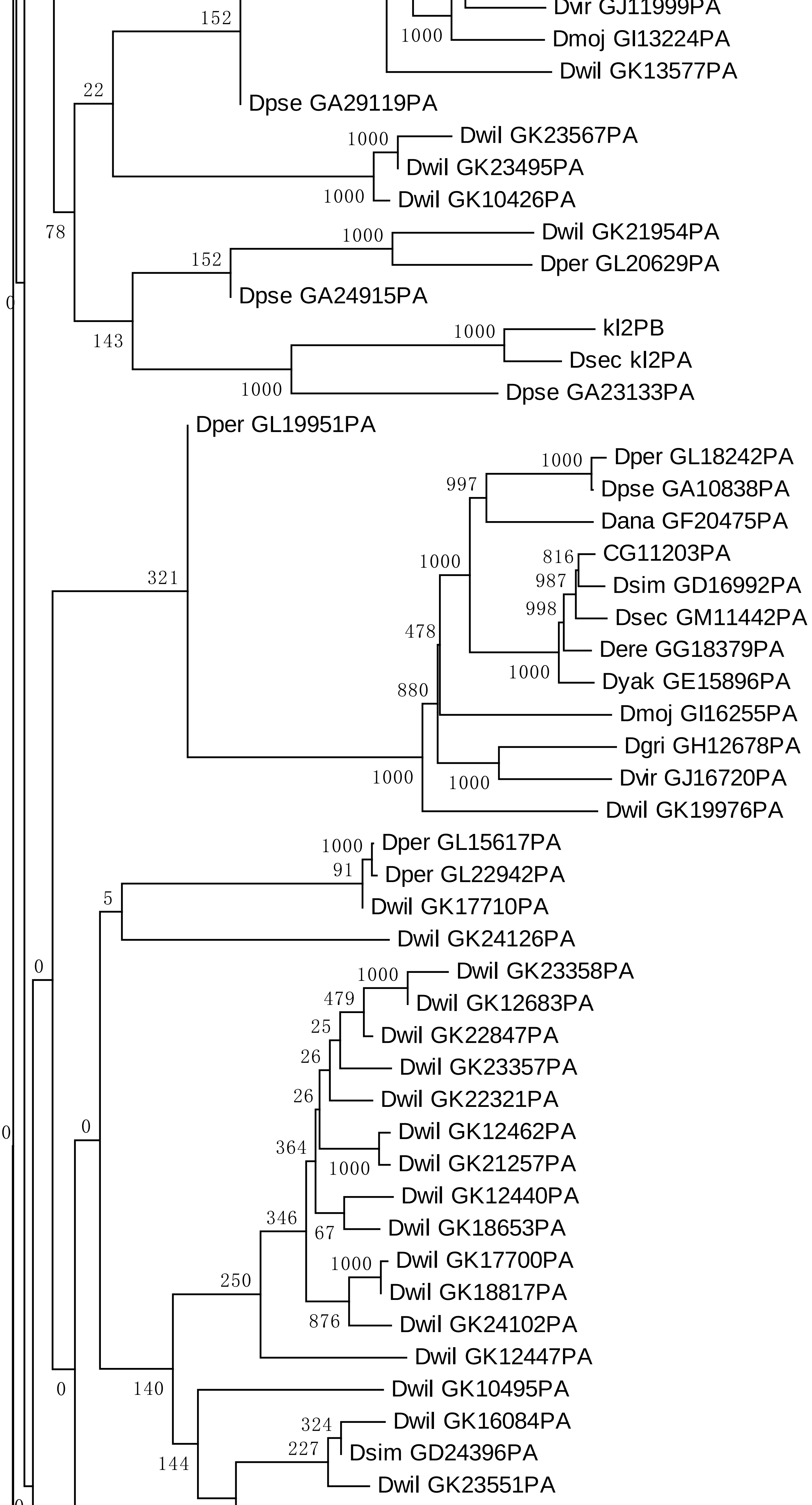

Dvir GJ11999PA
152
1000
Dmoj GI13224PA
Dwil GK13577PA
22
Dpse GA29119PA
1000
Dwil GK23567PA
Dwil GK23495PA
1000
Dwil GK10426PA
78
1000
Dwil GK21954PA
152
Dper GL20629PA
0
Dpse GA24915PA
1000
kl2PB
Dsec kl2PA
143
1000
Dpse GA23133PA
Dper GL19951PA
1000
Dper GL18242PA
997
Dpse GA10838PA
Dana GF20475PA
816
CG11203PA
1000
987
Dsim GD16992PA
998
Dsec GM11442PA
321
478
Dere GG18379PA
1000
Dyak GE15896PA
880
Dmoj GI16255PA
Dgri GH12678PA
1000
1000
Dvir GJ16720PA
Dwil GK19976PA
1000
Dper GL15617PA
91
Dper GL22942PA
5
Dwil GK17710PA
Dwil GK24126PA
0
1000
Dwil GK23358PA
479
Dwil GK12683PA
25
Dwil GK22847PA
26
Dwil GK23357PA
26
Dwil GK22321PA
0
0
Dwil GK12462PA
364
1000
Dwil GK21257PA
Dwil GK12440PA
346
67
Dwil GK18653PA
1000
Dwil GK17700PA
250
Dwil GK18817PA
876
Dwil GK24102PA
Dwil GK12447PA
0
140
Dwil GK10495PA
324
Dwil GK16084PA
227
Dsim GD24396PA
144
Dwil GK23551PA
0

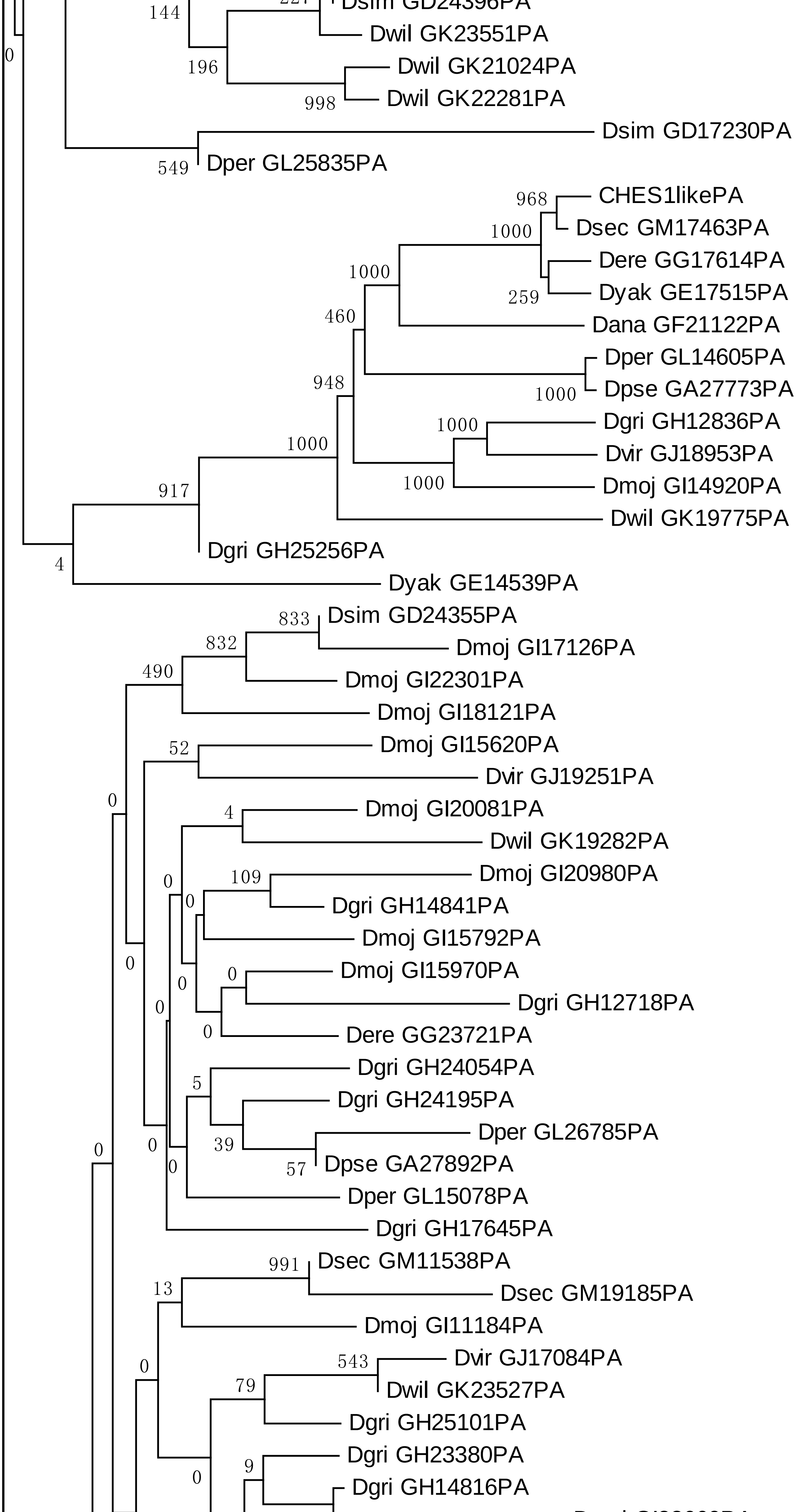
144
0
Dwil GK23551PA
196
Dwil GK21024PA
998
Dwil GK22281PA
Dsim GD17230PA
549
Dper GL25835PA
968
CHES1likePA
1000
Dsec GM17463PA
1000
Dere GG17614PA
259
Dyak GE17515PA
460
Dana GF21122PA
948
Dper GL14605PA
1000
Dpse GA27773PA
1000
Dgri GH12836PA
1000
Dvir GJ18953PA
917
1000
Dmoj GI14920PA
Dwil GK19775PA
Dgri GH25256PA
4
Dyak GE14539PA
833
Dsim GD24355PA
832
Dmoj GI17126PA
490
Dmoj GI22301PA
Dmoj GI18121PA
52
Dmoj GI15620PA
Dvir GJ19251PA
0
4
Dmoj GI20081PA
Dwil GK19282PA
0
109
Dmoj GI20980PA
0
Dgri GH14841PA
Dmoj GI15792PA
0
0
Dmoj GI15970PA
0
Dgri GH12718PA
0
Dere GG23721PA
5
Dgri GH24054PA
Dgri GH24195PA
0
39
Dper GL26785PA
0
0
57
Dpse GA27892PA
Dper GL15078PA
Dgri GH17645PA
991
Dsec GM11538PA
13
Dsec GM19185PA
Dmoj GI11184PA
0
543
Dvir GJ17084PA
79
Dwil GK23527PA
Dgri GH25101PA
Dgri GH23380PA
9
0
Dgri GH14816PA

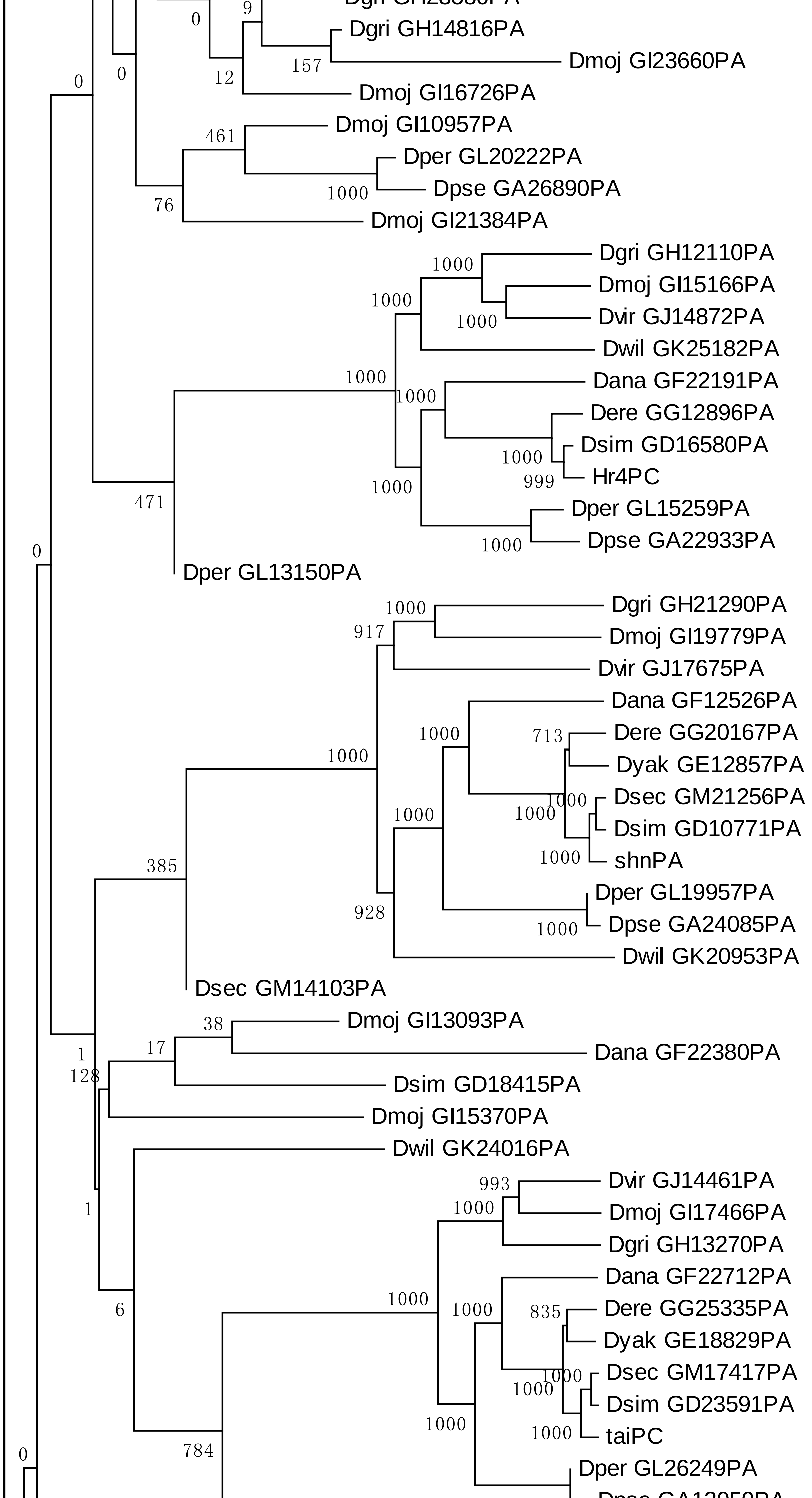

Dgri GH14816PA
Dmoj GI23660PA
Dmoj GI16726PA
Dmoj GI10957PA
Dper GL20222PA
Dpse GA26890PA
Dmoj GI21384PA
Dgri GH12110PA
Dmoj GI15166PA
Dvir GJ14872PA
Dwil GK25182PA
Dana GF22191PA
Dere GG12896PA
Dsim GD16580PA
Hr4PC
Dper GL15259PA
Dpse GA22933PA
Dper GL13150PA
Dgri GH21290PA
Dmoj GI19779PA
Dvir GJ17675PA
Dana GF12526PA
Dere GG20167PA
Dyak GE12857PA
Dsec GM21256PA
Dsim GD10771PA
shnPA
Dper GL19957PA
Dpse GA24085PA
Dwil GK20953PA
Dsec GM14103PA
Dmoj GI13093PA
Dana GF22380PA
Dsim GD18415PA
Dmoj GI15370PA
Dwil GK24016PA
Dvir GJ14461PA
Dmoj GI17466PA
Dgri GH13270PA
Dana GF22712PA
Dere GG25335PA
Dyak GE18829PA
Dsec GM17417PA
Dsim GD23591PA
taiPC
Dper GL26249PA

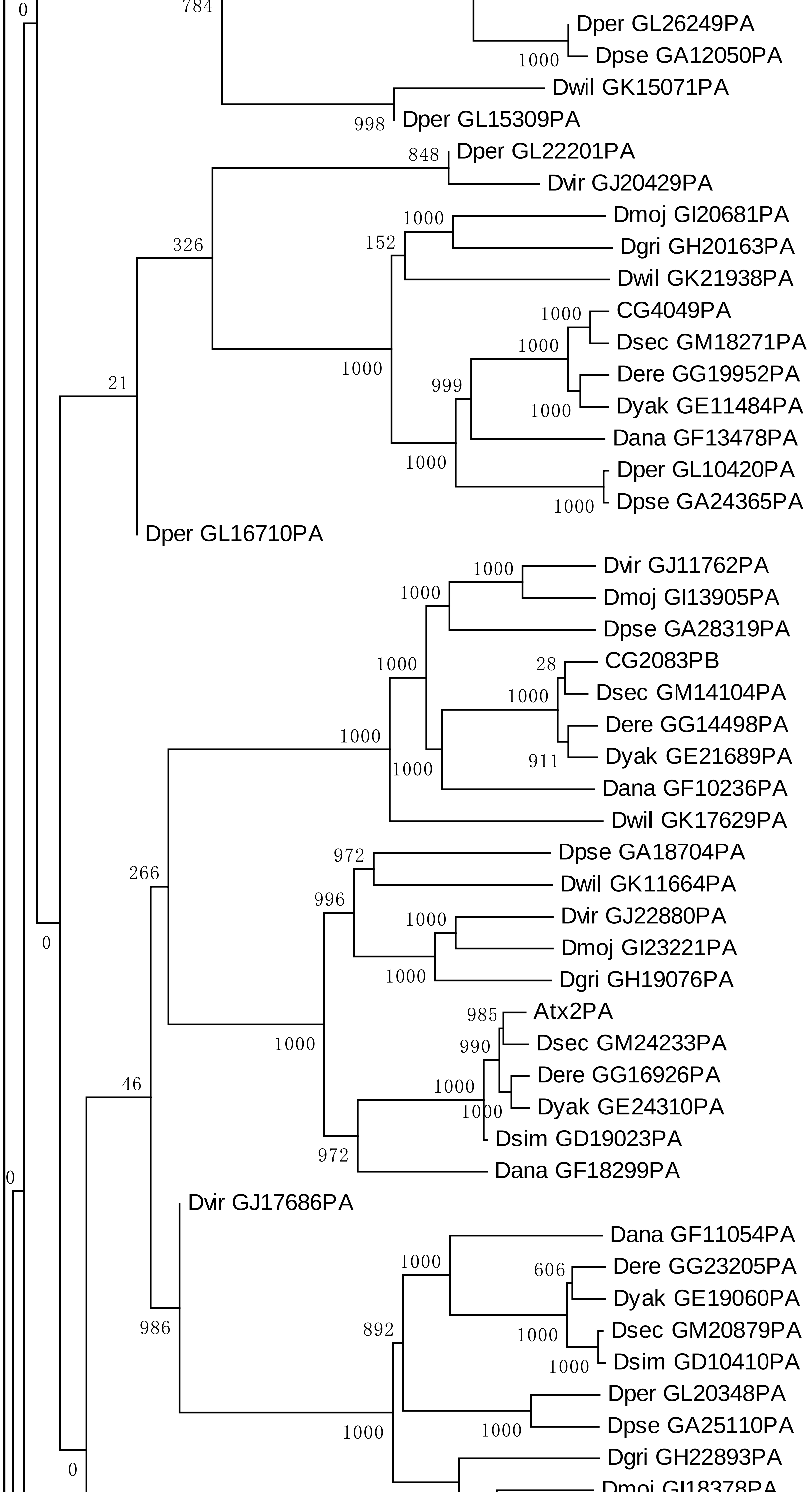
0
784
Dper GL26249PA
1000
Dpse GA12050PA
Dwil GK15071PA
998
Dper GL15309PA
848
Dper GL22201PA
Dvir GJ20429PA
1000
Dmoj GI20681PA
152
Dgri GH20163PA
326
Dwil GK21938PA
1000
CG4049PA
1000
Dsec GM18271PA
1000
Dere GG19952PA
21
999
1000
Dyak GE11484PA
Dana GF13478PA
1000
Dper GL10420PA
1000
Dpse GA24365PA
Dper GL16710PA
1000
Dvir GJ11762PA
1000
Dmoj GI13905PA
Dpse GA28319PA
1000
28
CG2083PB
1000
Dsec GM14104PA
1000
Dere GG14498PA
911
Dyak GE21689PA
1000
Dana GF10236PA
Dwil GK17629PA
972
Dpse GA18704PA
266
Dwil GK11664PA
996
1000
Dvir GJ22880PA
0
Dmoj GI23221PA
1000
Dgri GH19076PA
985
Atx2PA
1000
990
Dsec GM24233PA
1000
Dere GG16926PA
46
1000
Dyak GE24310PA
Dsim GD19023PA
972
Dana GF18299PA
0
Dvir GJ17686PA
Dana GF11054PA
1000
606
Dere GG23205PA
Dyak GE19060PA
986
892
1000
Dsec GM20879PA
1000
Dsim GD10410PA
Dper GL20348PA
1000
1000
Dpse GA25110PA
Dgri GH22893PA
0
Dmoj GI18378PA

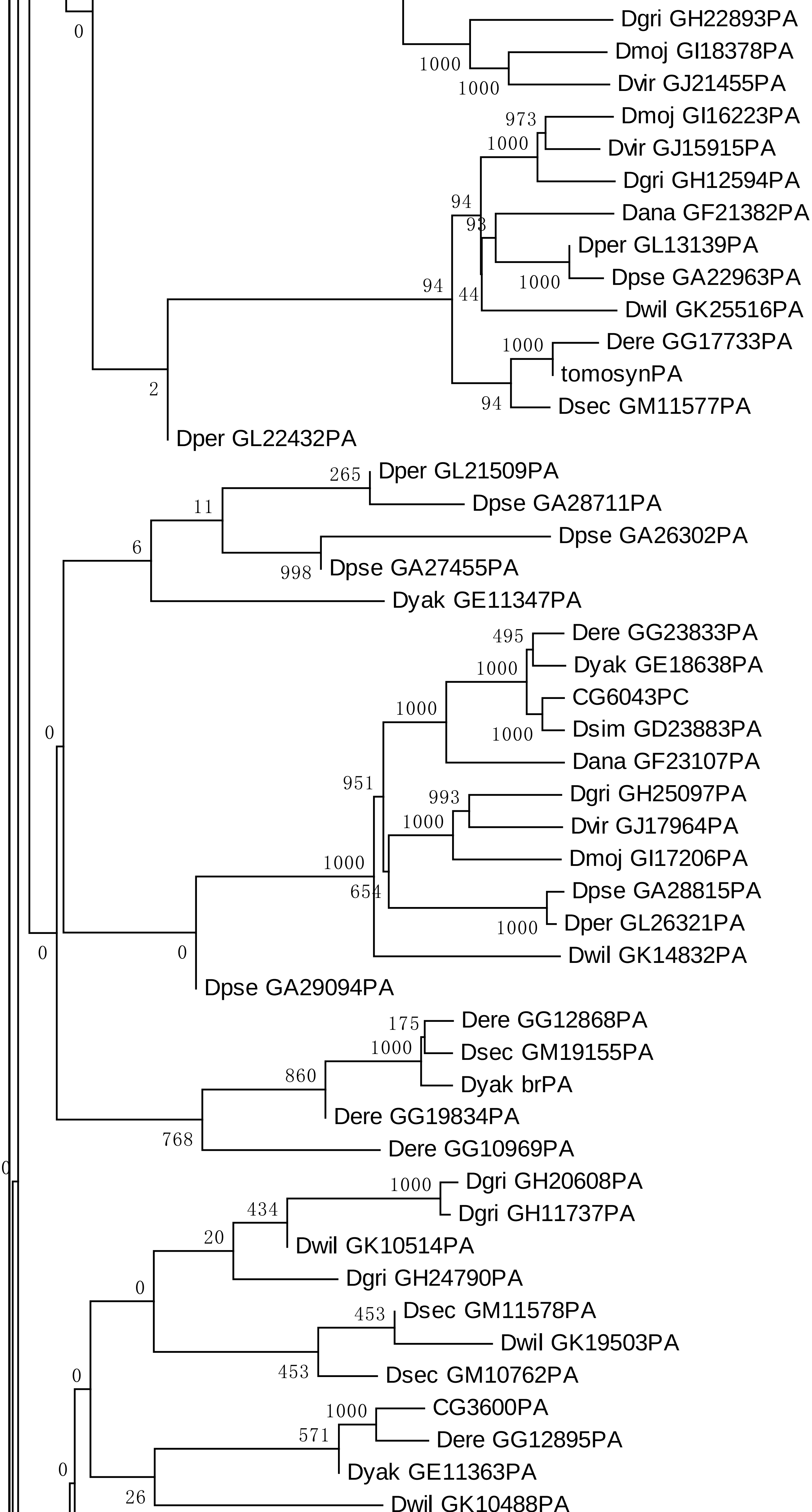
0
Dgri GH22893PA
1000
Dmoj GI18378PA
1000
Dvir GJ21455PA
973
Dmoj GI16223PA
1000
Dvir GJ15915PA
Dgri GH12594PA
94
Dana GF21382PA
93
Dper GL13139PA
1000
Dpse GA22963PA
94
44
Dwil GK25516PA
1000
Dere GG17733PA
tomosynPA
2
94
Dsec GM11577PA
Dper GL22432PA
265
Dper GL21509PA
Dpse GA28711PA
11
6
Dpse GA26302PA
998
Dpse GA27455PA
Dyak GE11347PA
495
Dere GG23833PA
1000
Dyak GE18638PA
1000
CG6043PC
0
1000
Dsim GD23883PA
Dana GF23107PA
951
Dgri GH25097PA
993
1000
Dvir GJ17964PA
Dmoj GI17206PA
1000
654
Dpse GA28815PA
1000
Dper GL26321PA
0
0
Dwil GK14832PA
Dpse GA29094PA
175
Dere GG12868PA
1000
Dsec GM19155PA
860
Dyak brPA
Dere GG19834PA
768
Dere GG10969PA
0
1000
Dgri GH20608PA
434
Dgri GH11737PA
20
Dwil GK10514PA
Dgri GH24790PA
0
453
Dsec GM11578PA
Dwil GK19503PA
0
453
Dsec GM10762PA
1000
CG3600PA
571
Dere GG12895PA
0
Dyak GE11363PA
26
Dwil GK10488PA

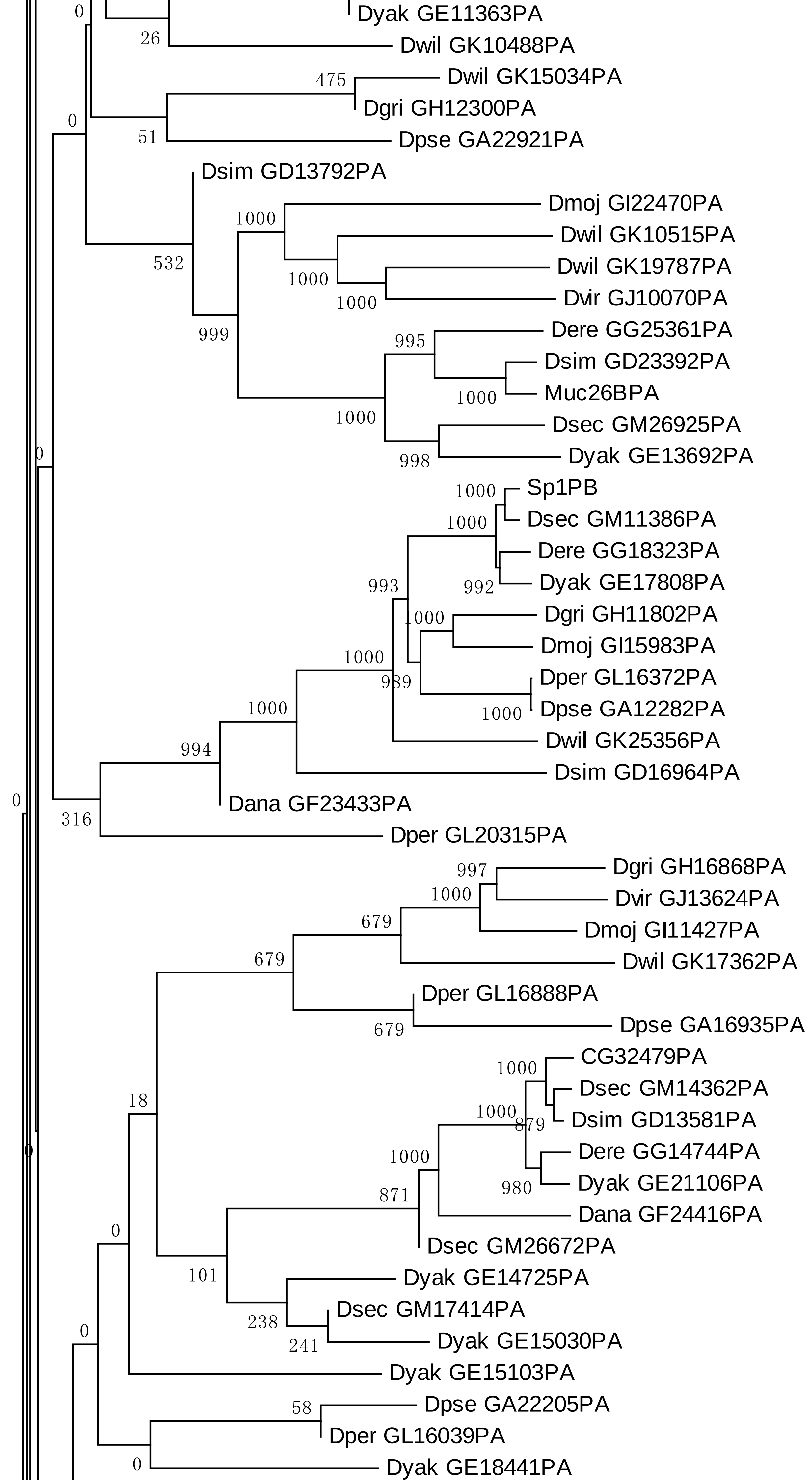
Dyak GE11363PA
0
26
Dwil GK10488PA
475
Dwil GK15034PA
Dgri GH12300PA
0
51
Dpse GA22921PA
Dsim GD13792PA
1000
Dmoj GI22470PA
Dwil GK10515PA
532
Dwil GK19787PA
1000
Dvir GJ10070PA
1000
999
995
Dere GG25361PA
Dsim GD23392PA
Muc26BPA
1000
1000
Dsec GM26925PA
0
Dyak GE13692PA
998
1000
Sp1PB
1000
Dsec GM11386PA
Dere GG18323PA
993
Dyak GE17808PA
992
1000
Dgri GH11802PA
Dmoj GI15983PA
1000
Dper GL16372PA
989
Dpse GA12282PA
1000
1000
Dwil GK25356PA
994
Dsim GD16964PA
0
Dana GF23433PA
316
Dper GL20315PA
997
Dgri GH16868PA
1000
Dvir GJ13624PA
679
Dmoj GI11427PA
679
Dwil GK17362PA
Dper GL16888PA
679
Dpse GA16935PA
CG32479PA
1000
Dsec GM14362PA
18
1000
Dsim GD13581PA
879
0
Dere GG14744PA
1000
Dyak GE21106PA
980
871
Dana GF24416PA
0
Dsec GM26672PA
101
Dyak GE14725PA
238
Dsec GM17414PA
0
241
Dyak GE15030PA
Dyak GE15103PA
58
Dpse GA22205PA
Dper GL16039PA
0
Dyak GE18441PA

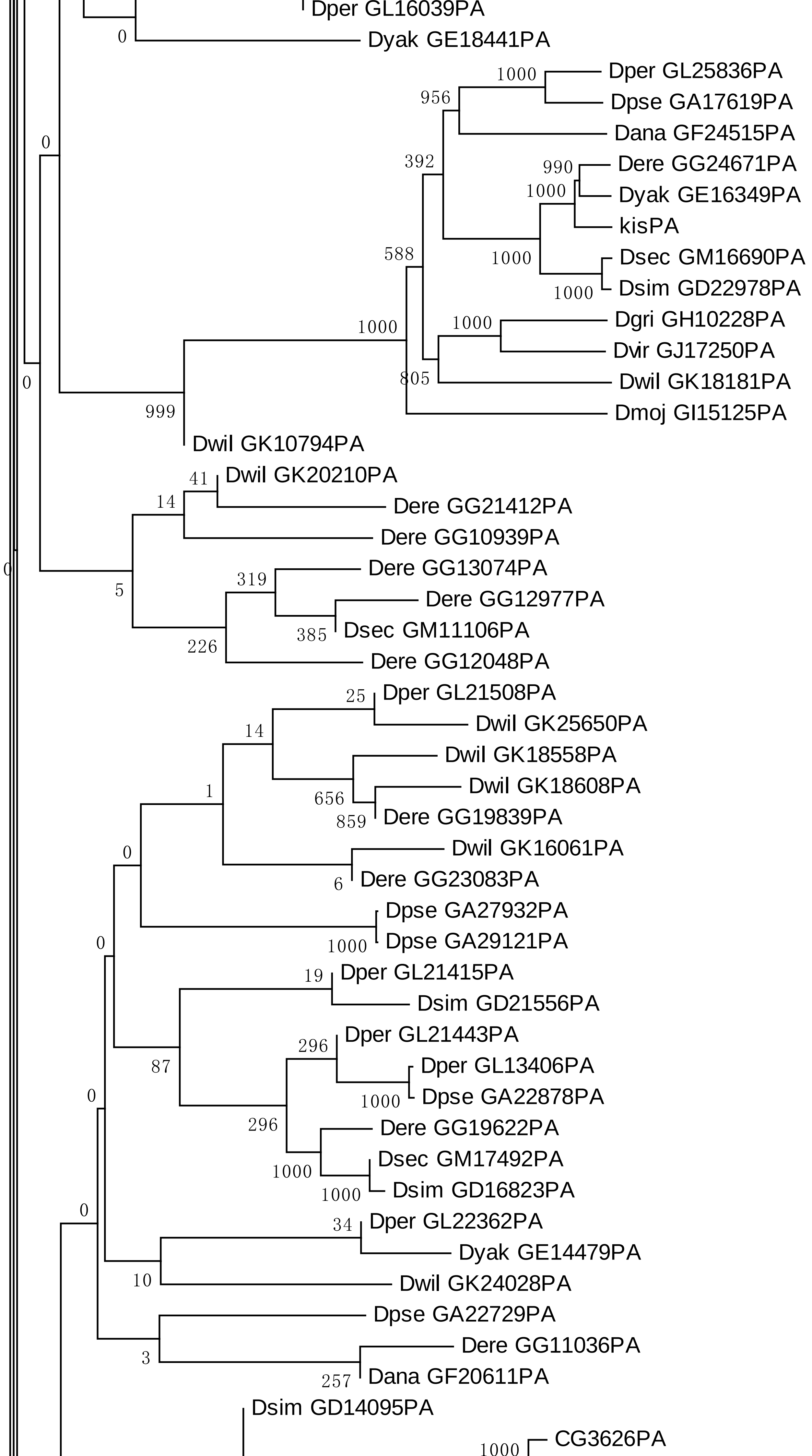
Dper GL16039PA
0
Dyak GE18441PA
1000
Dper GL25836PA
956
Dpse GA17619PA
0
Dana GF24515PA
392
990
Dere GG24671PA
1000
Dyak GE16349PA
kisPA
588
1000
Dsec GM16690PA
1000
Dsim GD22978PA
1000
1000
Dgri GH10228PA
Dvir GJ17250PA
0
805
Dwil GK18181PA
Dmoj GI15125PA
999
Dwil GK10794PA
41
Dwil GK20210PA
14
Dere GG21412PA
Dere GG10939PA
0
319
Dere GG13074PA
5
Dere GG12977PA
385
Dsec GM11106PA
226
Dere GG12048PA
25
Dper GL21508PA
14
Dwil GK25650PA
Dwil GK18558PA
1
656
Dwil GK18608PA
859
Dere GG19839PA
0
Dwil GK16061PA
6
Dere GG23083PA
Dpse GA27932PA
0
1000
Dpse GA29121PA
19
Dper GL21415PA
Dsim GD21556PA
296
Dper GL21443PA
87
Dper GL13406PA
1000
Dpse GA22878PA
0
296
Dere GG19622PA
1000
Dsec GM17492PA
1000
Dsim GD16823PA
0
34
Dper GL22362PA
Dyak GE14479PA
10
Dwil GK24028PA
Dpse GA22729PA
3
Dere GG11036PA
257
Dana GF20611PA
Dsim GD14095PA
1000
CG3626PA

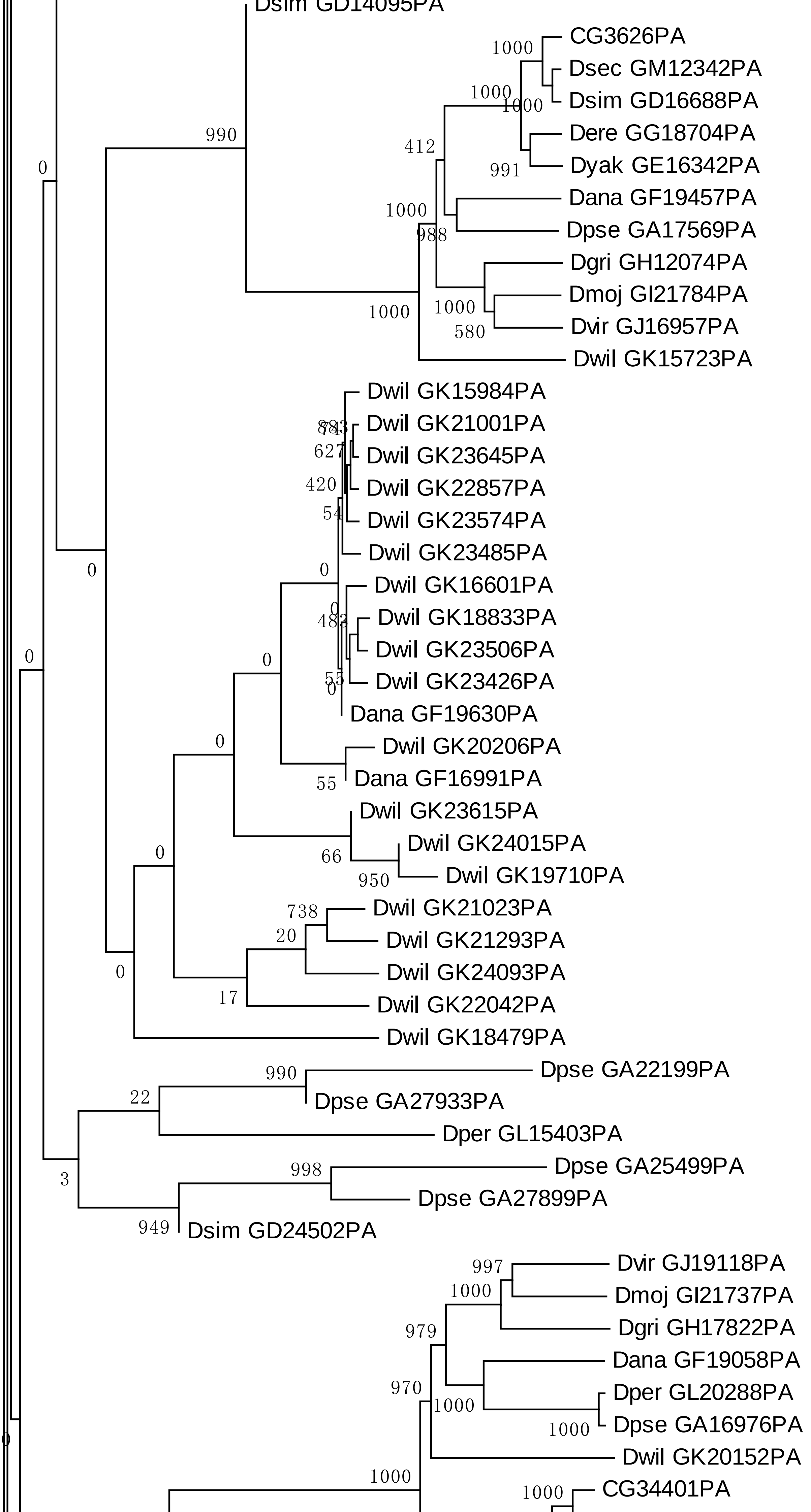

Dsim GD14095PA
CG3626PA
Dsec GM12342PA
Dsim GD16688PA
Dere GG18704PA
Dyak GE16342PA
Dana GF19457PA
Dpse GA17569PA
Dgri GH12074PA
Dmoj GI21784PA
Dvir GJ16957PA
Dwil GK15723PA
Dwil GK15984PA
Dwil GK21001PA
Dwil GK23645PA
Dwil GK22857PA
Dwil GK23574PA
Dwil GK23485PA
Dwil GK16601PA
Dwil GK18833PA
Dwil GK23506PA
Dwil GK23426PA
Dana GF19630PA
Dwil GK20206PA
Dana GF16991PA
Dwil GK23615PA
Dwil GK24015PA
Dwil GK19710PA
Dwil GK21023PA
Dwil GK21293PA
Dwil GK24093PA
Dwil GK22042PA
Dwil GK18479PA
Dpse GA22199PA
Dpse GA27933PA
Dper GL15403PA
Dpse GA25499PA
Dpse GA27899PA
Dsim GD24502PA
Dvir GJ19118PA
Dmoj GI21737PA
Dgri GH17822PA
Dana GF19058PA
Dper GL20288PA
Dpse GA16976PA
Dwil GK20152PA
CG34401PA
1000
1000
1000
990
412
991
0
1000
988
1000
1000
580
627
420
54
0
0
0
0
55
55
66
950
738
20
17
0
0
22
990
3
998
949
997
1000
979
970
1000
1000
1000
1000
0

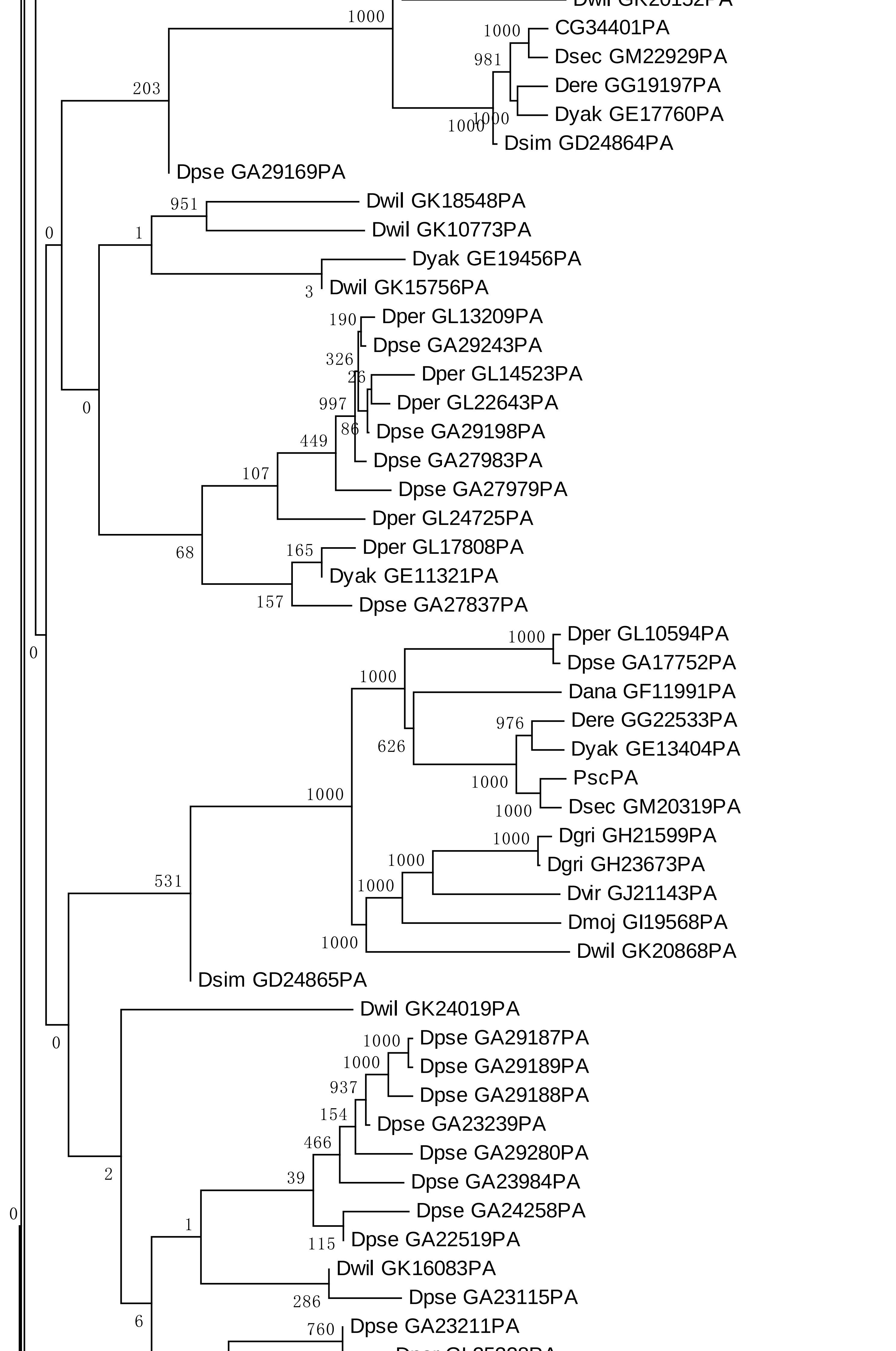

CG34401PA
Dsec GM22929PA
Dere GG19197PA
Dyak GE17760PA
Dsim GD24864PA
Dpse GA29169PA
Dwil GK18548PA
Dwil GK10773PA
Dyak GE19456PA
Dwil GK15756PA
Dper GL13209PA
Dpse GA29243PA
Dper GL14523PA
Dper GL22643PA
Dpse GA29198PA
Dpse GA27983PA
Dpse GA27979PA
Dper GL24725PA
Dper GL17808PA
Dyak GE11321PA
Dpse GA27837PA
Dper GL10594PA
Dpse GA17752PA
Dana GF11991PA
Dere GG22533PA
Dyak GE13404PA
PscPA
Dsec GM20319PA
Dgri GH21599PA
Dgri GH23673PA
Dvir GJ21143PA
Dmoj GI19568PA
Dwil GK20868PA
Dsim GD24865PA
Dwil GK24019PA
Dpse GA29187PA
Dpse GA29189PA
Dpse GA29188PA
Dpse GA23239PA
Dpse GA29280PA
Dpse GA23984PA
Dpse GA24258PA
Dpse GA22519PA
Dwil GK16083PA
Dpse GA23115PA
Dpse GA23211PA

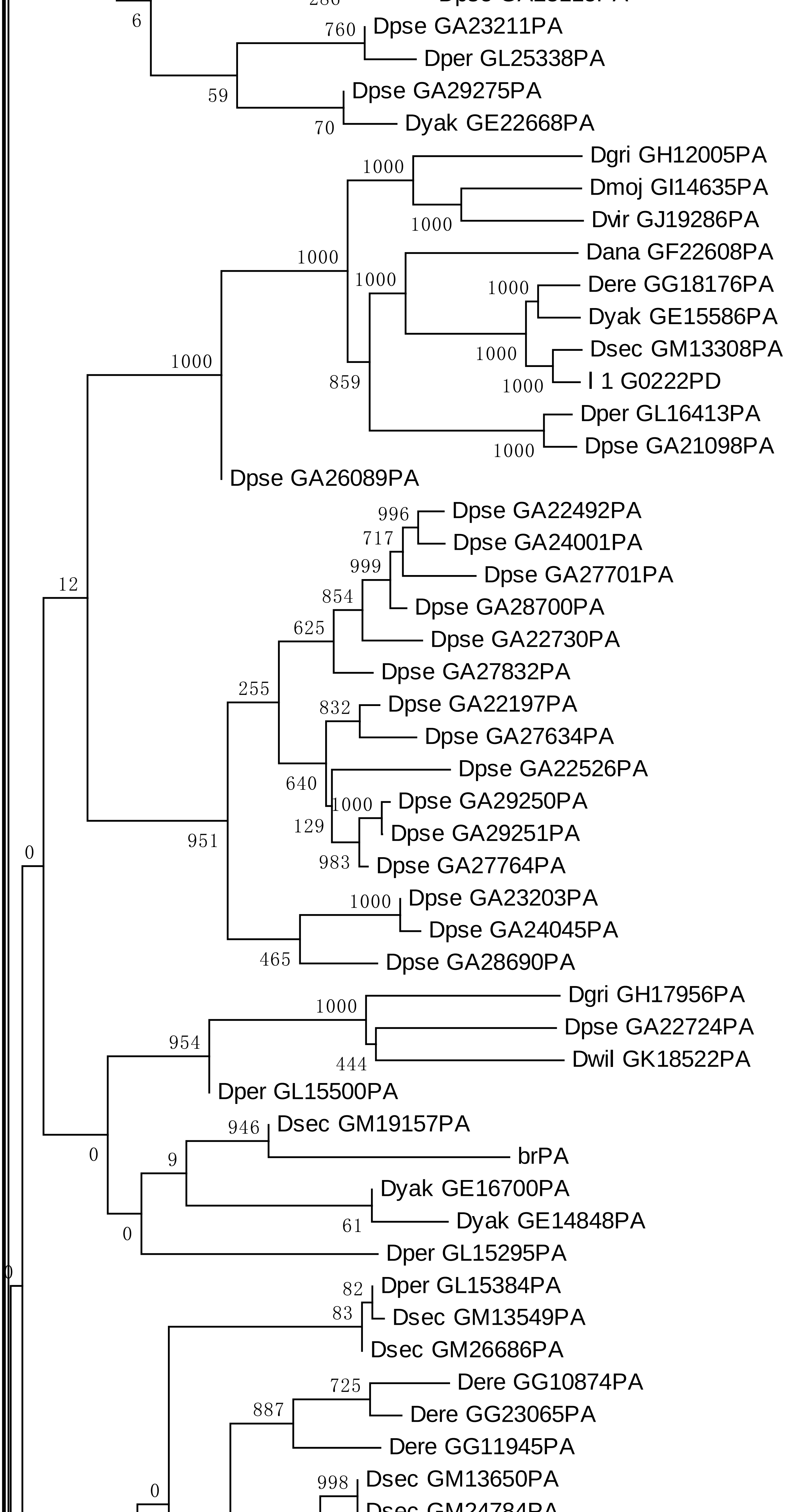

6
760
Dpse GA23211PA
Dper GL25338PA
59
Dpse GA29275PA
70
Dyak GE22668PA
1000
Dgri GH12005PA
Dmoj GI14635PA
1000
Dvir GJ19286PA
1000
Dana GF22608PA
1000
1000
Dere GG18176PA
Dyak GE15586PA
1000
1000
Dsec GM13308PA
859
1000
l 1 G0222PD
Dper GL16413PA
1000
Dpse GA21098PA
Dpse GA26089PA
996
Dpse GA22492PA
717
Dpse GA24001PA
999
Dpse GA27701PA
854
Dpse GA28700PA
625
Dpse GA22730PA
Dpse GA27832PA
255
832
Dpse GA22197PA
Dpse GA27634PA
12
Dpse GA22526PA
640
1000
Dpse GA29250PA
129
Dpse GA29251PA
951
983
Dpse GA27764PA
0
1000
Dpse GA23203PA
Dpse GA24045PA
465
Dpse GA28690PA
1000
Dgri GH17956PA
Dpse GA22724PA
954
444
Dwil GK18522PA
Dper GL15500PA
946
Dsec GM19157PA
0
9
brPA
Dyak GE16700PA
61
Dyak GE14848PA
0
Dper GL15295PA
0
82
Dper GL15384PA
83
Dsec GM13549PA
Dsec GM26686PA
725
Dere GG10874PA
887
Dere GG23065PA
Dere GG11945PA
998
Dsec GM13650PA
0

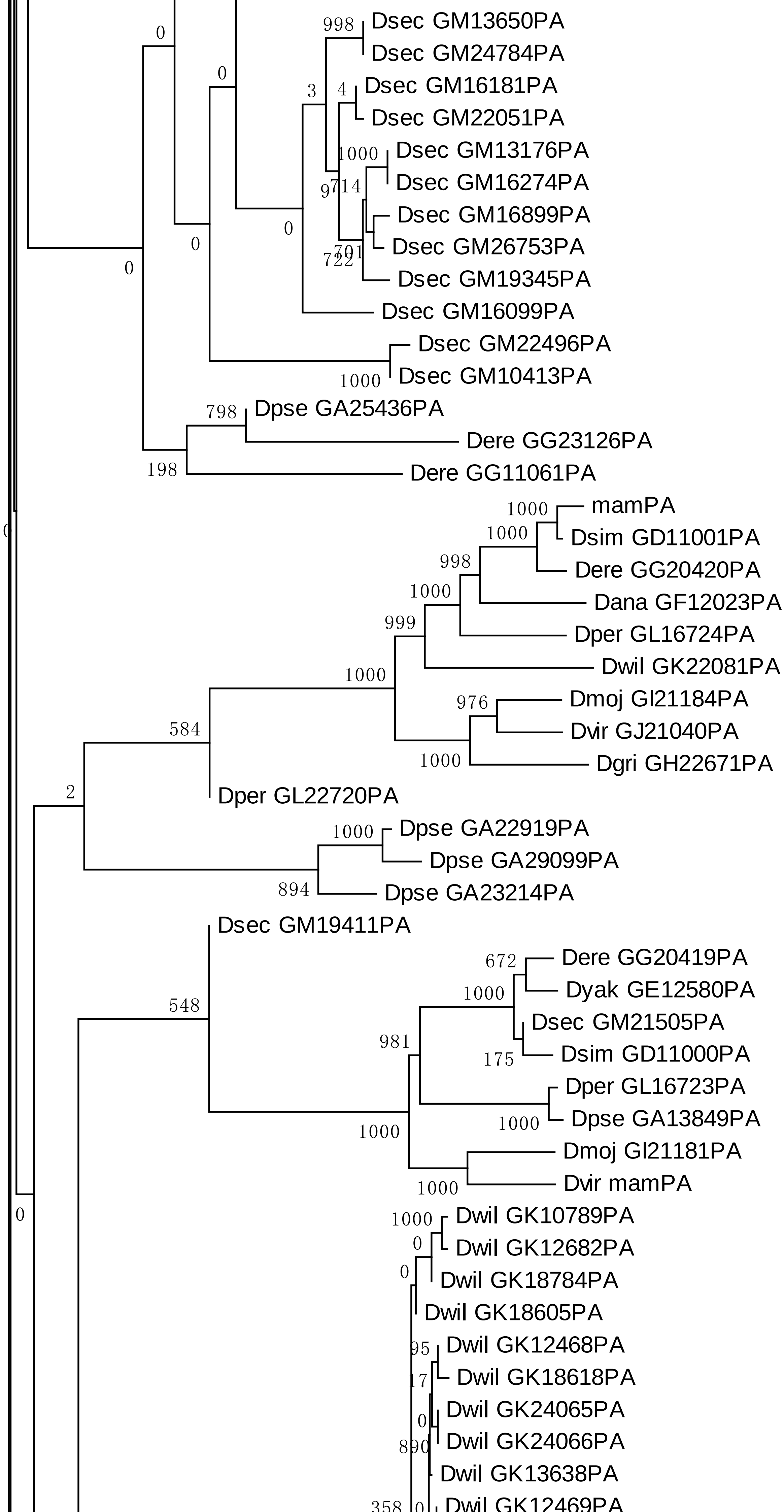

Dsec GM13650PA
Dsec GM24784PA
Dsec GM16181PA
Dsec GM22051PA
Dsec GM13176PA
Dsec GM16274PA
Dsec GM16899PA
Dsec GM26753PA
Dsec GM19345PA
Dsec GM16099PA
Dsec GM22496PA
Dsec GM10413PA
Dpse GA25436PA
Dere GG23126PA
Dere GG11061PA
mamPA
Dsim GD11001PA
Dere GG20420PA
Dana GF12023PA
Dper GL16724PA
Dwil GK22081PA
Dmoj GI21184PA
Dvir GJ21040PA
Dgri GH22671PA
Dper GL22720PA
Dpse GA22919PA
Dpse GA29099PA
Dpse GA23214PA
Dsec GM19411PA
Dere GG20419PA
Dyak GE12580PA
Dsec GM21505PA
Dsim GD11000PA
Dper GL16723PA
Dpse GA13849PA
Dmoj GI21181PA
Dvir mamPA
Dwil GK10789PA
Dwil GK12682PA
Dwil GK18784PA
Dwil GK18605PA
Dwil GK12468PA
Dwil GK18618PA
Dwil GK24065PA
Dwil GK24066PA
Dwil GK13638PA
Dwil GK12469PA

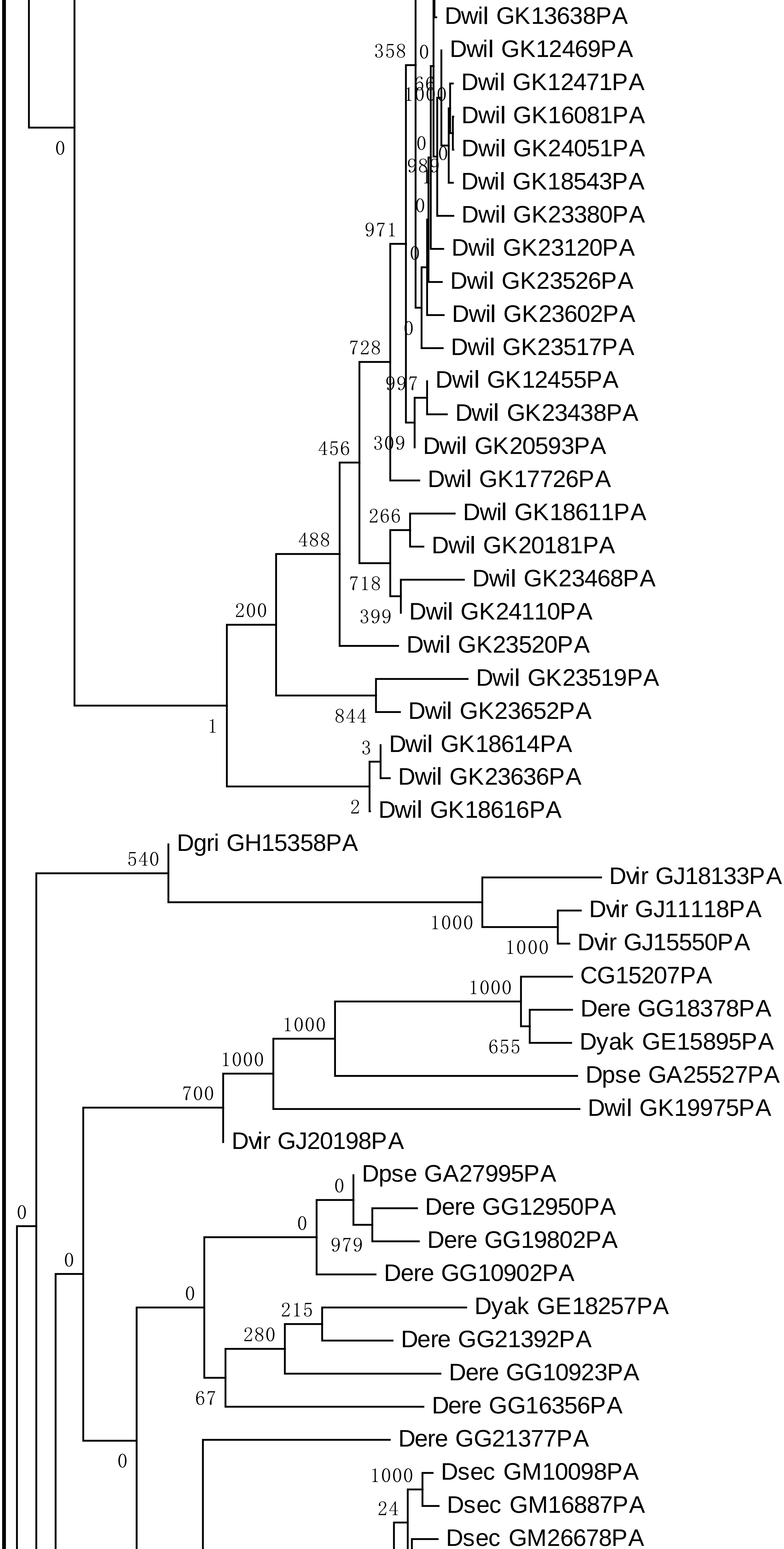

Dwil GK13638PA
358
0
Dwil GK12469PA
Dwil GK12471PA
Dwil GK16081PA
Dwil GK24051PA
Dwil GK18543PA
Dwil GK23380PA
Dwil GK23120PA
Dwil GK23526PA
Dwil GK23602PA
Dwil GK23517PA
0
971
0
728
Dwil GK12455PA
997
Dwil GK23438PA
309
Dwil GK20593PA
456
Dwil GK17726PA
266
Dwil GK18611PA
488
Dwil GK20181PA
718
Dwil GK23468PA
399
Dwil GK24110PA
200
Dwil GK23520PA
Dwil GK23519PA
844
Dwil GK23652PA
1
3
Dwil GK18614PA
Dwil GK23636PA
2
Dwil GK18616PA
540
Dgri GH15358PA
Dvir GJ18133PA
1000
Dvir GJ11118PA
1000
Dvir GJ15550PA
1000
CG15207PA
Dere GG18378PA
655
Dyak GE15895PA
1000
Dpse GA25527PA
1000
700
Dwil GK19975PA
Dvir GJ20198PA
0
Dpse GA27995PA
Dere GG12950PA
0
979
Dere GG19802PA
Dere GG10902PA
0
0
0
215
Dyak GE18257PA
280
Dere GG21392PA
Dere GG10923PA
67
Dere GG16356PA
Dere GG21377PA
0
1000
Dsec GM10098PA
24
Dsec GM16887PA
Dsec GM26678PA

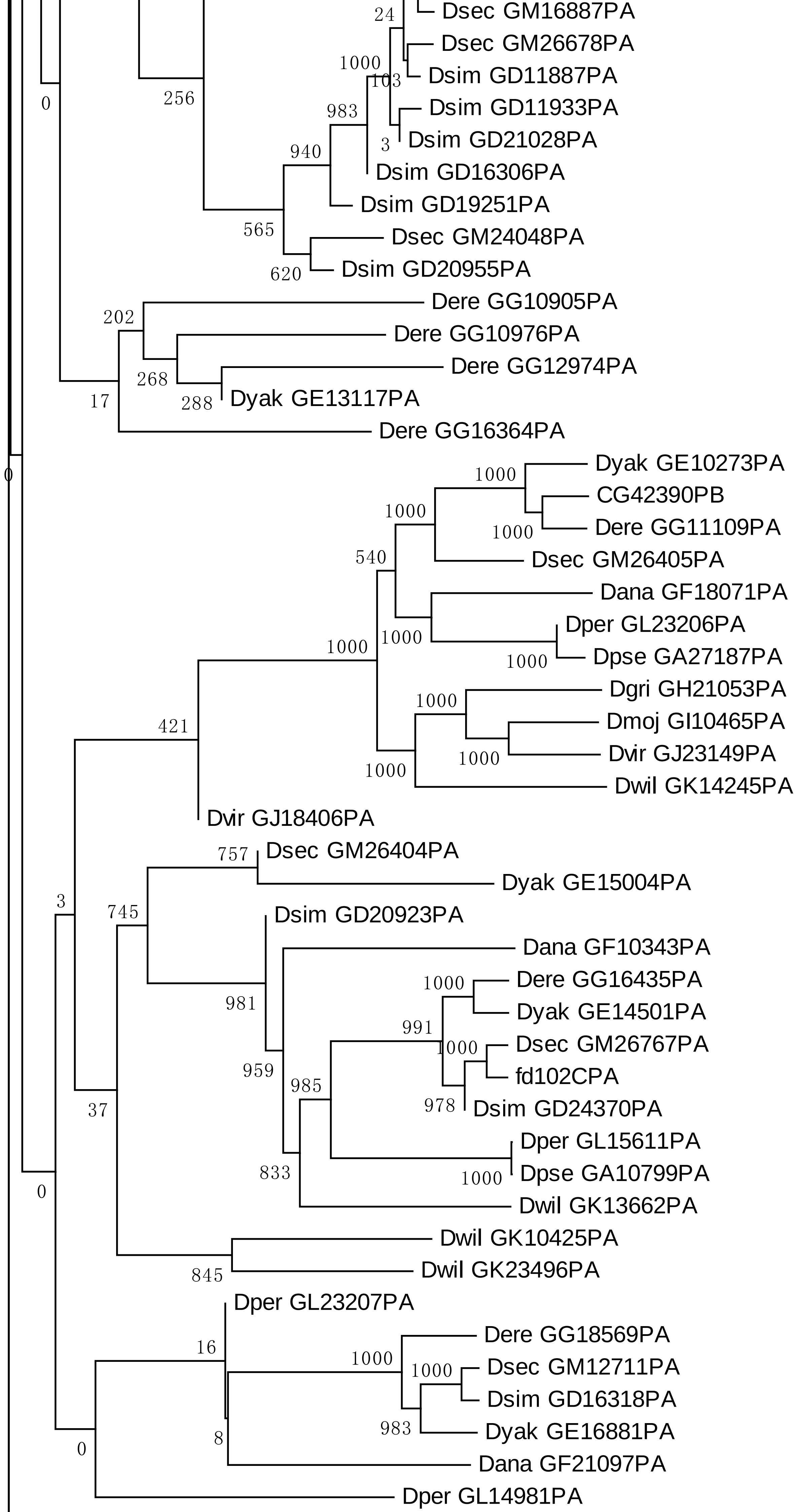
24
Dsec GM16887PA
Dsec GM26678PA
1000
103
Dsim GD11887PA
0
256
983
Dsim GD11933PA
3
Dsim GD21028PA
940
Dsim GD16306PA
Dsim GD19251PA
565
Dsec GM24048PA
620
Dsim GD20955PA
202
Dere GG10905PA
Dere GG10976PA
Dere GG12974PA
268
17
288
Dyak GE13117PA
Dere GG16364PA
0
Dyak GE10273PA
1000
CG42390PB
1000
Dere GG11109PA
1000
540
Dsec GM26405PA
Dana GF18071PA
Dper GL23206PA
1000
1000
1000
Dpse GA27187PA
1000
Dgri GH21053PA
421
Dmoj GI10465PA
Dvir GJ23149PA
1000
1000
Dwil GK14245PA
Dvir GJ18406PA
757
Dsec GM26404PA
Dyak GE15004PA
3
745
Dsim GD20923PA
Dana GF10343PA
1000
Dere GG16435PA
981
Dyak GE14501PA
991
Dsec GM26767PA
1000
959
fd102CPA
985
37
978
Dsim GD24370PA
Dper GL15611PA
833
1000
Dpse GA10799PA
0
Dwil GK13662PA
Dwil GK10425PA
845
Dwil GK23496PA
Dper GL23207PA
16
Dere GG18569PA
1000
1000
Dsec GM12711PA
Dsim GD16318PA
983
Dyak GE16881PA
8
0
Dana GF21097PA
Dper GL14981PA

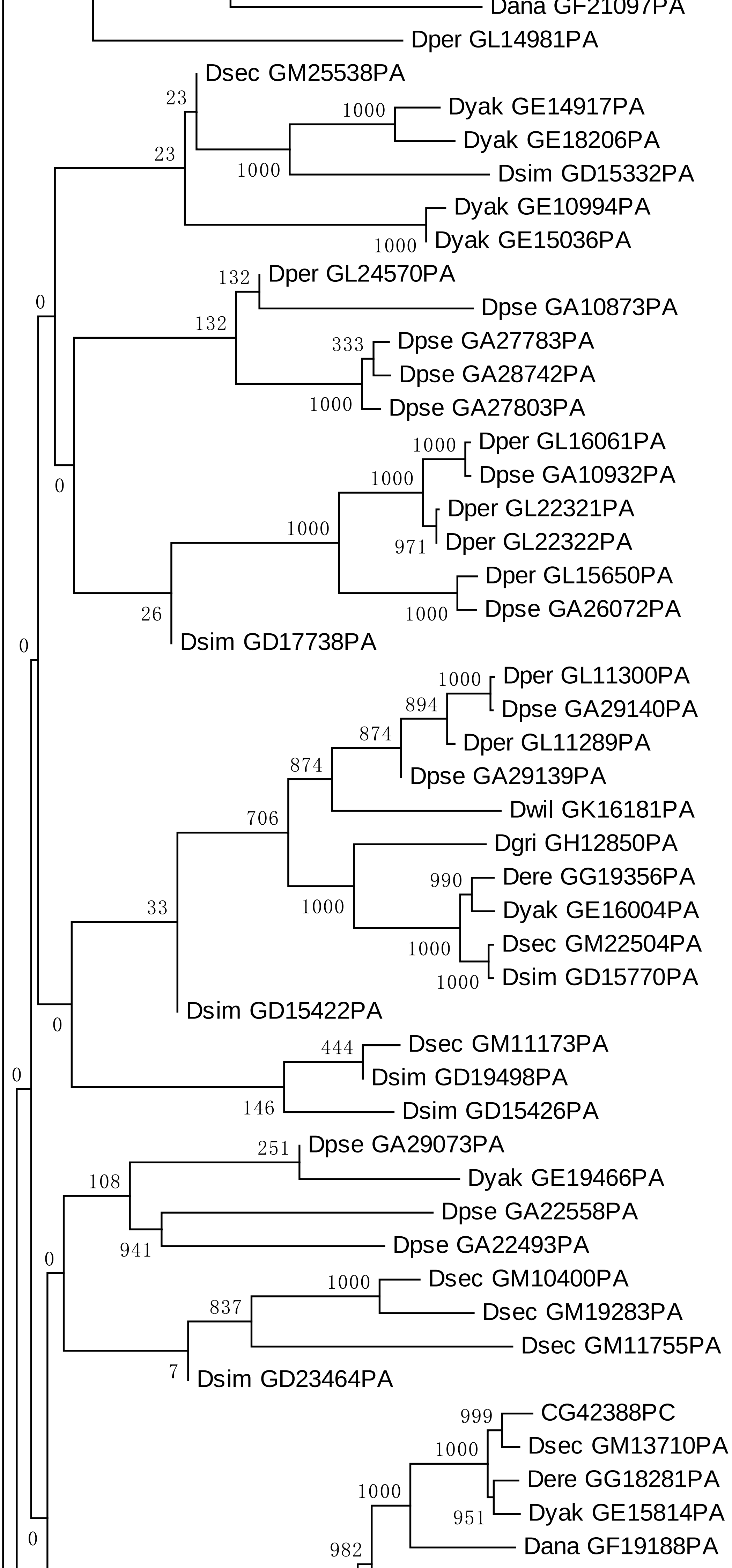
Dana GF21097PA
Dper GL14981PA
Dsec GM25538PA
Dyak GE14917PA
Dyak GE18206PA
Dsim GD15332PA
Dyak GE10994PA
Dyak GE15036PA
Dper GL24570PA
Dpse GA10873PA
Dpse GA27783PA
Dpse GA28742PA
Dpse GA27803PA
Dper GL16061PA
Dpse GA10932PA
Dper GL22321PA
Dper GL22322PA
Dper GL15650PA
Dpse GA26072PA
Dsim GD17738PA
Dper GL11300PA
Dpse GA29140PA
Dper GL11289PA
Dpse GA29139PA
Dwil GK16181PA
Dgri GH12850PA
Dere GG19356PA
Dyak GE16004PA
Dsec GM22504PA
Dsim GD15770PA
Dsim GD15422PA
Dsec GM11173PA
Dsim GD19498PA
Dsim GD15426PA
Dpse GA29073PA
Dyak GE19466PA
Dpse GA22558PA
Dpse GA22493PA
Dsec GM10400PA
Dsec GM19283PA
Dsec GM11755PA
Dsim GD23464PA
CG42388PC
Dsec GM13710PA
Dere GG18281PA
Dyak GE15814PA
Dana GF19188PA

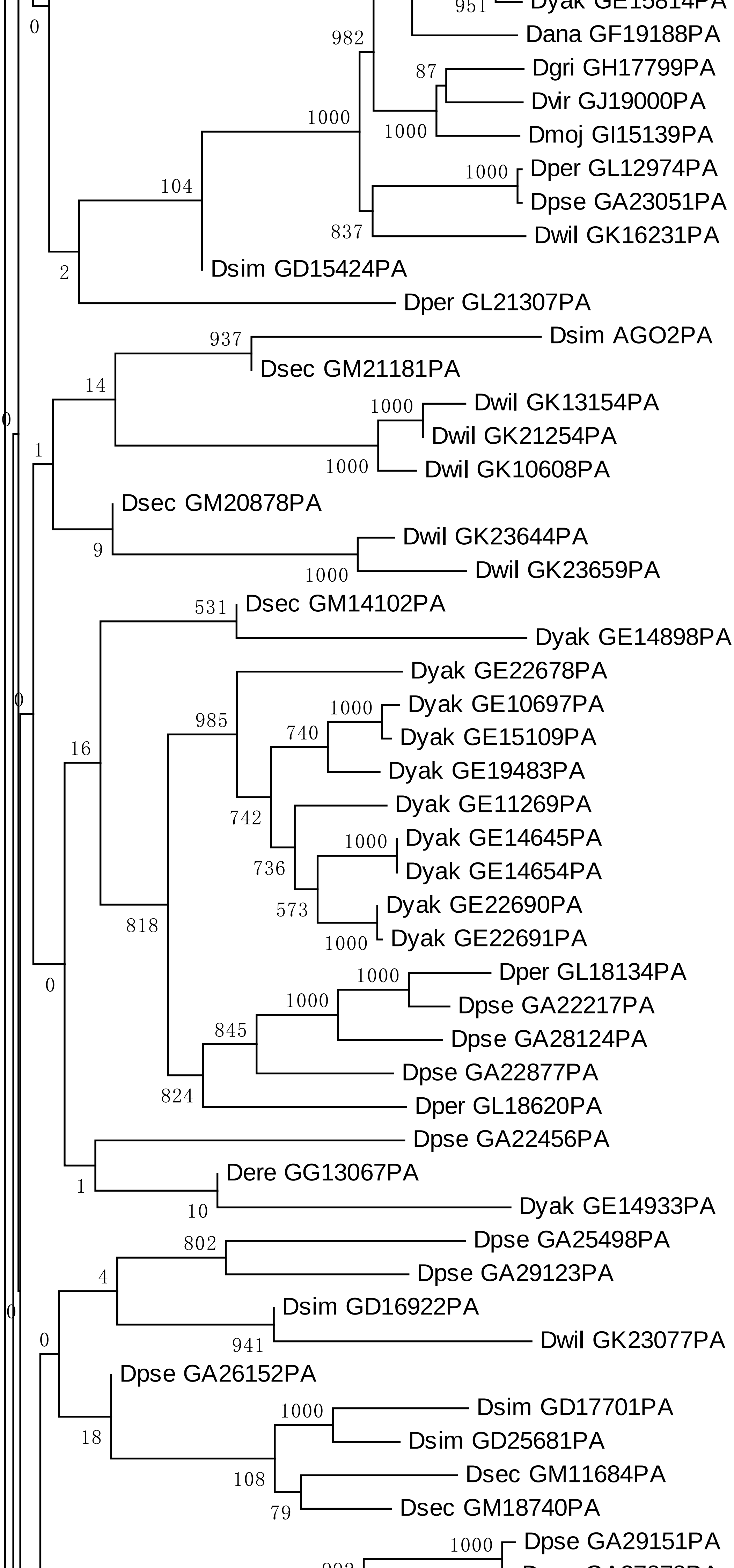
0
Dana GF19188PA
982
87
Dgri GH17799PA
Dvir GJ19000PA
1000
1000
Dmoj GI15139PA
1000
Dper GL12974PA
104
Dpse GA23051PA
837
Dwil GK16231PA
2
Dsim GD15424PA
Dper GL21307PA
937
Dsim AGO2PA
Dsec GM21181PA
14
1000
Dwil GK13154PA
0
Dwil GK21254PA
1
1000
Dwil GK10608PA
Dsec GM20878PA
Dwil GK23644PA
9
1000
Dwil GK23659PA
531
Dsec GM14102PA
Dyak GE14898PA
Dyak GE22678PA
1000
Dyak GE10697PA
0
985
740
Dyak GE15109PA
16
Dyak GE19483PA
742
Dyak GE11269PA
1000
Dyak GE14645PA
736
Dyak GE14654PA
573
Dyak GE22690PA
818
1000
Dyak GE22691PA
1000
Dper GL18134PA
0
1000
Dpse GA22217PA
845
Dpse GA28124PA
Dpse GA22877PA
824
Dper GL18620PA
Dpse GA22456PA
Dere GG13067PA
1
10
Dyak GE14933PA
802
Dpse GA25498PA
4
Dpse GA29123PA
Dsim GD16922PA
0
0
941
Dwil GK23077PA
Dpse GA26152PA
1000
Dsim GD17701PA
18
Dsim GD25681PA
108
Dsec GM11684PA
79
Dsec GM18740PA
1000
Dpse GA29151PA

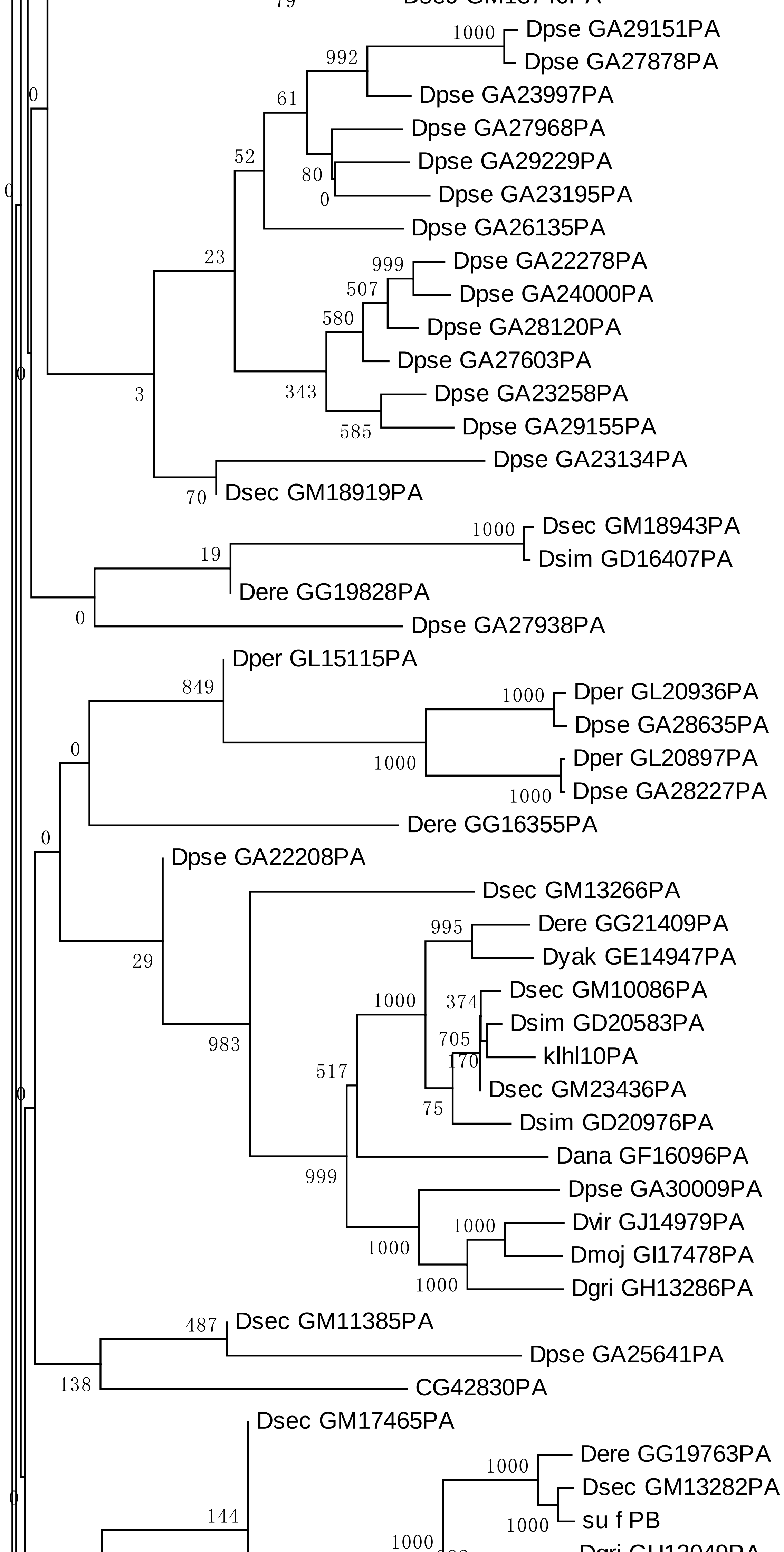

79
1000
Dpse GA29151PA
Dpse GA27878PA
992
Dpse GA23997PA
0
61
Dpse GA27968PA
52
Dpse GA29229PA
0
80
0
Dpse GA23195PA
Dpse GA26135PA
23
999
Dpse GA22278PA
507
Dpse GA24000PA
580
Dpse GA28120PA
Dpse GA27603PA
0
3
343
Dpse GA23258PA
585
Dpse GA29155PA
Dpse GA23134PA
70
Dsec GM18919PA
1000
Dsec GM18943PA
19
Dsim GD16407PA
Dere GG19828PA
0
Dpse GA27938PA
Dper GL15115PA
849
1000
Dper GL20936PA
Dpse GA28635PA
0
1000
Dper GL20897PA
1000
Dpse GA28227PA
0
Dere GG16355PA
Dpse GA22208PA
Dsec GM13266PA
995
Dere GG21409PA
29
Dyak GE14947PA
1000
374
Dsec GM10086PA
983
705
Dsim GD20583PA
517
170
klhl10PA
Dsec GM23436PA
0
75
Dsim GD20976PA
Dana GF16096PA
999
Dpse GA30009PA
1000
Dvir GJ14979PA
1000
Dmoj GI17478PA
1000
Dgri GH13286PA
487
Dsec GM11385PA
Dpse GA25641PA
138
CG42830PA
Dsec GM17465PA
1000
Dere GG19763PA
0
Dsec GM13282PA
144
1000
su f PB
1000

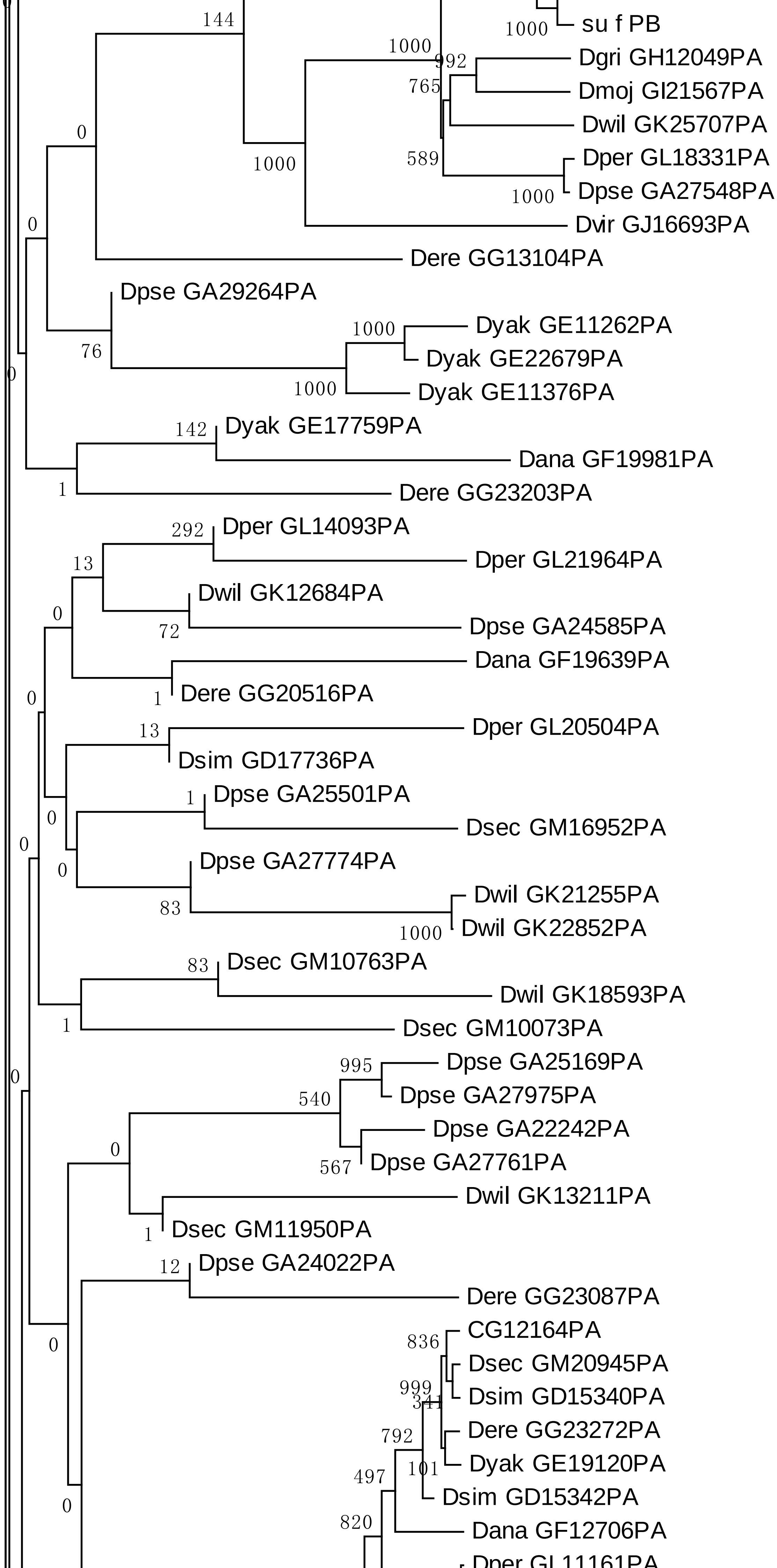

su f PB
Dgri GH12049PA
Dmoj GI21567PA
Dwil GK25707PA
Dper GL18331PA
Dpse GA27548PA
Dvir GJ16693PA
Dere GG13104PA
Dpse GA29264PA
Dyak GE11262PA
Dyak GE22679PA
Dyak GE11376PA
Dyak GE17759PA
Dana GF19981PA
Dere GG23203PA
Dper GL14093PA
Dper GL21964PA
Dwil GK12684PA
Dpse GA24585PA
Dana GF19639PA
Dere GG20516PA
Dper GL20504PA
Dsim GD17736PA
Dpse GA25501PA
Dsec GM16952PA
Dpse GA27774PA
Dwil GK21255PA
Dwil GK22852PA
Dsec GM10763PA
Dwil GK18593PA
Dsec GM10073PA
Dpse GA25169PA
Dpse GA27975PA
Dpse GA22242PA
Dpse GA27761PA
Dwil GK13211PA
Dsec GM11950PA
Dpse GA24022PA
Dere GG23087PA
CG12164PA
Dsec GM20945PA
Dsim GD15340PA
Dere GG23272PA
Dyak GE19120PA
Dsim GD15342PA
Dana GF12706PA
144
1000
1000
992
765
0
1000
589
1000
0
1000
76
1000
0
142
1
292
13
0
72
1
0
13
1
0
0
0
83
1000
83
1
995
0
540
567
0
1
12
0
836
999
341
792
101
497
820

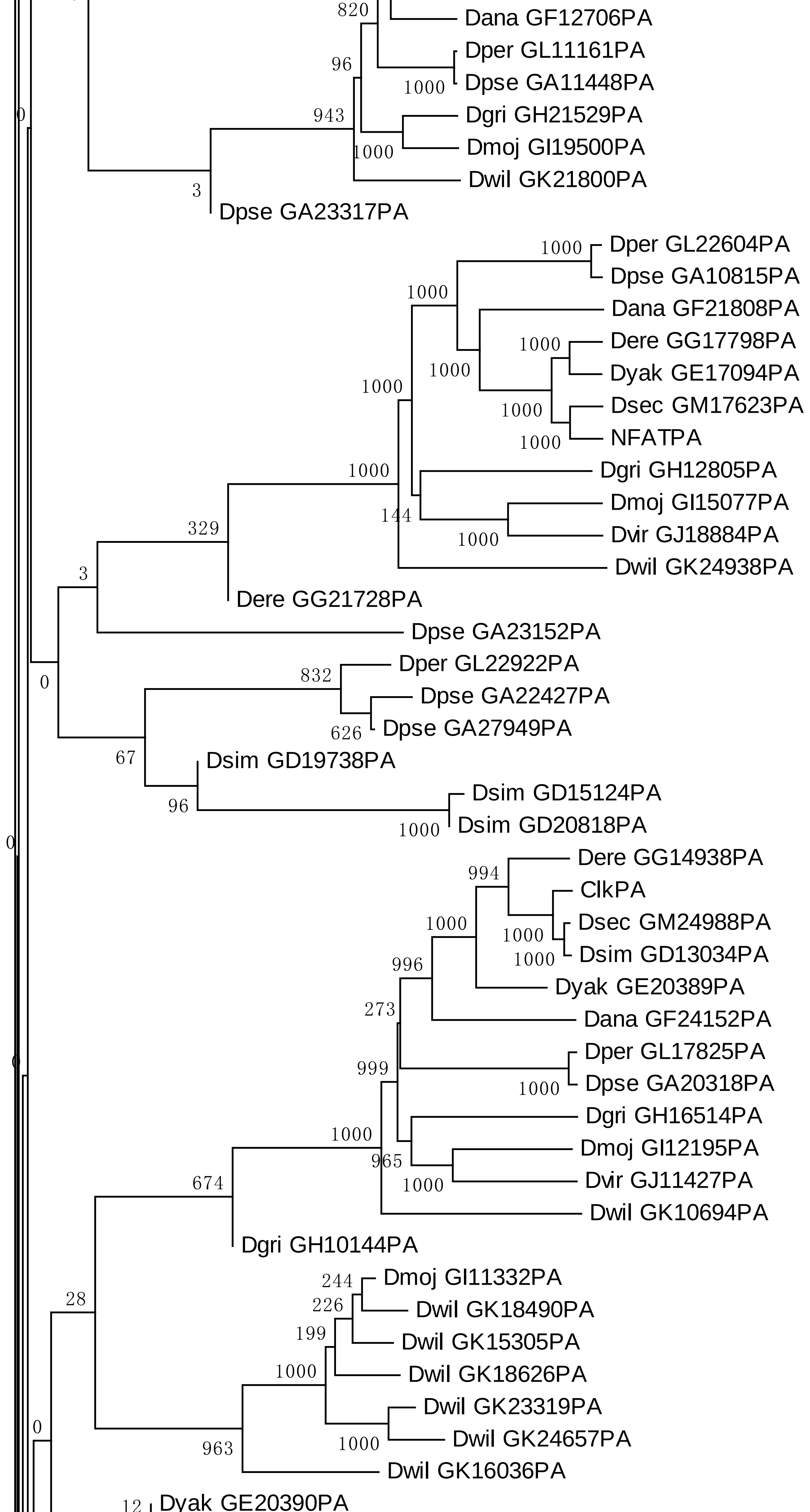

Dana GF12706PA
Dper GL11161PA
Dpse GA11448PA
Dgri GH21529PA
Dmoj GI19500PA
Dwil GK21800PA
Dpse GA23317PA
Dper GL22604PA
Dpse GA10815PA
Dana GF21808PA
Dere GG17798PA
Dyak GE17094PA
Dsec GM17623PA
NFATPA
Dgri GH12805PA
Dmoj GI15077PA
Dvir GJ18884PA
Dwil GK24938PA
Dere GG21728PA
Dpse GA23152PA
Dper GL22922PA
Dpse GA22427PA
Dpse GA27949PA
Dsim GD19738PA
Dsim GD15124PA
Dsim GD20818PA
Dere GG14938PA
ClkPA
Dsec GM24988PA
Dsim GD13034PA
Dyak GE20389PA
Dana GF24152PA
Dper GL17825PA
Dpse GA20318PA
Dgri GH16514PA
Dmoj GI12195PA
Dvir GJ11427PA
Dwil GK10694PA
Dgri GH10144PA
Dmoj GI11332PA
Dwil GK18490PA
Dwil GK15305PA
Dwil GK18626PA
Dwil GK23319PA
Dwil GK24657PA
Dwil GK16036PA
Dyak GE20390PA

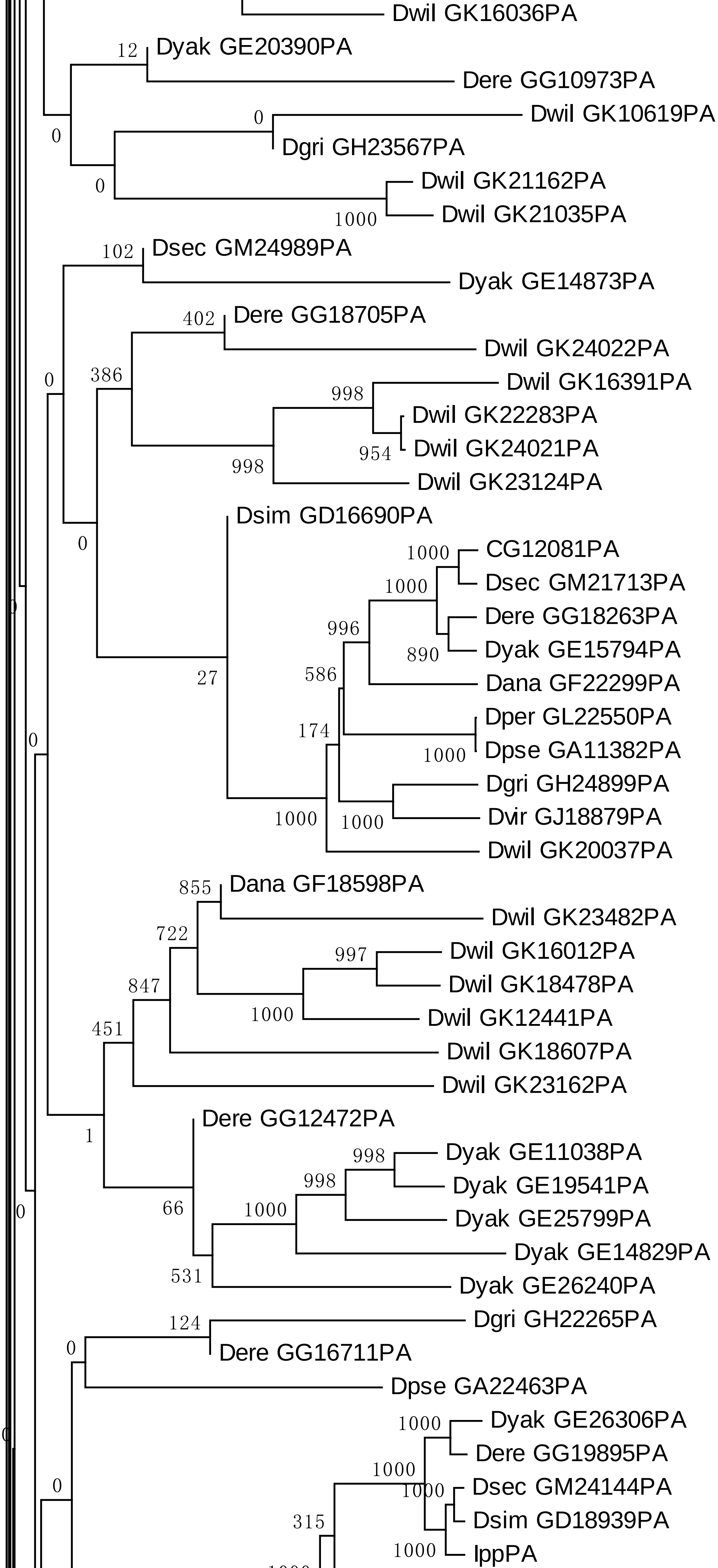

Dwil GK16036PA
12
Dyak GE20390PA
Dere GG10973PA
0
Dwil GK10619PA
0
Dgri GH23567PA
0
Dwil GK21162PA
1000
Dwil GK21035PA
102
Dsec GM24989PA
Dyak GE14873PA
402
Dere GG18705PA
Dwil GK24022PA
0
386
998
Dwil GK16391PA
Dwil GK22283PA
954
Dwil GK24021PA
998
Dwil GK23124PA
Dsim GD16690PA
0
1000
CG12081PA
Dsec GM21713PA
1000
996
Dere GG18263PA
890
Dyak GE15794PA
27
586
Dana GF22299PA
174
Dper GL22550PA
0
1000
Dpse GA11382PA
Dgri GH24899PA
1000
1000
Dvir GJ18879PA
Dwil GK20037PA
855
Dana GF18598PA
Dwil GK23482PA
722
997
Dwil GK16012PA
847
Dwil GK18478PA
1000
Dwil GK12441PA
451
Dwil GK18607PA
Dwil GK23162PA
Dere GG12472PA
1
998
Dyak GE11038PA
998
Dyak GE19541PA
0
66
1000
Dyak GE25799PA
Dyak GE14829PA
531
Dyak GE26240PA
124
Dgri GH22265PA
0
Dere GG16711PA
Dpse GA22463PA
1000
Dyak GE26306PA
Dere GG19895PA
1000
1000
Dsec GM24144PA
0
315
Dsim GD18939PA
1000
IppPA

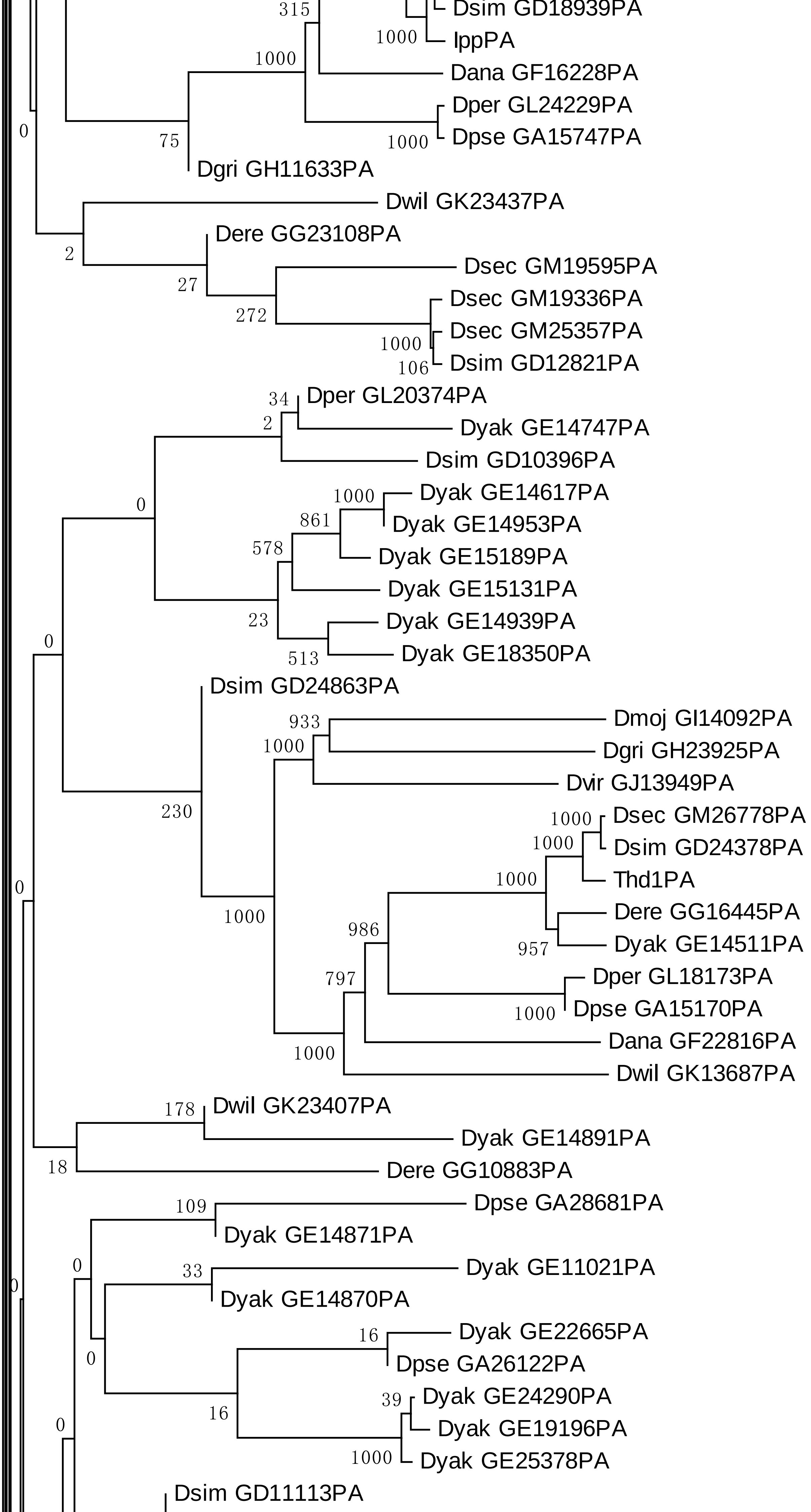
Dsim GD18939PA
315
1000
IppPA
1000
Dana GF16228PA
Dper GL24229PA
0
75
1000
Dpse GA15747PA
Dgri GH11633PA
Dwil GK23437PA
Dere GG23108PA
2
Dsec GM19595PA
27
Dsec GM19336PA
272
Dsec GM25357PA
1000
106
Dsim GD12821PA
34
Dper GL20374PA
2
Dyak GE14747PA
Dsim GD10396PA
1000
Dyak GE14617PA
0
861
Dyak GE14953PA
578
Dyak GE15189PA
Dyak GE15131PA
23
Dyak GE14939PA
0
513
Dyak GE18350PA
Dsim GD24863PA
933
Dmoj GI14092PA
1000
Dgri GH23925PA
Dvir GJ13949PA
230
1000
Dsec GM26778PA
1000
Dsim GD24378PA
1000
Thd1PA
0
Dere GG16445PA
1000
986
Dyak GE14511PA
957
797
Dper GL18173PA
1000
Dpse GA15170PA
1000
Dana GF22816PA
Dwil GK13687PA
178
Dwil GK23407PA
Dyak GE14891PA
18
Dere GG10883PA
109
Dpse GA28681PA
Dyak GE14871PA
0
33
Dyak GE11021PA
0
Dyak GE14870PA
16
Dyak GE22665PA
0
Dpse GA26122PA
16
39
Dyak GE24290PA
0
Dyak GE19196PA
1000
Dyak GE25378PA
Dsim GD11113PA

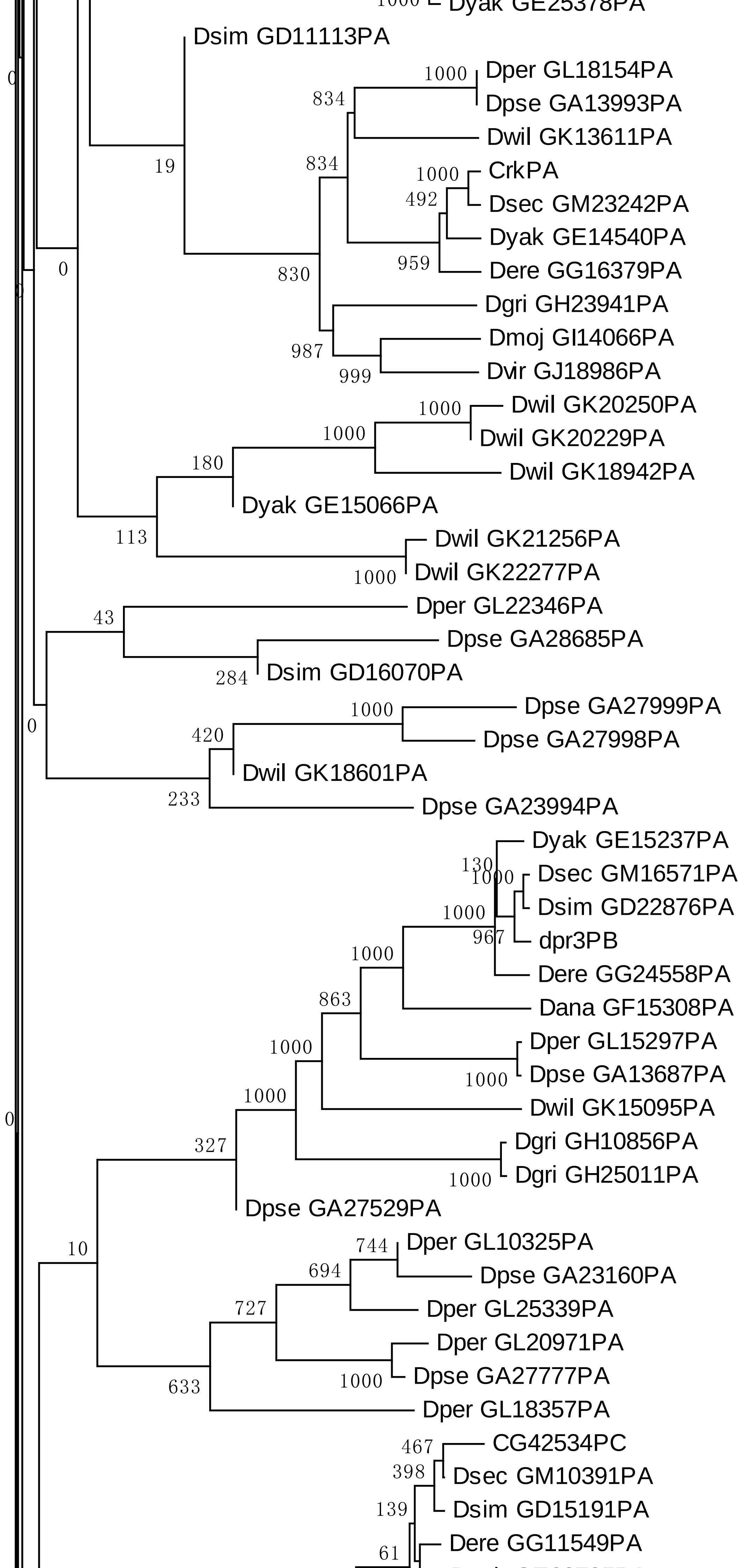
Dyak GE25378PA
1000
Dsim GD11113PA
Dper GL18154PA
Dpse GA13993PA
Dwil GK13611PA
CrkPA
Dsec GM23242PA
Dyak GE14540PA
Dere GG16379PA
Dgri GH23941PA
Dmoj GI14066PA
Dvir GJ18986PA
Dwil GK20250PA
Dwil GK20229PA
Dwil GK18942PA
Dyak GE15066PA
Dwil GK21256PA
Dwil GK22277PA
Dper GL22346PA
Dpse GA28685PA
Dsim GD16070PA
Dpse GA27999PA
Dpse GA27998PA
Dwil GK18601PA
Dpse GA23994PA
Dyak GE15237PA
Dsec GM16571PA
Dsim GD22876PA
dpr3PB
Dere GG24558PA
Dana GF15308PA
Dper GL15297PA
Dpse GA13687PA
Dwil GK15095PA
Dgri GH10856PA
Dgri GH25011PA
Dpse GA27529PA
Dper GL10325PA
Dpse GA23160PA
Dper GL25339PA
Dper GL20971PA
Dpse GA27777PA
Dper GL18357PA
CG42534PC
Dsec GM10391PA
Dsim GD15191PA
Dere GG11549PA
1000
834
834
19
1000
492
959
830
987
999
0
0
0
1000
1000
180
113
1000
43
284
0
1000
420
233
130
1000
1000
967
1000
863
1000
1000
1000
1000
327
0
10
744
694
727
1000
633
467
398
139
61

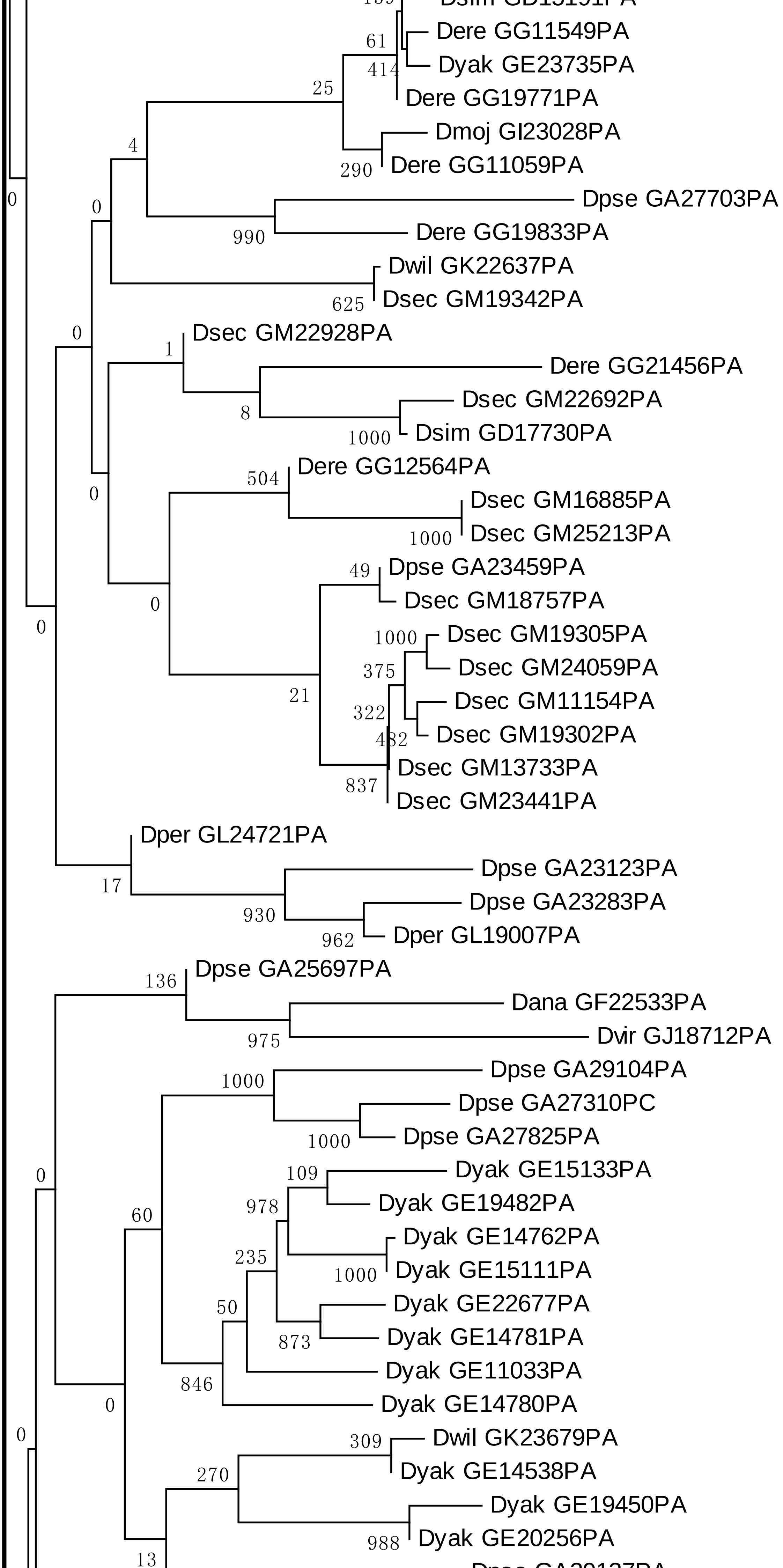

Dere GG11549PA
Dyak GE23735PA
Dere GG19771PA
Dmoj GI23028PA
Dere GG11059PA
Dpse GA27703PA
Dere GG19833PA
Dwil GK22637PA
Dsec GM19342PA
Dsec GM22928PA
Dere GG21456PA
Dsec GM22692PA
Dsim GD17730PA
Dere GG12564PA
Dsec GM16885PA
Dsec GM25213PA
Dpse GA23459PA
Dsec GM18757PA
Dsec GM19305PA
Dsec GM24059PA
Dsec GM11154PA
Dsec GM19302PA
Dsec GM13733PA
Dsec GM23441PA
Dper GL24721PA
Dpse GA23123PA
Dpse GA23283PA
Dper GL19007PA
Dpse GA25697PA
Dana GF22533PA
Dvir GJ18712PA
Dpse GA29104PA
Dpse GA27310PC
Dpse GA27825PA
Dyak GE15133PA
Dyak GE19482PA
Dyak GE14762PA
Dyak GE15111PA
Dyak GE22677PA
Dyak GE14781PA
Dyak GE11033PA
Dyak GE14780PA
Dwil GK23679PA
Dyak GE14538PA
Dyak GE19450PA
Dyak GE20256PA
61
414
25
290
4
0
990
625
0
0
1
8
1000
504
1000
0
49
1000
375
322
482
21
837
0
17
930
962
136
975
1000
1000
109
978
235
1000
873
50
846
60
0
0
309
270
988
13
0

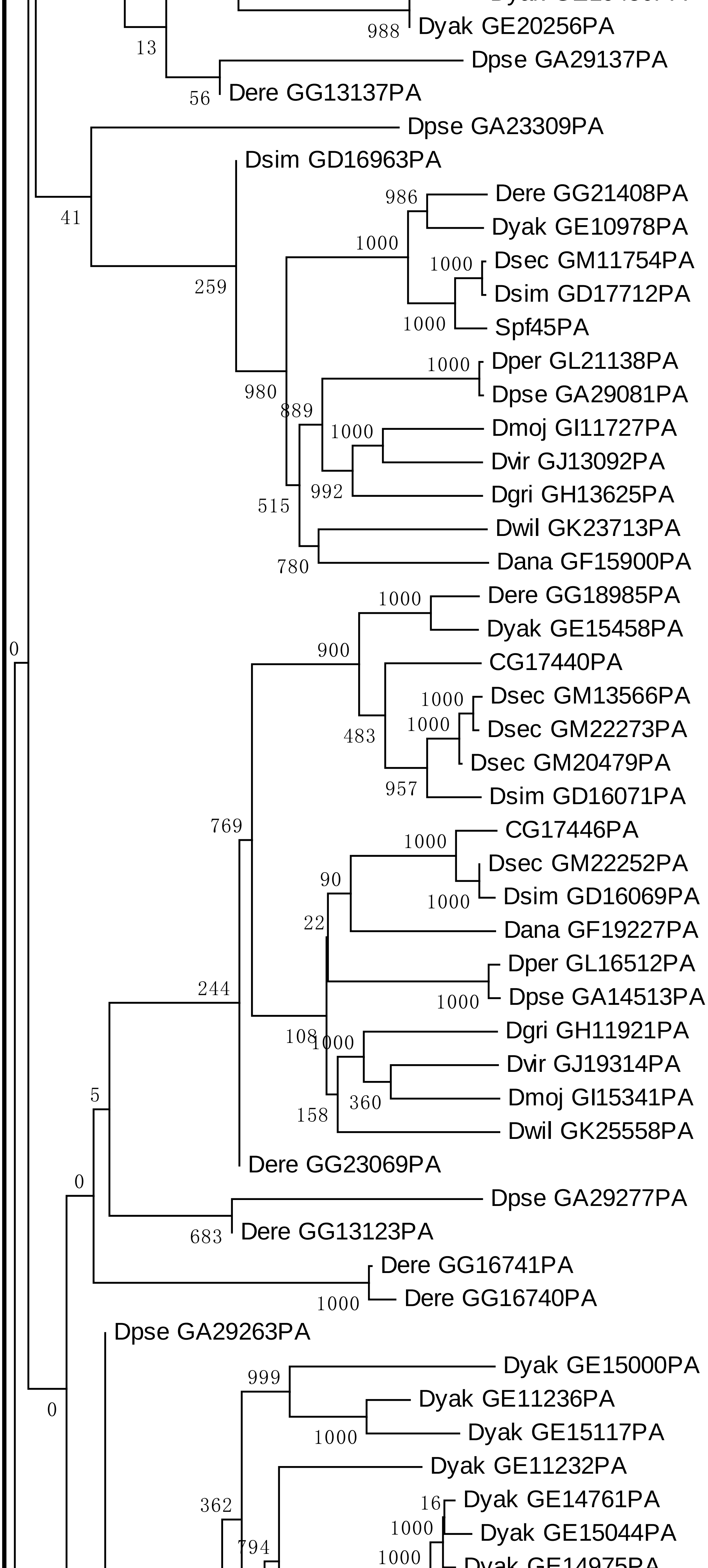
Dyak GE20256PA
Dpse GA29137PA
Dere GG13137PA
Dpse GA23309PA
Dsim GD16963PA
Dere GG21408PA
Dyak GE10978PA
Dsec GM11754PA
Dsim GD17712PA
Spf45PA
Dper GL21138PA
Dpse GA29081PA
Dmoj GI11727PA
Dvir GJ13092PA
Dgri GH13625PA
Dwil GK23713PA
Dana GF15900PA
Dere GG18985PA
Dyak GE15458PA
CG17440PA
Dsec GM13566PA
Dsec GM22273PA
Dsec GM20479PA
Dsim GD16071PA
CG17446PA
Dsec GM22252PA
Dsim GD16069PA
Dana GF19227PA
Dper GL16512PA
Dpse GA14513PA
Dgri GH11921PA
Dvir GJ19314PA
Dmoj GI15341PA
Dwil GK25558PA
Dere GG23069PA
Dpse GA29277PA
Dere GG13123PA
Dere GG16741PA
Dere GG16740PA
Dpse GA29263PA
Dyak GE15000PA
Dyak GE11236PA
Dyak GE15117PA
Dyak GE11232PA
Dyak GE14761PA
Dyak GE15044PA
Dyak GE14975PA

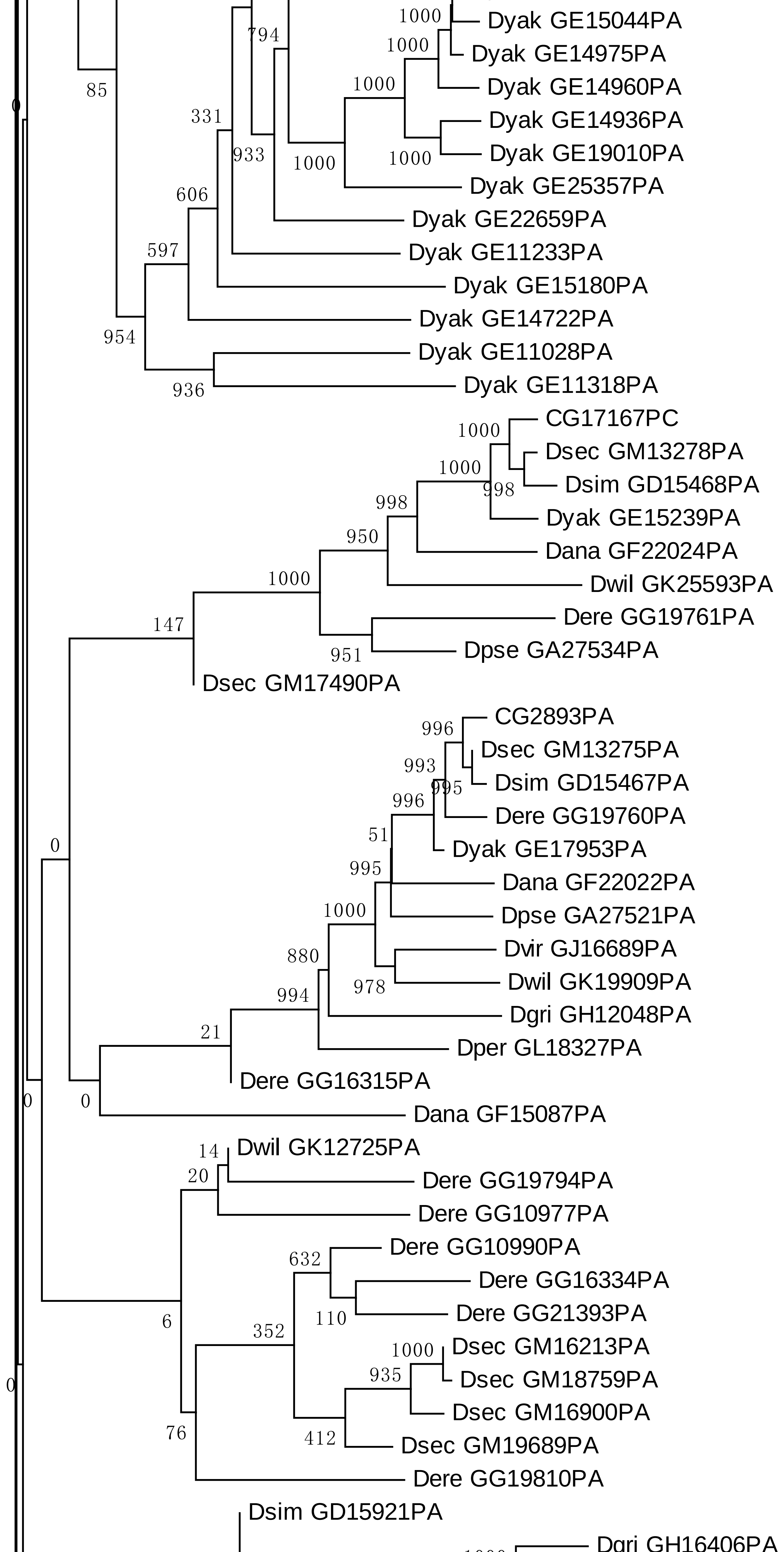
Dyak GE15044PA
Dyak GE14975PA
Dyak GE14960PA
Dyak GE14936PA
Dyak GE19010PA
Dyak GE25357PA
Dyak GE22659PA
Dyak GE11233PA
Dyak GE15180PA
Dyak GE14722PA
Dyak GE11028PA
Dyak GE11318PA
CG17167PC
Dsec GM13278PA
Dsim GD15468PA
Dyak GE15239PA
Dana GF22024PA
Dwil GK25593PA
Dere GG19761PA
Dpse GA27534PA
Dsec GM17490PA
CG2893PA
Dsec GM13275PA
Dsim GD15467PA
Dere GG19760PA
Dyak GE17953PA
Dana GF22022PA
Dpse GA27521PA
Dvir GJ16689PA
Dwil GK19909PA
Dgri GH12048PA
Dper GL18327PA
Dere GG16315PA
Dana GF15087PA
Dwil GK12725PA
Dere GG19794PA
Dere GG10977PA
Dere GG10990PA
Dere GG16334PA
Dere GG21393PA
Dsec GM16213PA
Dsec GM18759PA
Dsec GM16900PA
Dsec GM19689PA
Dere GG19810PA
Dsim GD15921PA
Dgri GH16406PA

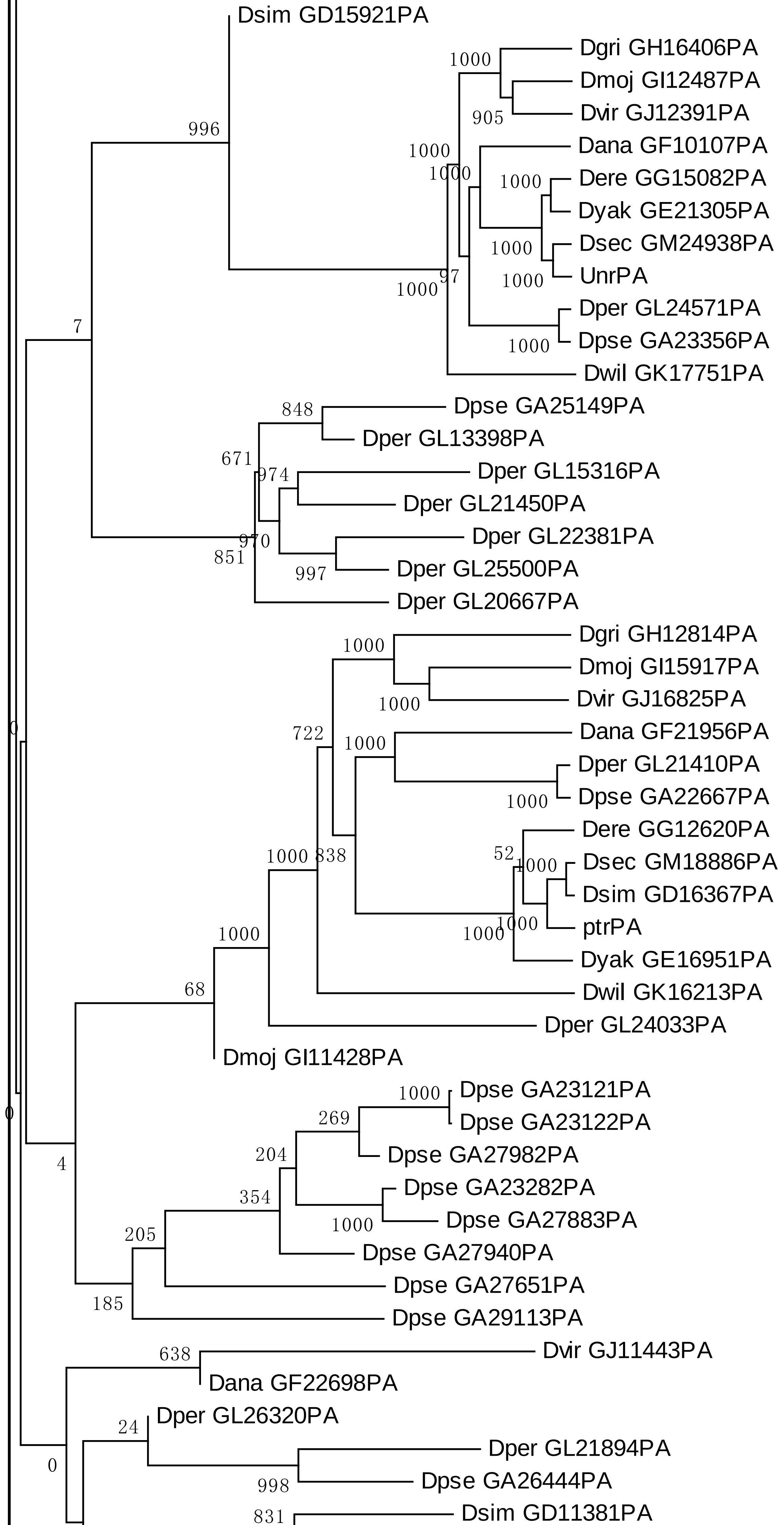

Dsim GD15921PA
996
1000
Dgri GH16406PA
Dmoj GI12487PA
905
Dvir GJ12391PA
1000
Dana GF10107PA
1000
1000
Dere GG15082PA
Dyak GE21305PA
1000
Dsec GM24938PA
UnrPA
1000
97
1000
Dper GL24571PA
Dpse GA23356PA
1000
Dwil GK17751PA
7
848
Dpse GA25149PA
Dper GL13398PA
671
974
Dper GL15316PA
Dper GL21450PA
970
Dper GL22381PA
851
997
Dper GL25500PA
Dper GL20667PA
1000
Dgri GH12814PA
Dmoj GI15917PA
1000
Dvir GJ16825PA
0
722
1000
Dana GF21956PA
Dper GL21410PA
1000
Dpse GA22667PA
Dere GG12620PA
1000
838
52
1000
Dsec GM18886PA
Dsim GD16367PA
1000
1000
ptrPA
Dyak GE16951PA
1000
Dwil GK16213PA
68
Dper GL24033PA
Dmoj GI11428PA
1000
Dpse GA23121PA
269
Dpse GA23122PA
0
Dpse GA27982PA
204
Dpse GA23282PA
354
1000
Dpse GA27883PA
4
205
Dpse GA27940PA
Dpse GA27651PA
185
Dpse GA29113PA
638
Dvir GJ11443PA
Dana GF22698PA
24
Dper GL26320PA
Dper GL21894PA
0
998
Dpse GA26444PA
831
Dsim GD11381PA

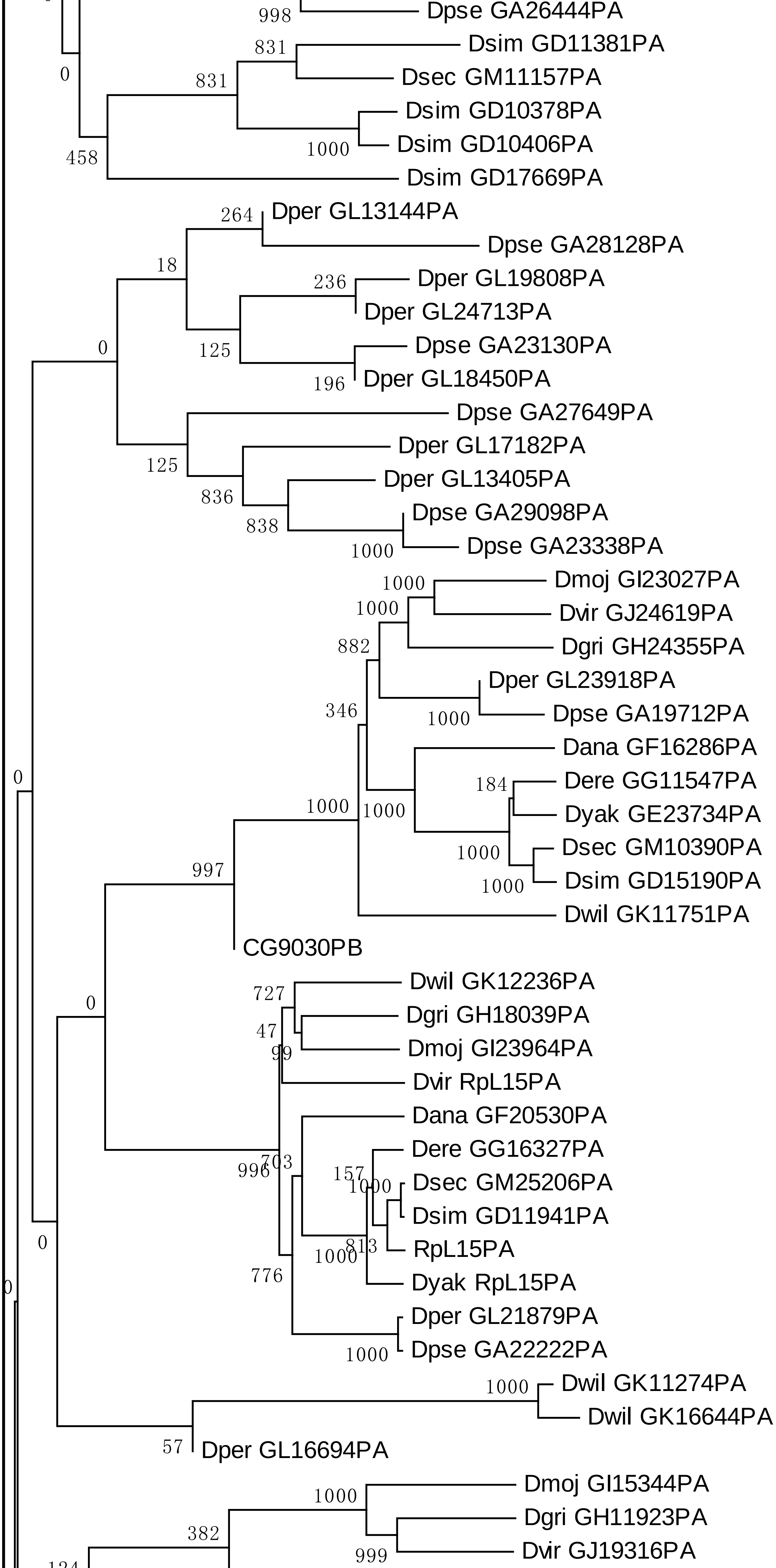
998
Dpse GA26444PA
831
Dsim GD11381PA
0
831
Dsec GM11157PA
Dsim GD10378PA
1000
Dsim GD10406PA
458
Dsim GD17669PA
264
Dper GL13144PA
Dpse GA28128PA
18
236
Dper GL19808PA
Dper GL24713PA
0
125
Dpse GA23130PA
196
Dper GL18450PA
Dpse GA27649PA
Dper GL17182PA
125
Dper GL13405PA
836
Dpse GA29098PA
838
1000
Dpse GA23338PA
1000
Dmoj GI23027PA
1000
Dvir GJ24619PA
882
Dgri GH24355PA
Dper GL23918PA
346
1000
Dpse GA19712PA
Dana GF16286PA
0
184
Dere GG11547PA
1000
1000
Dyak GE23734PA
Dsec GM10390PA
1000
997
1000
Dsim GD15190PA
Dwil GK11751PA
CG9030PB
727
Dwil GK12236PA
0
47
Dgri GH18039PA
99
Dmoj GI23964PA
Dvir RpL15PA
Dana GF20530PA
Dere GG16327PA
996
703
157
1000
Dsec GM25206PA
Dsim GD11941PA
0
1000
813
RpL15PA
776
Dyak RpL15PA
0
Dper GL21879PA
1000
Dpse GA22222PA
1000
Dwil GK11274PA
Dwil GK16644PA
57
Dper GL16694PA
1000
Dmoj GI15344PA
382
Dgri GH11923PA
999
Dvir GJ19316PA

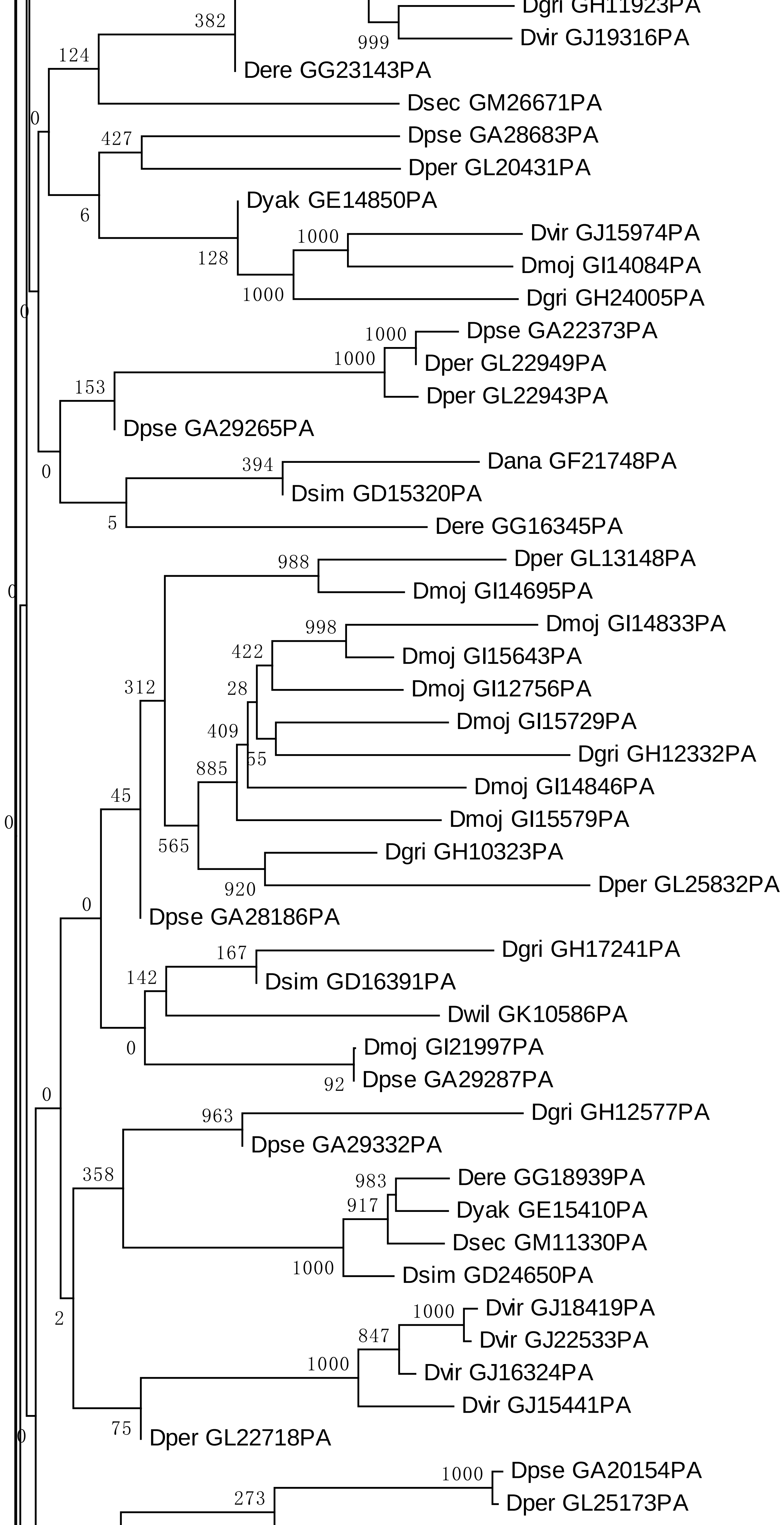

Dgri GH11923PA
382
999
Dvir GJ19316PA
124
Dere GG23143PA
0
Dsec GM26671PA
427
Dpse GA28683PA
Dper GL20431PA
Dyak GE14850PA
6
1000
Dvir GJ15974PA
128
Dmoj GI14084PA
1000
Dgri GH24005PA
0
1000
Dpse GA22373PA
1000
Dper GL22949PA
153
Dper GL22943PA
Dpse GA29265PA
394
Dana GF21748PA
0
Dsim GD15320PA
5
Dere GG16345PA
988
Dper GL13148PA
0
Dmoj GI14695PA
998
Dmoj GI14833PA
422
Dmoj GI15643PA
312
28
Dmoj GI12756PA
Dmoj GI15729PA
409
Dgri GH12332PA
55
885
Dmoj GI14846PA
45
0
Dmoj GI15579PA
565
Dgri GH10323PA
920
Dper GL25832PA
0
Dpse GA28186PA
167
Dgri GH17241PA
142
Dsim GD16391PA
Dwil GK10586PA
0
Dmoj GI21997PA
92
Dpse GA29287PA
0
963
Dgri GH12577PA
Dpse GA29332PA
358
983
Dere GG18939PA
917
Dyak GE15410PA
Dsec GM11330PA
1000
Dsim GD24650PA
2
1000
Dvir GJ18419PA
Dvir GJ22533PA
847
1000
Dvir GJ16324PA
Dvir GJ15441PA
75
0
Dper GL22718PA
1000
Dpse GA20154PA
273
Dper GL25173PA

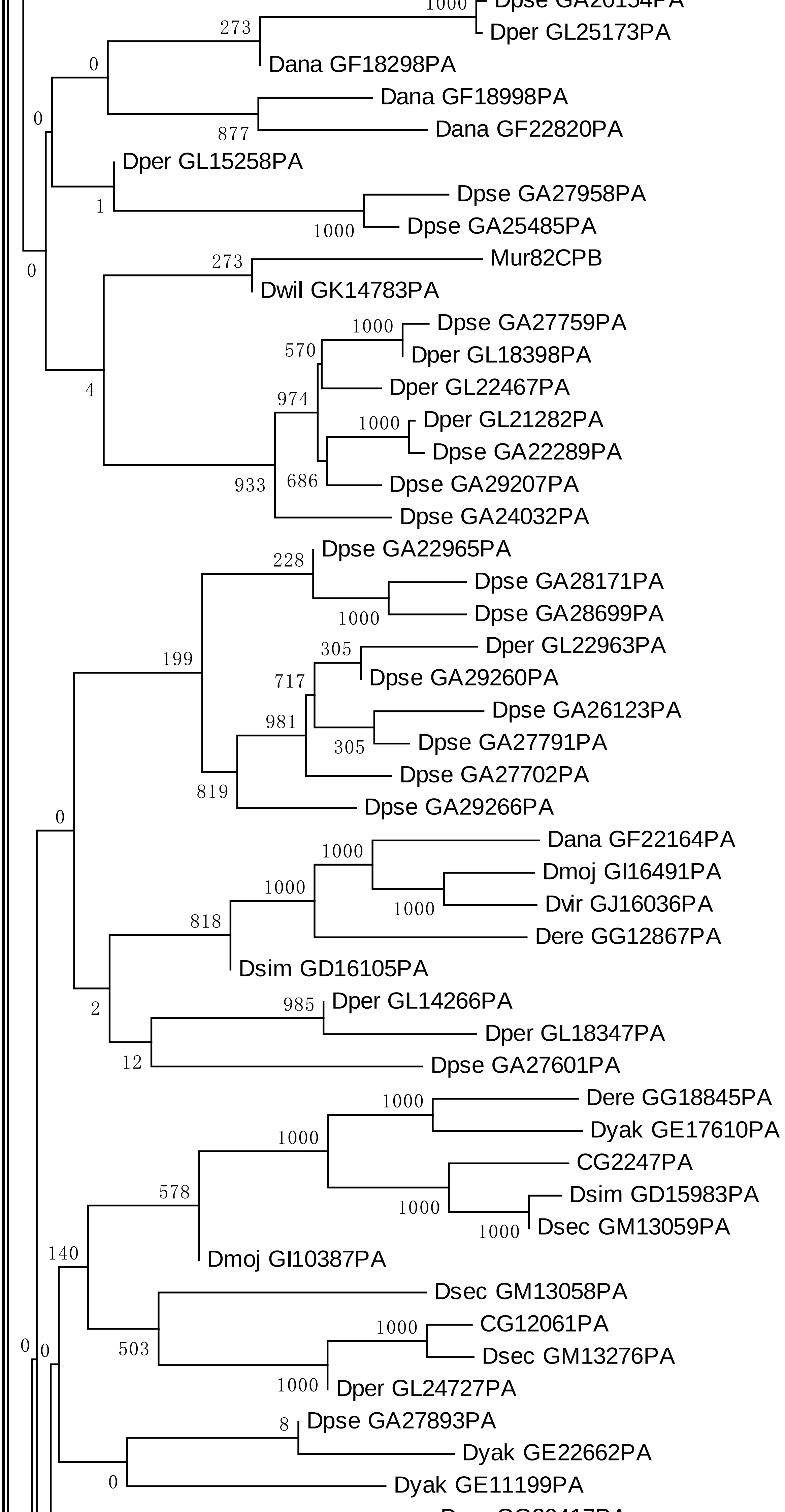
Dper GL25173PA
273
0
Dana GF18298PA
Dana GF18998PA
0
877
Dana GF22820PA
Dper GL15258PA
Dpse GA27958PA
1
1000
Dpse GA25485PA
273
Mur82CPB
0
Dwil GK14783PA
1000
Dpse GA27759PA
570
Dper GL18398PA
Dper GL22467PA
4
974
1000
Dper GL21282PA
Dpse GA22289PA
933
686
Dpse GA29207PA
Dpse GA24032PA
228
Dpse GA22965PA
Dpse GA28171PA
1000
Dpse GA28699PA
199
305
Dper GL22963PA
717
Dpse GA29260PA
Dpse GA26123PA
981
305
Dpse GA27791PA
Dpse GA27702PA
819
Dpse GA29266PA
0
Dana GF22164PA
1000
Dmoj GI16491PA
1000
1000
Dvir GJ16036PA
818
Dere GG12867PA
Dsim GD16105PA
985
Dper GL14266PA
2
Dper GL18347PA
12
Dpse GA27601PA
Dere GG18845PA
1000
Dyak GE17610PA
1000
CG2247PA
578
Dsim GD15983PA
1000
1000
Dsec GM13059PA
140
Dmoj GI10387PA
Dsec GM13058PA
1000
CG12061PA
503
Dsec GM13276PA
0
0
1000
Dper GL24727PA
8
Dpse GA27893PA
Dyak GE22662PA
0
Dyak GE11199PA

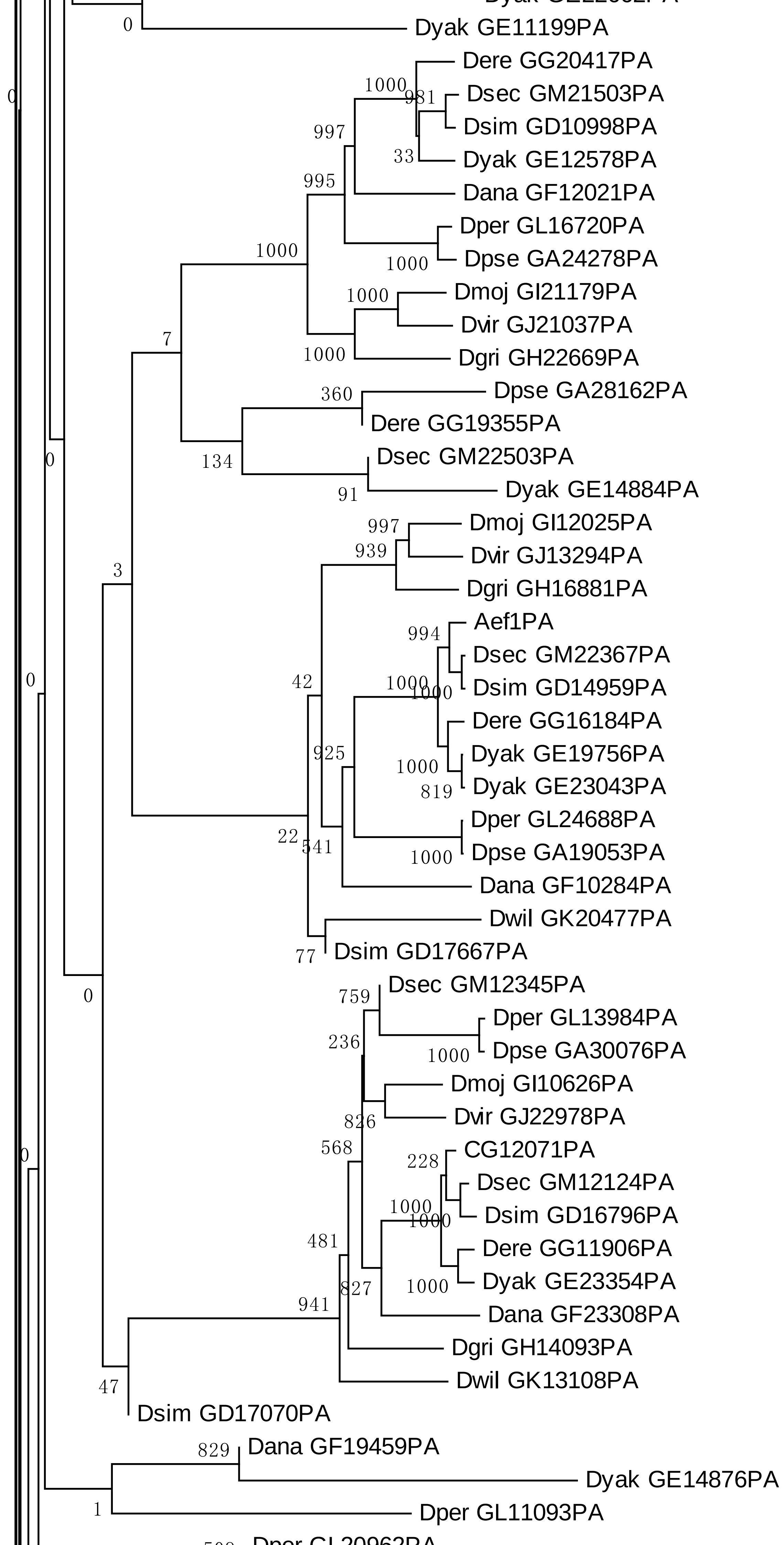
Dyak GE11199PA
Dere GG20417PA
Dsec GM21503PA
Dsim GD10998PA
Dyak GE12578PA
Dana GF12021PA
Dper GL16720PA
Dpse GA24278PA
Dmoj GI21179PA
Dvir GJ21037PA
Dgri GH22669PA
Dpse GA28162PA
Dere GG19355PA
Dsec GM22503PA
Dyak GE14884PA
Dmoj GI12025PA
Dvir GJ13294PA
Dgri GH16881PA
Aef1PA
Dsec GM22367PA
Dsim GD14959PA
Dere GG16184PA
Dyak GE19756PA
Dyak GE23043PA
Dper GL24688PA
Dpse GA19053PA
Dana GF10284PA
Dwil GK20477PA
Dsim GD17667PA
Dsec GM12345PA
Dper GL13984PA
Dpse GA30076PA
Dmoj GI10626PA
Dvir GJ22978PA
CG12071PA
Dsec GM12124PA
Dsim GD16796PA
Dere GG11906PA
Dyak GE23354PA
Dana GF23308PA
Dgri GH14093PA
Dwil GK13108PA
Dsim GD17070PA
Dana GF19459PA
Dyak GE14876PA
Dper GL11093PA

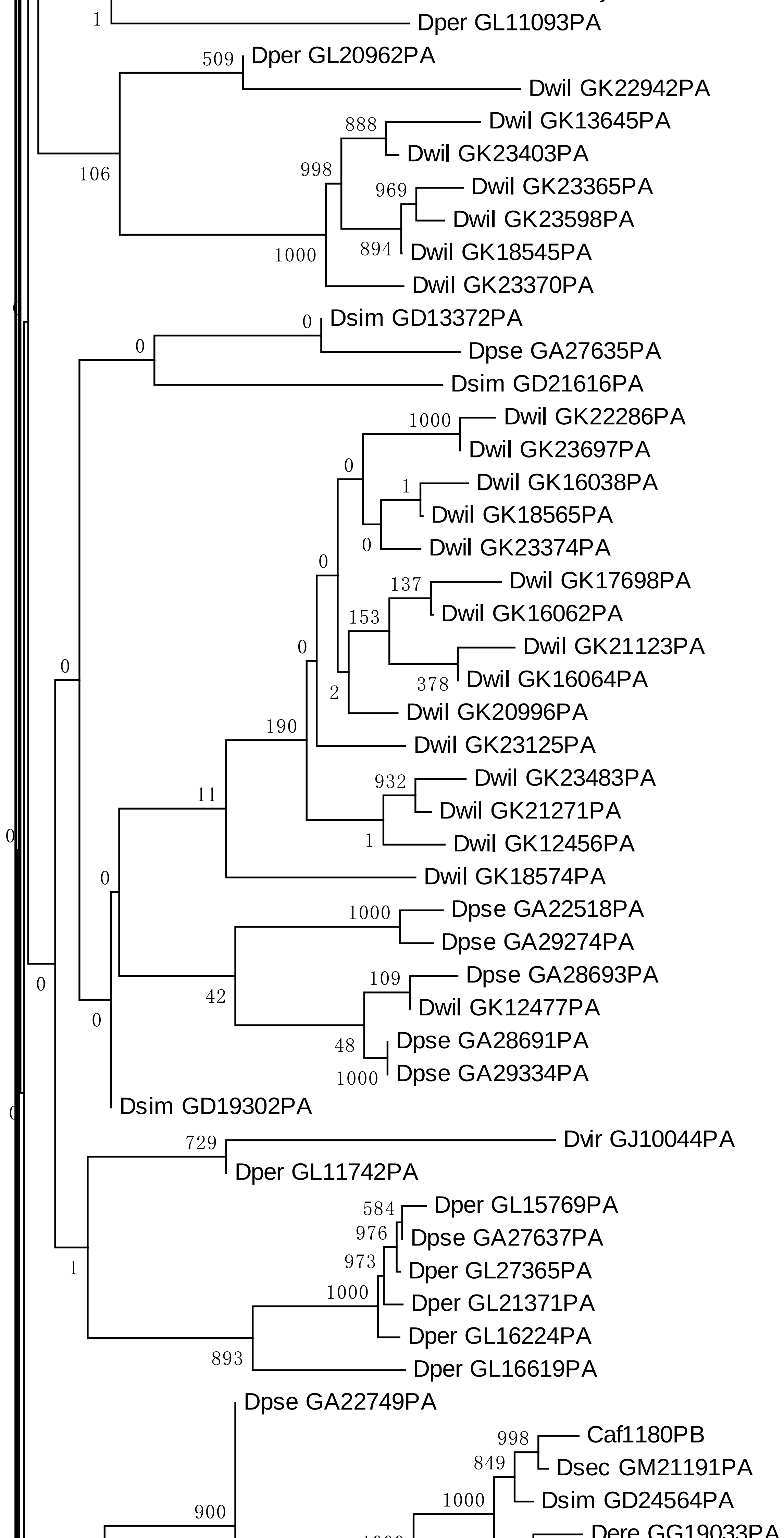
Dper GL11093PA
Dper GL20962PA
Dwil GK22942PA
Dwil GK13645PA
Dwil GK23403PA
Dwil GK23365PA
Dwil GK23598PA
Dwil GK18545PA
Dwil GK23370PA
Dsim GD13372PA
Dpse GA27635PA
Dsim GD21616PA
Dwil GK22286PA
Dwil GK23697PA
Dwil GK16038PA
Dwil GK18565PA
Dwil GK23374PA
Dwil GK17698PA
Dwil GK16062PA
Dwil GK21123PA
Dwil GK16064PA
Dwil GK20996PA
Dwil GK23125PA
Dwil GK23483PA
Dwil GK21271PA
Dwil GK12456PA
Dwil GK18574PA
Dpse GA22518PA
Dpse GA29274PA
Dpse GA28693PA
Dwil GK12477PA
Dpse GA28691PA
Dpse GA29334PA
Dsim GD19302PA
Dvir GJ10044PA
Dper GL11742PA
Dper GL15769PA
Dpse GA27637PA
Dper GL27365PA
Dper GL21371PA
Dper GL16224PA
Dper GL16619PA
Dpse GA22749PA
Caf1180PB
Dsec GM21191PA
Dsim GD24564PA

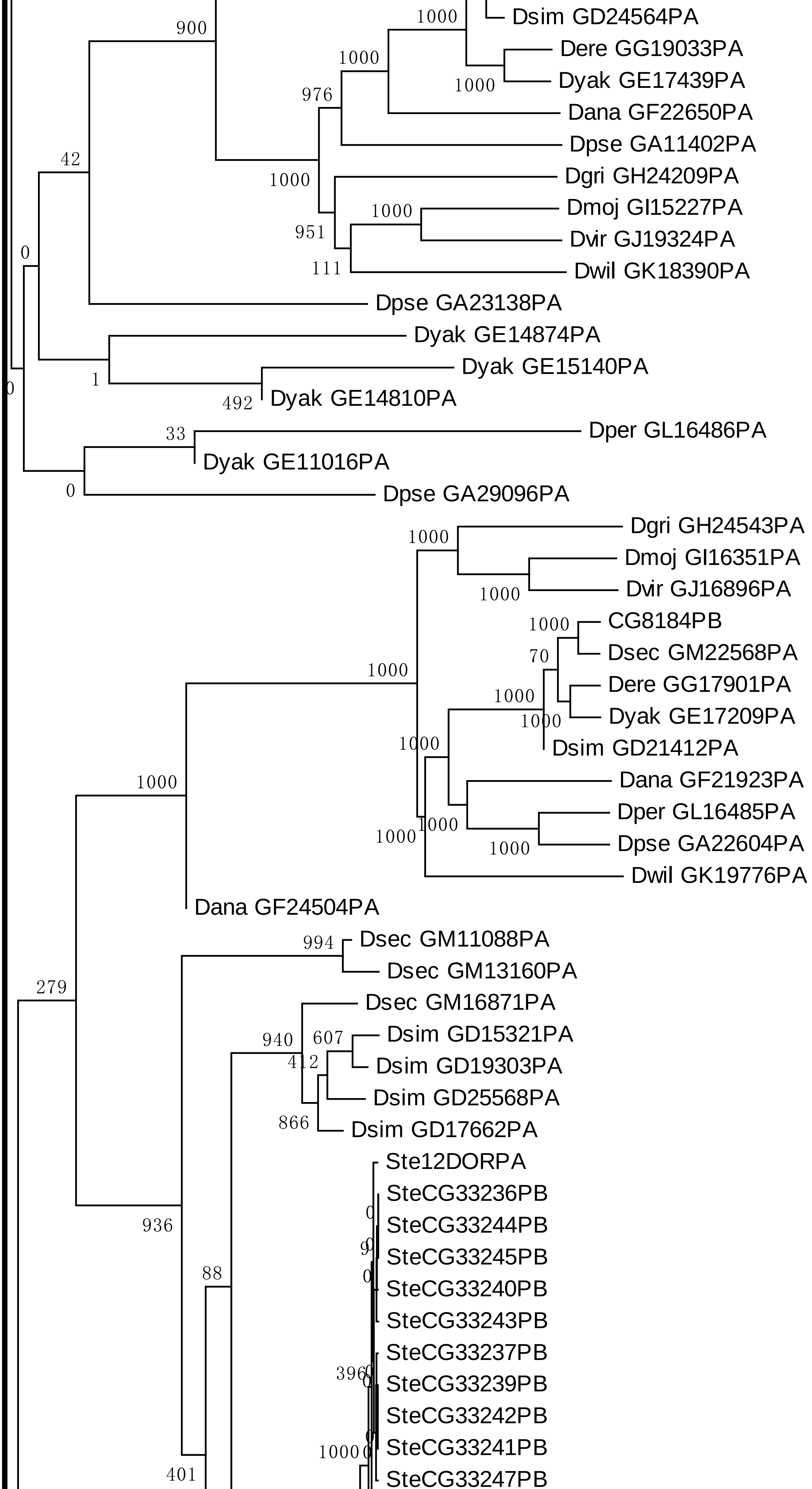
Dsim GD24564PA
Dere GG19033PA
Dyak GE17439PA
Dana GF22650PA
Dpse GA11402PA
Dgri GH24209PA
Dmoj GI15227PA
Dvir GJ19324PA
Dwil GK18390PA
Dpse GA23138PA
Dyak GE14874PA
Dyak GE15140PA
Dyak GE14810PA
Dper GL16486PA
Dyak GE11016PA
Dpse GA29096PA
Dgri GH24543PA
Dmoj GI16351PA
Dvir GJ16896PA
CG8184PB
Dsec GM22568PA
Dere GG17901PA
Dyak GE17209PA
Dsim GD21412PA
Dana GF21923PA
Dper GL16485PA
Dpse GA22604PA
Dwil GK19776PA
Dana GF24504PA
Dsec GM11088PA
Dsec GM13160PA
Dsec GM16871PA
Dsim GD15321PA
Dsim GD19303PA
Dsim GD25568PA
Dsim GD17662PA
Ste12DORPA
SteCG33236PB
SteCG33244PB
SteCG33245PB
SteCG33240PB
SteCG33243PB
SteCG33237PB
SteCG33239PB
SteCG33242PB
SteCG33241PB
SteCG33247PB
900
1000
1000
1000
976
1000
1000
951
111
42
0
1
492
33
0
1000
1000
1000
70
1000
1000
1000
1000
1000
1000
1000
1000
994
279
940
607
412
866
936
88
396
1000
401

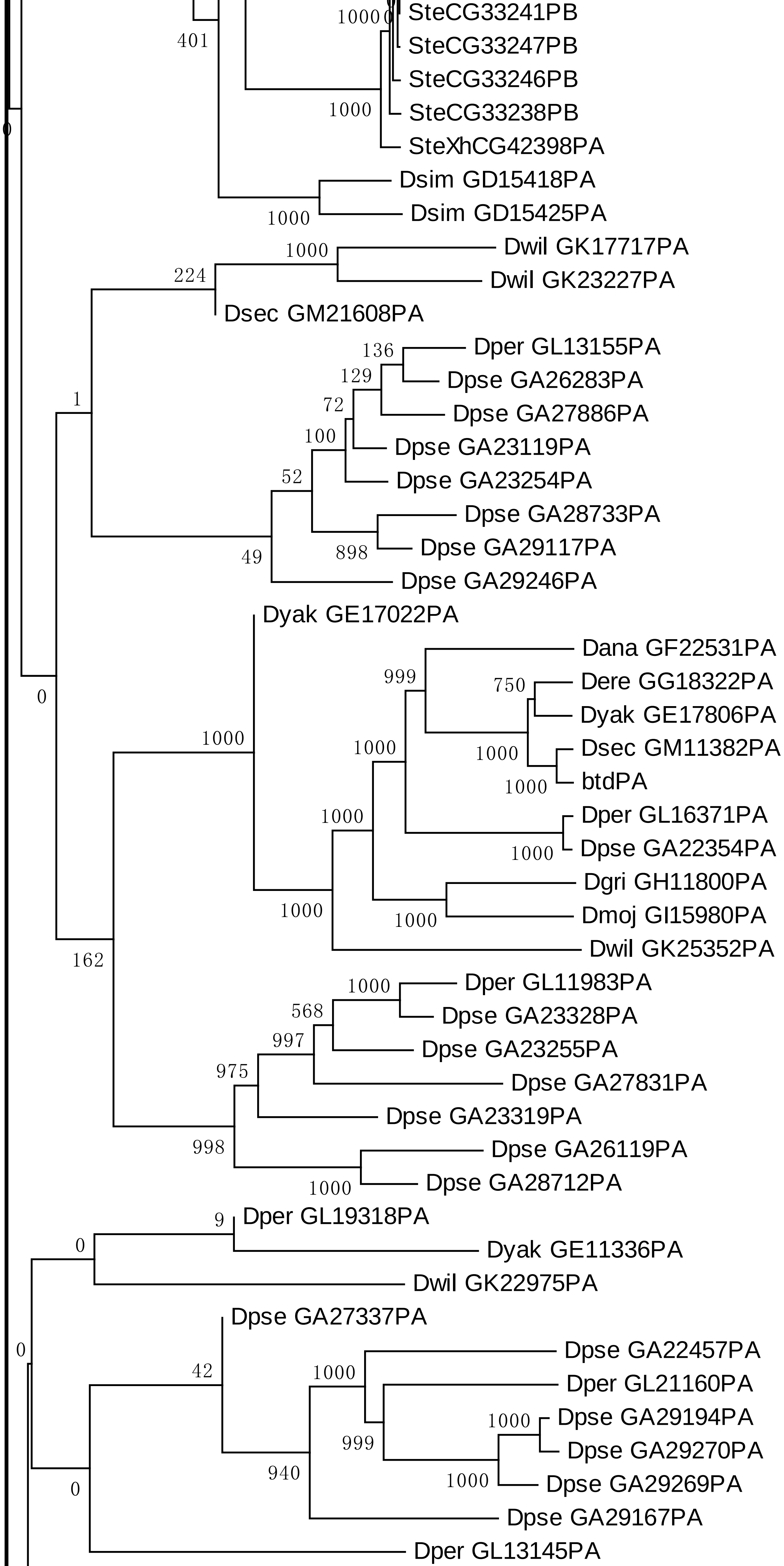

SteCG33241PB
1000
401
SteCG33247PB
SteCG33246PB
1000
SteCG33238PB
0
SteXhCG42398PA
Dsim GD15418PA
1000
Dsim GD15425PA
1000
Dwil GK17717PA
224
Dwil GK23227PA
Dsec GM21608PA
136
Dper GL13155PA
129
Dpse GA26283PA
1
72
Dpse GA27886PA
100
Dpse GA23119PA
52
Dpse GA23254PA
Dpse GA28733PA
898
Dpse GA29117PA
49
Dpse GA29246PA
Dyak GE17022PA
Dana GF22531PA
999
750
Dere GG18322PA
0
Dyak GE17806PA
1000
1000
1000
Dsec GM11382PA
1000
btdPA
1000
Dper GL16371PA
1000
Dpse GA22354PA
Dgri GH11800PA
1000
1000
Dmoj GI15980PA
162
Dwil GK25352PA
1000
Dper GL11983PA
568
Dpse GA23328PA
997
Dpse GA23255PA
975
Dpse GA27831PA
Dpse GA23319PA
998
Dpse GA26119PA
1000
Dpse GA28712PA
9
Dper GL19318PA
0
Dyak GE11336PA
Dwil GK22975PA
Dpse GA27337PA
0
Dpse GA22457PA
42
1000
Dper GL21160PA
1000
Dpse GA29194PA
999
Dpse GA29270PA
940
1000
Dpse GA29269PA
0
Dpse GA29167PA
Dper GL13145PA

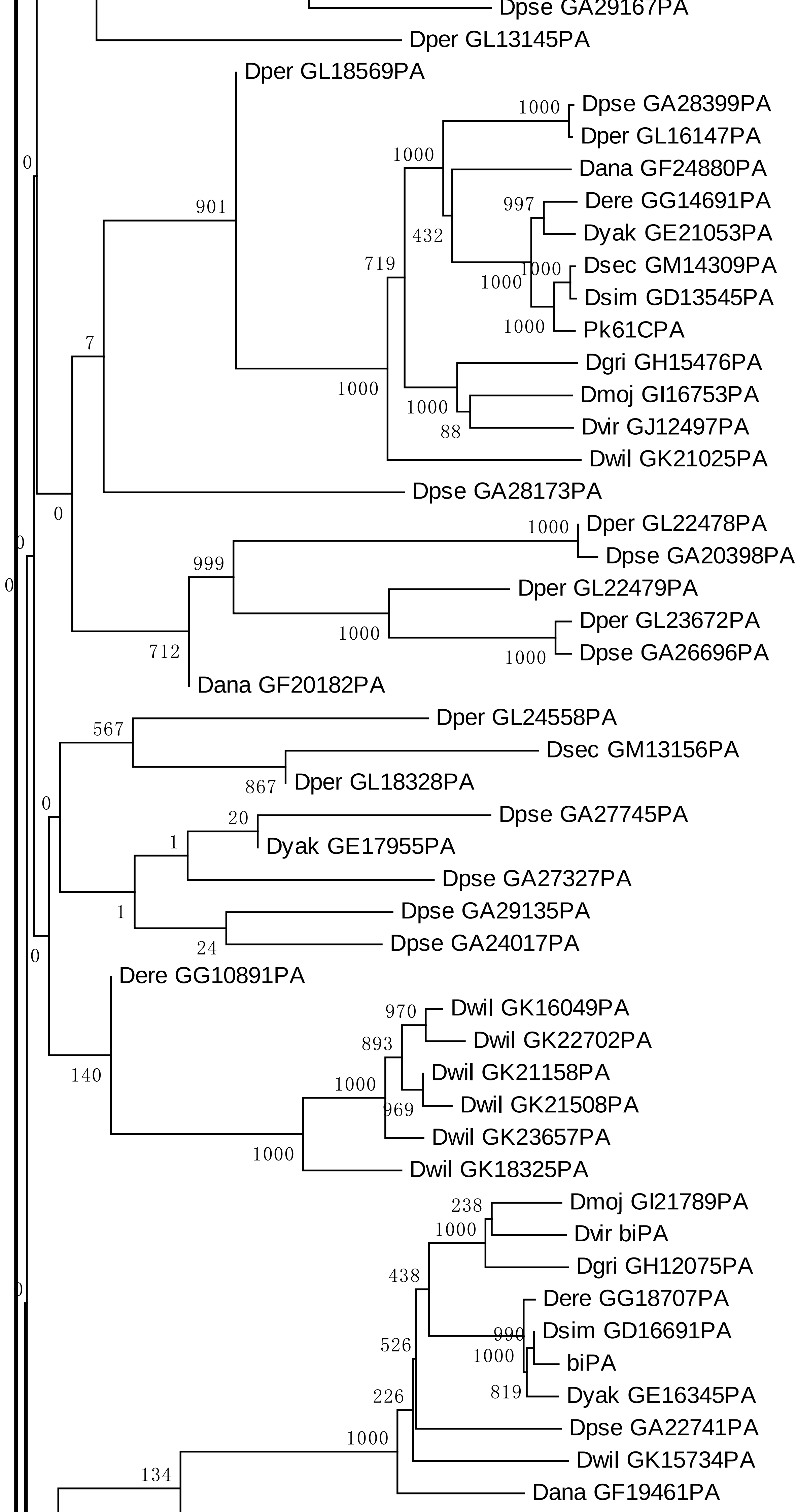

Dpse GA29167PA
Dper GL13145PA
Dper GL18569PA
Dpse GA28399PA
Dper GL16147PA
Dana GF24880PA
Dere GG14691PA
Dyak GE21053PA
Dsec GM14309PA
Dsim GD13545PA
Pk61CPA
Dgri GH15476PA
Dmoj GI16753PA
Dvir GJ12497PA
Dwil GK21025PA
Dpse GA28173PA
Dper GL22478PA
Dpse GA20398PA
Dper GL22479PA
Dper GL23672PA
Dpse GA26696PA
Dana GF20182PA
Dper GL24558PA
Dsec GM13156PA
Dper GL18328PA
Dpse GA27745PA
Dyak GE17955PA
Dpse GA27327PA
Dpse GA29135PA
Dpse GA24017PA
Dere GG10891PA
Dwil GK16049PA
Dwil GK22702PA
Dwil GK21158PA
Dwil GK21508PA
Dwil GK23657PA
Dwil GK18325PA
Dmoj GI21789PA
Dvir biPA
Dgri GH12075PA
Dere GG18707PA
Dsim GD16691PA
biPA
Dyak GE16345PA
Dpse GA22741PA
Dwil GK15734PA
Dana GF19461PA

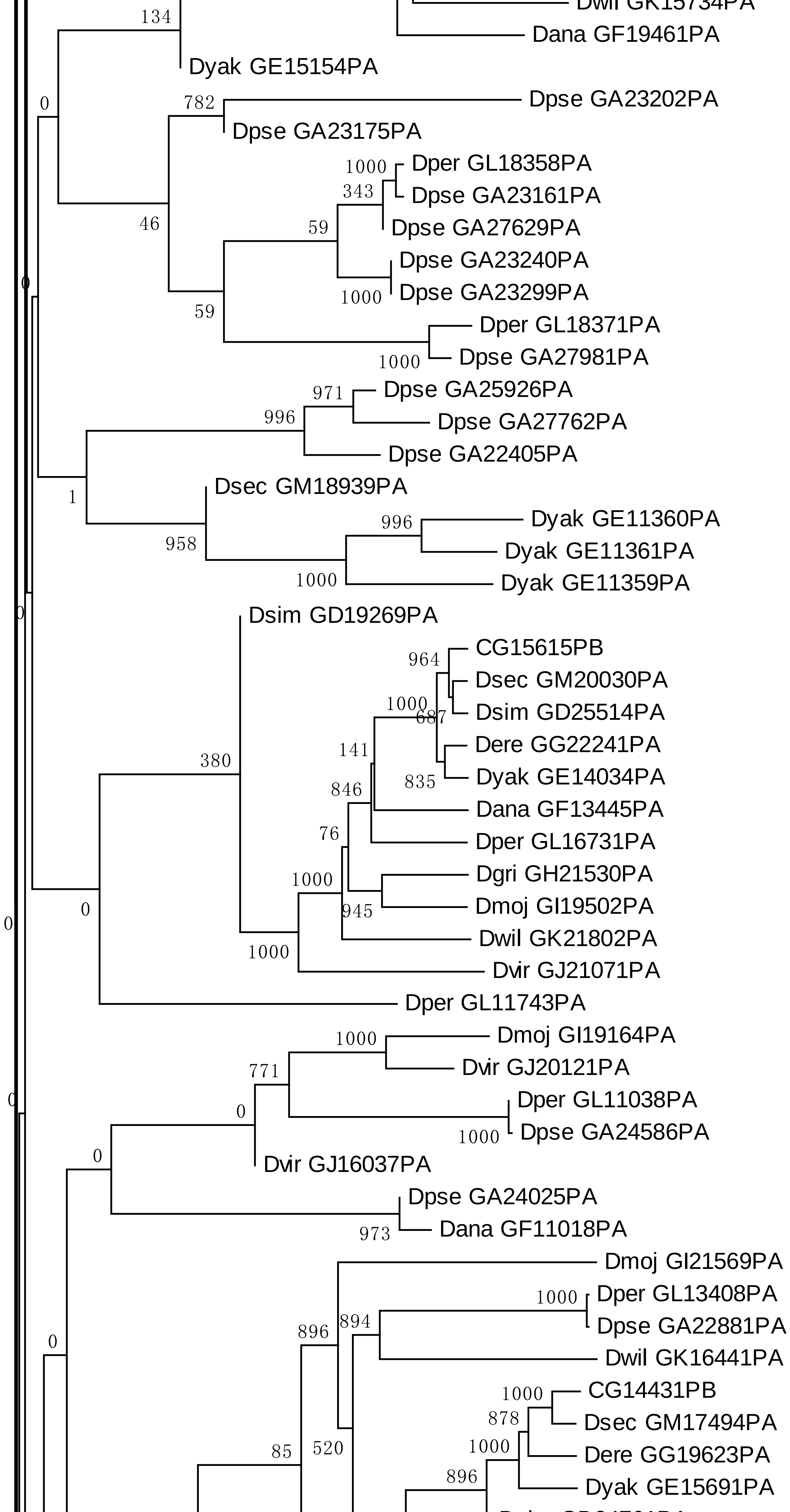
Dana GF19461PA
Dyak GE15154PA
Dpse GA23202PA
Dpse GA23175PA
Dper GL18358PA
Dpse GA23161PA
Dpse GA27629PA
Dpse GA23240PA
Dpse GA23299PA
Dper GL18371PA
Dpse GA27981PA
Dpse GA25926PA
Dpse GA27762PA
Dpse GA22405PA
Dsec GM18939PA
Dyak GE11360PA
Dyak GE11361PA
Dyak GE11359PA
Dsim GD19269PA
CG15615PB
Dsec GM20030PA
Dsim GD25514PA
Dere GG22241PA
Dyak GE14034PA
Dana GF13445PA
Dper GL16731PA
Dgri GH21530PA
Dmoj GI19502PA
Dwil GK21802PA
Dvir GJ21071PA
Dper GL11743PA
Dmoj GI19164PA
Dvir GJ20121PA
Dper GL11038PA
Dpse GA24586PA
Dvir GJ16037PA
Dpse GA24025PA
Dana GF11018PA
Dmoj GI21569PA
Dper GL13408PA
Dpse GA22881PA
Dwil GK16441PA
CG14431PB
Dsec GM17494PA
Dere GG19623PA
Dyak GE15691PA

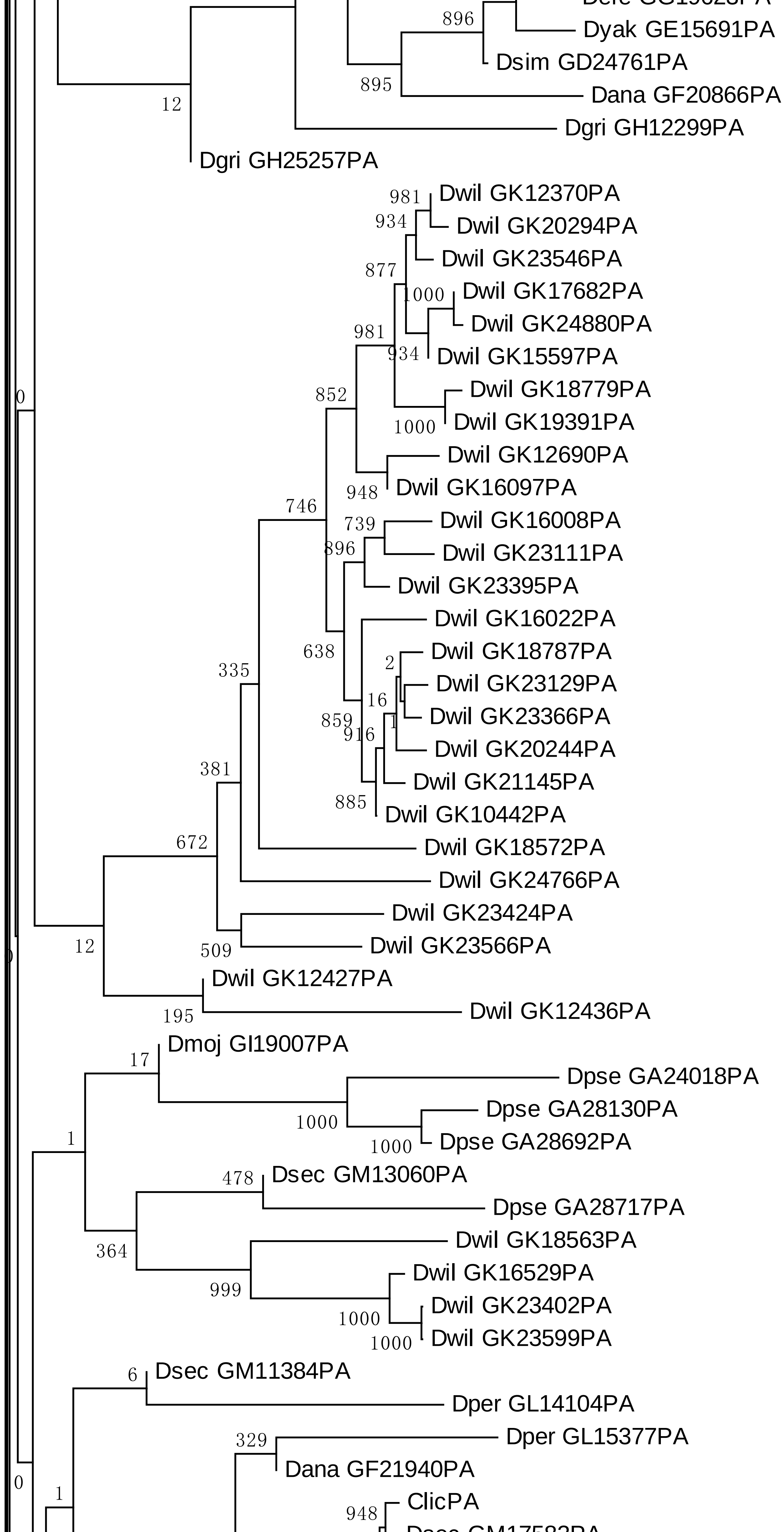
Dyak GE15691PA
Dsim GD24761PA
Dana GF20866PA
Dgri GH12299PA
Dgri GH25257PA
Dwil GK12370PA
Dwil GK20294PA
Dwil GK23546PA
Dwil GK17682PA
Dwil GK24880PA
Dwil GK15597PA
Dwil GK18779PA
Dwil GK19391PA
Dwil GK12690PA
Dwil GK16097PA
Dwil GK16008PA
Dwil GK23111PA
Dwil GK23395PA
Dwil GK16022PA
Dwil GK18787PA
Dwil GK23129PA
Dwil GK23366PA
Dwil GK20244PA
Dwil GK21145PA
Dwil GK10442PA
Dwil GK18572PA
Dwil GK24766PA
Dwil GK23424PA
Dwil GK23566PA
Dwil GK12427PA
Dwil GK12436PA
Dmoj GI19007PA
Dpse GA24018PA
Dpse GA28130PA
Dpse GA28692PA
Dsec GM13060PA
Dpse GA28717PA
Dwil GK18563PA
Dwil GK16529PA
Dwil GK23402PA
Dwil GK23599PA
Dsec GM11384PA
Dper GL14104PA
Dper GL15377PA
Dana GF21940PA
ClicPA

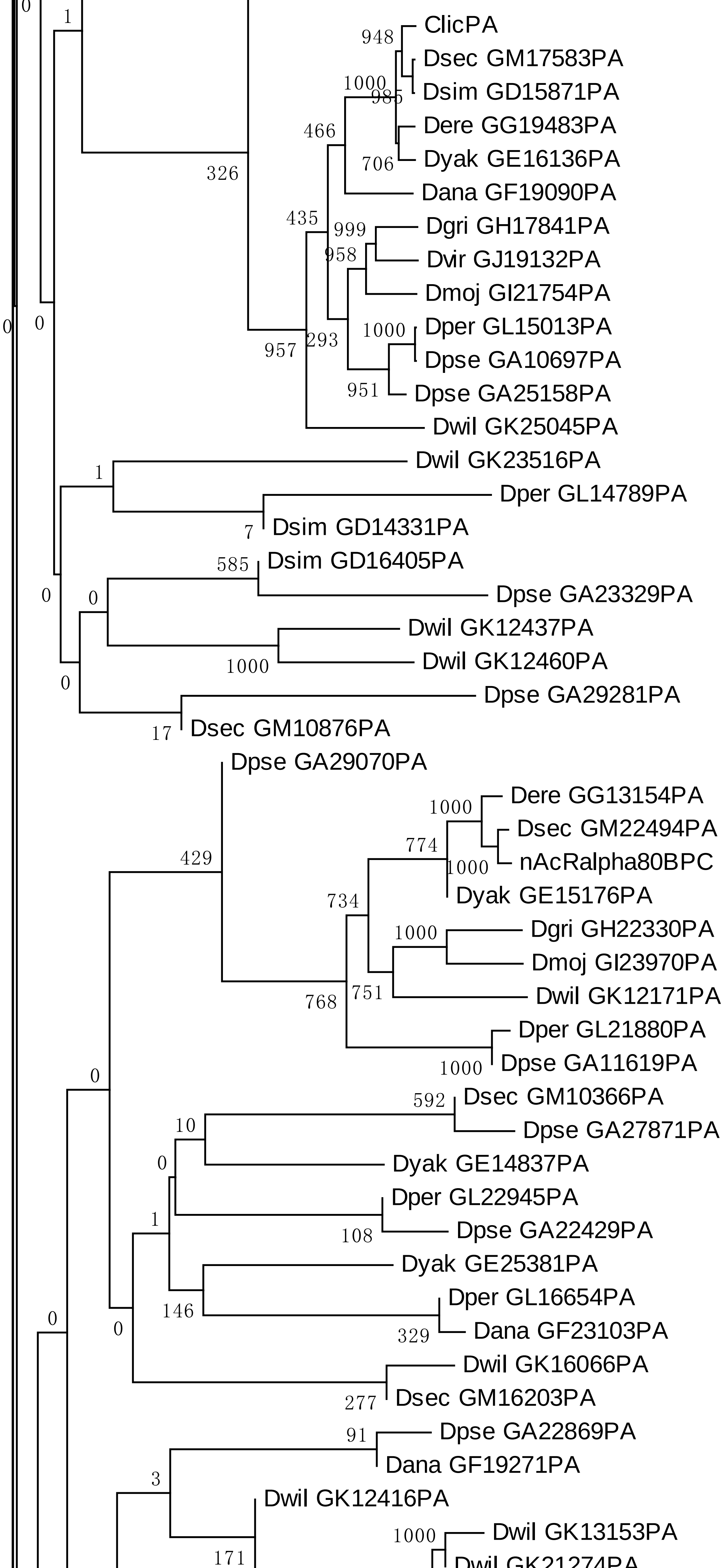
ClicPA
Dsec GM17583PA
Dsim GD15871PA
Dere GG19483PA
Dyak GE16136PA
Dana GF19090PA
Dgri GH17841PA
Dvir GJ19132PA
Dmoj GI21754PA
Dper GL15013PA
Dpse GA10697PA
Dpse GA25158PA
Dwil GK25045PA
Dwil GK23516PA
Dper GL14789PA
Dsim GD14331PA
Dsim GD16405PA
Dpse GA23329PA
Dwil GK12437PA
Dwil GK12460PA
Dpse GA29281PA
Dsec GM10876PA
Dpse GA29070PA
Dere GG13154PA
Dsec GM22494PA
nAcRalpha80BPC
Dyak GE15176PA
Dgri GH22330PA
Dmoj GI23970PA
Dwil GK12171PA
Dper GL21880PA
Dpse GA11619PA
Dsec GM10366PA
Dpse GA27871PA
Dyak GE14837PA
Dper GL22945PA
Dpse GA22429PA
Dyak GE25381PA
Dper GL16654PA
Dana GF23103PA
Dwil GK16066PA
Dsec GM16203PA
Dpse GA22869PA
Dana GF19271PA
Dwil GK12416PA
Dwil GK13153PA

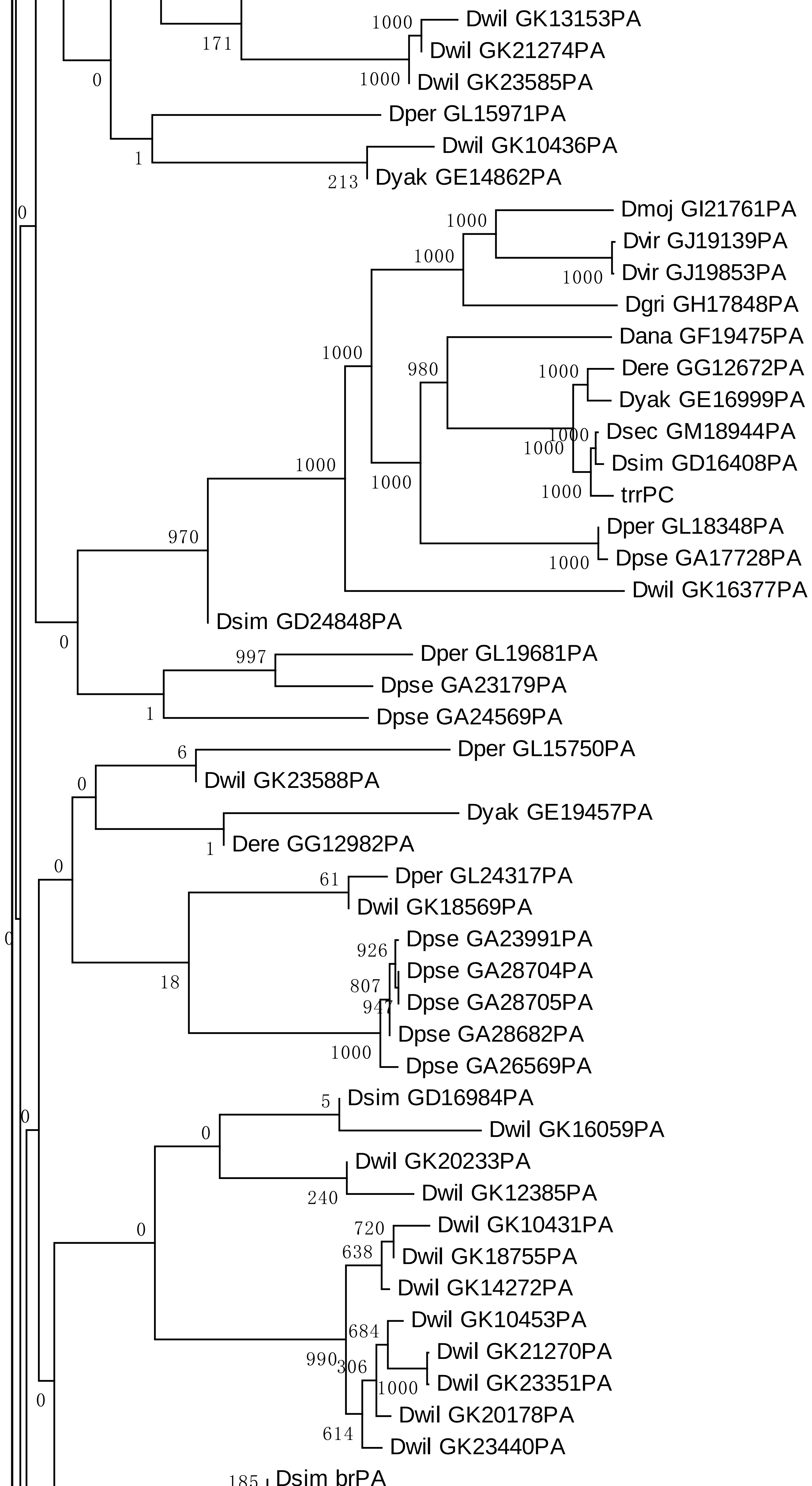
1000
Dwil GK13153PA
171
Dwil GK21274PA
0
1000
Dwil GK23585PA
Dper GL15971PA
Dwil GK10436PA
1
213
Dyak GE14862PA
0
Dmoj GI21761PA
1000
Dvir GJ19139PA
1000
Dvir GJ19853PA
1000
Dgri GH17848PA
Dana GF19475PA
1000
980
1000
Dere GG12672PA
Dyak GE16999PA
1000
Dsec GM18944PA
1000
Dsim GD16408PA
1000
1000
1000
trrPC
970
Dper GL18348PA
1000
Dpse GA17728PA
Dwil GK16377PA
Dsim GD24848PA
0
997
Dper GL19681PA
Dpse GA23179PA
1
Dpse GA24569PA
6
Dper GL15750PA
0
Dwil GK23588PA
Dyak GE19457PA
0
1
Dere GG12982PA
61
Dper GL24317PA
Dwil GK18569PA
0
926
Dpse GA23991PA
18
807
Dpse GA28704PA
947
Dpse GA28705PA
Dpse GA28682PA
1000
Dpse GA26569PA
5
Dsim GD16984PA
0
Dwil GK16059PA
0
Dwil GK20233PA
240
Dwil GK12385PA
0
720
Dwil GK10431PA
638
Dwil GK18755PA
Dwil GK14272PA
Dwil GK10453PA
684
Dwil GK21270PA
990
306
1000
Dwil GK23351PA
0
Dwil GK20178PA
614
Dwil GK23440PA
185
Dsim brPA

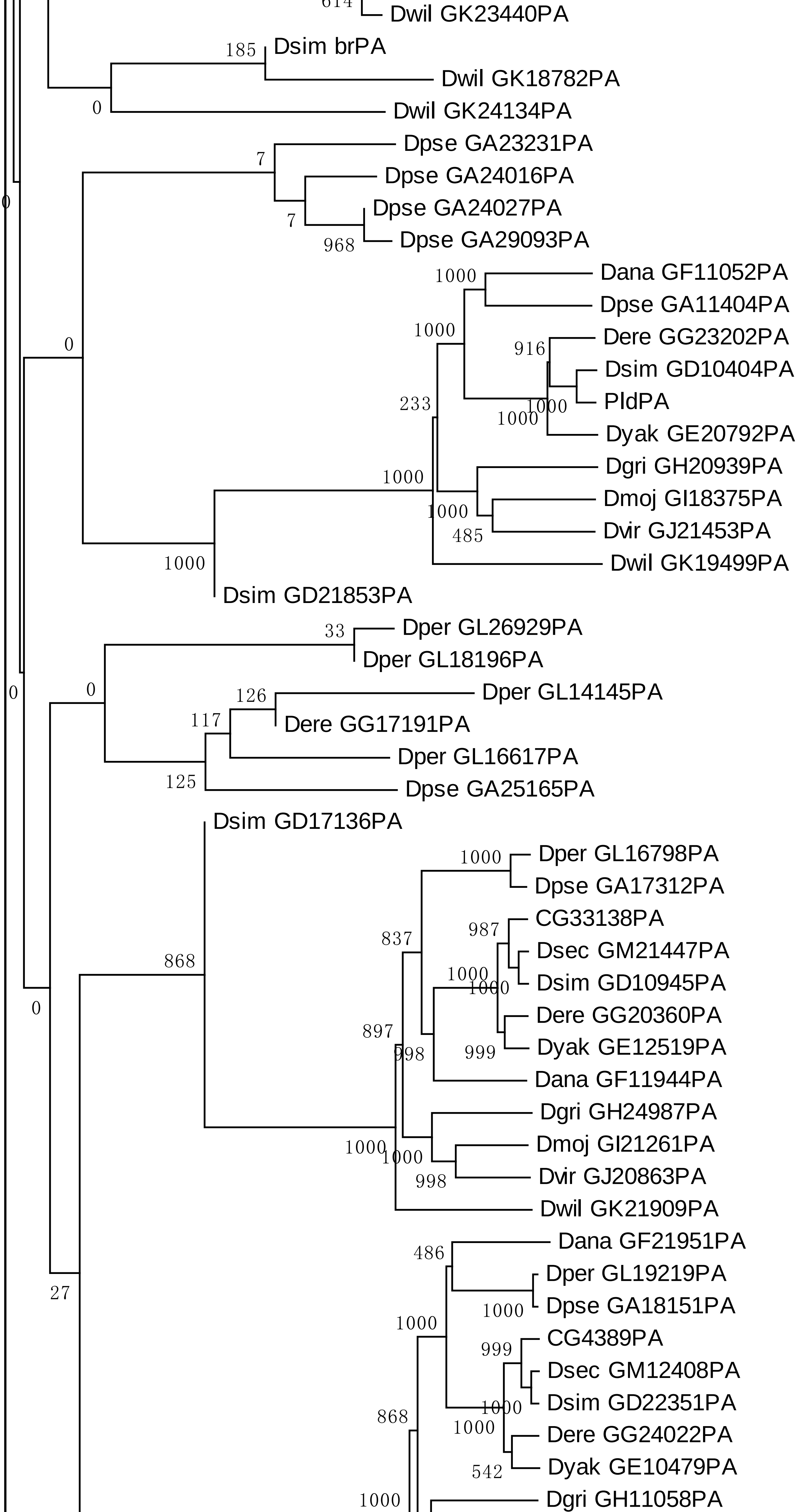

Dwil GK23440PA
185
Dsim brPA
Dwil GK18782PA
0
Dwil GK24134PA
Dpse GA23231PA
7
Dpse GA24016PA
0
Dpse GA24027PA
7
968
Dpse GA29093PA
1000
Dana GF11052PA
Dpse GA11404PA
1000
Dere GG23202PA
916
Dsim GD10404PA
0
PldPA
233
1000
1000
Dyak GE20792PA
Dgri GH20939PA
1000
Dmoj GI18375PA
1000
485
Dvir GJ21453PA
Dwil GK19499PA
1000
Dsim GD21853PA
33
Dper GL26929PA
Dper GL18196PA
0
0
126
Dper GL14145PA
117
Dere GG17191PA
Dper GL16617PA
125
Dpse GA25165PA
Dsim GD17136PA
1000
Dper GL16798PA
Dpse GA17312PA
CG33138PA
987
837
Dsec GM21447PA
868
1000
Dsim GD10945PA
1000
0
Dere GG20360PA
897
Dyak GE12519PA
998
999
Dana GF11944PA
Dgri GH24987PA
Dmoj GI21261PA
1000
1000
998
Dvir GJ20863PA
Dwil GK21909PA
Dana GF21951PA
486
Dper GL19219PA
27
1000
Dpse GA18151PA
1000
999
CG4389PA
Dsec GM12408PA
1000
Dsim GD22351PA
868
1000
Dere GG24022PA
542
Dyak GE10479PA
1000
Dgri GH11058PA

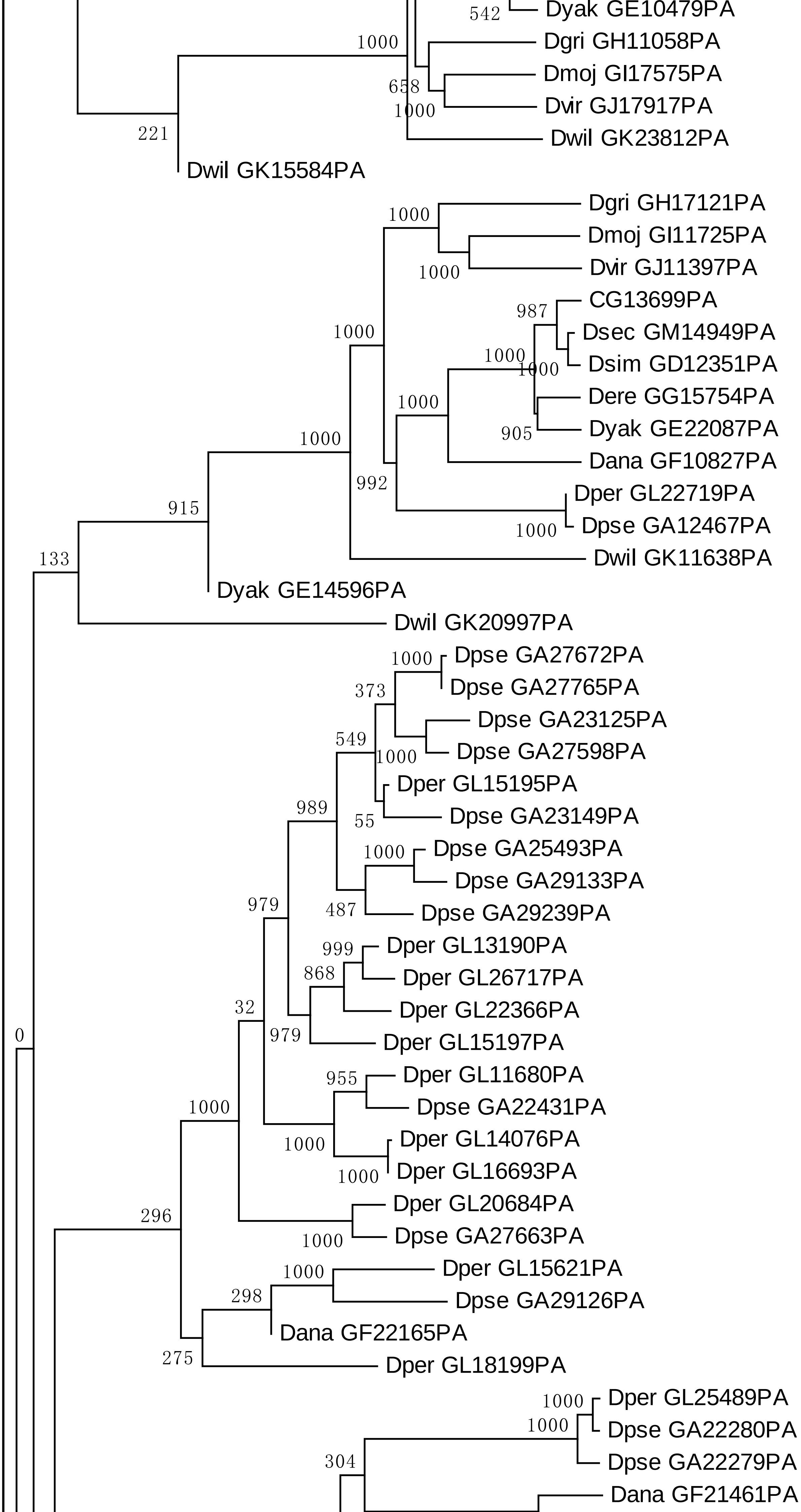
542
Dyak GE10479PA
1000
Dgri GH11058PA
Dmoj GI17575PA
658
Dvir GJ17917PA
1000
Dwil GK23812PA
221
Dwil GK15584PA
Dgri GH17121PA
1000
Dmoj GI11725PA
1000
Dvir GJ11397PA
CG13699PA
987
Dsec GM14949PA
1000
Dsim GD12351PA
1000
1000
Dere GG15754PA
1000
Dyak GE22087PA
905
1000
Dana GF10827PA
992
Dper GL22719PA
Dpse GA12467PA
1000
Dwil GK11638PA
915
133
Dyak GE14596PA
Dwil GK20997PA
1000
Dpse GA27672PA
373
Dpse GA27765PA
Dpse GA23125PA
549
Dpse GA27598PA
1000
Dper GL15195PA
989
Dpse GA23149PA
55
1000
Dpse GA25493PA
Dpse GA29133PA
979
487
Dpse GA29239PA
999
Dper GL13190PA
868
Dper GL26717PA
Dper GL22366PA
32
979
Dper GL15197PA
955
Dper GL11680PA
0
Dpse GA22431PA
1000
Dper GL14076PA
1000
1000
Dper GL16693PA
Dper GL20684PA
296
1000
Dpse GA27663PA
1000
Dper GL15621PA
298
Dpse GA29126PA
Dana GF22165PA
275
Dper GL18199PA
1000
Dper GL25489PA
1000
Dpse GA22280PA
304
Dpse GA22279PA
Dana GF21461PA

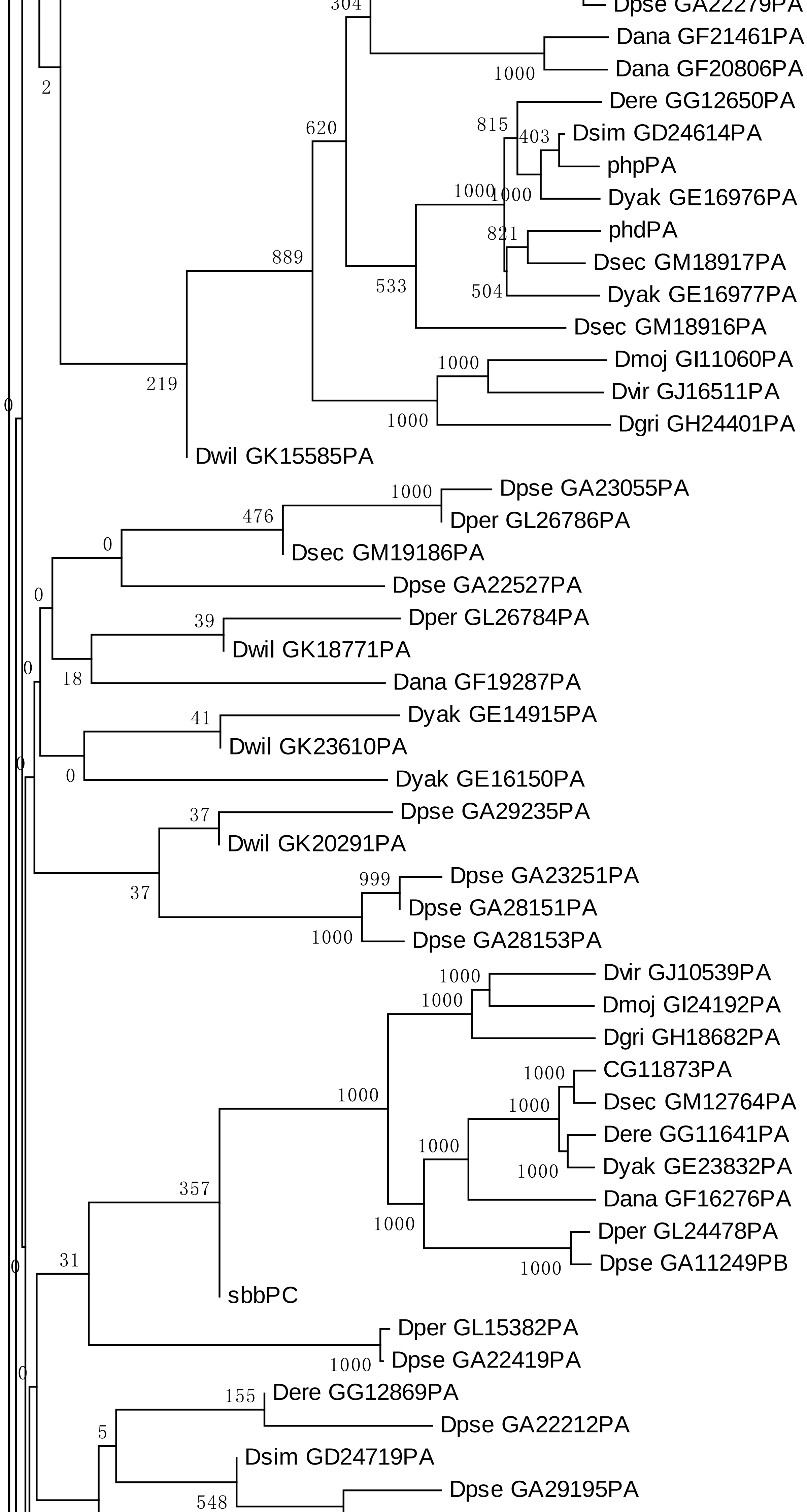

304
Dpse GA22279PA
Dana GF21461PA
2
1000
Dana GF20806PA
Dere GG12650PA
620
815
403
Dsim GD24614PA
phpPA
1000
1000
Dyak GE16976PA
821
phdPA
889
Dsec GM18917PA
533
504
Dyak GE16977PA
Dsec GM18916PA
1000
Dmoj GI11060PA
219
Dvir GJ16511PA
0
1000
Dgri GH24401PA
Dwil GK15585PA
1000
Dpse GA23055PA
476
Dper GL26786PA
0
Dsec GM19186PA
0
Dpse GA22527PA
39
Dper GL26784PA
Dwil GK18771PA
0
18
Dana GF19287PA
41
Dyak GE14915PA
Dwil GK23610PA
0
0
Dyak GE16150PA
37
Dpse GA29235PA
Dwil GK20291PA
999
Dpse GA23251PA
37
Dpse GA28151PA
1000
Dpse GA28153PA
1000
Dvir GJ10539PA
1000
Dmoj GI24192PA
Dgri GH18682PA
1000
CG11873PA
1000
1000
Dsec GM12764PA
Dere GG11641PA
1000
357
1000
Dyak GE23832PA
Dana GF16276PA
1000
Dper GL24478PA
31
1000
Dpse GA11249PB
0
sbbPC
Dper GL15382PA
1000
Dpse GA22419PA
0
155
Dere GG12869PA
5
Dpse GA22212PA
Dsim GD24719PA
Dpse GA29195PA
548

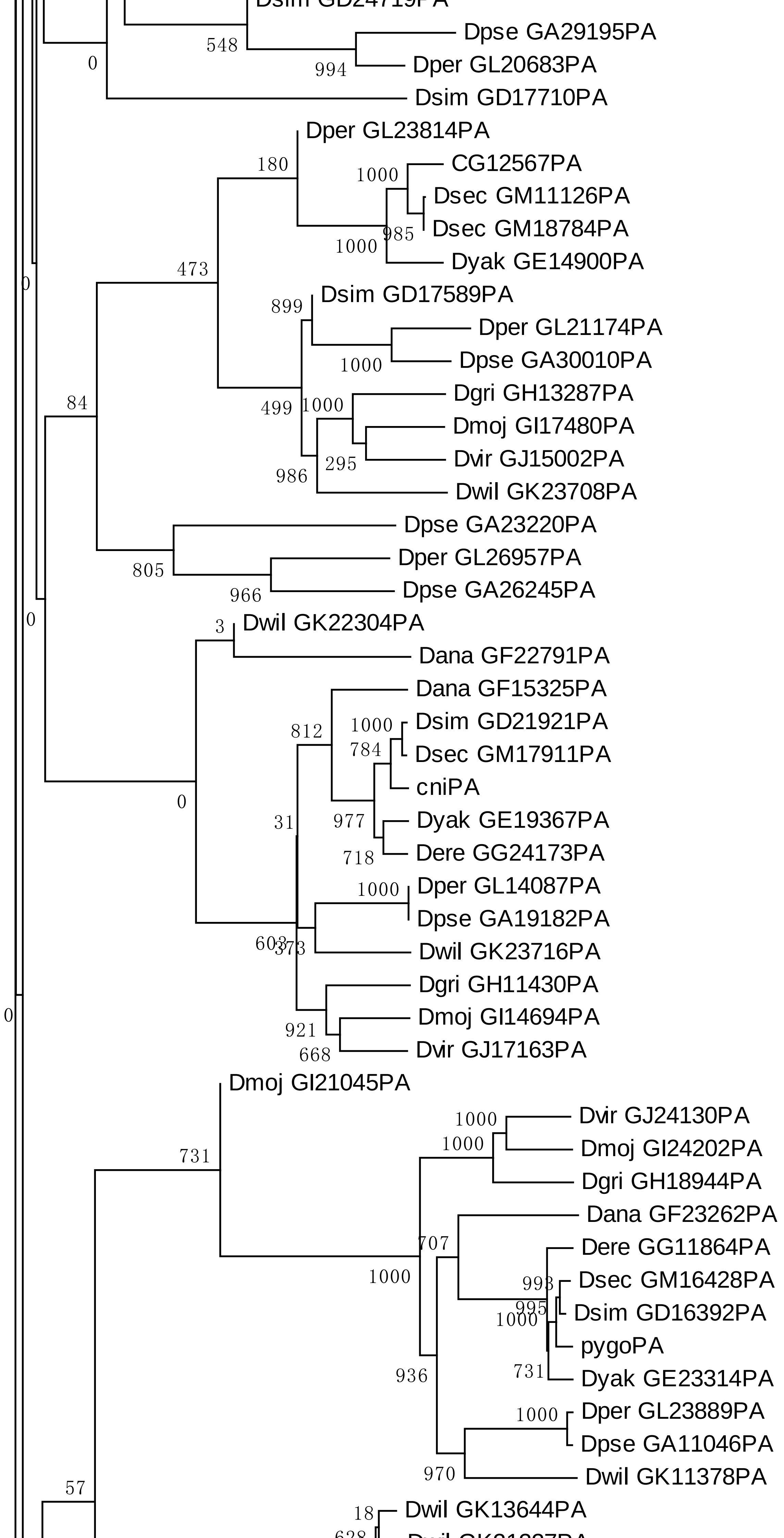
Dpse GA29195PA
548
Dper GL20683PA
994
0
Dsim GD17710PA
Dper GL23814PA
180
CG12567PA
1000
Dsec GM11126PA
Dsec GM18784PA
985
1000
Dyak GE14900PA
473
0
Dsim GD17589PA
899
Dper GL21174PA
1000
Dpse GA30010PA
Dgri GH13287PA
499
1000
84
Dmoj GI17480PA
Dvir GJ15002PA
295
986
Dwil GK23708PA
Dpse GA23220PA
Dper GL26957PA
805
Dpse GA26245PA
966
Dwil GK22304PA
3
0
Dana GF22791PA
Dana GF15325PA
Dsim GD21921PA
812
1000
784
Dsec GM17911PA
cniPA
0
31
977
Dyak GE19367PA
718
Dere GG24173PA
Dper GL14087PA
1000
Dpse GA19182PA
603
373
Dwil GK23716PA
Dgri GH11430PA
0
Dmoj GI14694PA
921
668
Dvir GJ17163PA
Dmoj GI21045PA
1000
Dvir GJ24130PA
1000
Dmoj GI24202PA
731
Dgri GH18944PA
Dana GF23262PA
707
Dere GG11864PA
1000
993
Dsec GM16428PA
995
Dsim GD16392PA
1000
pygoPA
936
731
Dyak GE23314PA
1000
Dper GL23889PA
Dpse GA11046PA
970
Dwil GK11378PA
57
18
Dwil GK13644PA

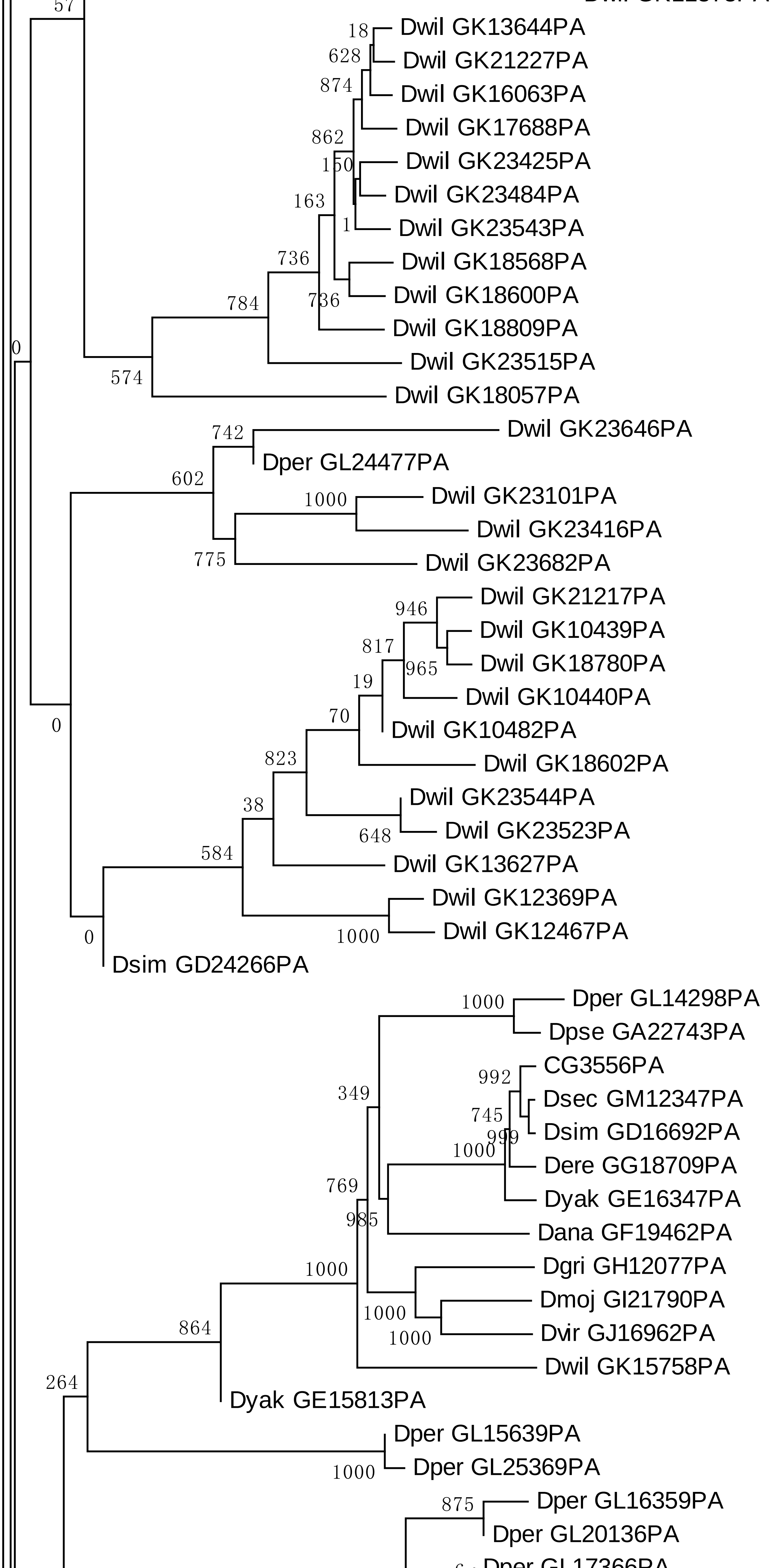

57
18
Dwil GK13644PA
628
Dwil GK21227PA
874
Dwil GK16063PA
Dwil GK17688PA
862
150
Dwil GK23425PA
163
Dwil GK23484PA
1
Dwil GK23543PA
736
Dwil GK18568PA
784
736
Dwil GK18600PA
Dwil GK18809PA
0
Dwil GK23515PA
574
Dwil GK18057PA
742
Dwil GK23646PA
Dper GL24477PA
602
1000
Dwil GK23101PA
Dwil GK23416PA
775
Dwil GK23682PA
946
Dwil GK21217PA
817
Dwil GK10439PA
965
Dwil GK18780PA
19
Dwil GK10440PA
70
0
Dwil GK10482PA
823
Dwil GK18602PA
Dwil GK23544PA
38
648
Dwil GK23523PA
584
Dwil GK13627PA
Dwil GK12369PA
0
1000
Dwil GK12467PA
Dsim GD24266PA
1000
Dper GL14298PA
Dpse GA22743PA
992
CG3556PA
349
Dsec GM12347PA
745
Dsim GD16692PA
999
1000
Dere GG18709PA
769
Dyak GE16347PA
985
Dana GF19462PA
1000
Dgri GH12077PA
Dmoj GI21790PA
1000
864
1000
Dvir GJ16962PA
Dwil GK15758PA
264
Dyak GE15813PA
Dper GL15639PA
1000
Dper GL25369PA
875
Dper GL16359PA
Dper GL20136PA

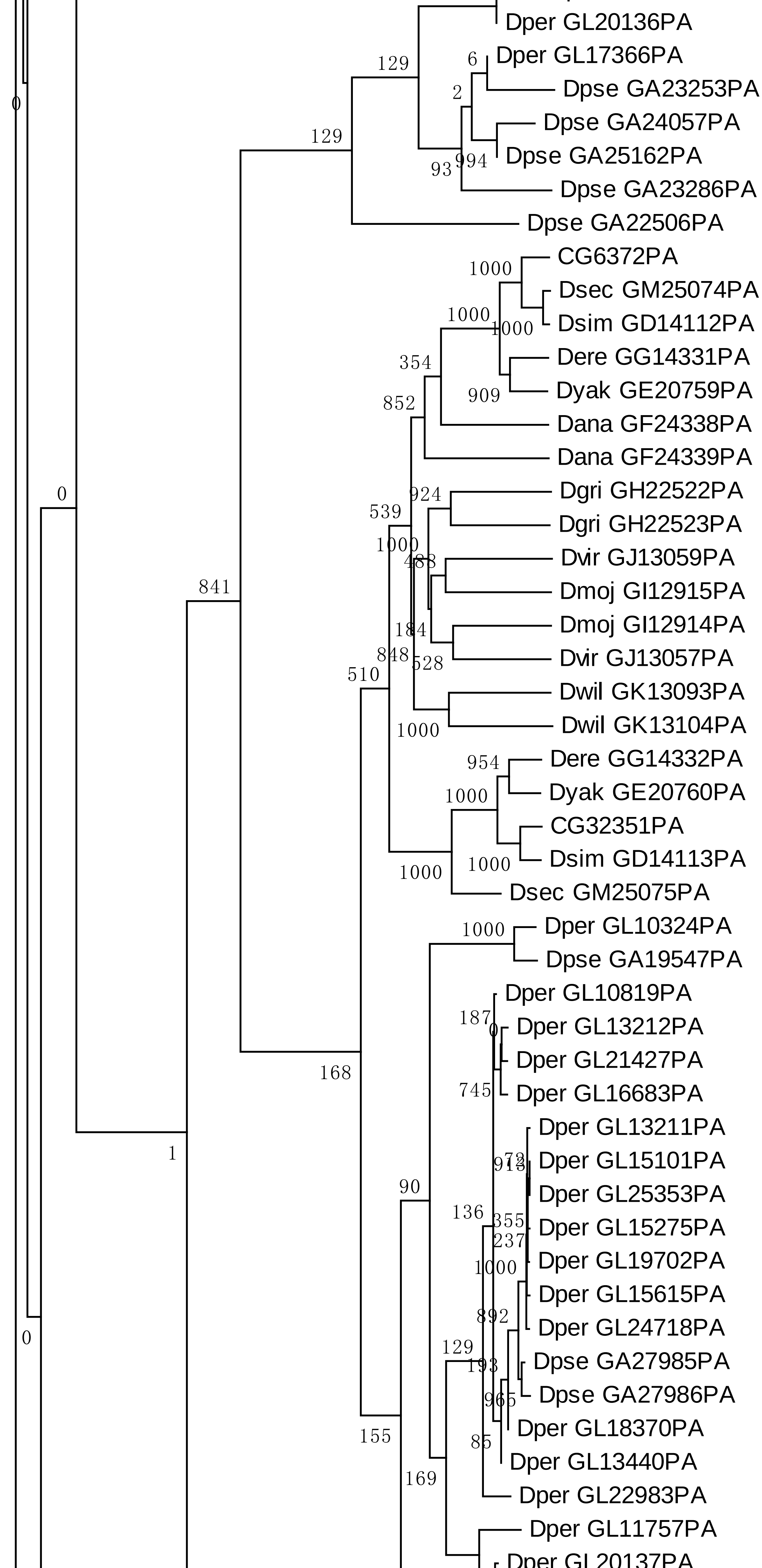

Dper GL20136PA
Dper GL17366PA
Dpse GA23253PA
Dpse GA24057PA
Dpse GA25162PA
Dpse GA23286PA
Dpse GA22506PA
CG6372PA
Dsec GM25074PA
Dsim GD14112PA
Dere GG14331PA
Dyak GE20759PA
Dana GF24338PA
Dana GF24339PA
Dgri GH22522PA
Dgri GH22523PA
Dvir GJ13059PA
Dmoj GI12915PA
Dmoj GI12914PA
Dvir GJ13057PA
Dwil GK13093PA
Dwil GK13104PA
Dere GG14332PA
Dyak GE20760PA
CG32351PA
Dsim GD14113PA
Dsec GM25075PA
Dper GL10324PA
Dpse GA19547PA
Dper GL10819PA
Dper GL13212PA
Dper GL21427PA
Dper GL16683PA
Dper GL13211PA
Dper GL15101PA
Dper GL25353PA
Dper GL15275PA
Dper GL19702PA
Dper GL15615PA
Dper GL24718PA
Dpse GA27985PA
Dpse GA27986PA
Dper GL18370PA
Dper GL13440PA
Dper GL22983PA
Dper GL11757PA
Dper GL20137PA
0
129
6
129
2
93
994
1000
1000
1000
354
909
852
0
924
539
1000
488
841
184
848
528
510
1000
954
1000
1000
1000
1000
187
0
168
745
1
913
72
90
136
355
237
1000
892
0
129
193
965
155
85
169

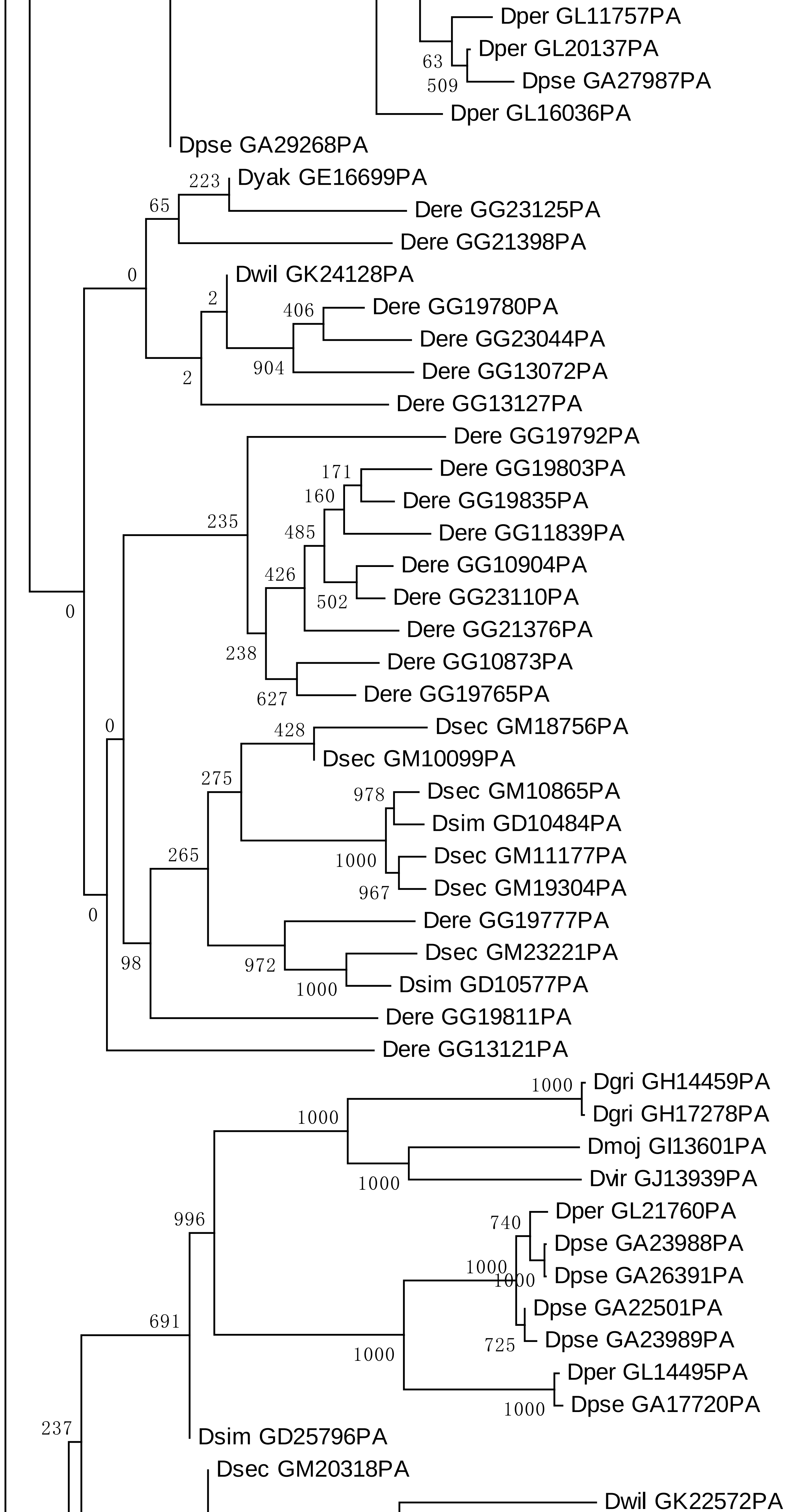
Dper GL11757PA
Dper GL20137PA
63
509
Dpse GA27987PA
Dper GL16036PA
Dpse GA29268PA
223
Dyak GE16699PA
65
Dere GG23125PA
Dere GG21398PA
0
Dwil GK24128PA
2
406
Dere GG19780PA
Dere GG23044PA
904
Dere GG13072PA
2
Dere GG13127PA
Dere GG19792PA
171
Dere GG19803PA
160
Dere GG19835PA
235
485
Dere GG11839PA
Dere GG10904PA
426
502
Dere GG23110PA
0
Dere GG21376PA
238
Dere GG10873PA
627
Dere GG19765PA
0
428
Dsec GM18756PA
Dsec GM10099PA
275
978
Dsec GM10865PA
Dsim GD10484PA
265
1000
Dsec GM11177PA
967
Dsec GM19304PA
0
Dere GG19777PA
98
972
Dsec GM23221PA
1000
Dsim GD10577PA
Dere GG19811PA
Dere GG13121PA
1000
Dgri GH14459PA
1000
Dgri GH17278PA
Dmoj GI13601PA
1000
Dvir GJ13939PA
740
Dper GL21760PA
996
Dpse GA23988PA
1000
1000
Dpse GA26391PA
Dpse GA22501PA
691
725
Dpse GA23989PA
1000
Dper GL14495PA
1000
Dpse GA17720PA
237
Dsim GD25796PA
Dsec GM20318PA
Dwil GK22572PA

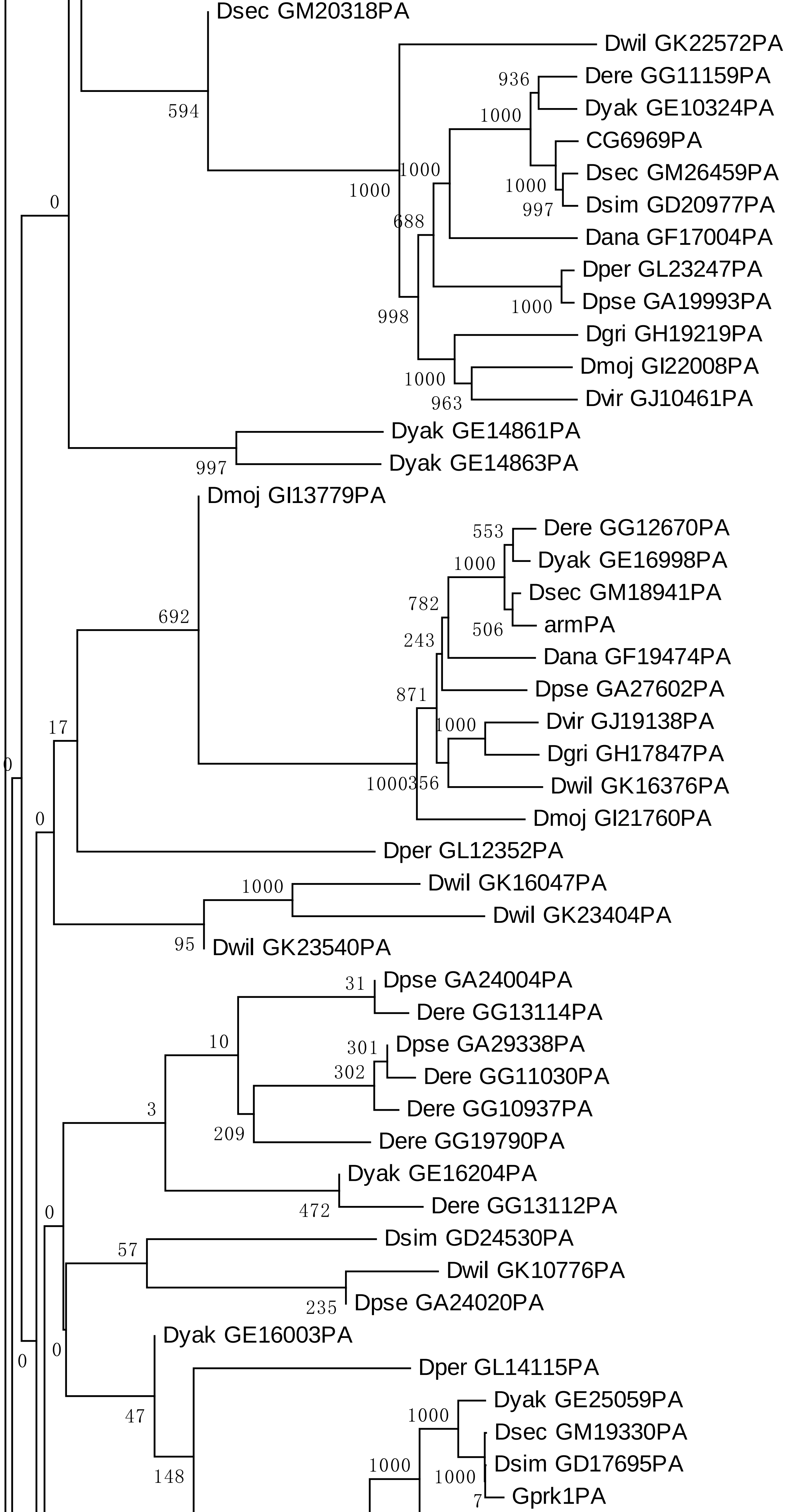
Dsec GM20318PA
Dwil GK22572PA
936
Dere GG11159PA
Dyak GE10324PA
594
1000
CG6969PA
1000
Dsec GM26459PA
1000
1000
0
997
Dsim GD20977PA
688
Dana GF17004PA
Dper GL23247PA
1000
Dpse GA19993PA
998
Dgri GH19219PA
1000
Dmoj GI22008PA
963
Dvir GJ10461PA
Dyak GE14861PA
997
Dyak GE14863PA
Dmoj GI13779PA
553
Dere GG12670PA
1000
Dyak GE16998PA
782
Dsec GM18941PA
692
506
armPA
243
Dana GF19474PA
871
Dpse GA27602PA
1000
Dvir GJ19138PA
17
Dgri GH17847PA
0
1000356
Dwil GK16376PA
Dmoj GI21760PA
0
Dper GL12352PA
1000
Dwil GK16047PA
Dwil GK23404PA
95
Dwil GK23540PA
31
Dpse GA24004PA
Dere GG13114PA
10
301
Dpse GA29338PA
302
Dere GG11030PA
3
Dere GG10937PA
209
Dere GG19790PA
Dyak GE16204PA
472
Dere GG13112PA
0
Dsim GD24530PA
57
Dwil GK10776PA
235
Dpse GA24020PA
0
Dyak GE16003PA
0
Dper GL14115PA
47
1000
Dyak GE25059PA
Dsec GM19330PA
148
1000
Dsim GD17695PA
1000
7
Gprk1PA

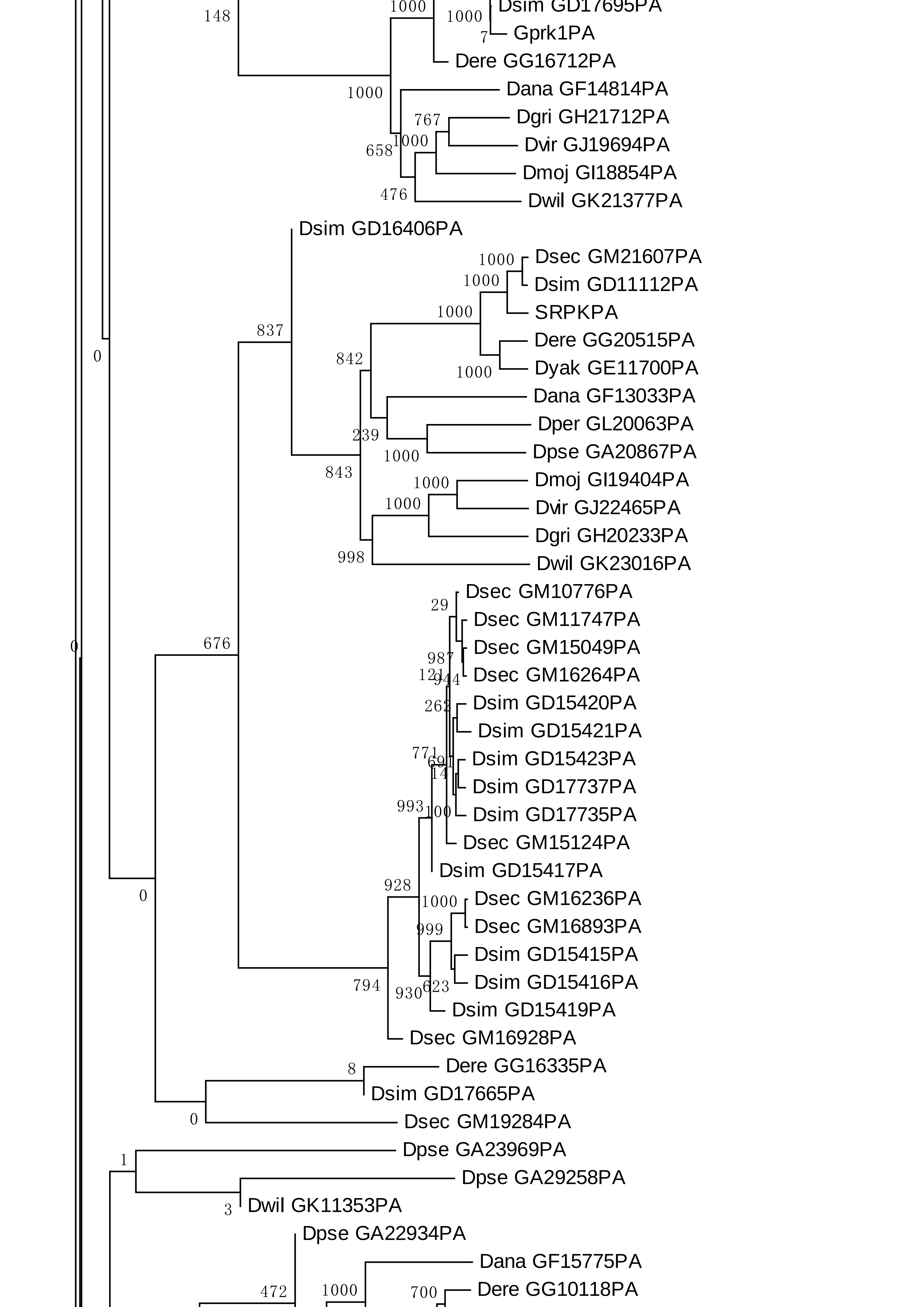

Dsim GD17695PA
Gprk1PA
Dere GG16712PA
Dana GF14814PA
Dgri GH21712PA
Dvir GJ19694PA
Dmoj GI18854PA
Dwil GK21377PA
Dsim GD16406PA
Dsec GM21607PA
Dsim GD11112PA
SRPKPA
Dere GG20515PA
Dyak GE11700PA
Dana GF13033PA
Dper GL20063PA
Dpse GA20867PA
Dmoj GI19404PA
Dvir GJ22465PA
Dgri GH20233PA
Dwil GK23016PA
Dsec GM10776PA
Dsec GM11747PA
Dsec GM15049PA
Dsec GM16264PA
Dsim GD15420PA
Dsim GD15421PA
Dsim GD15423PA
Dsim GD17737PA
Dsim GD17735PA
Dsec GM15124PA
Dsim GD15417PA
Dsec GM16236PA
Dsec GM16893PA
Dsim GD15415PA
Dsim GD15416PA
Dsim GD15419PA
Dsec GM16928PA
Dere GG16335PA
Dsim GD17665PA
Dsec GM19284PA
Dpse GA23969PA
Dpse GA29258PA
Dwil GK11353PA
Dpse GA22934PA
Dana GF15775PA
Dere GG10118PA

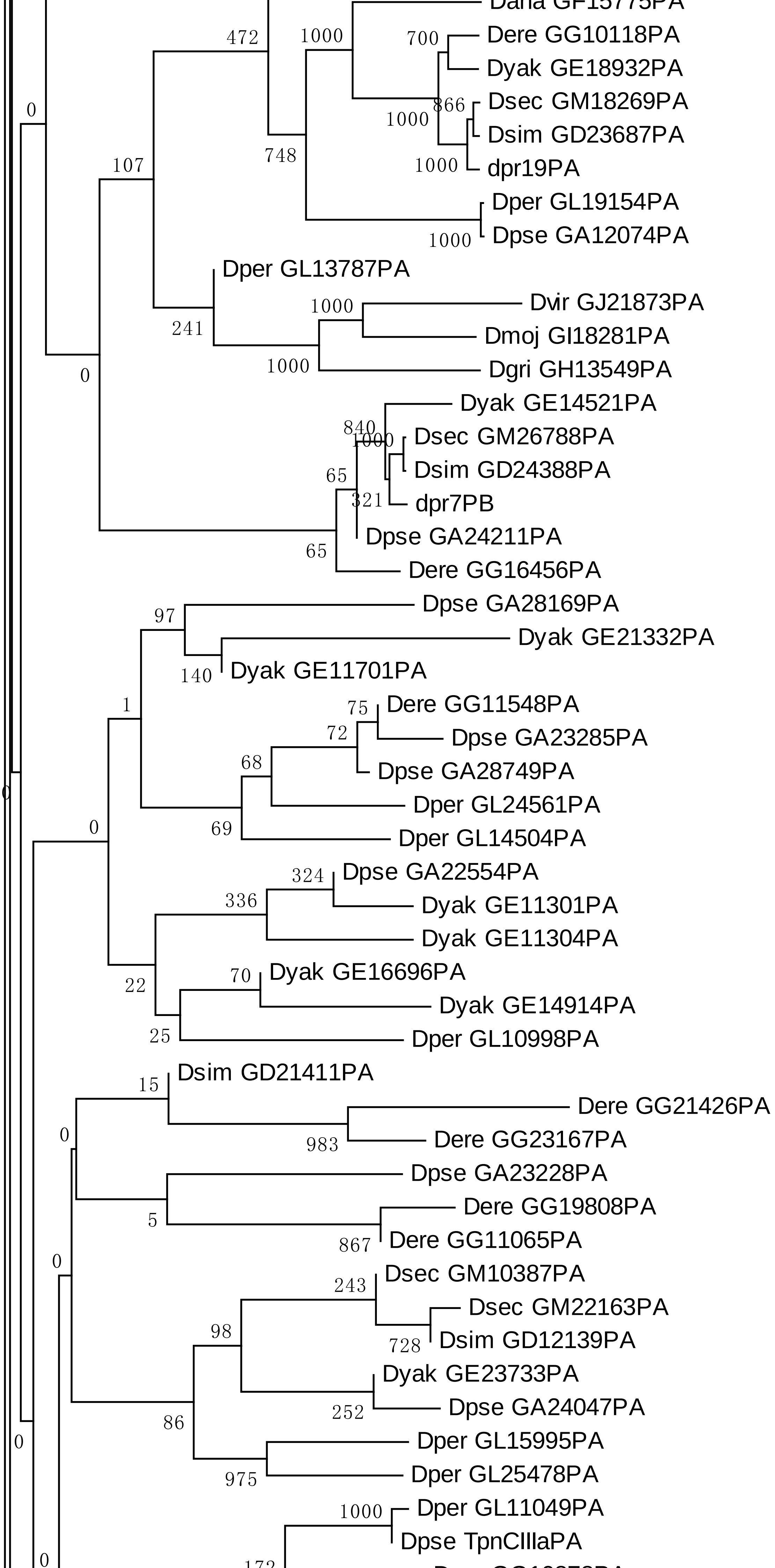
Dere GG10118PA
Dyak GE18932PA
Dsec GM18269PA
Dsim GD23687PA
dpr19PA
Dper GL19154PA
Dpse GA12074PA
Dper GL13787PA
Dvir GJ21873PA
Dmoj GI18281PA
Dgri GH13549PA
Dyak GE14521PA
Dsec GM26788PA
Dsim GD24388PA
dpr7PB
Dpse GA24211PA
Dere GG16456PA
Dpse GA28169PA
Dyak GE21332PA
Dyak GE11701PA
Dere GG11548PA
Dpse GA23285PA
Dpse GA28749PA
Dper GL24561PA
Dper GL14504PA
Dpse GA22554PA
Dyak GE11301PA
Dyak GE11304PA
Dyak GE16696PA
Dyak GE14914PA
Dper GL10998PA
Dsim GD21411PA
Dere GG21426PA
Dere GG23167PA
Dpse GA23228PA
Dere GG19808PA
Dere GG11065PA
Dsec GM10387PA
Dsec GM22163PA
Dsim GD12139PA
Dyak GE23733PA
Dpse GA24047PA
Dper GL15995PA
Dper GL25478PA
Dper GL11049PA
Dpse TpnCIIIaPA

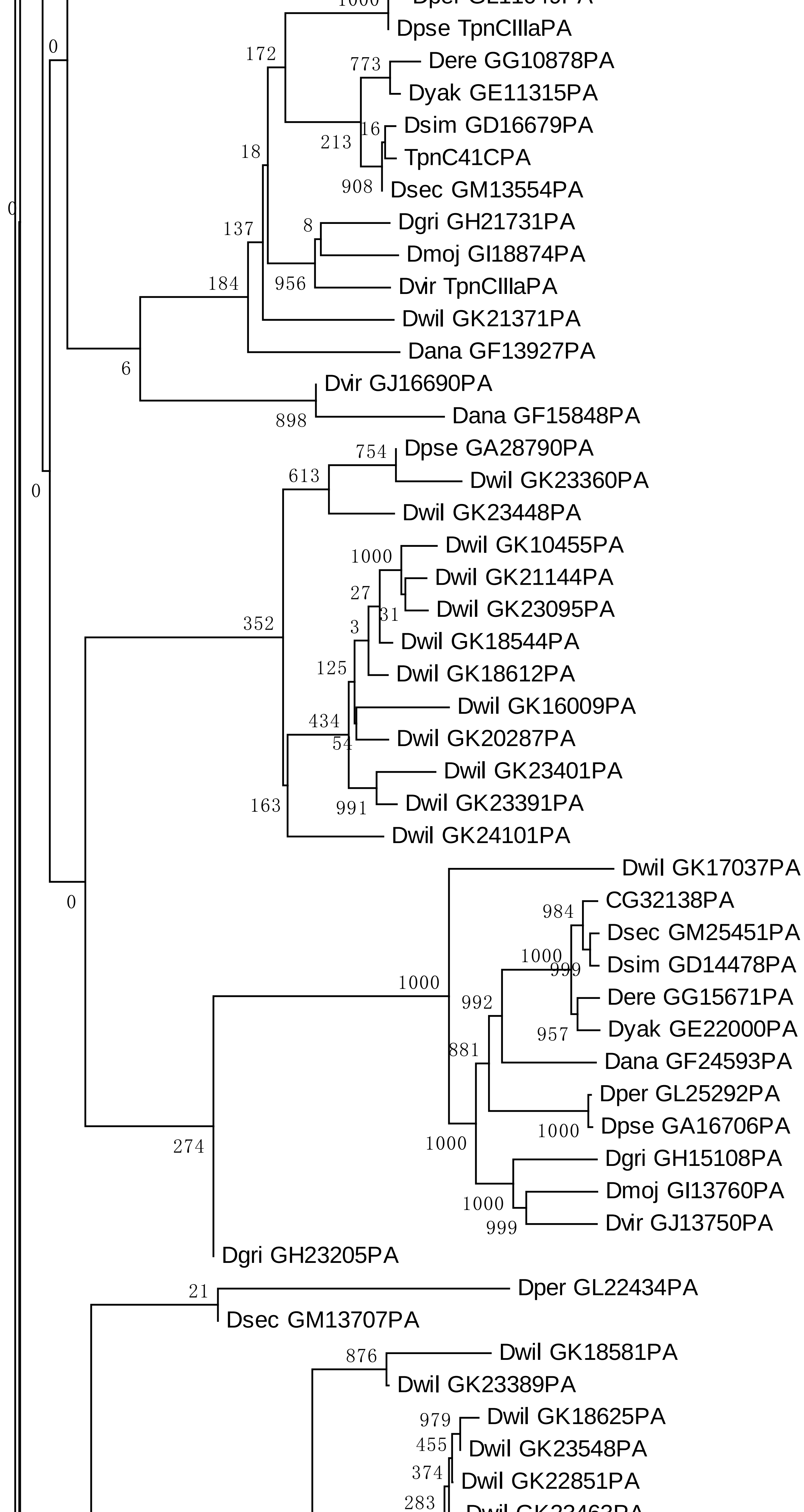
Dpse TpnCIIIaPA
Dere GG10878PA
Dyak GE11315PA
Dsim GD16679PA
TpnC41CPA
Dsec GM13554PA
Dgri GH21731PA
Dmoj GI18874PA
Dvir TpnCIIIaPA
Dwil GK21371PA
Dana GF13927PA
Dvir GJ16690PA
Dana GF15848PA
Dpse GA28790PA
Dwil GK23360PA
Dwil GK23448PA
Dwil GK10455PA
Dwil GK21144PA
Dwil GK23095PA
Dwil GK18544PA
Dwil GK18612PA
Dwil GK16009PA
Dwil GK20287PA
Dwil GK23401PA
Dwil GK23391PA
Dwil GK24101PA
Dwil GK17037PA
CG32138PA
Dsec GM25451PA
Dsim GD14478PA
Dere GG15671PA
Dyak GE22000PA
Dana GF24593PA
Dper GL25292PA
Dpse GA16706PA
Dgri GH15108PA
Dmoj GI13760PA
Dvir GJ13750PA
Dgri GH23205PA
Dper GL22434PA
Dsec GM13707PA
Dwil GK18581PA
Dwil GK23389PA
Dwil GK18625PA
Dwil GK23548PA
Dwil GK22851PA

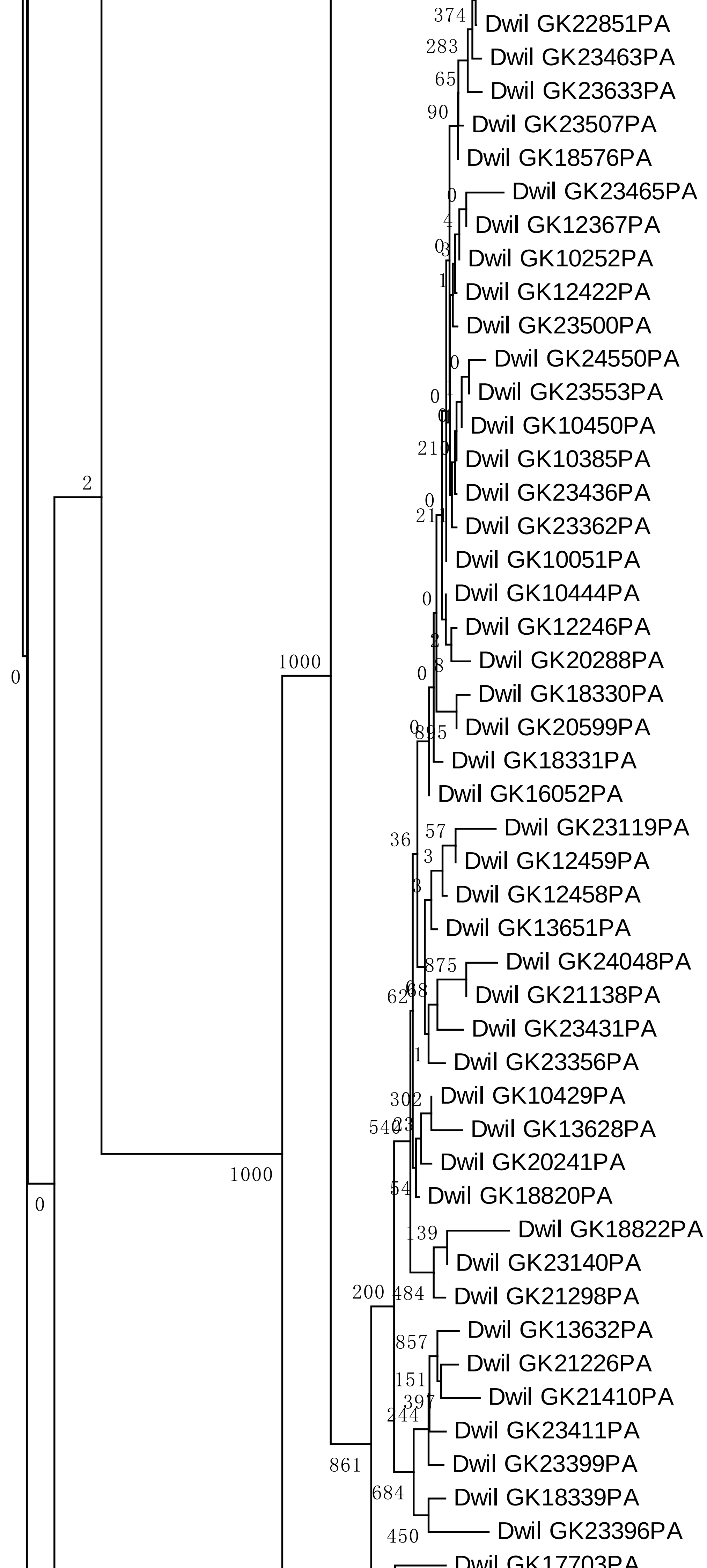
Dwil GK22851PA
Dwil GK23463PA
Dwil GK23633PA
Dwil GK23507PA
Dwil GK18576PA
Dwil GK23465PA
Dwil GK12367PA
Dwil GK10252PA
Dwil GK12422PA
Dwil GK23500PA
Dwil GK24550PA
Dwil GK23553PA
Dwil GK10450PA
Dwil GK10385PA
Dwil GK23436PA
Dwil GK23362PA
Dwil GK10051PA
Dwil GK10444PA
Dwil GK12246PA
Dwil GK20288PA
Dwil GK18330PA
Dwil GK20599PA
Dwil GK18331PA
Dwil GK16052PA
Dwil GK23119PA
Dwil GK12459PA
Dwil GK12458PA
Dwil GK13651PA
Dwil GK24048PA
Dwil GK21138PA
Dwil GK23431PA
Dwil GK23356PA
Dwil GK10429PA
Dwil GK13628PA
Dwil GK20241PA
Dwil GK18820PA
Dwil GK18822PA
Dwil GK23140PA
Dwil GK21298PA
Dwil GK13632PA
Dwil GK21226PA
Dwil GK21410PA
Dwil GK23411PA
Dwil GK23399PA
Dwil GK18339PA
Dwil GK23396PA
Dwil GK17703PA

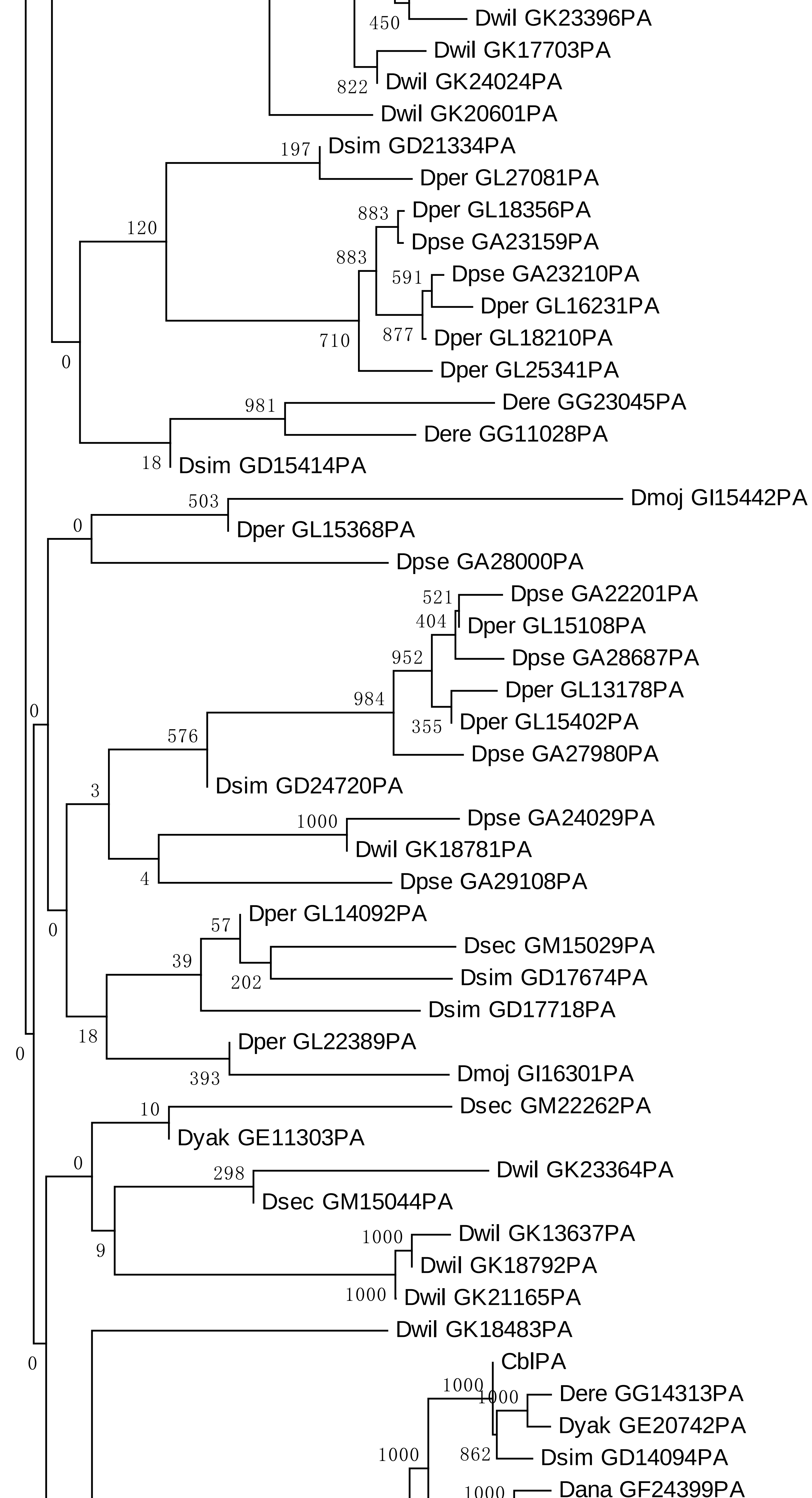
450
Dwil GK23396PA
Dwil GK17703PA
822
Dwil GK24024PA
Dwil GK20601PA
197
Dsim GD21334PA
Dper GL27081PA
883
Dper GL18356PA
120
Dpse GA23159PA
883
591
Dpse GA23210PA
Dper GL16231PA
877
Dper GL18210PA
710
0
Dper GL25341PA
981
Dere GG23045PA
Dere GG11028PA
18
Dsim GD15414PA
503
Dmoj GI15442PA
0
Dper GL15368PA
Dpse GA28000PA
521
Dpse GA22201PA
404
Dper GL15108PA
952
Dpse GA28687PA
984
Dper GL13178PA
0
355
Dper GL15402PA
576
Dpse GA27980PA
Dsim GD24720PA
3
1000
Dpse GA24029PA
Dwil GK18781PA
4
Dpse GA29108PA
0
Dper GL14092PA
57
Dsec GM15029PA
39
202
Dsim GD17674PA
Dsim GD17718PA
18
Dper GL22389PA
0
393
Dmoj GI16301PA
10
Dsec GM22262PA
Dyak GE11303PA
0
298
Dwil GK23364PA
Dsec GM15044PA
1000
Dwil GK13637PA
9
Dwil GK18792PA
1000
Dwil GK21165PA
Dwil GK18483PA
0
CblPA
1000
1000
Dere GG14313PA
Dyak GE20742PA
1000
862
Dsim GD14094PA
1000
Dana GF24399PA

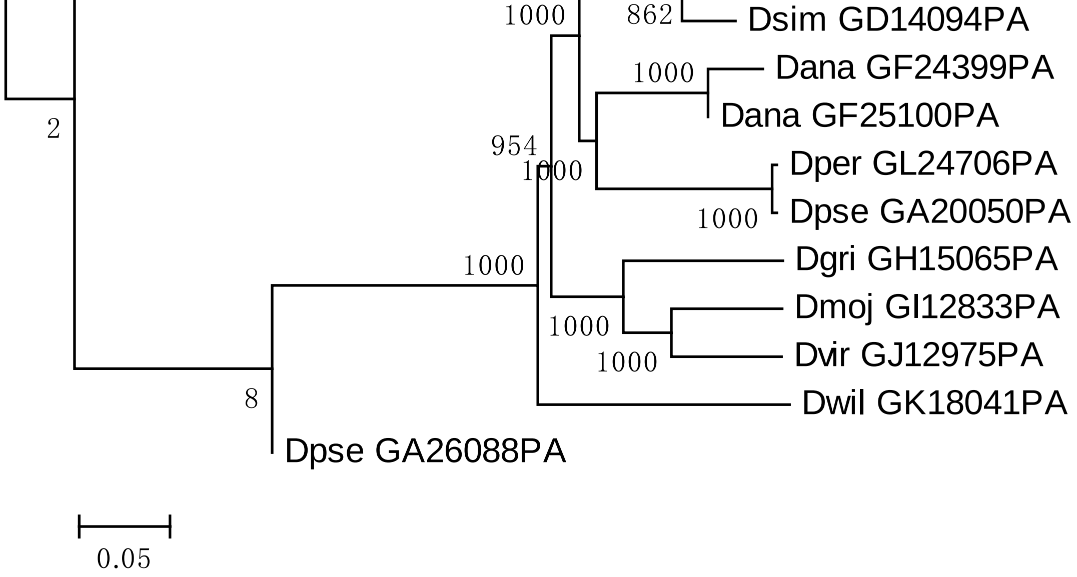

1000
862
Dsim GD14094PA
1000
Dana GF24399PA
Dana GF25100PA
2
954
1000
Dper GL24706PA
1000
Dpse GA20050PA
1000
Dgri GH15065PA
1000
Dmoj GI12833PA
1000
Dvir GJ12975PA
8
Dwil GK18041PA
Dpse GA26088PA
0.05

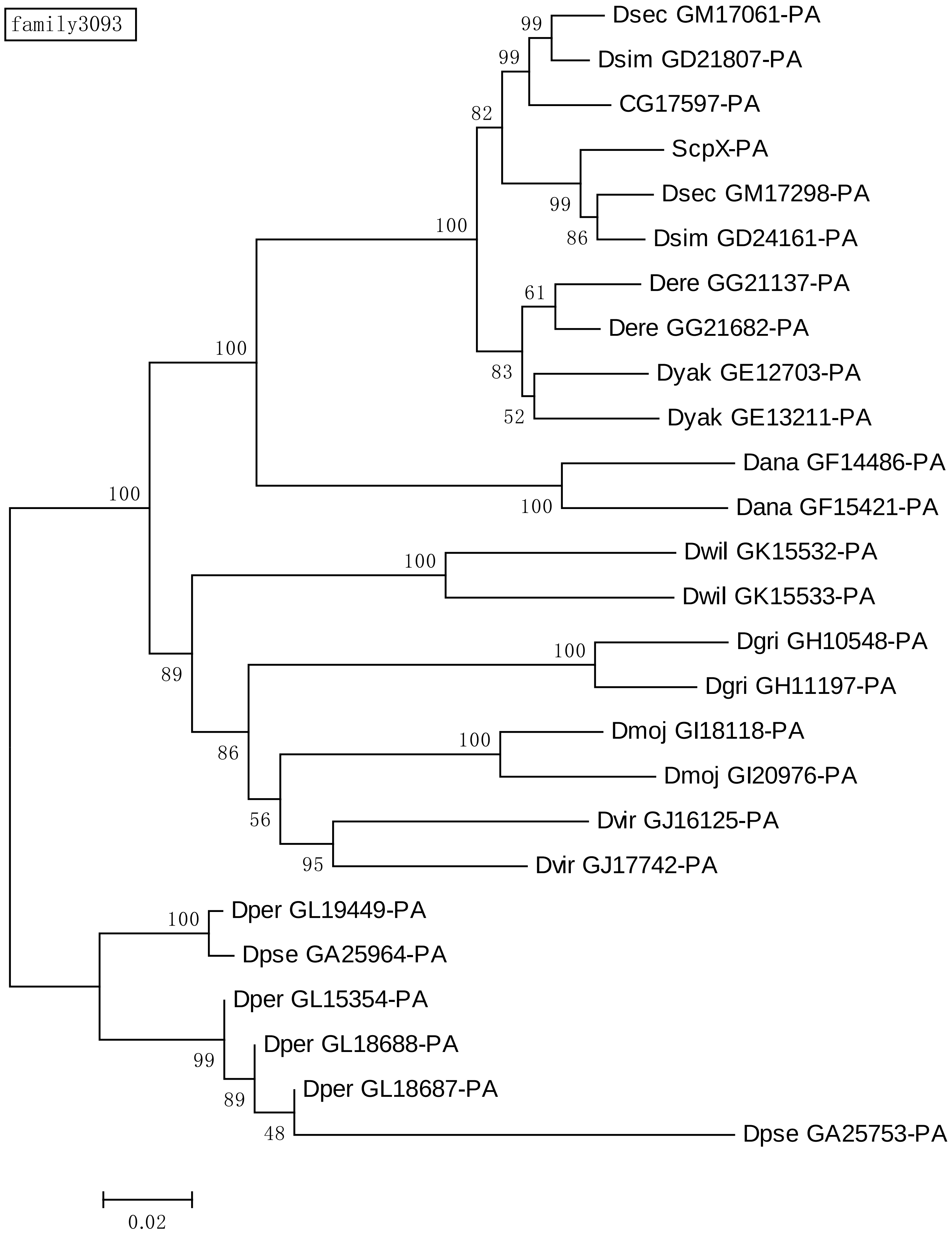
family3093
Dsec GM17061-PA
Dsim GD21807-PA
CG17597-PA
ScpX-PA
Dsec GM17298-PA
Dsim GD24161-PA
Dere GG21137-PA
Dere GG21682-PA
Dyak GE12703-PA
Dyak GE13211-PA
Dana GF14486-PA
Dana GF15421-PA
Dwil GK15532-PA
Dwil GK15533-PA
Dgri GH10548-PA
Dgri GH11197-PA
Dmoj GI18118-PA
Dmoj GI20976-PA
Dvir GJ16125-PA
Dvir GJ17742-PA
Dper GL19449-PA
Dpse GA25964-PA
Dper GL15354-PA
Dper GL18688-PA
Dper GL18687-PA
Dpse GA25753-PA
99
99
82
99
86
100
61
83
52
100
100
100
100
89
100
86
100
56
95
100
99
89
48
0.02

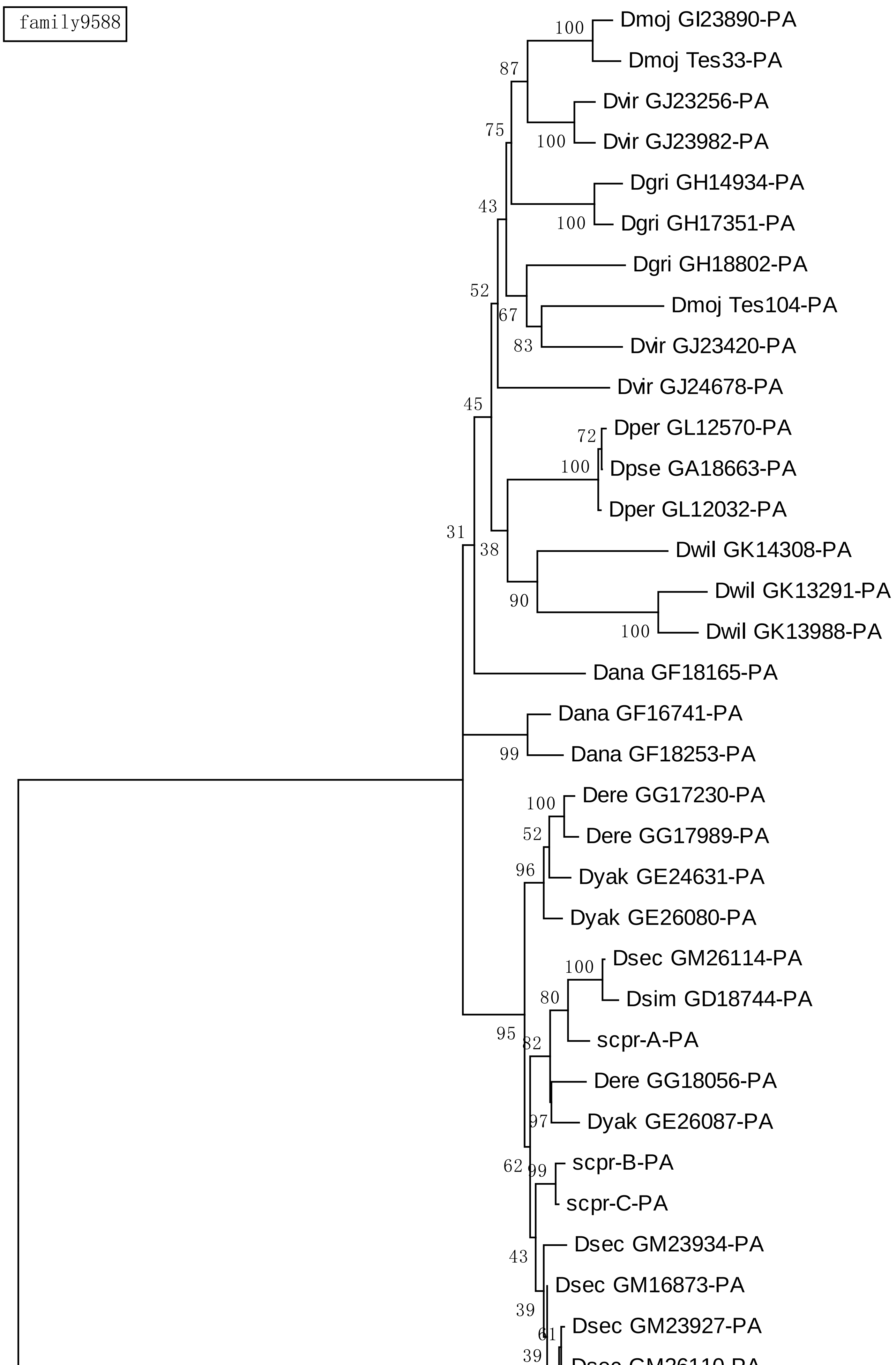

family9588
Dmoj GI23890-PA
Dmoj Tes33-PA
Dvir GJ23256-PA
Dvir GJ23982-PA
Dgri GH14934-PA
Dgri GH17351-PA
Dgri GH18802-PA
Dmoj Tes104-PA
Dvir GJ23420-PA
Dvir GJ24678-PA
Dper GL12570-PA
Dpse GA18663-PA
Dper GL12032-PA
Dwil GK14308-PA
Dwil GK13291-PA
Dwil GK13988-PA
Dana GF18165-PA
Dana GF16741-PA
Dana GF18253-PA
Dere GG17230-PA
Dere GG17989-PA
Dyak GE24631-PA
Dyak GE26080-PA
Dsec GM26114-PA
Dsim GD18744-PA
scpr-A-PA
Dere GG18056-PA
Dyak GE26087-PA
scpr-B-PA
scpr-C-PA
Dsec GM23934-PA
Dsec GM16873-PA
Dsec GM23927-PA
100
87
100
75
100
43
52
67
83
45
72
100
31
38
90
100
99
100
52
96
100
80
95
82
97
62
99
43
39
61
39

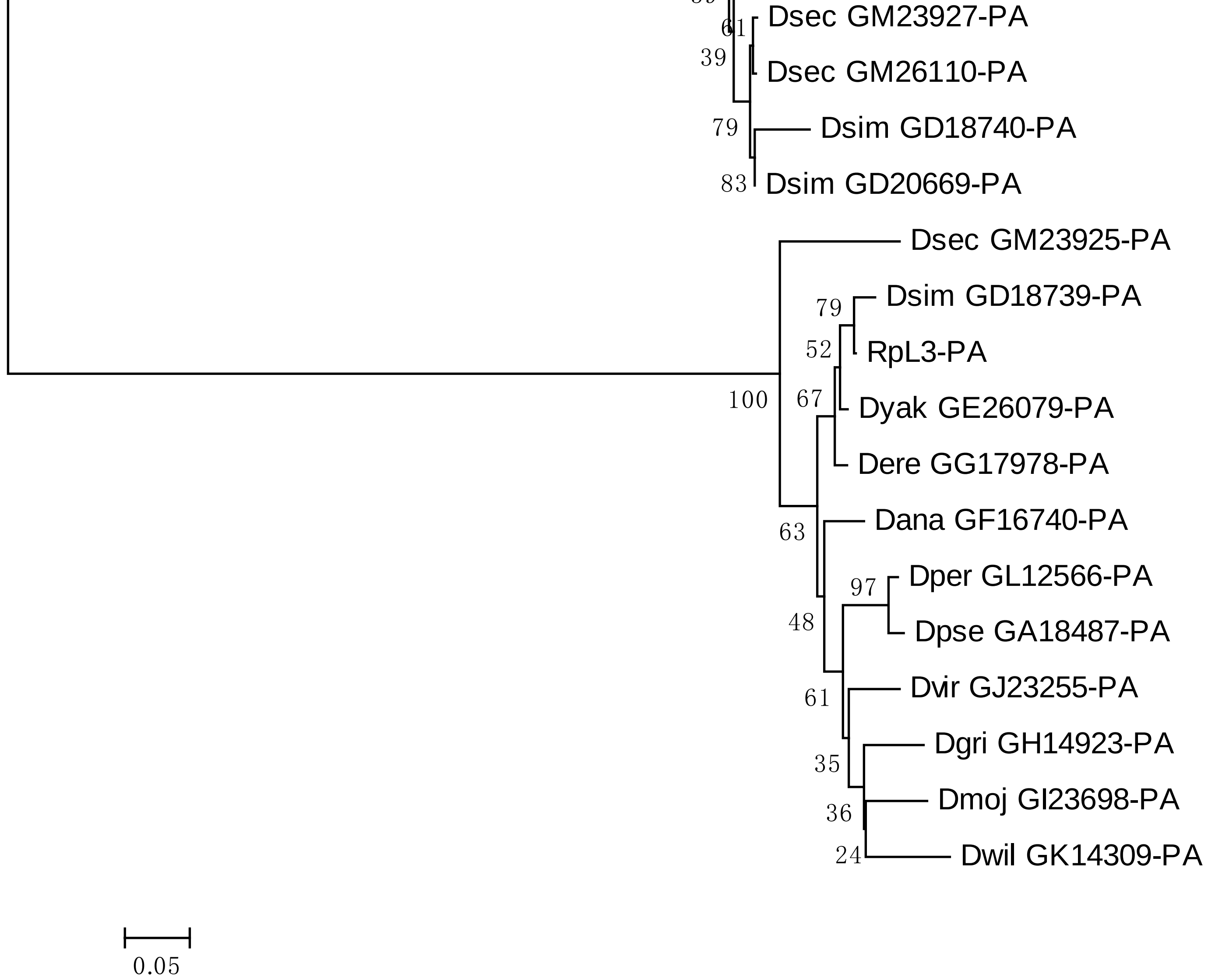
Dsec GM23927-PA
61
39
Dsec GM26110-PA
79
Dsim GD18740-PA
83
Dsim GD20669-PA
Dsec GM23925-PA
79
Dsim GD18739-PA
52
RpL3-PA
100
67
Dyak GE26079-PA
Dere GG17978-PA
63
Dana GF16740-PA
97
Dper GL12566-PA
48
Dpse GA18487-PA
61
Dvir GJ23255-PA
35
Dgri GH14923-PA
36
Dmoj GI23698-PA
24
Dwil GK14309-PA
0.05

family2419

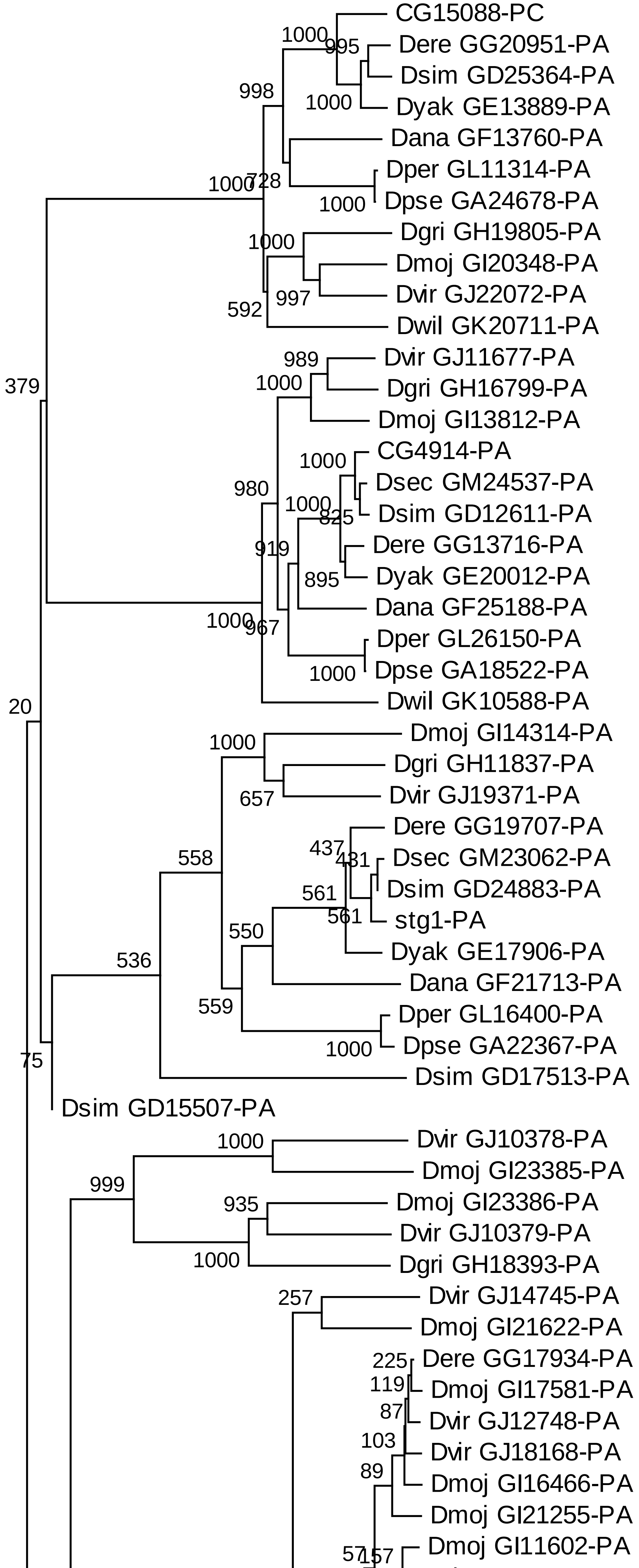
CG15088-PC
Dere GG20951-PA
Dsim GD25364-PA
Dyak GE13889-PA
Dana GF13760-PA
Dper GL11314-PA
Dpse GA24678-PA
Dgri GH19805-PA
Dmoj GI20348-PA
Dvir GJ22072-PA
Dwil GK20711-PA
Dvir GJ11677-PA
Dgri GH16799-PA
Dmoj GI13812-PA
CG4914-PA
Dsec GM24537-PA
Dsim GD12611-PA
Dere GG13716-PA
Dyak GE20012-PA
Dana GF25188-PA
Dper GL26150-PA
Dpse GA18522-PA
Dwil GK10588-PA
Dmoj GI14314-PA
Dgri GH11837-PA
Dvir GJ19371-PA
Dere GG19707-PA
Dsec GM23062-PA
Dsim GD24883-PA
stg1-PA
Dyak GE17906-PA
Dana GF21713-PA
Dper GL16400-PA
Dpse GA22367-PA
Dsim GD17513-PA
Dsim GD15507-PA
Dvir GJ10378-PA
Dmoj GI23385-PA
Dmoj GI23386-PA
Dvir GJ10379-PA
Dgri GH18393-PA
Dvir GJ14745-PA
Dmoj GI21622-PA
Dere GG17934-PA
Dmoj GI17581-PA
Dvir GJ12748-PA
Dvir GJ18168-PA
Dmoj GI16466-PA
Dmoj GI21255-PA
Dmoj GI11602-PA

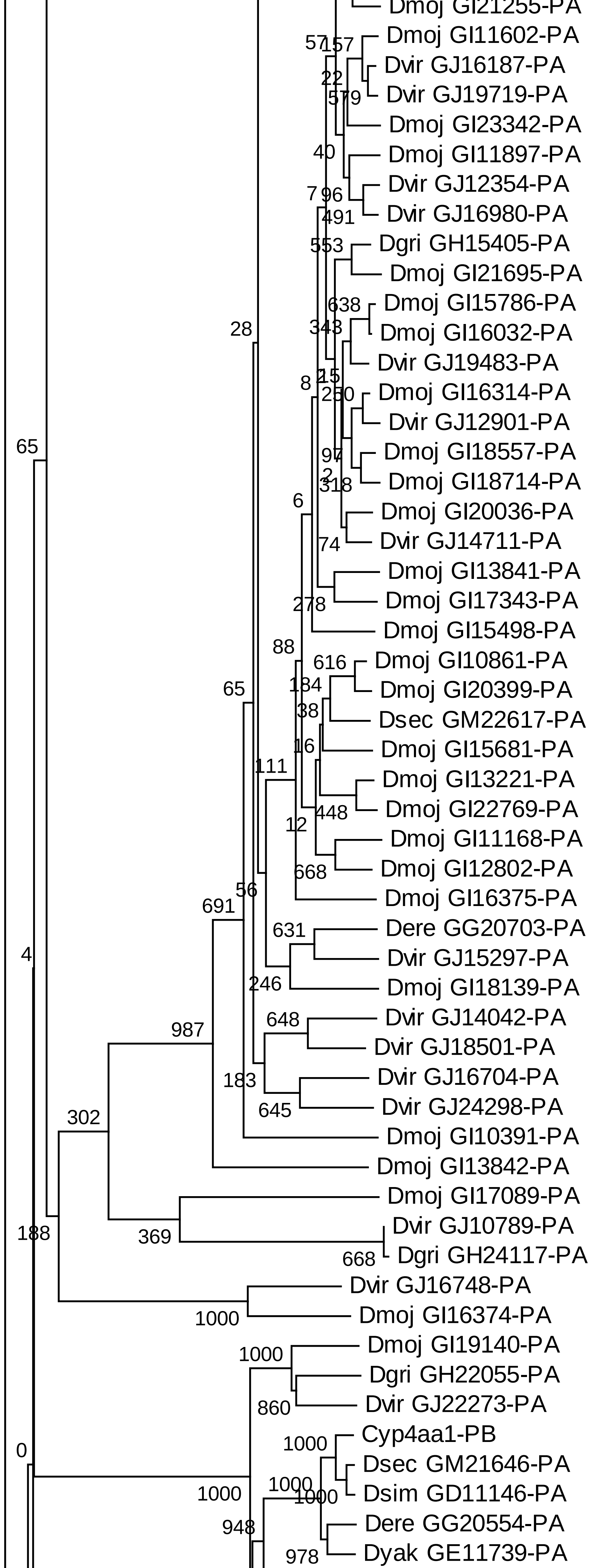

Dmoj GI21255-PA
Dmoj GI11602-PA
Dvir GJ16187-PA
Dvir GJ19719-PA
Dmoj GI23342-PA
Dmoj GI11897-PA
Dvir GJ12354-PA
Dvir GJ16980-PA
Dgri GH15405-PA
Dmoj GI21695-PA
Dmoj GI15786-PA
Dmoj GI16032-PA
Dvir GJ19483-PA
Dmoj GI16314-PA
Dvir GJ12901-PA
Dmoj GI18557-PA
Dmoj GI18714-PA
Dmoj GI20036-PA
Dvir GJ14711-PA
Dmoj GI13841-PA
Dmoj GI17343-PA
Dmoj GI15498-PA
Dmoj GI10861-PA
Dmoj GI20399-PA
Dsec GM22617-PA
Dmoj GI15681-PA
Dmoj GI13221-PA
Dmoj GI22769-PA
Dmoj GI11168-PA
Dmoj GI12802-PA
Dmoj GI16375-PA
Dere GG20703-PA
Dvir GJ15297-PA
Dmoj GI18139-PA
Dvir GJ14042-PA
Dvir GJ18501-PA
Dvir GJ16704-PA
Dvir GJ24298-PA
Dmoj GI10391-PA
Dmoj GI13842-PA
Dmoj GI17089-PA
Dvir GJ10789-PA
Dgri GH24117-PA
Dvir GJ16748-PA
Dmoj GI16374-PA
Dmoj GI19140-PA
Dgri GH22055-PA
Dvir GJ22273-PA
Cyp4aa1-PB
Dsec GM21646-PA
Dsim GD11146-PA
Dere GG20554-PA
Dyak GE11739-PA

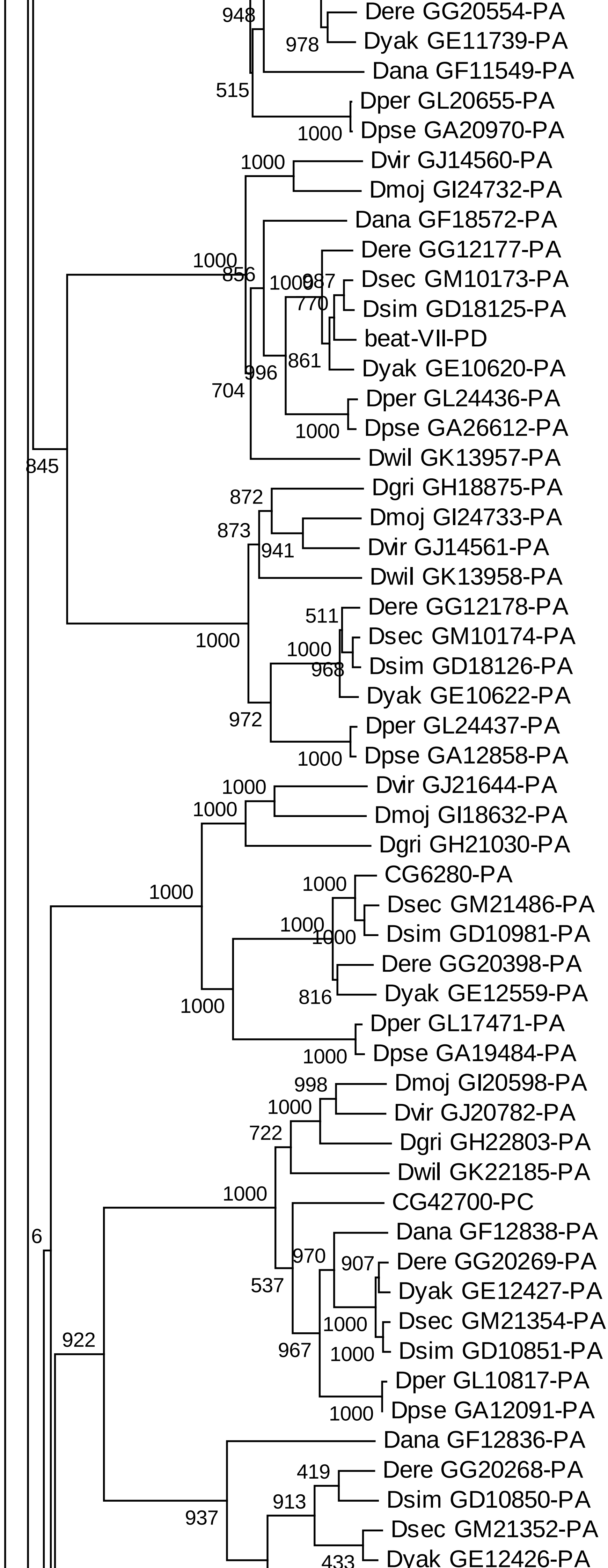
Dere GG20554-PA
Dyak GE11739-PA
Dana GF11549-PA
Dper GL20655-PA
Dpse GA20970-PA
Dvir GJ14560-PA
Dmoj GI24732-PA
Dana GF18572-PA
Dere GG12177-PA
Dsec GM10173-PA
Dsim GD18125-PA
beat-VII-PD
Dyak GE10620-PA
Dper GL24436-PA
Dpse GA26612-PA
Dwil GK13957-PA
Dgri GH18875-PA
Dmoj GI24733-PA
Dvir GJ14561-PA
Dwil GK13958-PA
Dere GG12178-PA
Dsec GM10174-PA
Dsim GD18126-PA
Dyak GE10622-PA
Dper GL24437-PA
Dpse GA12858-PA
Dvir GJ21644-PA
Dmoj GI18632-PA
Dgri GH21030-PA
CG6280-PA
Dsec GM21486-PA
Dsim GD10981-PA
Dere GG20398-PA
Dyak GE12559-PA
Dper GL17471-PA
Dpse GA19484-PA
Dmoj GI20598-PA
Dvir GJ20782-PA
Dgri GH22803-PA
Dwil GK22185-PA
CG42700-PC
Dana GF12838-PA
Dere GG20269-PA
Dyak GE12427-PA
Dsec GM21354-PA
Dsim GD10851-PA
Dper GL10817-PA
Dpse GA12091-PA
Dana GF12836-PA
Dere GG20268-PA
Dsim GD10850-PA
Dsec GM21352-PA
Dyak GE12426-PA

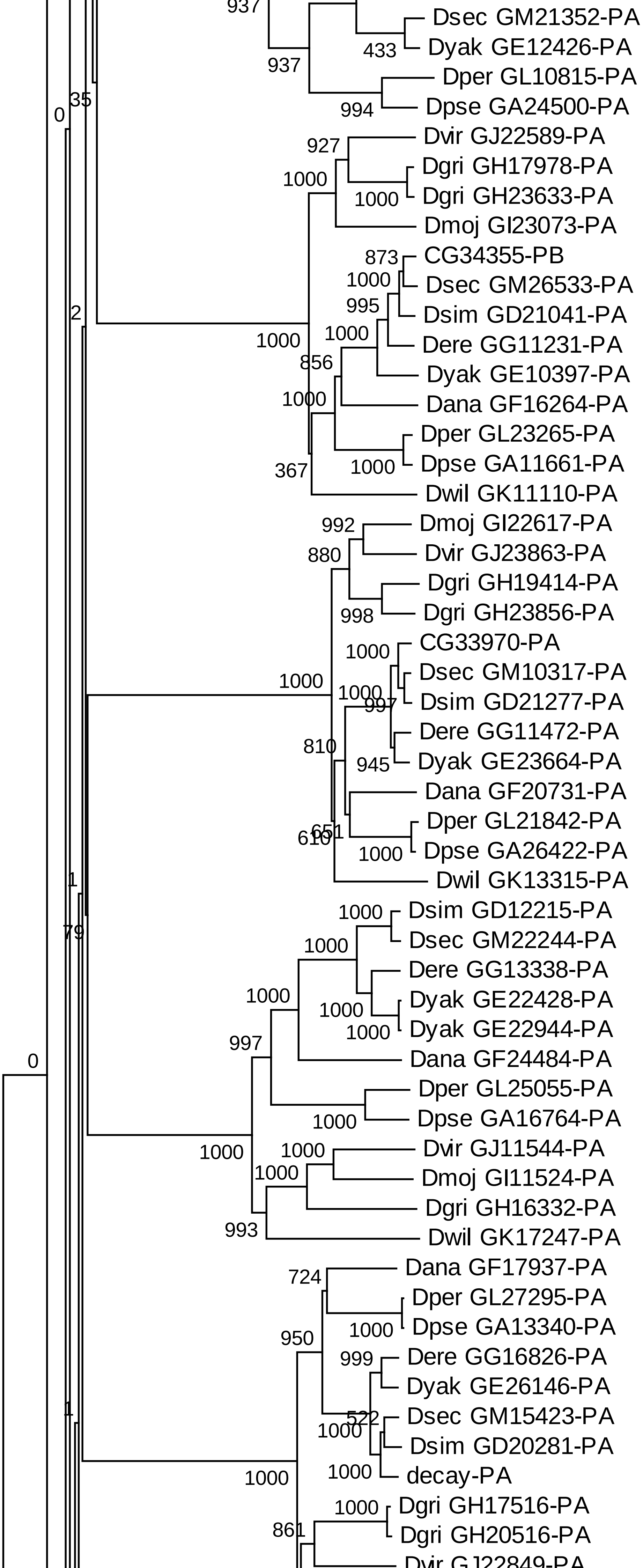

Dsec GM21352-PA
Dyak GE12426-PA
Dper GL10815-PA
Dpse GA24500-PA
Dvir GJ22589-PA
Dgri GH17978-PA
Dgri GH23633-PA
Dmoj GI23073-PA
CG34355-PB
Dsec GM26533-PA
Dsim GD21041-PA
Dere GG11231-PA
Dyak GE10397-PA
Dana GF16264-PA
Dper GL23265-PA
Dpse GA11661-PA
Dwil GK11110-PA
Dmoj GI22617-PA
Dvir GJ23863-PA
Dgri GH19414-PA
Dgri GH23856-PA
CG33970-PA
Dsec GM10317-PA
Dsim GD21277-PA
Dere GG11472-PA
Dyak GE23664-PA
Dana GF20731-PA
Dper GL21842-PA
Dpse GA26422-PA
Dwil GK13315-PA
Dsim GD12215-PA
Dsec GM22244-PA
Dere GG13338-PA
Dyak GE22428-PA
Dyak GE22944-PA
Dana GF24484-PA
Dper GL25055-PA
Dpse GA16764-PA
Dvir GJ11544-PA
Dmoj GI11524-PA
Dgri GH16332-PA
Dwil GK17247-PA
Dana GF17937-PA
Dper GL27295-PA
Dpse GA13340-PA
Dere GG16826-PA
Dyak GE26146-PA
Dsec GM15423-PA
Dsim GD20281-PA
decay-PA
Dgri GH17516-PA
Dgri GH20516-PA

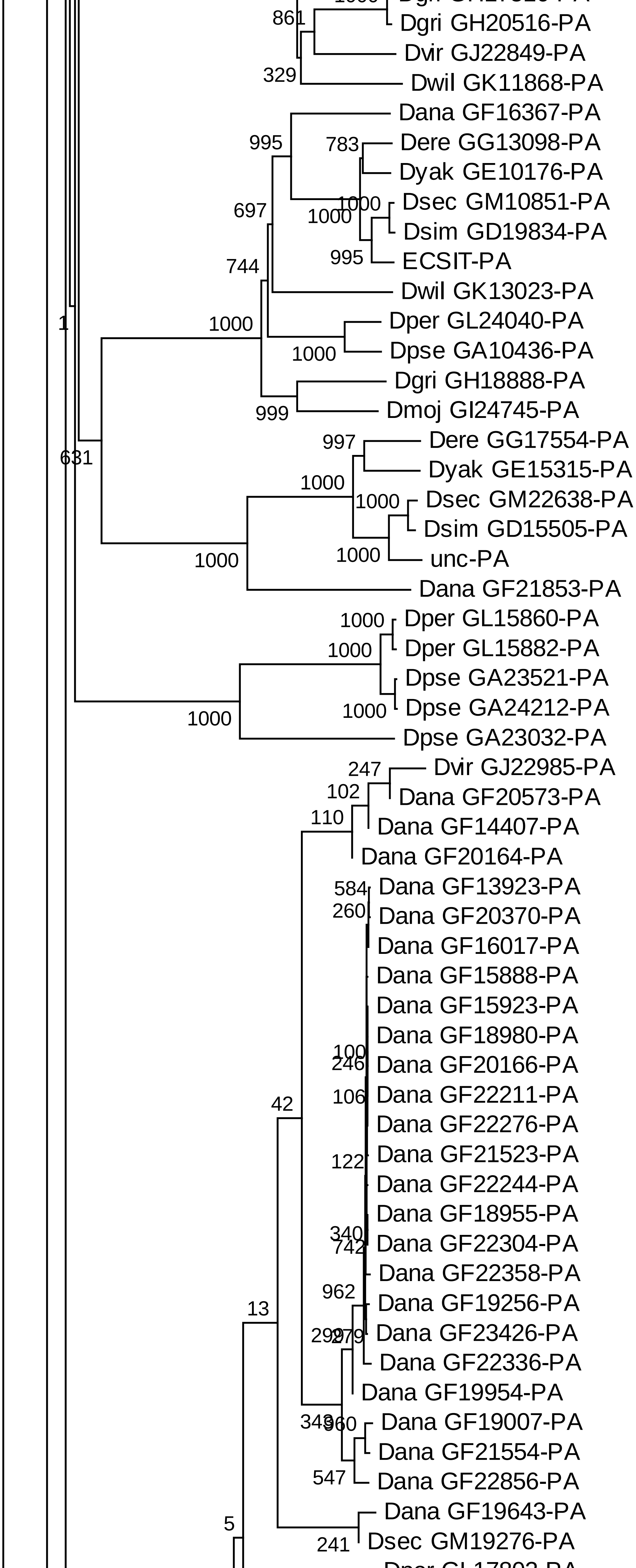
Dgri GH20516-PA
Dvir GJ22849-PA
Dwil GK11868-PA
Dana GF16367-PA
Dere GG13098-PA
Dyak GE10176-PA
Dsec GM10851-PA
Dsim GD19834-PA
ECSIT-PA
Dwil GK13023-PA
Dper GL24040-PA
Dpse GA10436-PA
Dgri GH18888-PA
Dmoj GI24745-PA
Dere GG17554-PA
Dyak GE15315-PA
Dsec GM22638-PA
Dsim GD15505-PA
unc-PA
Dana GF21853-PA
Dper GL15860-PA
Dper GL15882-PA
Dpse GA23521-PA
Dpse GA24212-PA
Dpse GA23032-PA
Dvir GJ22985-PA
Dana GF20573-PA
Dana GF14407-PA
Dana GF20164-PA
Dana GF13923-PA
Dana GF20370-PA
Dana GF16017-PA
Dana GF15888-PA
Dana GF15923-PA
Dana GF18980-PA
Dana GF20166-PA
Dana GF22211-PA
Dana GF22276-PA
Dana GF21523-PA
Dana GF22244-PA
Dana GF18955-PA
Dana GF22304-PA
Dana GF22358-PA
Dana GF19256-PA
Dana GF23426-PA
Dana GF22336-PA
Dana GF19954-PA
Dana GF19007-PA
Dana GF21554-PA
Dana GF22856-PA
Dana GF19643-PA
Dsec GM19276-PA

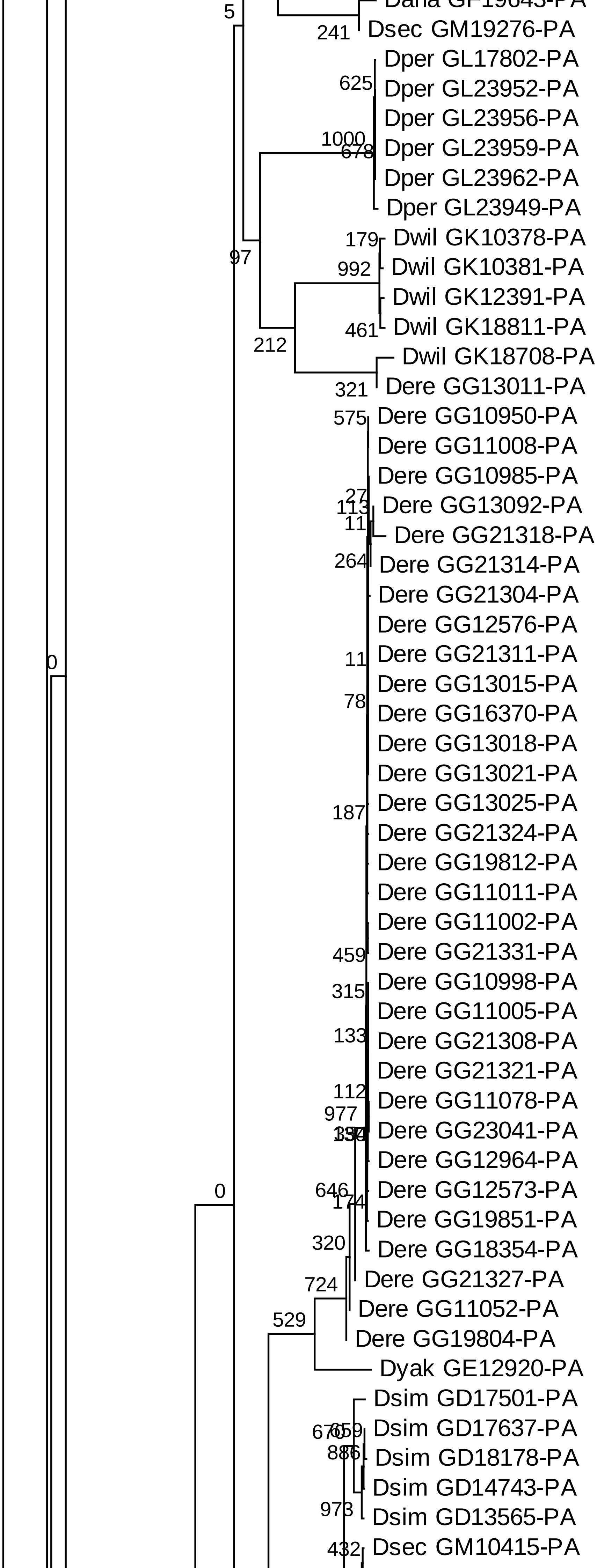

Dsec GM19276-PA
Dper GL17802-PA
Dper GL23952-PA
Dper GL23956-PA
Dper GL23959-PA
Dper GL23962-PA
Dper GL23949-PA
Dwil GK10378-PA
Dwil GK10381-PA
Dwil GK12391-PA
Dwil GK18811-PA
Dwil GK18708-PA
Dere GG13011-PA
Dere GG10950-PA
Dere GG11008-PA
Dere GG10985-PA
Dere GG13092-PA
Dere GG21318-PA
Dere GG21314-PA
Dere GG21304-PA
Dere GG12576-PA
Dere GG21311-PA
Dere GG13015-PA
Dere GG16370-PA
Dere GG13018-PA
Dere GG13021-PA
Dere GG13025-PA
Dere GG21324-PA
Dere GG19812-PA
Dere GG11011-PA
Dere GG11002-PA
Dere GG21331-PA
Dere GG10998-PA
Dere GG11005-PA
Dere GG21308-PA
Dere GG21321-PA
Dere GG11078-PA
Dere GG23041-PA
Dere GG12964-PA
Dere GG12573-PA
Dere GG19851-PA
Dere GG18354-PA
Dere GG21327-PA
Dere GG11052-PA
Dere GG19804-PA
Dyak GE12920-PA
Dsim GD17501-PA
Dsim GD17637-PA
Dsim GD18178-PA
Dsim GD14743-PA
Dsim GD13565-PA
Dsec GM10415-PA
5
241
625
1000
97
179
992
461
212
321
575
264
11
78
187
459
315
133
112
977
0
646
320
724
529
973
432

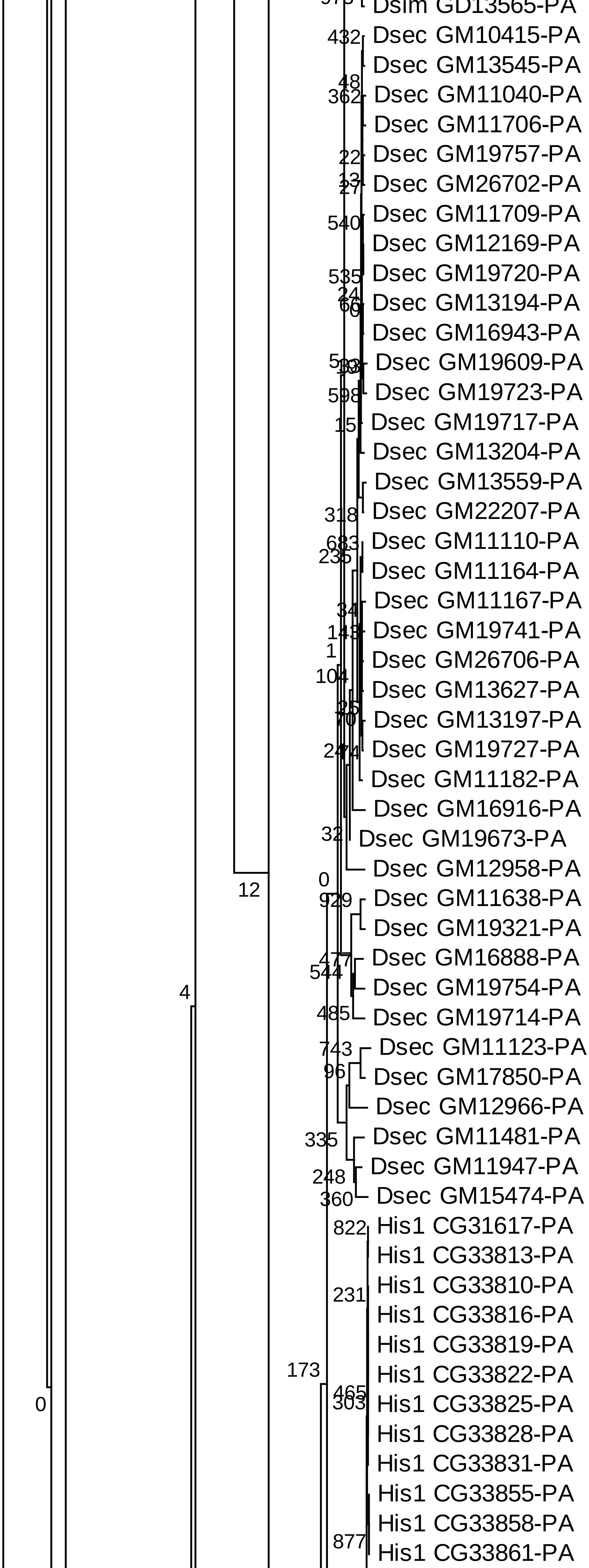
Dsim GD13565-PA
Dsec GM10415-PA
Dsec GM13545-PA
Dsec GM11040-PA
Dsec GM11706-PA
Dsec GM19757-PA
Dsec GM26702-PA
Dsec GM11709-PA
Dsec GM12169-PA
Dsec GM19720-PA
Dsec GM13194-PA
Dsec GM16943-PA
Dsec GM19609-PA
Dsec GM19723-PA
Dsec GM19717-PA
Dsec GM13204-PA
Dsec GM13559-PA
Dsec GM22207-PA
Dsec GM11110-PA
Dsec GM11164-PA
Dsec GM11167-PA
Dsec GM19741-PA
Dsec GM26706-PA
Dsec GM13627-PA
Dsec GM13197-PA
Dsec GM19727-PA
Dsec GM11182-PA
Dsec GM16916-PA
Dsec GM19673-PA
Dsec GM12958-PA
Dsec GM11638-PA
Dsec GM19321-PA
Dsec GM16888-PA
Dsec GM19754-PA
Dsec GM19714-PA
Dsec GM11123-PA
Dsec GM17850-PA
Dsec GM12966-PA
Dsec GM11481-PA
Dsec GM11947-PA
Dsec GM15474-PA
His1 CG31617-PA
His1 CG33813-PA
His1 CG33810-PA
His1 CG33816-PA
His1 CG33819-PA
His1 CG33822-PA
His1 CG33825-PA
His1 CG33828-PA
His1 CG33831-PA
His1 CG33855-PA
His1 CG33858-PA
His1 CG33861-PA

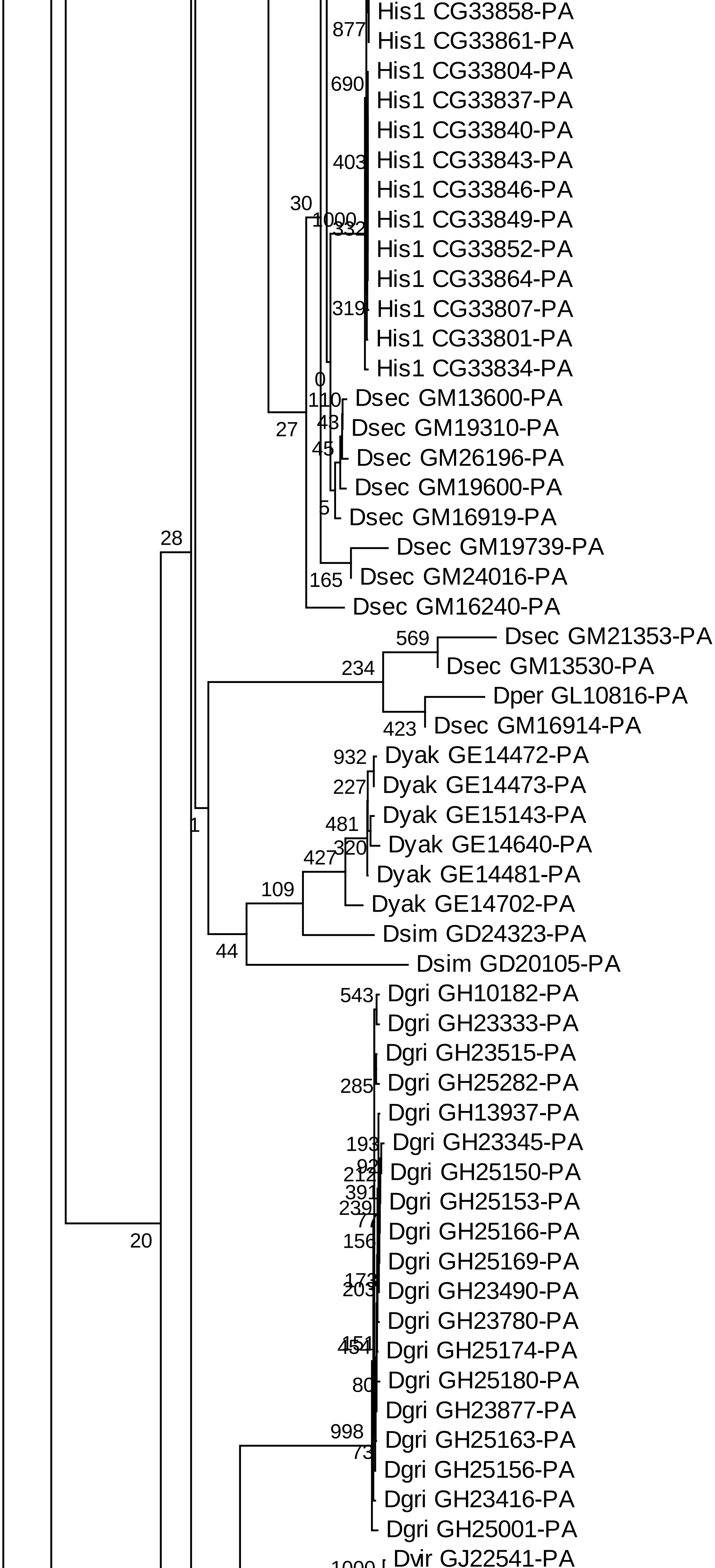

His1 CG33858-PA
877
His1 CG33861-PA
His1 CG33804-PA
690
His1 CG33837-PA
His1 CG33840-PA
403
His1 CG33843-PA
His1 CG33846-PA
30
1000
332
His1 CG33849-PA
His1 CG33852-PA
His1 CG33864-PA
319
His1 CG33807-PA
His1 CG33801-PA
His1 CG33834-PA
0
110
Dsec GM13600-PA
43
Dsec GM19310-PA
27
45
Dsec GM26196-PA
Dsec GM19600-PA
5
Dsec GM16919-PA
28
Dsec GM19739-PA
165
Dsec GM24016-PA
Dsec GM16240-PA
569
Dsec GM21353-PA
234
Dsec GM13530-PA
Dper GL10816-PA
423
Dsec GM16914-PA
932
Dyak GE14472-PA
227
Dyak GE14473-PA
1
481
Dyak GE15143-PA
320
Dyak GE14640-PA
427
Dyak GE14481-PA
109
Dyak GE14702-PA
Dsim GD24323-PA
44
Dsim GD20105-PA
543
Dgri GH10182-PA
Dgri GH23333-PA
Dgri GH23515-PA
285
Dgri GH25282-PA
Dgri GH13937-PA
193
Dgri GH23345-PA
92
212
Dgri GH25150-PA
391
Dgri GH25153-PA
239
77
Dgri GH25166-PA
20
156
Dgri GH25169-PA
173
203
Dgri GH23490-PA
Dgri GH23780-PA
151
454
Dgri GH25174-PA
80
Dgri GH25180-PA
Dgri GH23877-PA
998
Dgri GH25163-PA
73
Dgri GH25156-PA
Dgri GH23416-PA
Dgri GH25001-PA
1000
Dvir GJ22541-PA

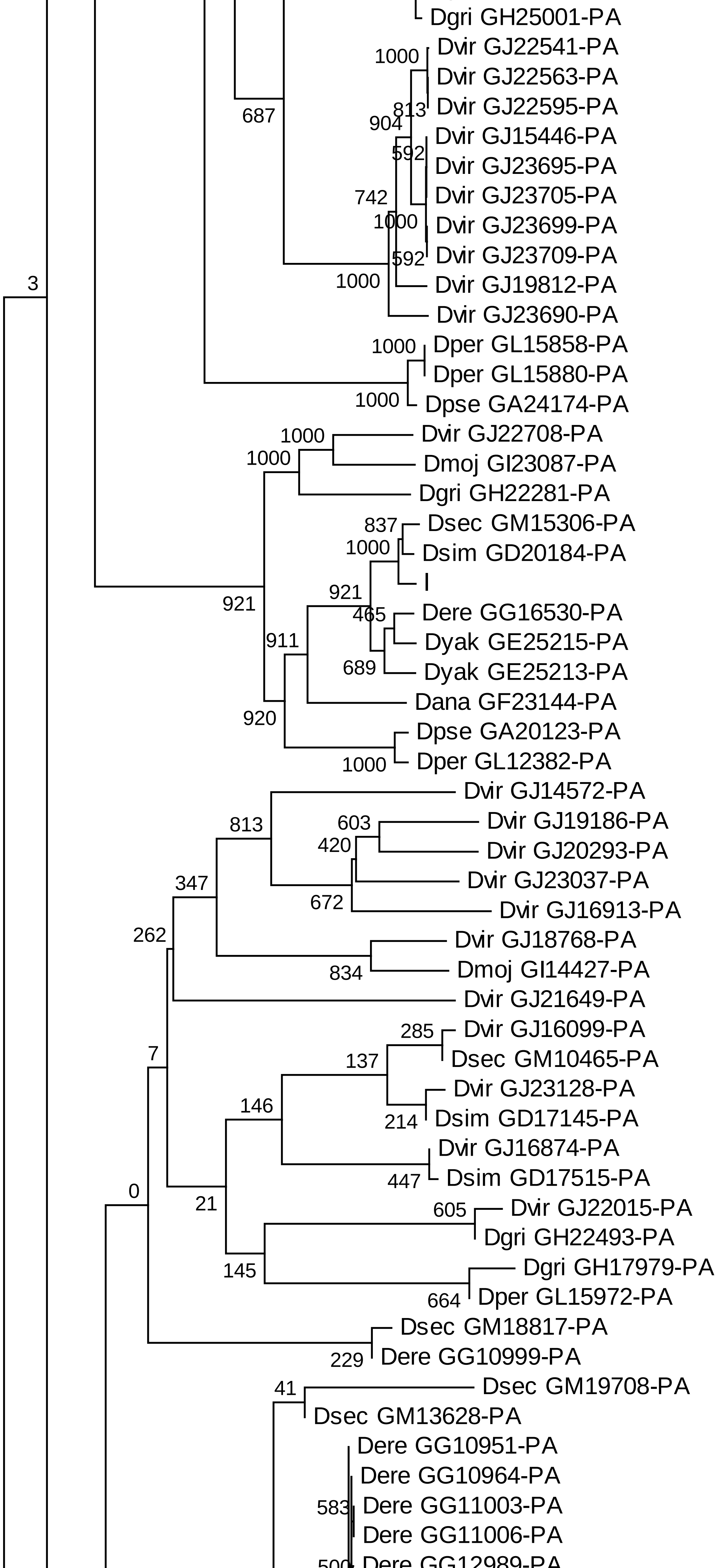

Dgri GH25001-PA
Dvir GJ22541-PA
Dvir GJ22563-PA
Dvir GJ22595-PA
Dvir GJ15446-PA
Dvir GJ23695-PA
Dvir GJ23705-PA
Dvir GJ23699-PA
Dvir GJ23709-PA
Dvir GJ19812-PA
Dvir GJ23690-PA
Dper GL15858-PA
Dper GL15880-PA
Dpse GA24174-PA
Dvir GJ22708-PA
Dmoj GI23087-PA
Dgri GH22281-PA
Dsec GM15306-PA
Dsim GD20184-PA
I
Dere GG16530-PA
Dyak GE25215-PA
Dyak GE25213-PA
Dana GF23144-PA
Dpse GA20123-PA
Dper GL12382-PA
Dvir GJ14572-PA
Dvir GJ19186-PA
Dvir GJ20293-PA
Dvir GJ23037-PA
Dvir GJ16913-PA
Dvir GJ18768-PA
Dmoj GI14427-PA
Dvir GJ21649-PA
Dvir GJ16099-PA
Dsec GM10465-PA
Dvir GJ23128-PA
Dsim GD17145-PA
Dvir GJ16874-PA
Dsim GD17515-PA
Dvir GJ22015-PA
Dgri GH22493-PA
Dgri GH17979-PA
Dper GL15972-PA
Dsec GM18817-PA
Dere GG10999-PA
Dsec GM19708-PA
Dsec GM13628-PA
Dere GG10951-PA
Dere GG10964-PA
Dere GG11003-PA
Dere GG11006-PA
Dere GG12989-PA

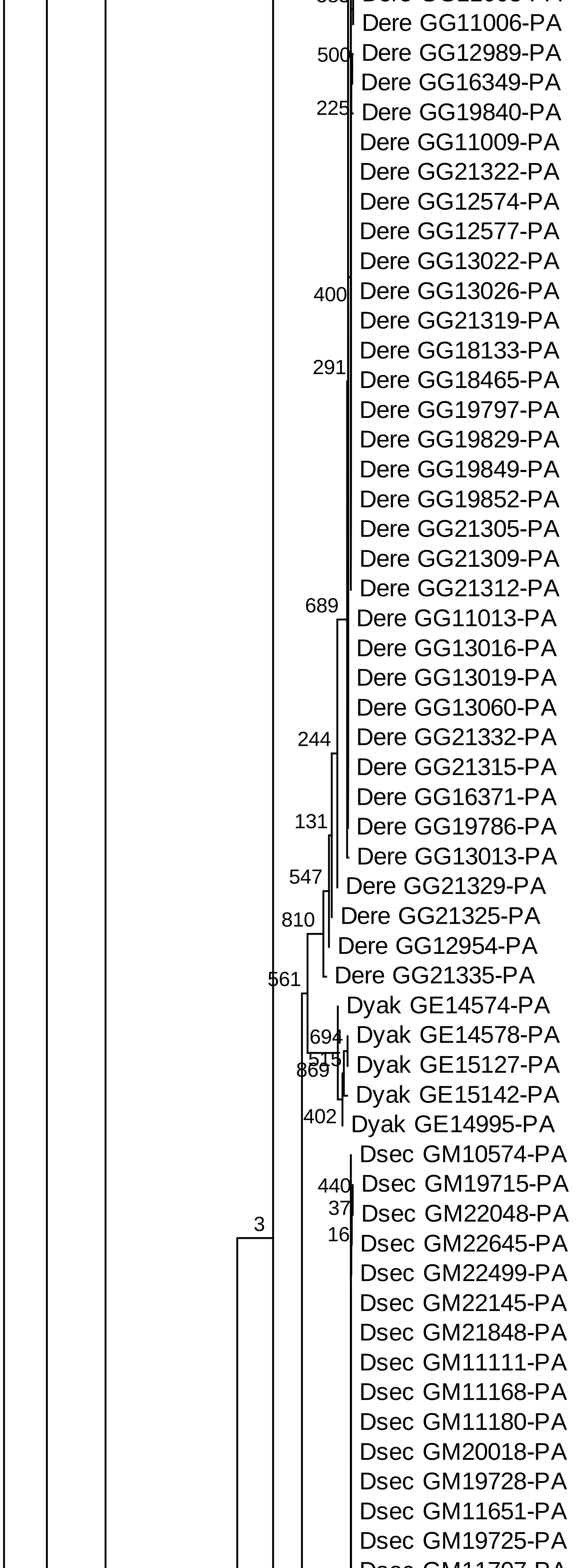
Dere GG11006-PA
500
Dere GG12989-PA
Dere GG16349-PA
225
Dere GG19840-PA
Dere GG11009-PA
Dere GG21322-PA
Dere GG12574-PA
Dere GG12577-PA
Dere GG13022-PA
400
Dere GG13026-PA
Dere GG21319-PA
Dere GG18133-PA
291
Dere GG18465-PA
Dere GG19797-PA
Dere GG19829-PA
Dere GG19849-PA
Dere GG19852-PA
Dere GG21305-PA
Dere GG21309-PA
Dere GG21312-PA
689
Dere GG11013-PA
Dere GG13016-PA
Dere GG13019-PA
Dere GG13060-PA
244
Dere GG21332-PA
Dere GG21315-PA
Dere GG16371-PA
131
Dere GG19786-PA
Dere GG13013-PA
547
Dere GG21329-PA
810
Dere GG21325-PA
Dere GG12954-PA
561
Dere GG21335-PA
Dyak GE14574-PA
694
Dyak GE14578-PA
515
869
Dyak GE15127-PA
Dyak GE15142-PA
402
Dyak GE14995-PA
Dsec GM10574-PA
440
Dsec GM19715-PA
37
Dsec GM22048-PA
3
16
Dsec GM22645-PA
Dsec GM22499-PA
Dsec GM22145-PA
Dsec GM21848-PA
Dsec GM11111-PA
Dsec GM11168-PA
Dsec GM11180-PA
Dsec GM20018-PA
Dsec GM19728-PA
Dsec GM11651-PA
Dsec GM19725-PA

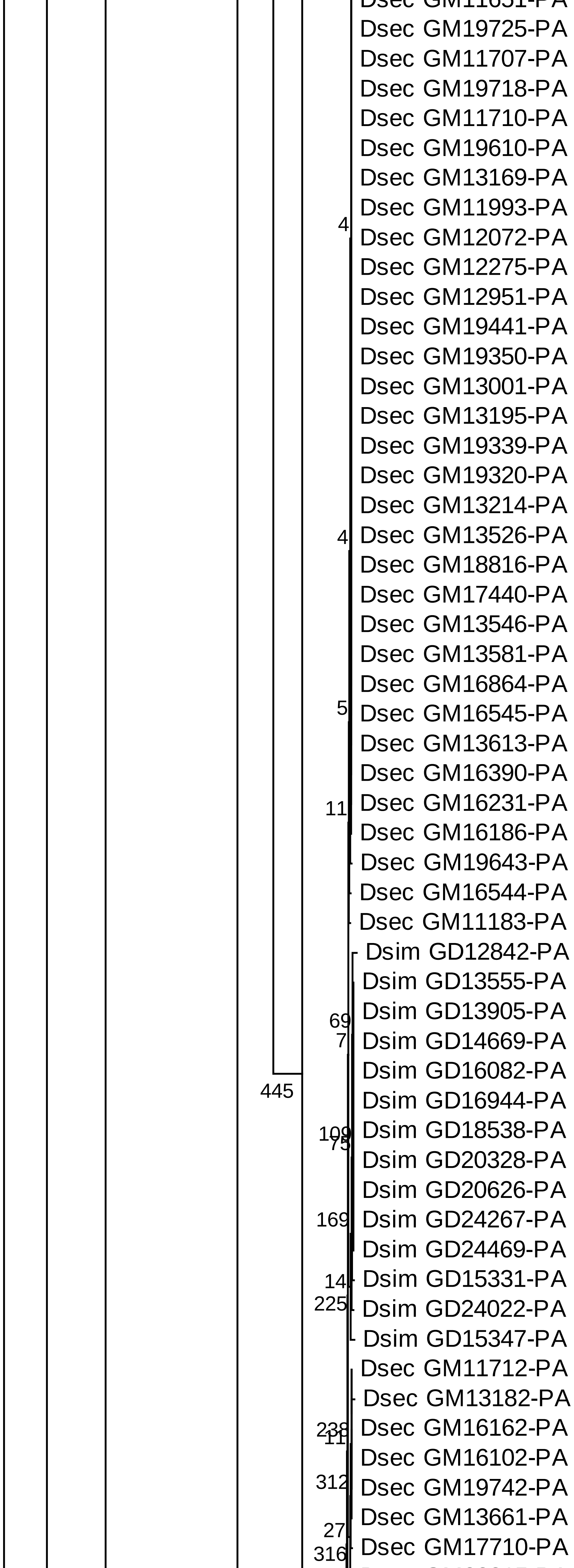
Dsec GM19725-PA
Dsec GM11707-PA
Dsec GM19718-PA
Dsec GM11710-PA
Dsec GM19610-PA
Dsec GM13169-PA
Dsec GM11993-PA
4
Dsec GM12072-PA
Dsec GM12275-PA
Dsec GM12951-PA
Dsec GM19441-PA
Dsec GM19350-PA
Dsec GM13001-PA
Dsec GM13195-PA
Dsec GM19339-PA
Dsec GM19320-PA
Dsec GM13214-PA
4
Dsec GM13526-PA
Dsec GM18816-PA
Dsec GM17440-PA
Dsec GM13546-PA
Dsec GM13581-PA
Dsec GM16864-PA
5
Dsec GM16545-PA
Dsec GM13613-PA
Dsec GM16390-PA
11
Dsec GM16231-PA
Dsec GM16186-PA
Dsec GM19643-PA
Dsec GM16544-PA
Dsec GM11183-PA
Dsim GD12842-PA
Dsim GD13555-PA
Dsim GD13905-PA
69
7
Dsim GD14669-PA
Dsim GD16082-PA
445
Dsim GD16944-PA
109
75
Dsim GD18538-PA
Dsim GD20328-PA
Dsim GD20626-PA
169
Dsim GD24267-PA
Dsim GD24469-PA
14
Dsim GD15331-PA
225
Dsim GD24022-PA
Dsim GD15347-PA
Dsec GM11712-PA
Dsec GM13182-PA
238
11
Dsec GM16162-PA
Dsec GM16102-PA
312
Dsec GM19742-PA
Dsec GM13661-PA
27
Dsec GM17710-PA
316

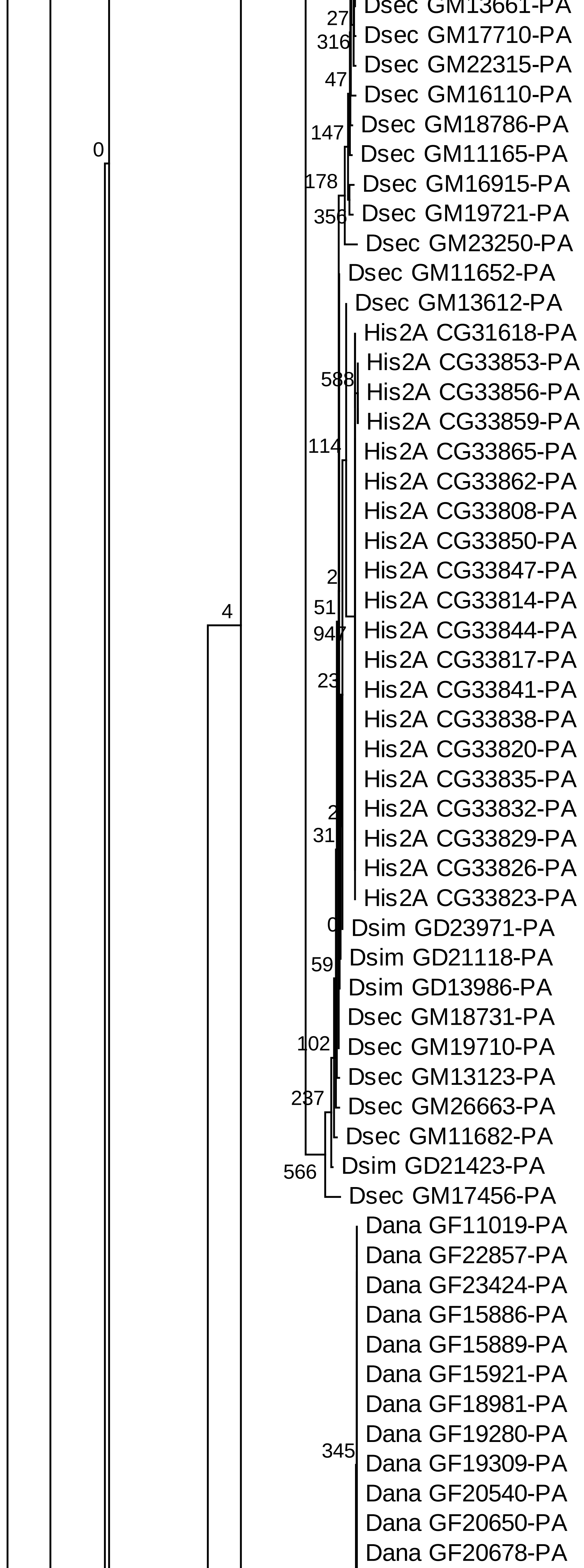

Dsec GM13661-PA
27
316
Dsec GM17710-PA
Dsec GM22315-PA
47
Dsec GM16110-PA
0
147
Dsec GM18786-PA
Dsec GM11165-PA
178
Dsec GM16915-PA
356
Dsec GM19721-PA
Dsec GM23250-PA
Dsec GM11652-PA
Dsec GM13612-PA
His2A CG31618-PA
His2A CG33853-PA
588
His2A CG33856-PA
His2A CG33859-PA
114
His2A CG33865-PA
His2A CG33862-PA
His2A CG33808-PA
His2A CG33850-PA
2
His2A CG33847-PA
4
51
His2A CG33814-PA
947
His2A CG33844-PA
His2A CG33817-PA
23
His2A CG33841-PA
His2A CG33838-PA
His2A CG33820-PA
His2A CG33835-PA
2
His2A CG33832-PA
31
His2A CG33829-PA
His2A CG33826-PA
His2A CG33823-PA
0
Dsim GD23971-PA
59
Dsim GD21118-PA
Dsim GD13986-PA
Dsec GM18731-PA
102
Dsec GM19710-PA
Dsec GM13123-PA
237
Dsec GM26663-PA
Dsec GM11682-PA
Dsim GD21423-PA
566
Dsec GM17456-PA
Dana GF11019-PA
Dana GF22857-PA
Dana GF23424-PA
Dana GF15886-PA
Dana GF15889-PA
Dana GF15921-PA
Dana GF18981-PA
Dana GF19280-PA
345
Dana GF19309-PA
Dana GF20540-PA
Dana GF20650-PA
Dana GF20678-PA

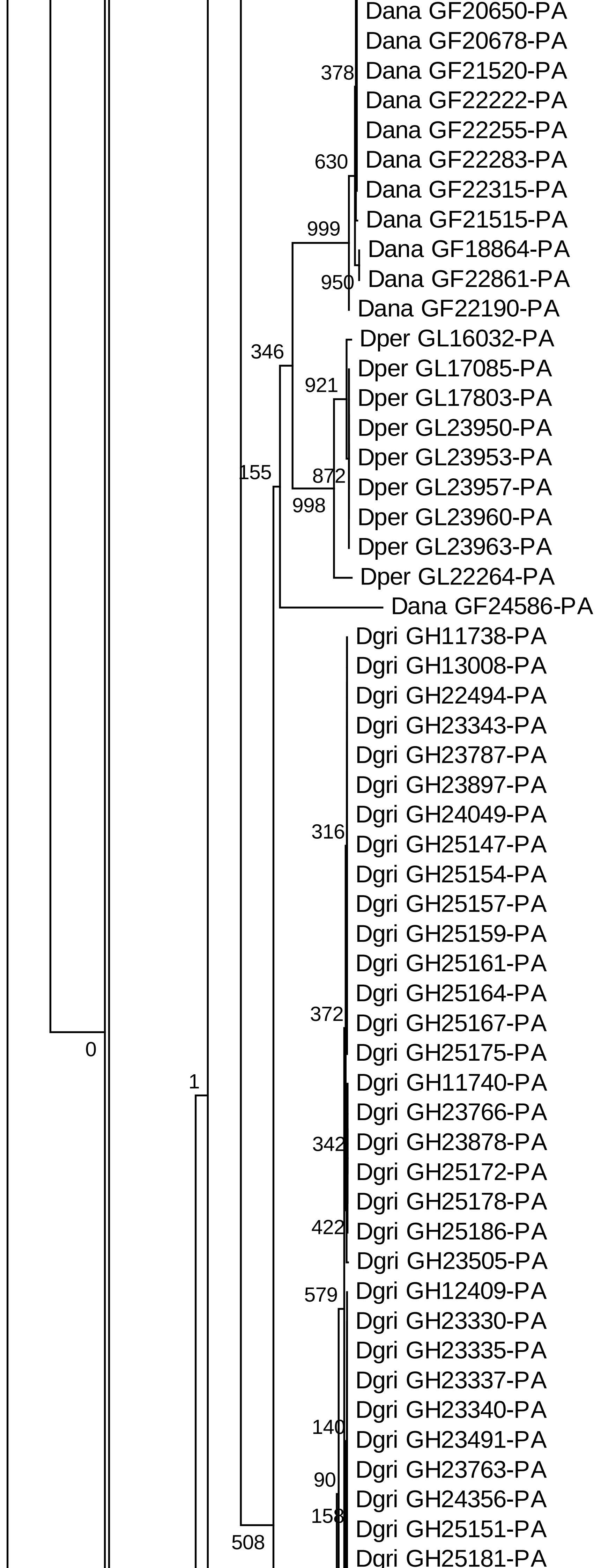
Dana GF20650-PA
Dana GF20678-PA
378
Dana GF21520-PA
Dana GF22222-PA
Dana GF22255-PA
630
Dana GF22283-PA
Dana GF22315-PA
999
Dana GF21515-PA
Dana GF18864-PA
950
Dana GF22861-PA
Dana GF22190-PA
Dper GL16032-PA
346
Dper GL17085-PA
921
Dper GL17803-PA
Dper GL23950-PA
Dper GL23953-PA
155
872
Dper GL23957-PA
998
Dper GL23960-PA
Dper GL23963-PA
Dper GL22264-PA
Dana GF24586-PA
Dgri GH11738-PA
Dgri GH13008-PA
Dgri GH22494-PA
Dgri GH23343-PA
Dgri GH23787-PA
Dgri GH23897-PA
Dgri GH24049-PA
316
Dgri GH25147-PA
Dgri GH25154-PA
Dgri GH25157-PA
Dgri GH25159-PA
Dgri GH25161-PA
Dgri GH25164-PA
372
Dgri GH25167-PA
0
Dgri GH25175-PA
1
Dgri GH11740-PA
Dgri GH23766-PA
342
Dgri GH23878-PA
Dgri GH25172-PA
Dgri GH25178-PA
422
Dgri GH25186-PA
Dgri GH23505-PA
579
Dgri GH12409-PA
Dgri GH23330-PA
Dgri GH23335-PA
Dgri GH23337-PA
Dgri GH23340-PA
140
Dgri GH23491-PA
Dgri GH23763-PA
90
Dgri GH24356-PA
158
Dgri GH25151-PA
508
Dgri GH25181-PA

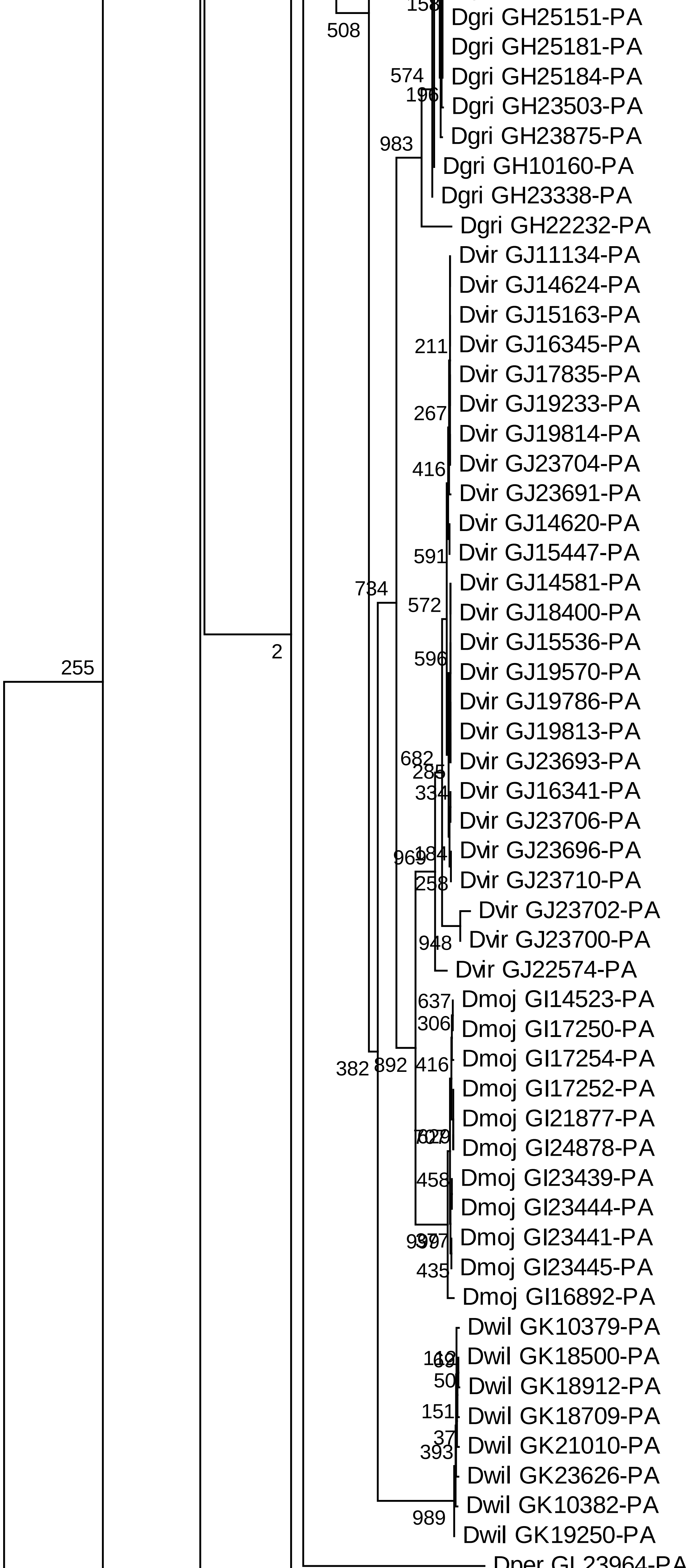

508
Dgri GH25151-PA
Dgri GH25181-PA
574
Dgri GH25184-PA
196
Dgri GH23503-PA
Dgri GH23875-PA
983
Dgri GH10160-PA
Dgri GH23338-PA
Dgri GH22232-PA
Dvir GJ11134-PA
Dvir GJ14624-PA
Dvir GJ15163-PA
211
Dvir GJ16345-PA
Dvir GJ17835-PA
267
Dvir GJ19233-PA
Dvir GJ19814-PA
416
Dvir GJ23704-PA
Dvir GJ23691-PA
Dvir GJ14620-PA
591
Dvir GJ15447-PA
734
Dvir GJ14581-PA
572
Dvir GJ18400-PA
2
Dvir GJ15536-PA
255
596
Dvir GJ19570-PA
Dvir GJ19786-PA
Dvir GJ19813-PA
682
Dvir GJ23693-PA
285
334
Dvir GJ16341-PA
Dvir GJ23706-PA
969
184
Dvir GJ23696-PA
258
Dvir GJ23710-PA
Dvir GJ23702-PA
948
Dvir GJ23700-PA
Dvir GJ22574-PA
637
Dmoj GI14523-PA
306
Dmoj GI17250-PA
382
892
416
Dmoj GI17254-PA
Dmoj GI17252-PA
Dmoj GI21877-PA
Dmoj GI24878-PA
458
Dmoj GI23439-PA
Dmoj GI23444-PA
Dmoj GI23441-PA
435
Dmoj GI23445-PA
Dmoj GI16892-PA
Dwil GK10379-PA
Dwil GK18500-PA
50
Dwil GK18912-PA
151
Dwil GK18709-PA
37
393
Dwil GK21010-PA
Dwil GK23626-PA
Dwil GK10382-PA
989
Dwil GK19250-PA
Dper GL23964-PA

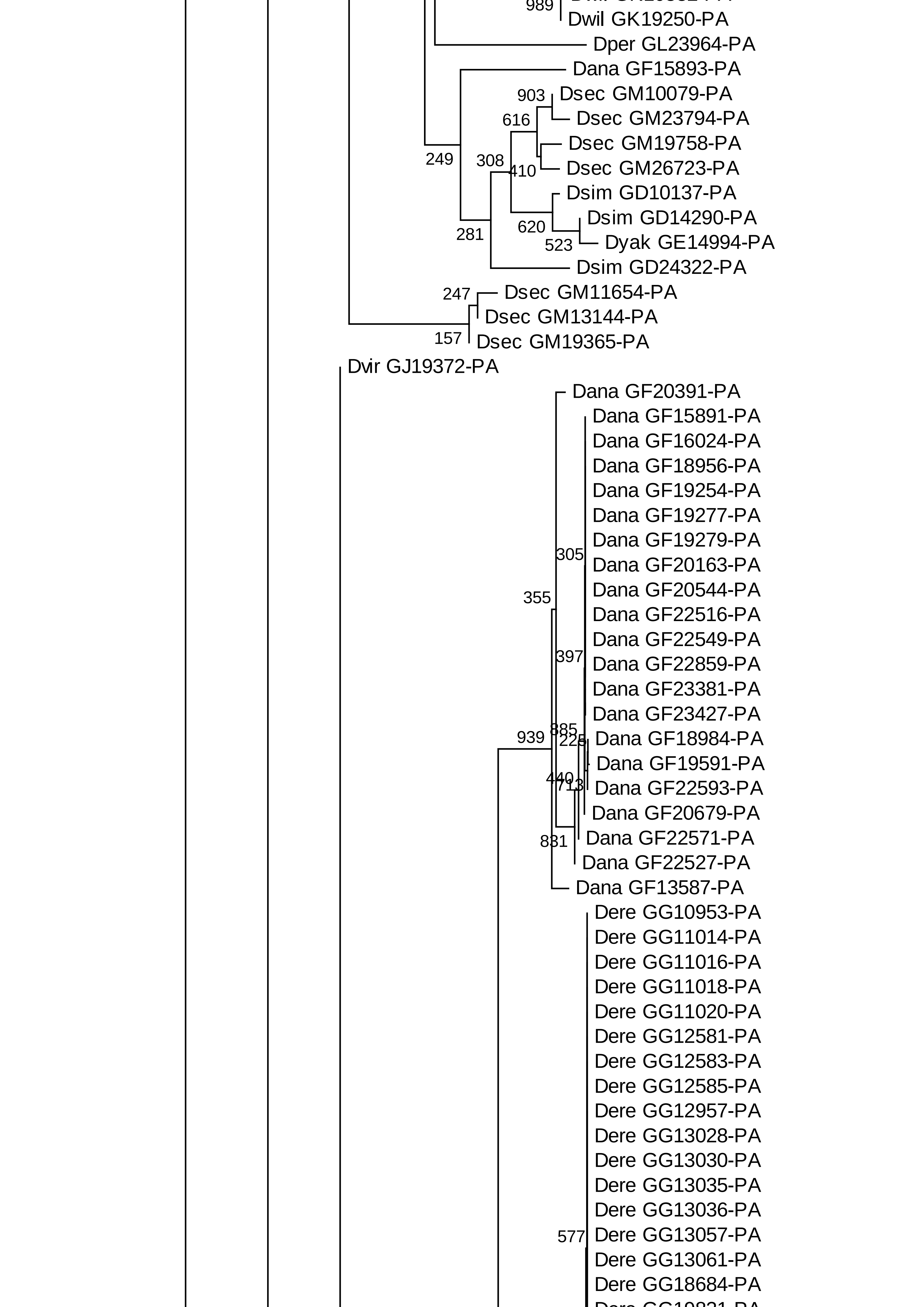
989
Dwil GK19250-PA
Dper GL23964-PA
Dana GF15893-PA
903
Dsec GM10079-PA
616
Dsec GM23794-PA
Dsec GM19758-PA
249
308
410
Dsec GM26723-PA
Dsim GD10137-PA
Dsim GD14290-PA
620
281
523
Dyak GE14994-PA
Dsim GD24322-PA
247
Dsec GM11654-PA
Dsec GM13144-PA
157
Dsec GM19365-PA
Dvir GJ19372-PA
Dana GF20391-PA
Dana GF15891-PA
Dana GF16024-PA
Dana GF18956-PA
Dana GF19254-PA
Dana GF19277-PA
Dana GF19279-PA
305
Dana GF20163-PA
355
Dana GF20544-PA
Dana GF22516-PA
Dana GF22549-PA
397
Dana GF22859-PA
Dana GF23381-PA
Dana GF23427-PA
885
939
Dana GF18984-PA
Dana GF19591-PA
440
713
Dana GF22593-PA
Dana GF20679-PA
Dana GF22571-PA
881
Dana GF22527-PA
Dana GF13587-PA
Dere GG10953-PA
Dere GG11014-PA
Dere GG11016-PA
Dere GG11018-PA
Dere GG11020-PA
Dere GG12581-PA
Dere GG12583-PA
Dere GG12585-PA
Dere GG12957-PA
Dere GG13028-PA
Dere GG13030-PA
Dere GG13035-PA
Dere GG13036-PA
577
Dere GG13057-PA
Dere GG13061-PA
Dere GG18684-PA

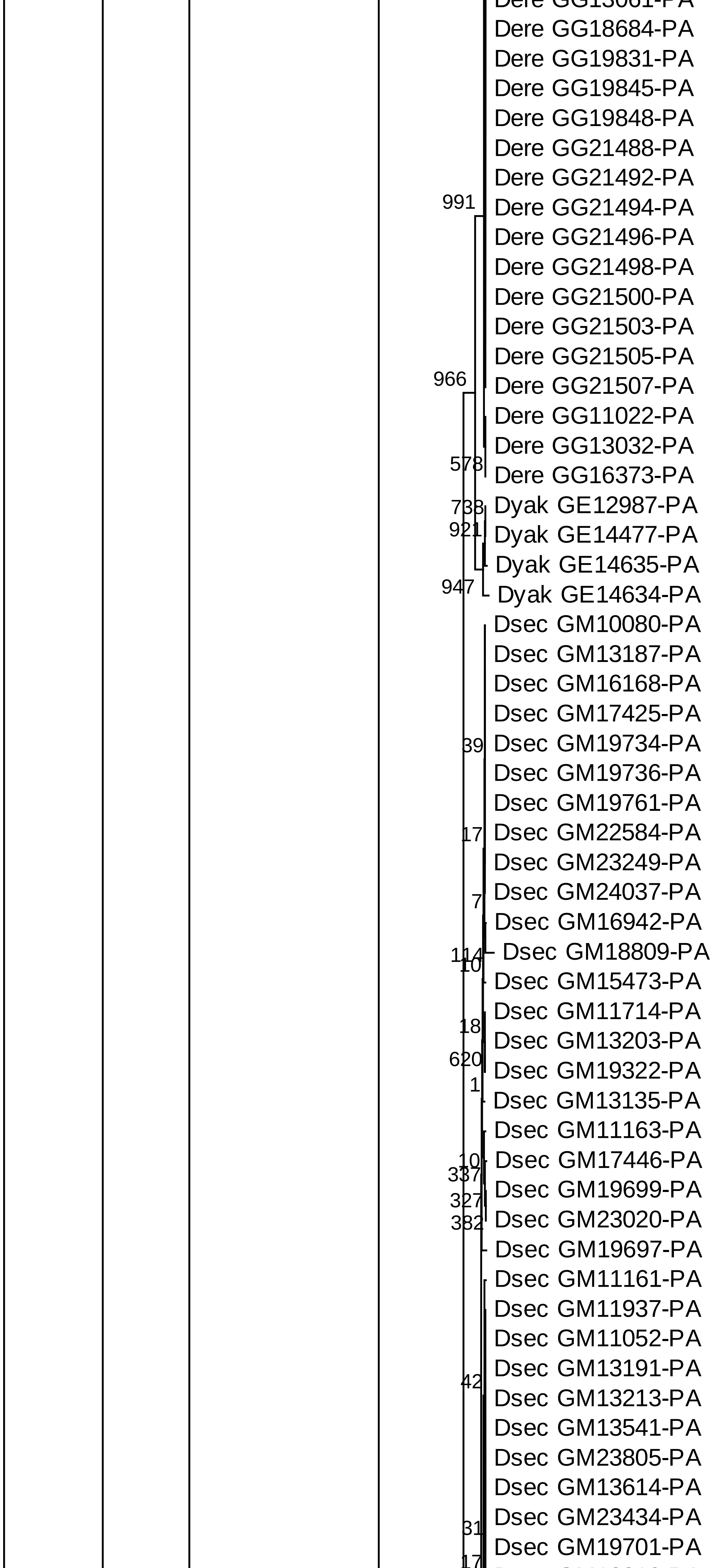

Dere GG13061-PA
Dere GG18684-PA
Dere GG19831-PA
Dere GG19845-PA
Dere GG19848-PA
Dere GG21488-PA
Dere GG21492-PA
991
Dere GG21494-PA
Dere GG21496-PA
Dere GG21498-PA
Dere GG21500-PA
Dere GG21503-PA
Dere GG21505-PA
966
Dere GG21507-PA
Dere GG11022-PA
Dere GG13032-PA
578
Dere GG16373-PA
738
Dyak GE12987-PA
921
Dyak GE14477-PA
Dyak GE14635-PA
947
Dyak GE14634-PA
Dsec GM10080-PA
Dsec GM13187-PA
Dsec GM16168-PA
Dsec GM17425-PA
39
Dsec GM19734-PA
Dsec GM19736-PA
Dsec GM19761-PA
17
Dsec GM22584-PA
Dsec GM23249-PA
Dsec GM24037-PA
7
Dsec GM16942-PA
114
Dsec GM18809-PA
10
Dsec GM15473-PA
Dsec GM11714-PA
18
Dsec GM13203-PA
620
Dsec GM19322-PA
1
Dsec GM13135-PA
Dsec GM11163-PA
10
Dsec GM17446-PA
337
Dsec GM19699-PA
327
382
Dsec GM23020-PA
Dsec GM19697-PA
Dsec GM11161-PA
Dsec GM11937-PA
Dsec GM11052-PA
Dsec GM13191-PA
42
Dsec GM13213-PA
Dsec GM13541-PA
Dsec GM23805-PA
Dsec GM13614-PA
31
Dsec GM23434-PA
Dsec GM19701-PA

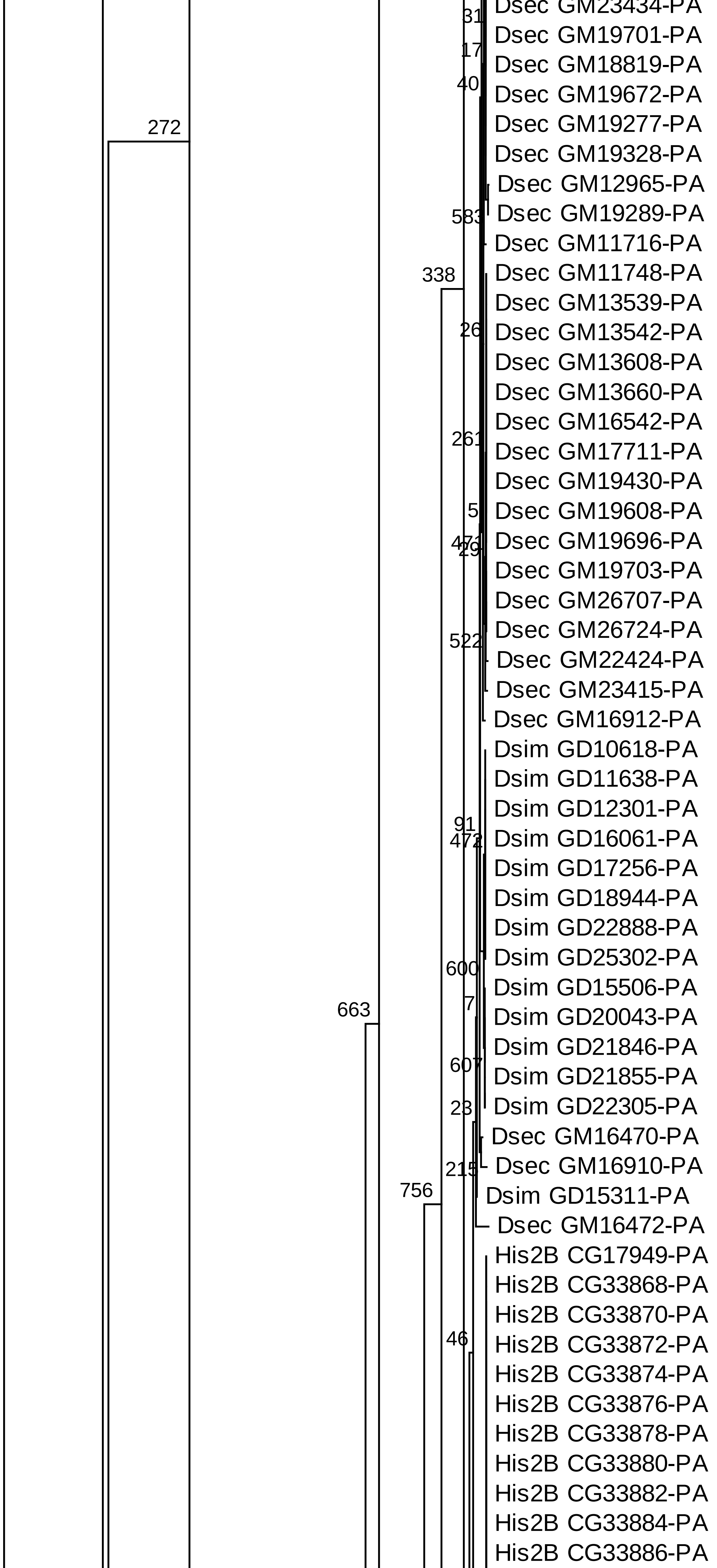

Dsec GM23434-PA
Dsec GM19701-PA
Dsec GM18819-PA
Dsec GM19672-PA
Dsec GM19277-PA
Dsec GM19328-PA
Dsec GM12965-PA
Dsec GM19289-PA
Dsec GM11716-PA
Dsec GM11748-PA
Dsec GM13539-PA
Dsec GM13542-PA
Dsec GM13608-PA
Dsec GM13660-PA
Dsec GM16542-PA
Dsec GM17711-PA
Dsec GM19430-PA
Dsec GM19608-PA
Dsec GM19696-PA
Dsec GM19703-PA
Dsec GM26707-PA
Dsec GM26724-PA
Dsec GM22424-PA
Dsec GM23415-PA
Dsec GM16912-PA
Dsim GD10618-PA
Dsim GD11638-PA
Dsim GD12301-PA
Dsim GD16061-PA
Dsim GD17256-PA
Dsim GD18944-PA
Dsim GD22888-PA
Dsim GD25302-PA
Dsim GD15506-PA
Dsim GD20043-PA
Dsim GD21846-PA
Dsim GD21855-PA
Dsim GD22305-PA
Dsec GM16470-PA
Dsec GM16910-PA
Dsim GD15311-PA
Dsec GM16472-PA
His2B CG17949-PA
His2B CG33868-PA
His2B CG33870-PA
His2B CG33872-PA
His2B CG33874-PA
His2B CG33876-PA
His2B CG33878-PA
His2B CG33880-PA
His2B CG33882-PA
His2B CG33884-PA
His2B CG33886-PA
272
338
663
756
17
40
583
26
261
5
522
91
600
7
607
23
215
46

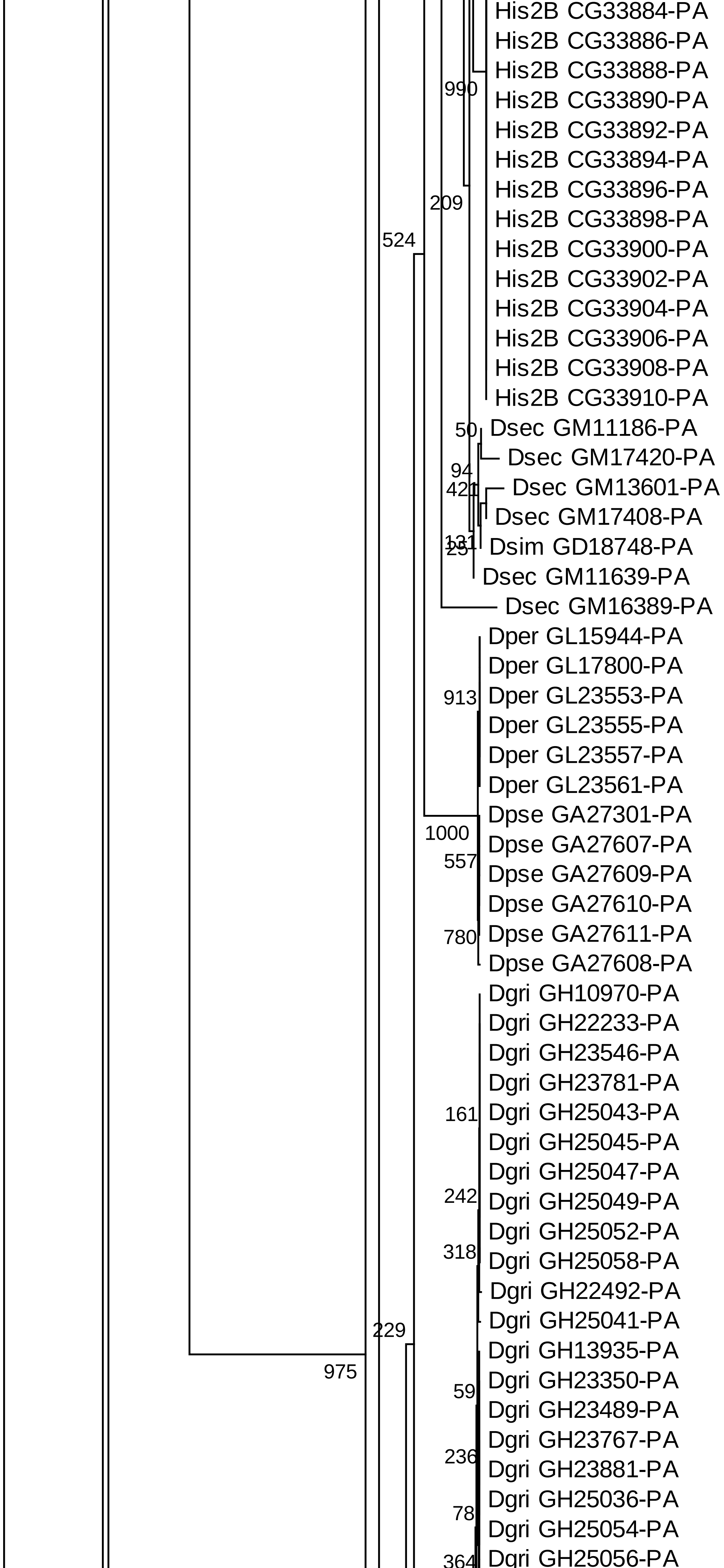
His2B CG33884-PA
His2B CG33886-PA
His2B CG33888-PA
990
His2B CG33890-PA
His2B CG33892-PA
His2B CG33894-PA
His2B CG33896-PA
209
His2B CG33898-PA
524
His2B CG33900-PA
His2B CG33902-PA
His2B CG33904-PA
His2B CG33906-PA
His2B CG33908-PA
His2B CG33910-PA
50
Dsec GM11186-PA
Dsec GM17420-PA
94
421
Dsec GM13601-PA
Dsec GM17408-PA
131
25
Dsim GD18748-PA
Dsec GM11639-PA
Dsec GM16389-PA
Dper GL15944-PA
Dper GL17800-PA
913
Dper GL23553-PA
Dper GL23555-PA
Dper GL23557-PA
Dper GL23561-PA
Dpse GA27301-PA
1000
Dpse GA27607-PA
557
Dpse GA27609-PA
Dpse GA27610-PA
780
Dpse GA27611-PA
Dpse GA27608-PA
Dgri GH10970-PA
Dgri GH22233-PA
Dgri GH23546-PA
Dgri GH23781-PA
161
Dgri GH25043-PA
Dgri GH25045-PA
Dgri GH25047-PA
242
Dgri GH25049-PA
Dgri GH25052-PA
318
Dgri GH25058-PA
Dgri GH22492-PA
Dgri GH25041-PA
229
Dgri GH13935-PA
975
Dgri GH23350-PA
59
Dgri GH23489-PA
Dgri GH23767-PA
236
Dgri GH23881-PA
Dgri GH25036-PA
78
Dgri GH25054-PA
364
Dgri GH25056-PA

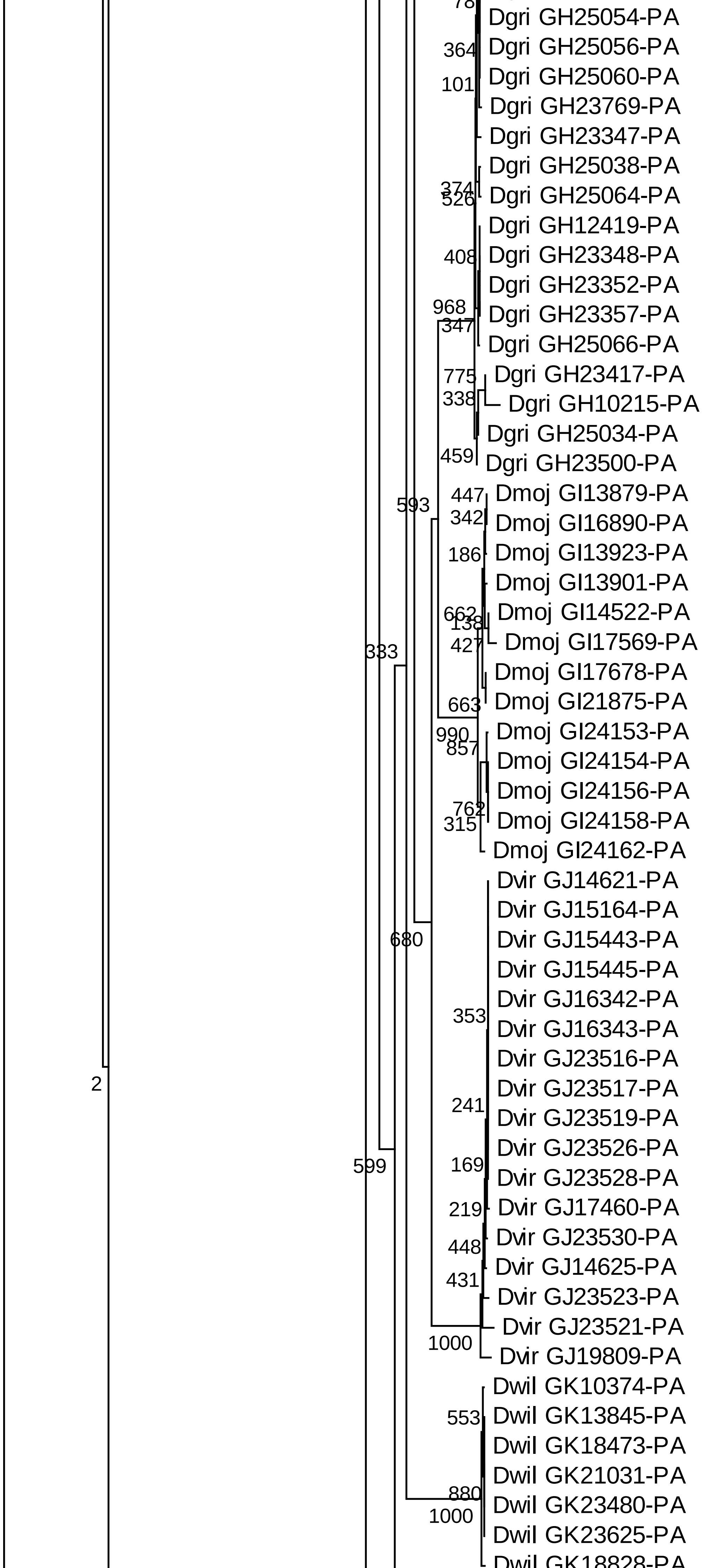

78
Dgri GH25054-PA
364
Dgri GH25056-PA
101
Dgri GH25060-PA
Dgri GH23769-PA
Dgri GH23347-PA
Dgri GH25038-PA
374
526
Dgri GH25064-PA
Dgri GH12419-PA
408
Dgri GH23348-PA
Dgri GH23352-PA
968
Dgri GH23357-PA
347
Dgri GH25066-PA
775
Dgri GH23417-PA
338
Dgri GH10215-PA
Dgri GH25034-PA
459
Dgri GH23500-PA
447
Dmoj GI13879-PA
593
342
Dmoj GI16890-PA
186
Dmoj GI13923-PA
Dmoj GI13901-PA
662
Dmoj GI14522-PA
138
427
Dmoj GI17569-PA
333
Dmoj GI17678-PA
663
Dmoj GI21875-PA
990
Dmoj GI24153-PA
857
Dmoj GI24154-PA
Dmoj GI24156-PA
762
Dmoj GI24158-PA
315
Dmoj GI24162-PA
Dvir GJ14621-PA
Dvir GJ15164-PA
680
Dvir GJ15443-PA
Dvir GJ15445-PA
Dvir GJ16342-PA
353
Dvir GJ16343-PA
Dvir GJ23516-PA
2
Dvir GJ23517-PA
241
Dvir GJ23519-PA
Dvir GJ23526-PA
599
169
Dvir GJ23528-PA
219
Dvir GJ17460-PA
448
Dvir GJ23530-PA
Dvir GJ14625-PA
431
Dvir GJ23523-PA
Dvir GJ23521-PA
1000
Dvir GJ19809-PA
Dwil GK10374-PA
553
Dwil GK13845-PA
Dwil GK18473-PA
Dwil GK21031-PA
880
Dwil GK23480-PA
1000
Dwil GK23625-PA
Dwil GK18828-PA

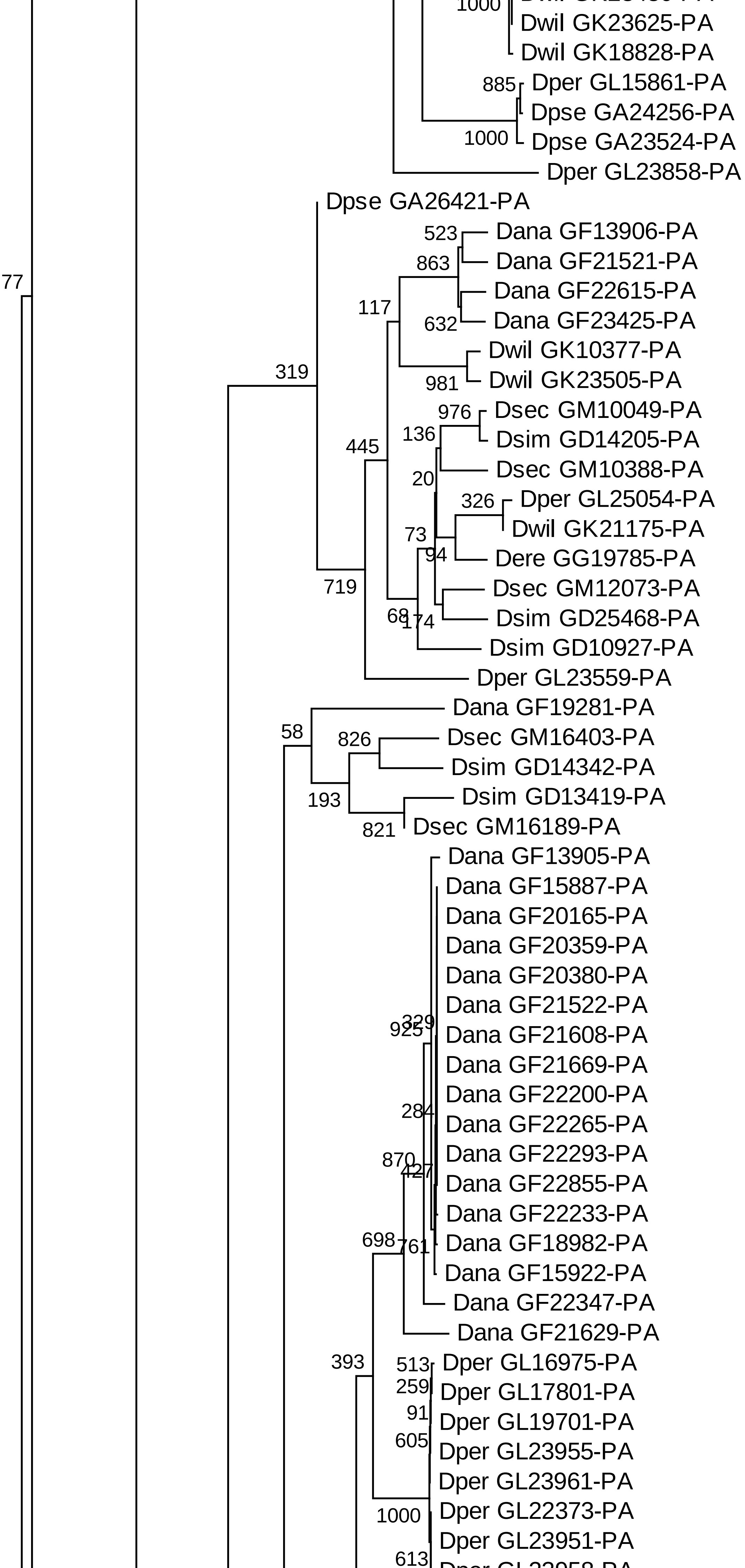
Dwil GK23625-PA
Dwil GK18828-PA
Dper GL15861-PA
Dpse GA24256-PA
Dpse GA23524-PA
Dper GL23858-PA
Dpse GA26421-PA
Dana GF13906-PA
Dana GF21521-PA
Dana GF22615-PA
Dana GF23425-PA
Dwil GK10377-PA
Dwil GK23505-PA
Dsec GM10049-PA
Dsim GD14205-PA
Dsec GM10388-PA
Dper GL25054-PA
Dwil GK21175-PA
Dere GG19785-PA
Dsec GM12073-PA
Dsim GD25468-PA
Dsim GD10927-PA
Dper GL23559-PA
Dana GF19281-PA
Dsec GM16403-PA
Dsim GD14342-PA
Dsim GD13419-PA
Dsec GM16189-PA
Dana GF13905-PA
Dana GF15887-PA
Dana GF20165-PA
Dana GF20359-PA
Dana GF20380-PA
Dana GF21522-PA
Dana GF21608-PA
Dana GF21669-PA
Dana GF22200-PA
Dana GF22265-PA
Dana GF22293-PA
Dana GF22855-PA
Dana GF22233-PA
Dana GF18982-PA
Dana GF15922-PA
Dana GF22347-PA
Dana GF21629-PA
Dper GL16975-PA
Dper GL17801-PA
Dper GL19701-PA
Dper GL23955-PA
Dper GL23961-PA
Dper GL22373-PA
Dper GL23951-PA

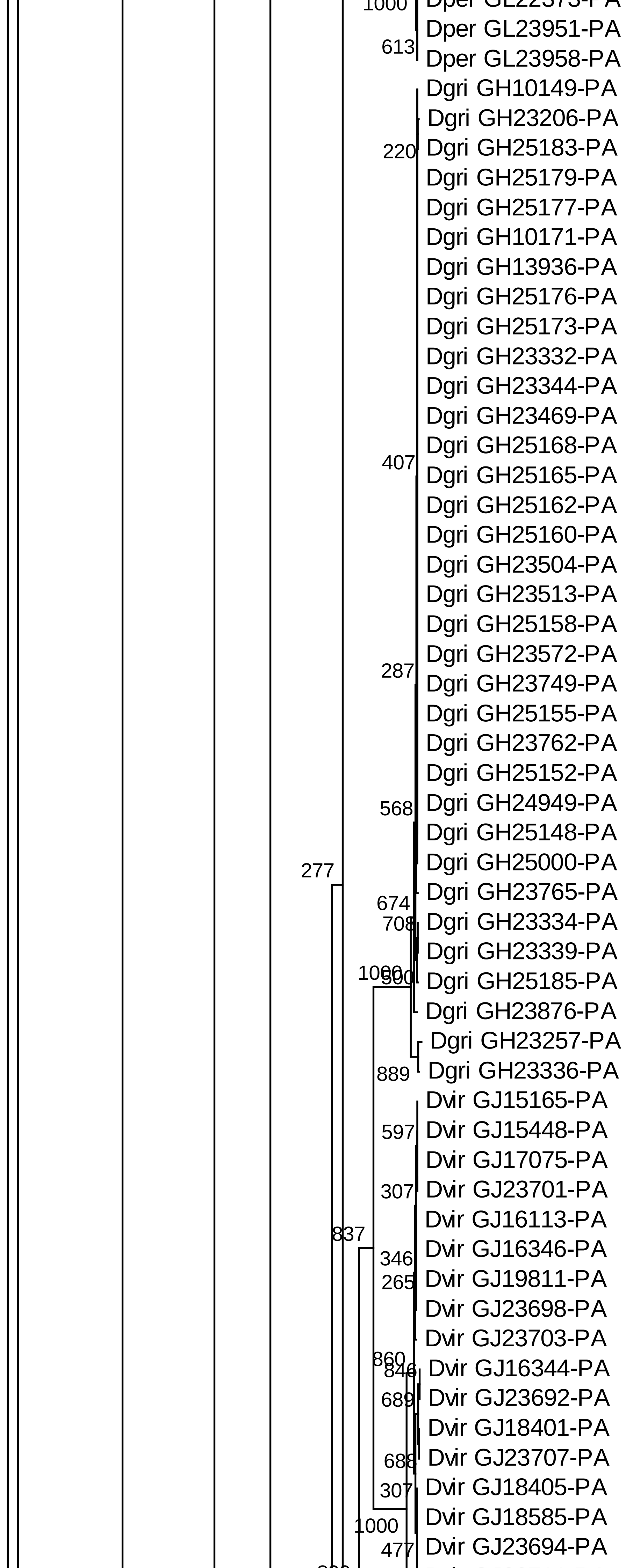
1000
Dper GL23951-PA
613
Dper GL23958-PA
Dgri GH10149-PA
Dgri GH23206-PA
220
Dgri GH25183-PA
Dgri GH25179-PA
Dgri GH25177-PA
Dgri GH10171-PA
Dgri GH13936-PA
Dgri GH25176-PA
Dgri GH25173-PA
Dgri GH23332-PA
Dgri GH23344-PA
Dgri GH23469-PA
Dgri GH25168-PA
407
Dgri GH25165-PA
Dgri GH25162-PA
Dgri GH25160-PA
Dgri GH23504-PA
Dgri GH23513-PA
Dgri GH25158-PA
Dgri GH23572-PA
287
Dgri GH23749-PA
Dgri GH25155-PA
Dgri GH23762-PA
Dgri GH25152-PA
Dgri GH24949-PA
568
Dgri GH25148-PA
Dgri GH25000-PA
277
Dgri GH23765-PA
674
708
Dgri GH23334-PA
Dgri GH23339-PA
1000
900
Dgri GH25185-PA
Dgri GH23876-PA
Dgri GH23257-PA
889
Dgri GH23336-PA
Dvir GJ15165-PA
597
Dvir GJ15448-PA
Dvir GJ17075-PA
307
Dvir GJ23701-PA
Dvir GJ16113-PA
837
346
Dvir GJ16346-PA
265
Dvir GJ19811-PA
Dvir GJ23698-PA
Dvir GJ23703-PA
860
846
Dvir GJ16344-PA
689
Dvir GJ23692-PA
Dvir GJ18401-PA
688
Dvir GJ23707-PA
307
Dvir GJ18405-PA
1000
Dvir GJ18585-PA
477
Dvir GJ23694-PA

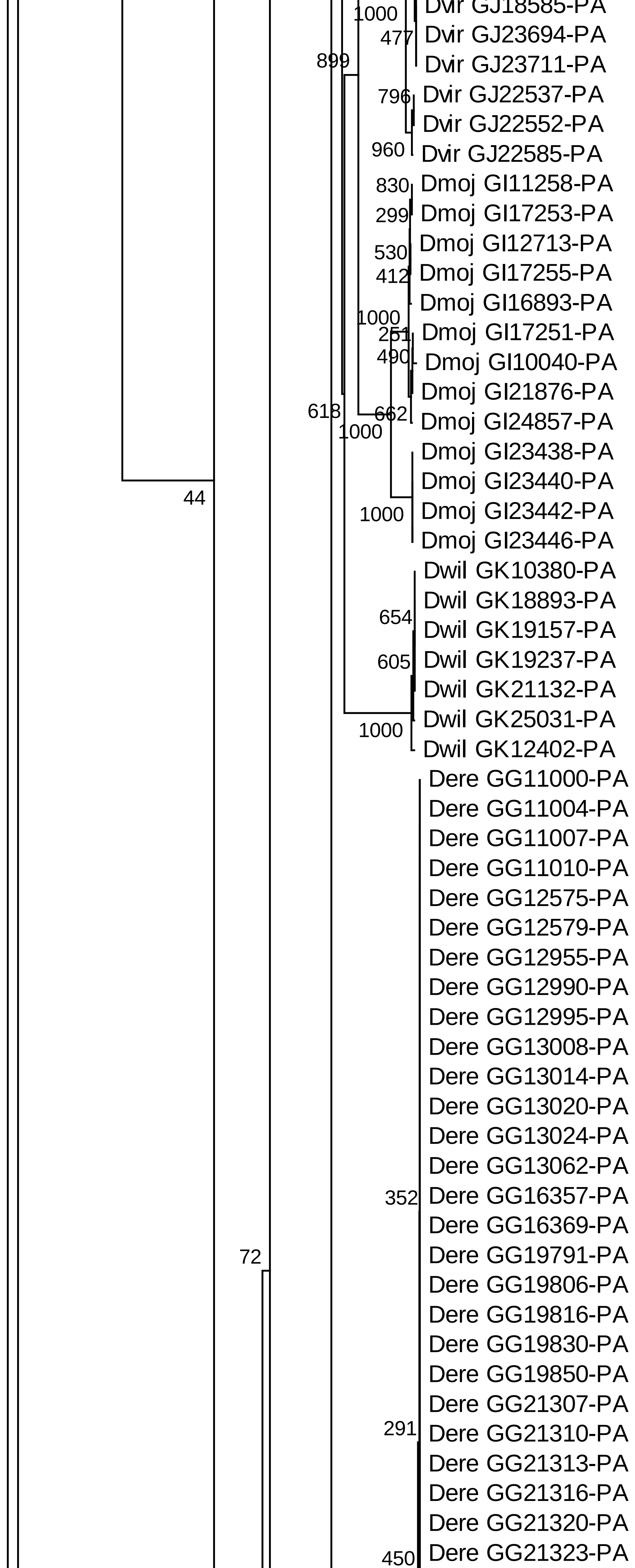
Dvir GJ18585-PA
Dvir GJ23694-PA
Dvir GJ23711-PA
Dvir GJ22537-PA
Dvir GJ22552-PA
Dvir GJ22585-PA
Dmoj GI11258-PA
Dmoj GI17253-PA
Dmoj GI12713-PA
Dmoj GI17255-PA
Dmoj GI16893-PA
Dmoj GI17251-PA
Dmoj GI10040-PA
Dmoj GI21876-PA
Dmoj GI24857-PA
Dmoj GI23438-PA
Dmoj GI23440-PA
Dmoj GI23442-PA
Dmoj GI23446-PA
Dwil GK10380-PA
Dwil GK18893-PA
Dwil GK19157-PA
Dwil GK19237-PA
Dwil GK21132-PA
Dwil GK25031-PA
Dwil GK12402-PA
Dere GG11000-PA
Dere GG11004-PA
Dere GG11007-PA
Dere GG11010-PA
Dere GG12575-PA
Dere GG12579-PA
Dere GG12955-PA
Dere GG12990-PA
Dere GG12995-PA
Dere GG13008-PA
Dere GG13014-PA
Dere GG13020-PA
Dere GG13024-PA
Dere GG13062-PA
Dere GG16357-PA
Dere GG16369-PA
Dere GG19791-PA
Dere GG19806-PA
Dere GG19816-PA
Dere GG19830-PA
Dere GG19850-PA
Dere GG21307-PA
Dere GG21310-PA
Dere GG21313-PA
Dere GG21316-PA
Dere GG21320-PA
Dere GG21323-PA
1000
477
899
796
960
830
299
530
412
1000
251
490
618
662
1000
44
1000
654
605
1000
352
72
291
450

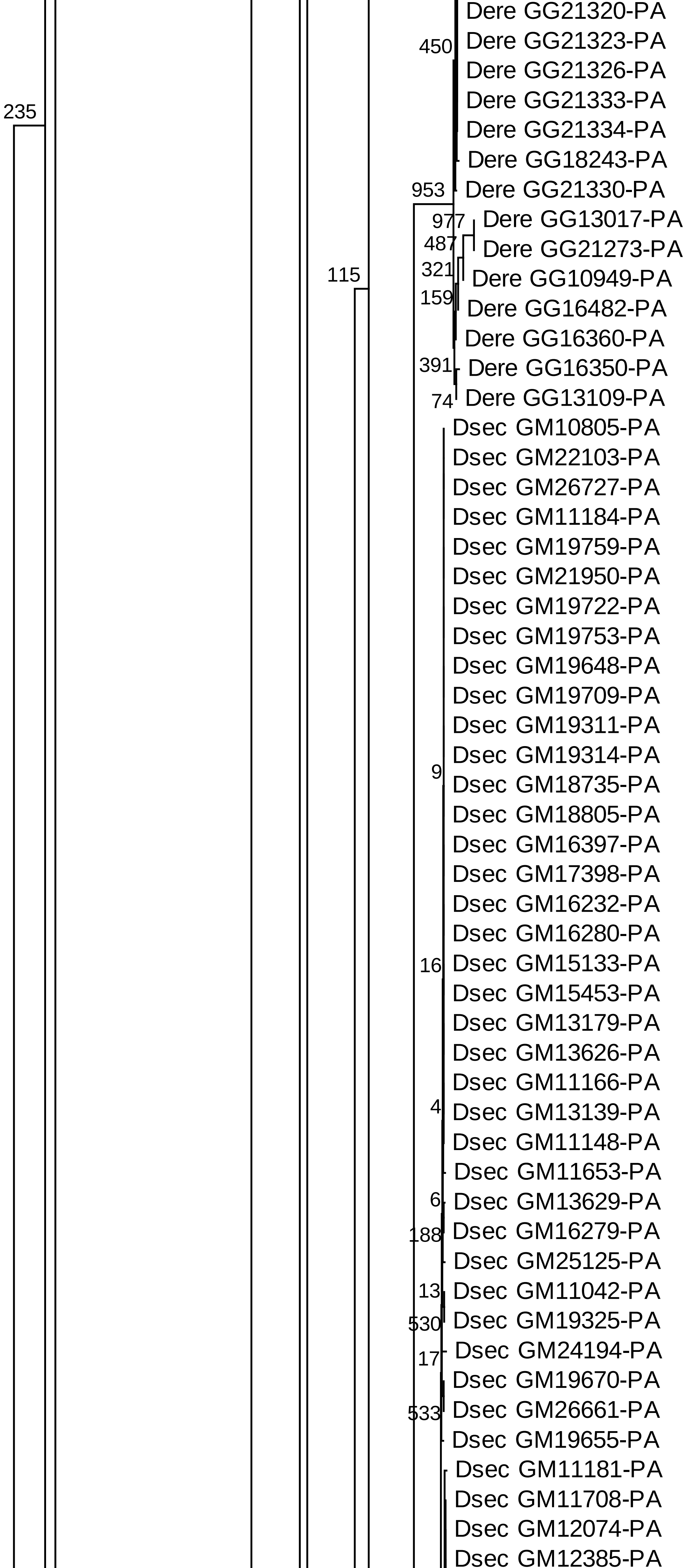

Dere GG21320-PA
450
Dere GG21323-PA
Dere GG21326-PA
235
Dere GG21333-PA
Dere GG21334-PA
Dere GG18243-PA
953
Dere GG21330-PA
977
Dere GG13017-PA
487
Dere GG21273-PA
321
115
Dere GG10949-PA
159
Dere GG16482-PA
Dere GG16360-PA
391
Dere GG16350-PA
74
Dere GG13109-PA
Dsec GM10805-PA
Dsec GM22103-PA
Dsec GM26727-PA
Dsec GM11184-PA
Dsec GM19759-PA
Dsec GM21950-PA
Dsec GM19722-PA
Dsec GM19753-PA
Dsec GM19648-PA
Dsec GM19709-PA
Dsec GM19311-PA
Dsec GM19314-PA
9
Dsec GM18735-PA
Dsec GM18805-PA
Dsec GM16397-PA
Dsec GM17398-PA
Dsec GM16232-PA
Dsec GM16280-PA
16
Dsec GM15133-PA
Dsec GM15453-PA
Dsec GM13179-PA
Dsec GM13626-PA
Dsec GM11166-PA
4
Dsec GM13139-PA
Dsec GM11148-PA
Dsec GM11653-PA
6
Dsec GM13629-PA
188
Dsec GM16279-PA
Dsec GM25125-PA
13
Dsec GM11042-PA
530
Dsec GM19325-PA
Dsec GM24194-PA
17
Dsec GM19670-PA
533
Dsec GM26661-PA
Dsec GM19655-PA
Dsec GM11181-PA
Dsec GM11708-PA
Dsec GM12074-PA
Dsec GM12385-PA

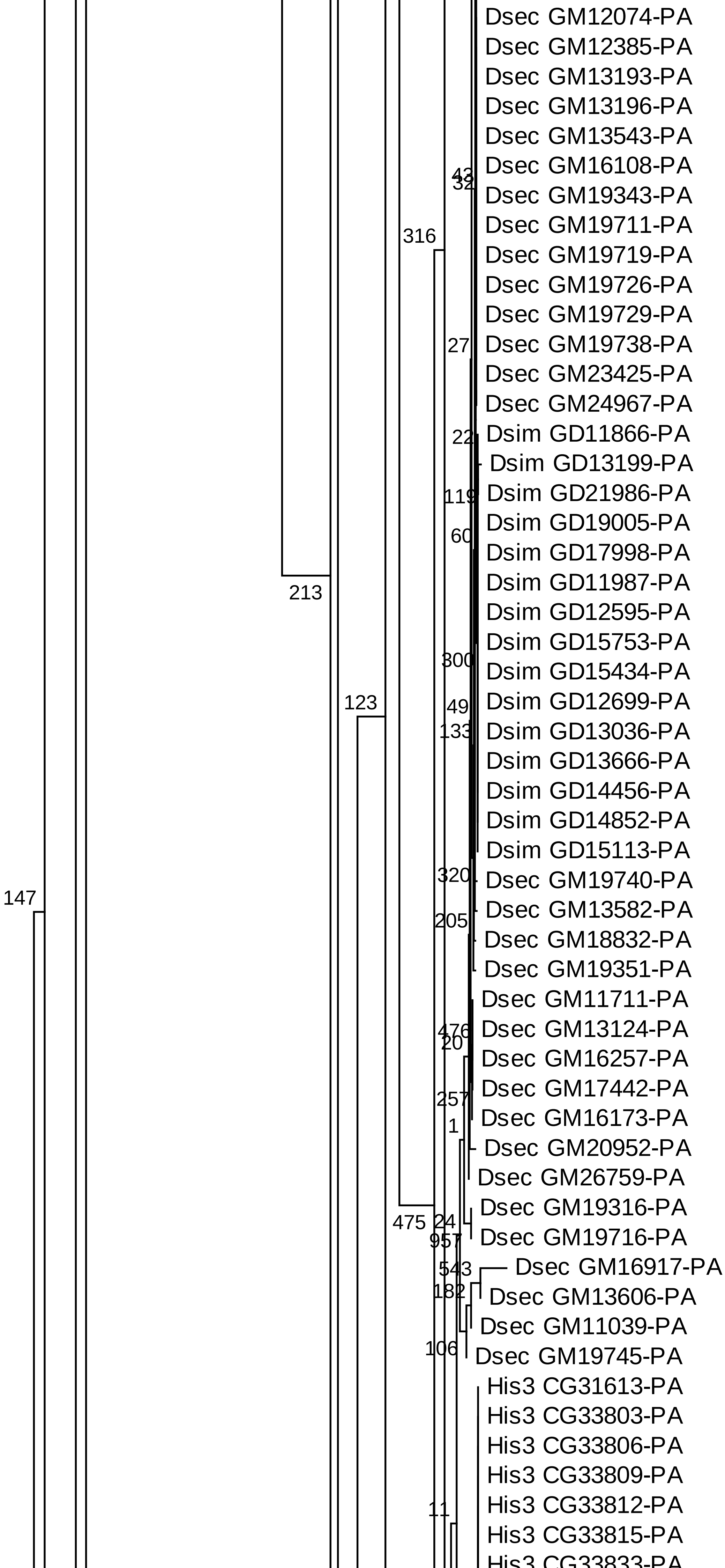

Dsec GM12074-PA
Dsec GM12385-PA
Dsec GM13193-PA
Dsec GM13196-PA
Dsec GM13543-PA
Dsec GM16108-PA
32
Dsec GM19343-PA
316
Dsec GM19711-PA
Dsec GM19719-PA
Dsec GM19726-PA
Dsec GM19729-PA
27
Dsec GM19738-PA
Dsec GM23425-PA
Dsec GM24967-PA
22
Dsim GD11866-PA
Dsim GD13199-PA
119
Dsim GD21986-PA
60
Dsim GD19005-PA
Dsim GD17998-PA
213
Dsim GD11987-PA
Dsim GD12595-PA
Dsim GD15753-PA
300
Dsim GD15434-PA
123
49
Dsim GD12699-PA
133
Dsim GD13036-PA
Dsim GD13666-PA
Dsim GD14456-PA
Dsim GD14852-PA
Dsim GD15113-PA
320
Dsec GM19740-PA
147
205
Dsec GM13582-PA
Dsec GM18832-PA
Dsec GM19351-PA
Dsec GM11711-PA
476
Dsec GM13124-PA
20
Dsec GM16257-PA
257
Dsec GM17442-PA
1
Dsec GM16173-PA
Dsec GM20952-PA
Dsec GM26759-PA
475
24
Dsec GM19316-PA
957
Dsec GM19716-PA
543
Dsec GM16917-PA
182
Dsec GM13606-PA
Dsec GM11039-PA
106
Dsec GM19745-PA
His3 CG31613-PA
His3 CG33803-PA
His3 CG33806-PA
His3 CG33809-PA
11
His3 CG33812-PA
His3 CG33815-PA

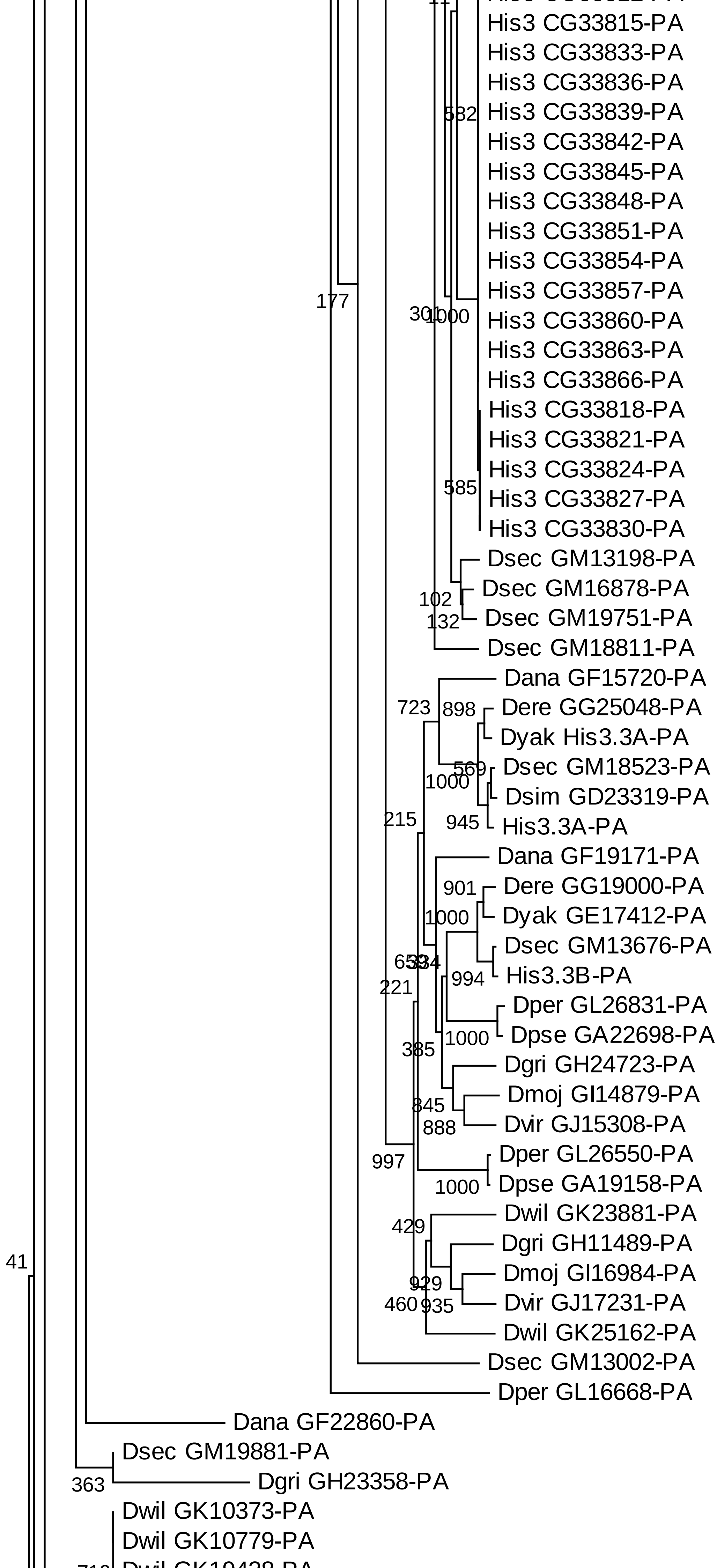

His3 CG33815-PA
His3 CG33833-PA
His3 CG33836-PA
582
His3 CG33839-PA
His3 CG33842-PA
His3 CG33845-PA
His3 CG33848-PA
His3 CG33851-PA
His3 CG33854-PA
His3 CG33857-PA
177
301
1000
His3 CG33860-PA
His3 CG33863-PA
His3 CG33866-PA
His3 CG33818-PA
His3 CG33821-PA
His3 CG33824-PA
585
His3 CG33827-PA
His3 CG33830-PA
Dsec GM13198-PA
Dsec GM16878-PA
102
132
Dsec GM19751-PA
Dsec GM18811-PA
Dana GF15720-PA
723
898
Dere GG25048-PA
Dyak His3.3A-PA
569
Dsec GM18523-PA
1000
Dsim GD23319-PA
215
945
His3.3A-PA
Dana GF19171-PA
901
Dere GG19000-PA
1000
Dyak GE17412-PA
Dsec GM13676-PA
221
994
His3.3B-PA
Dper GL26831-PA
1000
Dpse GA22698-PA
385
Dgri GH24723-PA
845
Dmoj GI14879-PA
888
Dvir GJ15308-PA
Dper GL26550-PA
997
1000
Dpse GA19158-PA
429
Dwil GK23881-PA
Dgri GH11489-PA
929
Dmoj GI16984-PA
460
935
Dvir GJ17231-PA
Dwil GK25162-PA
41
Dsec GM13002-PA
Dper GL16668-PA
Dana GF22860-PA
Dsec GM19881-PA
363
Dgri GH23358-PA
Dwil GK10373-PA
Dwil GK10779-PA

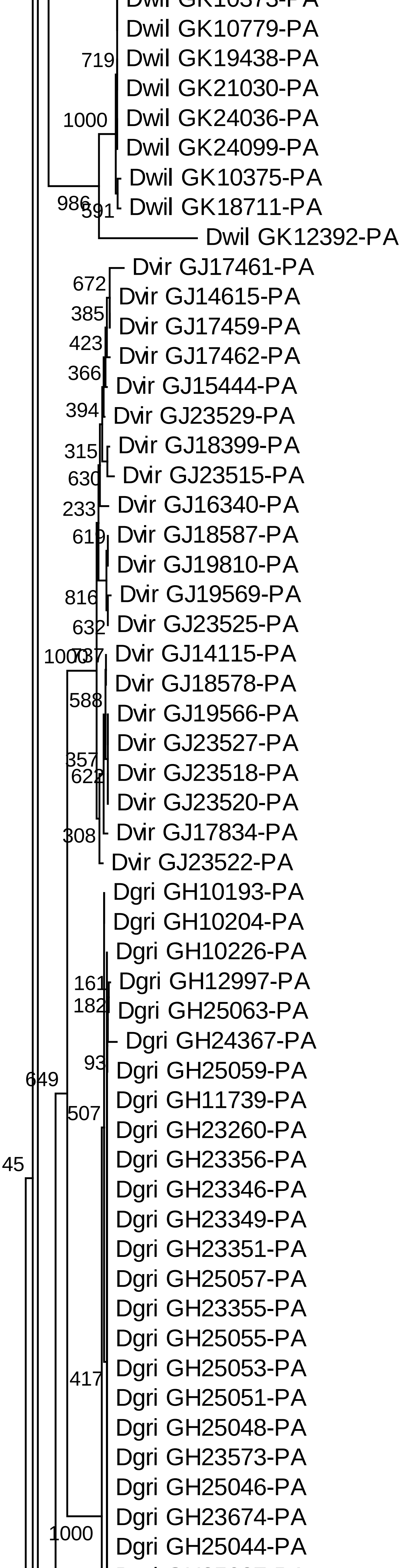

Dwil GK10779-PA
719
Dwil GK19438-PA
Dwil GK21030-PA
1000
Dwil GK24036-PA
Dwil GK24099-PA
Dwil GK10375-PA
986
591
Dwil GK18711-PA
Dwil GK12392-PA
Dvir GJ17461-PA
672
Dvir GJ14615-PA
385
Dvir GJ17459-PA
423
Dvir GJ17462-PA
366
Dvir GJ15444-PA
394
Dvir GJ23529-PA
315
Dvir GJ18399-PA
630
Dvir GJ23515-PA
233
Dvir GJ16340-PA
619
Dvir GJ18587-PA
Dvir GJ19810-PA
816
Dvir GJ19569-PA
632
Dvir GJ23525-PA
1000
737
Dvir GJ14115-PA
Dvir GJ18578-PA
588
Dvir GJ19566-PA
Dvir GJ23527-PA
357
622
Dvir GJ23518-PA
Dvir GJ23520-PA
308
Dvir GJ17834-PA
Dvir GJ23522-PA
Dgri GH10193-PA
Dgri GH10204-PA
Dgri GH10226-PA
161
Dgri GH12997-PA
182
Dgri GH25063-PA
Dgri GH24367-PA
93
Dgri GH25059-PA
649
Dgri GH11739-PA
507
Dgri GH23260-PA
45
Dgri GH23356-PA
Dgri GH23346-PA
Dgri GH23349-PA
Dgri GH23351-PA
Dgri GH25057-PA
Dgri GH23355-PA
Dgri GH25055-PA
Dgri GH25053-PA
417
Dgri GH25051-PA
Dgri GH25048-PA
Dgri GH23573-PA
Dgri GH25046-PA
Dgri GH23674-PA
1000
Dgri GH25044-PA

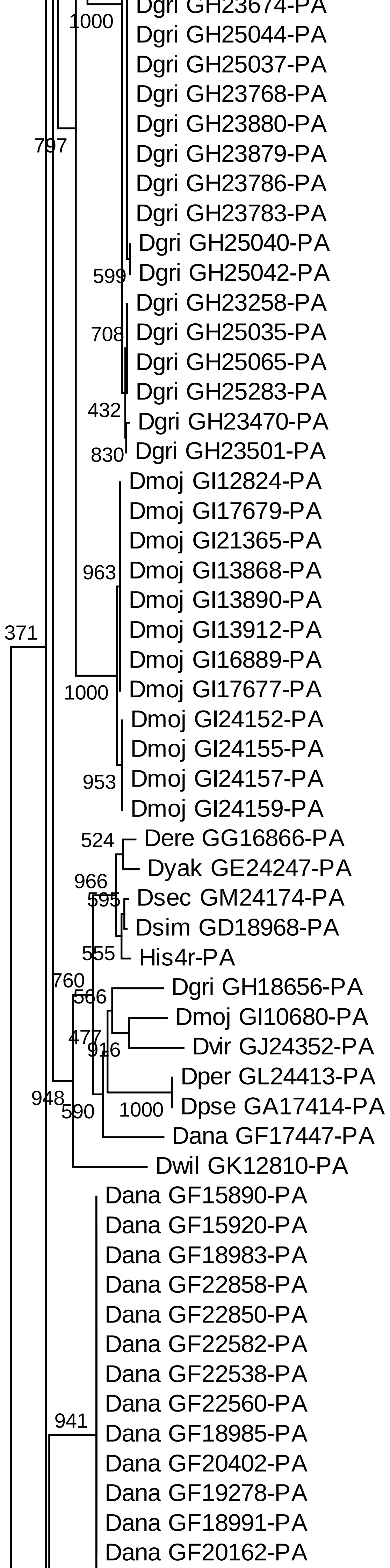

Dgri GH23674-PA
1000
Dgri GH25044-PA
Dgri GH25037-PA
Dgri GH23768-PA
Dgri GH23880-PA
797
Dgri GH23879-PA
Dgri GH23786-PA
Dgri GH23783-PA
Dgri GH25040-PA
599
Dgri GH25042-PA
Dgri GH23258-PA
708
Dgri GH25035-PA
Dgri GH25065-PA
Dgri GH25283-PA
432
Dgri GH23470-PA
830
Dgri GH23501-PA
Dmoj GI12824-PA
Dmoj GI17679-PA
Dmoj GI21365-PA
963
Dmoj GI13868-PA
Dmoj GI13890-PA
371
Dmoj GI13912-PA
Dmoj GI16889-PA
1000
Dmoj GI17677-PA
Dmoj GI24152-PA
Dmoj GI24155-PA
953
Dmoj GI24157-PA
Dmoj GI24159-PA
524
Dere GG16866-PA
966
Dyak GE24247-PA
595
Dsec GM24174-PA
Dsim GD18968-PA
555
His4r-PA
760
566
Dgri GH18656-PA
Dmoj GI10680-PA
477
916
Dvir GJ24352-PA
Dper GL24413-PA
948
590
1000
Dpse GA17414-PA
Dana GF17447-PA
Dwil GK12810-PA
Dana GF15890-PA
Dana GF15920-PA
Dana GF18983-PA
Dana GF22858-PA
Dana GF22850-PA
Dana GF22582-PA
Dana GF22538-PA
Dana GF22560-PA
941
Dana GF18985-PA
Dana GF20402-PA
Dana GF19278-PA
Dana GF18991-PA
Dana GF20162-PA

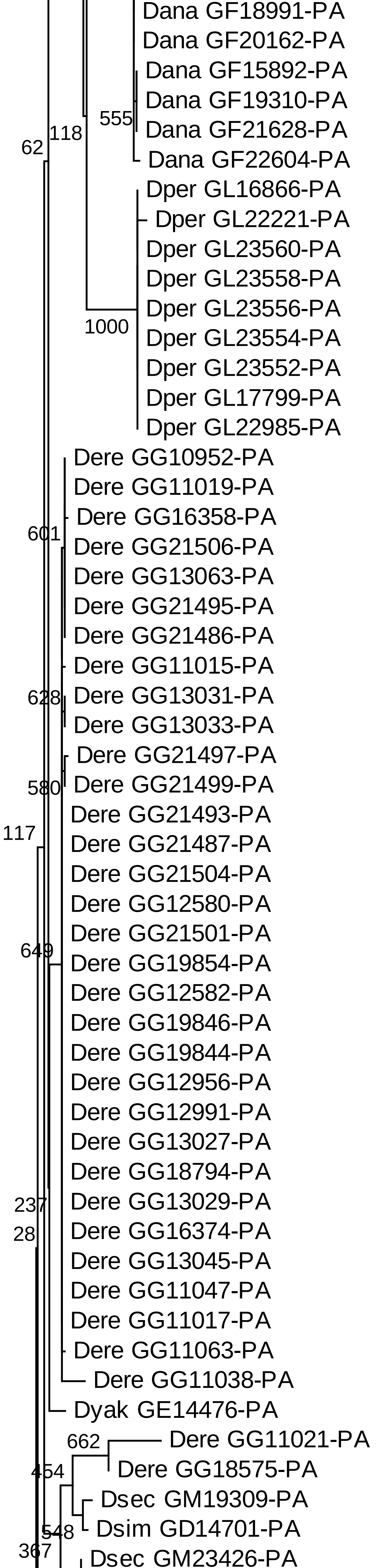
Dana GF18991-PA
Dana GF20162-PA
Dana GF15892-PA
Dana GF19310-PA
555
Dana GF21628-PA
118
62
Dana GF22604-PA
Dper GL16866-PA
Dper GL22221-PA
Dper GL23560-PA
Dper GL23558-PA
Dper GL23556-PA
1000
Dper GL23554-PA
Dper GL23552-PA
Dper GL17799-PA
Dper GL22985-PA
Dere GG10952-PA
Dere GG11019-PA
Dere GG16358-PA
601
Dere GG21506-PA
Dere GG13063-PA
Dere GG21495-PA
Dere GG21486-PA
Dere GG11015-PA
628
Dere GG13031-PA
Dere GG13033-PA
Dere GG21497-PA
580
Dere GG21499-PA
Dere GG21493-PA
117
Dere GG21487-PA
Dere GG21504-PA
Dere GG12580-PA
Dere GG21501-PA
649
Dere GG19854-PA
Dere GG12582-PA
Dere GG19846-PA
Dere GG19844-PA
Dere GG12956-PA
Dere GG12991-PA
Dere GG13027-PA
Dere GG18794-PA
237
Dere GG13029-PA
28
Dere GG16374-PA
Dere GG13045-PA
Dere GG11047-PA
Dere GG11017-PA
Dere GG11063-PA
Dere GG11038-PA
Dyak GE14476-PA
662
Dere GG11021-PA
454
Dere GG18575-PA
Dsec GM19309-PA
548
Dsim GD14701-PA
367
Dsec GM23426-PA

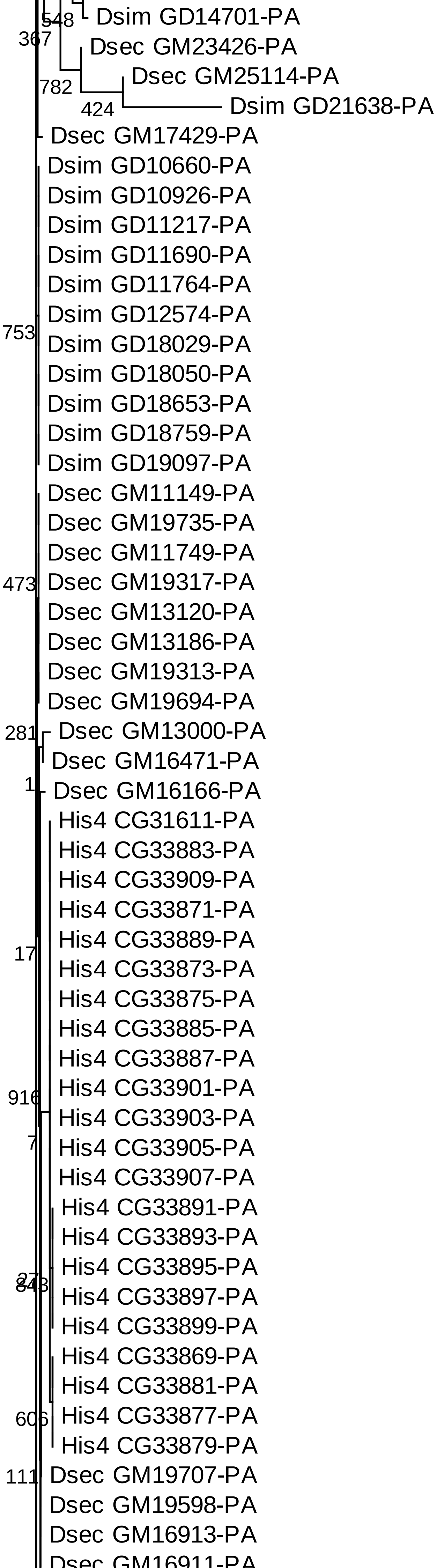
Dsim GD14701-PA
548
367
Dsec GM23426-PA
Dsec GM25114-PA
782
424
Dsim GD21638-PA
Dsec GM17429-PA
Dsim GD10660-PA
Dsim GD10926-PA
Dsim GD11217-PA
Dsim GD11690-PA
Dsim GD11764-PA
Dsim GD12574-PA
753
Dsim GD18029-PA
Dsim GD18050-PA
Dsim GD18653-PA
Dsim GD18759-PA
Dsim GD19097-PA
Dsec GM11149-PA
Dsec GM19735-PA
Dsec GM11749-PA
473
Dsec GM19317-PA
Dsec GM13120-PA
Dsec GM13186-PA
Dsec GM19313-PA
Dsec GM19694-PA
281
Dsec GM13000-PA
Dsec GM16471-PA
1
Dsec GM16166-PA
His4 CG31611-PA
His4 CG33883-PA
His4 CG33909-PA
His4 CG33871-PA
His4 CG33889-PA
17
His4 CG33873-PA
His4 CG33875-PA
His4 CG33885-PA
His4 CG33887-PA
His4 CG33901-PA
916
His4 CG33903-PA
7
His4 CG33905-PA
His4 CG33907-PA
His4 CG33891-PA
His4 CG33893-PA
His4 CG33895-PA
27
843
His4 CG33897-PA
His4 CG33899-PA
His4 CG33869-PA
His4 CG33881-PA
606
His4 CG33877-PA
His4 CG33879-PA
111
Dsec GM19707-PA
Dsec GM19598-PA
Dsec GM16913-PA
Dsec GM16911-PA

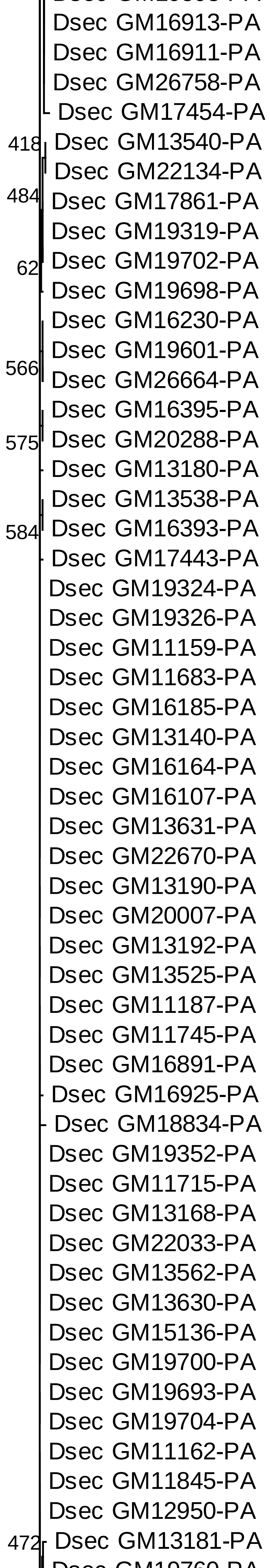
Dsec GM16913-PA
Dsec GM16911-PA
Dsec GM26758-PA
Dsec GM17454-PA
418
Dsec GM13540-PA
Dsec GM22134-PA
484
Dsec GM17861-PA
Dsec GM19319-PA
62
Dsec GM19702-PA
Dsec GM19698-PA
Dsec GM16230-PA
Dsec GM19601-PA
566
Dsec GM26664-PA
Dsec GM16395-PA
575
Dsec GM20288-PA
Dsec GM13180-PA
Dsec GM13538-PA
584
Dsec GM16393-PA
Dsec GM17443-PA
Dsec GM19324-PA
Dsec GM19326-PA
Dsec GM11159-PA
Dsec GM11683-PA
Dsec GM16185-PA
Dsec GM13140-PA
Dsec GM16164-PA
Dsec GM16107-PA
Dsec GM13631-PA
Dsec GM22670-PA
Dsec GM13190-PA
Dsec GM20007-PA
Dsec GM13192-PA
Dsec GM13525-PA
Dsec GM11187-PA
Dsec GM11745-PA
Dsec GM16891-PA
Dsec GM16925-PA
Dsec GM18834-PA
Dsec GM19352-PA
Dsec GM11715-PA
Dsec GM13168-PA
Dsec GM22033-PA
Dsec GM13562-PA
Dsec GM13630-PA
Dsec GM15136-PA
Dsec GM19700-PA
Dsec GM19693-PA
Dsec GM19704-PA
Dsec GM11162-PA
Dsec GM11845-PA
Dsec GM12950-PA
472
Dsec GM13181-PA

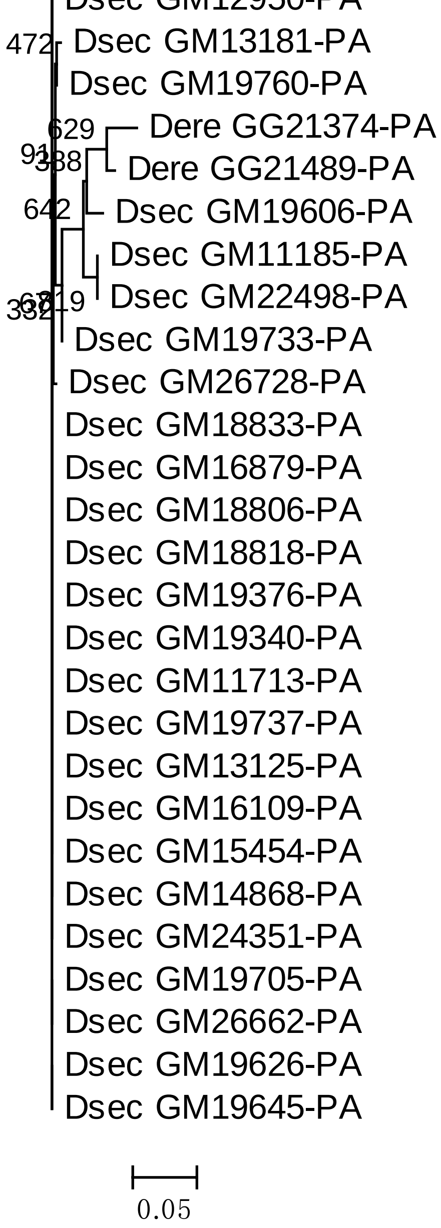

Dsec GM12950-PA
472
Dsec GM13181-PA
Dsec GM19760-PA
629
Dere GG21374-PA
91
338
Dere GG21489-PA
642
Dsec GM19606-PA
Dsec GM11185-PA
Dsec GM22498-PA
Dsec GM19733-PA
Dsec GM26728-PA
Dsec GM18833-PA
Dsec GM16879-PA
Dsec GM18806-PA
Dsec GM18818-PA
Dsec GM19376-PA
Dsec GM19340-PA
Dsec GM11713-PA
Dsec GM19737-PA
Dsec GM13125-PA
Dsec GM16109-PA
Dsec GM15454-PA
Dsec GM14868-PA
Dsec GM24351-PA
Dsec GM19705-PA
Dsec GM26662-PA
Dsec GM19626-PA
Dsec GM19645-PA
0.05

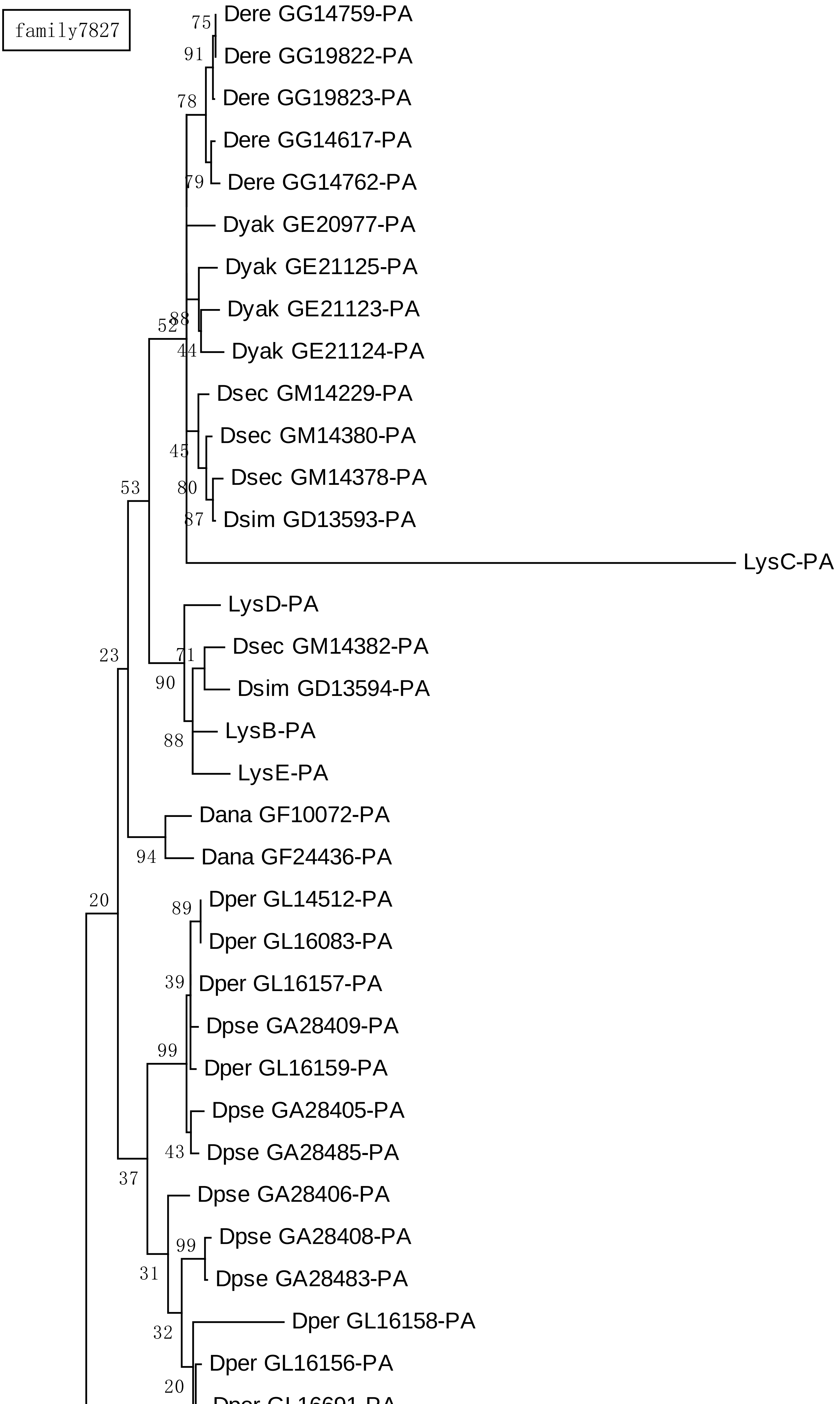
family7827
Dere GG14759-PA
Dere GG19822-PA
Dere GG19823-PA
Dere GG14617-PA
Dere GG14762-PA
Dyak GE20977-PA
Dyak GE21125-PA
Dyak GE21123-PA
Dyak GE21124-PA
Dsec GM14229-PA
Dsec GM14380-PA
Dsec GM14378-PA
Dsim GD13593-PA
LysC-PA
LysD-PA
Dsec GM14382-PA
Dsim GD13594-PA
LysB-PA
LysE-PA
Dana GF10072-PA
Dana GF24436-PA
Dper GL14512-PA
Dper GL16083-PA
Dper GL16157-PA
Dpse GA28409-PA
Dper GL16159-PA
Dpse GA28405-PA
Dpse GA28485-PA
Dpse GA28406-PA
Dpse GA28408-PA
Dpse GA28483-PA
Dper GL16158-PA
Dper GL16156-PA

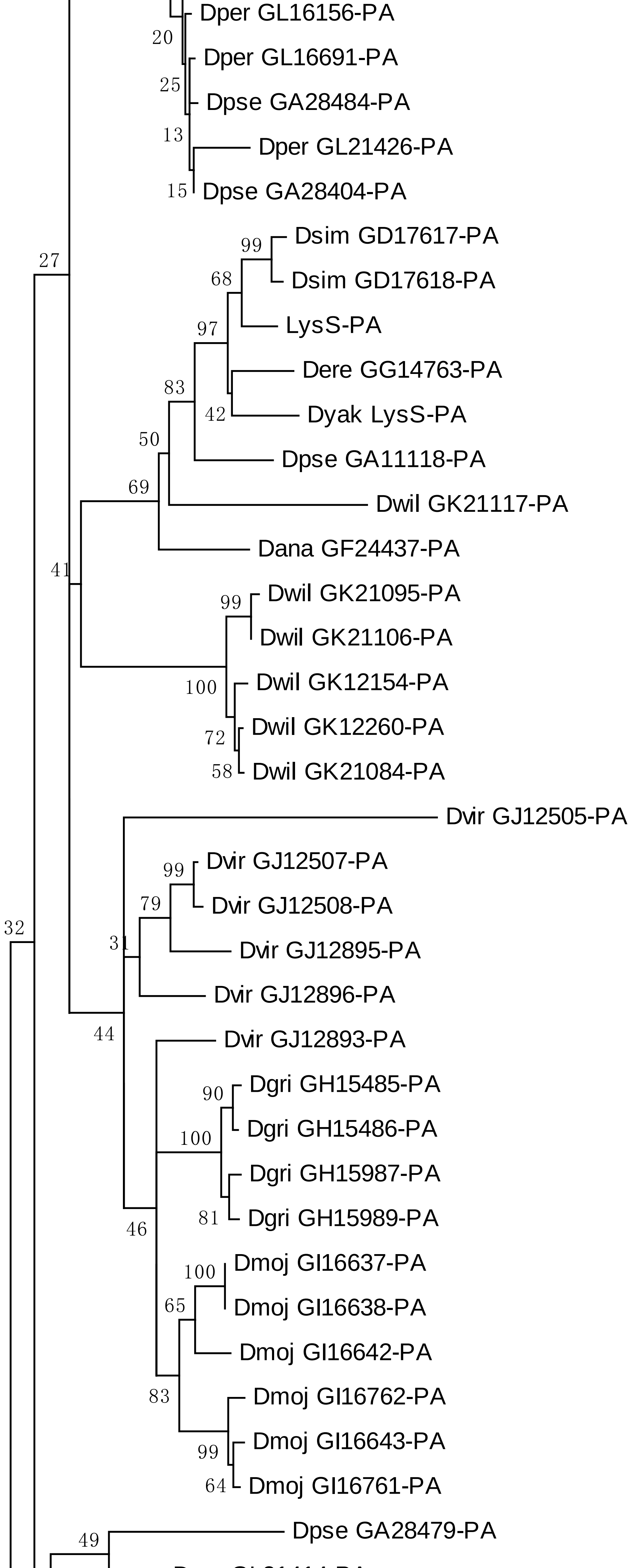

Dper GL16156-PA
20
Dper GL16691-PA
25
Dpse GA28484-PA
13
Dper GL21426-PA
15
Dpse GA28404-PA
27
99
Dsim GD17617-PA
68
Dsim GD17618-PA
97
LysS-PA
83
Dere GG14763-PA
42
Dyak LysS-PA
50
Dpse GA11118-PA
69
Dwil GK21117-PA
41
Dana GF24437-PA
99
Dwil GK21095-PA
Dwil GK21106-PA
100
Dwil GK12154-PA
72
Dwil GK12260-PA
58
Dwil GK21084-PA
Dvir GJ12505-PA
99
Dvir GJ12507-PA
79
Dvir GJ12508-PA
32
31
Dvir GJ12895-PA
Dvir GJ12896-PA
44
Dvir GJ12893-PA
90
Dgri GH15485-PA
100
Dgri GH15486-PA
Dgri GH15987-PA
81
Dgri GH15989-PA
46
100
Dmoj GI16637-PA
65
Dmoj GI16638-PA
Dmoj GI16642-PA
83
Dmoj GI16762-PA
99
Dmoj GI16643-PA
64
Dmoj GI16761-PA
49
Dpse GA28479-PA

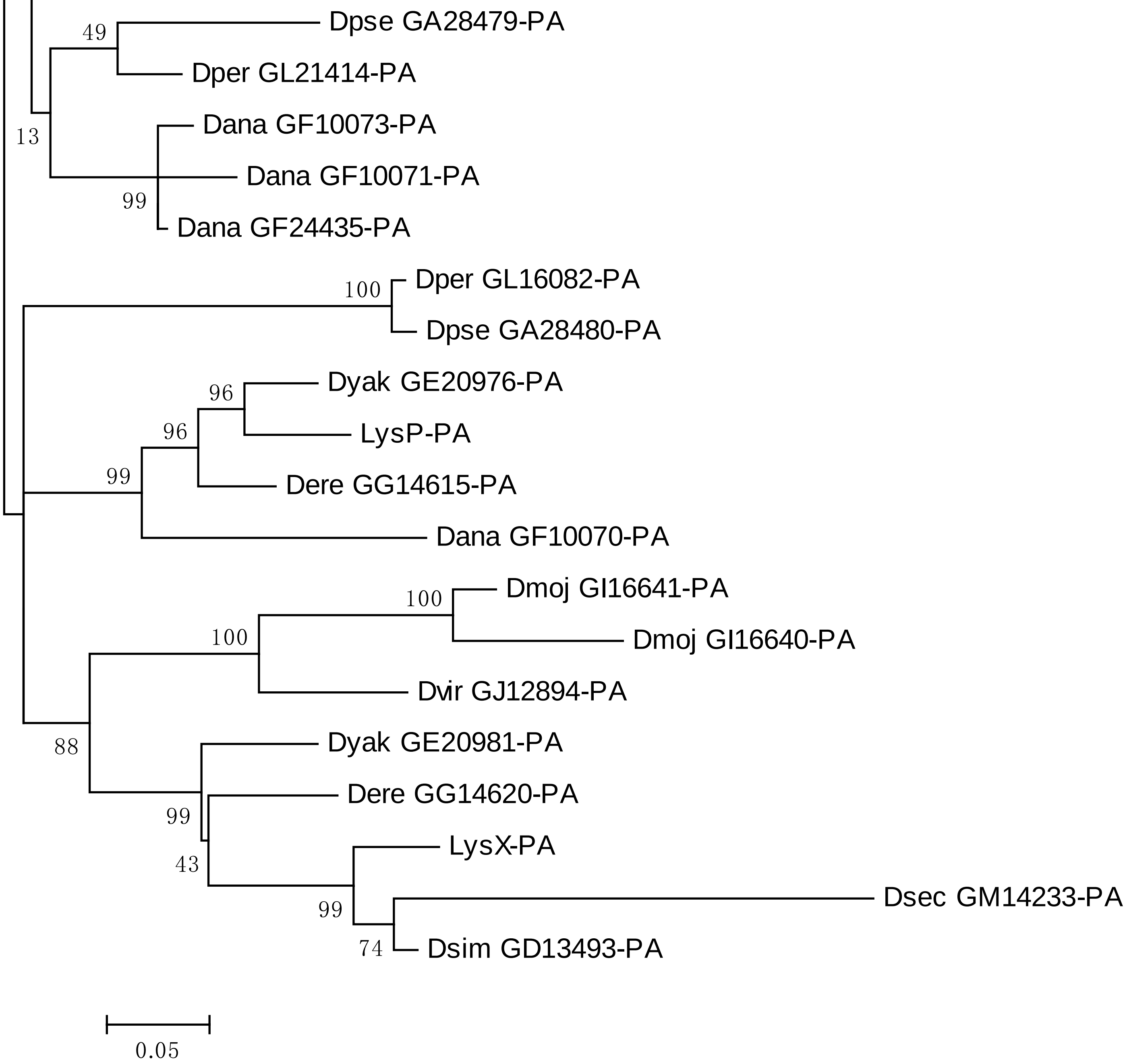
Dpse GA28479-PA
Dper GL21414-PA
Dana GF10073-PA
Dana GF10071-PA
Dana GF24435-PA
Dper GL16082-PA
Dpse GA28480-PA
Dyak GE20976-PA
LysP-PA
Dere GG14615-PA
Dana GF10070-PA
Dmoj GI16641-PA
Dmoj GI16640-PA
Dvir GJ12894-PA
Dyak GE20981-PA
Dere GG14620-PA
LysX-PA
Dsec GM14233-PA
Dsim GD13493-PA
49
13
99
100
96
96
99
100
100
88
99
43
99
74
0.05

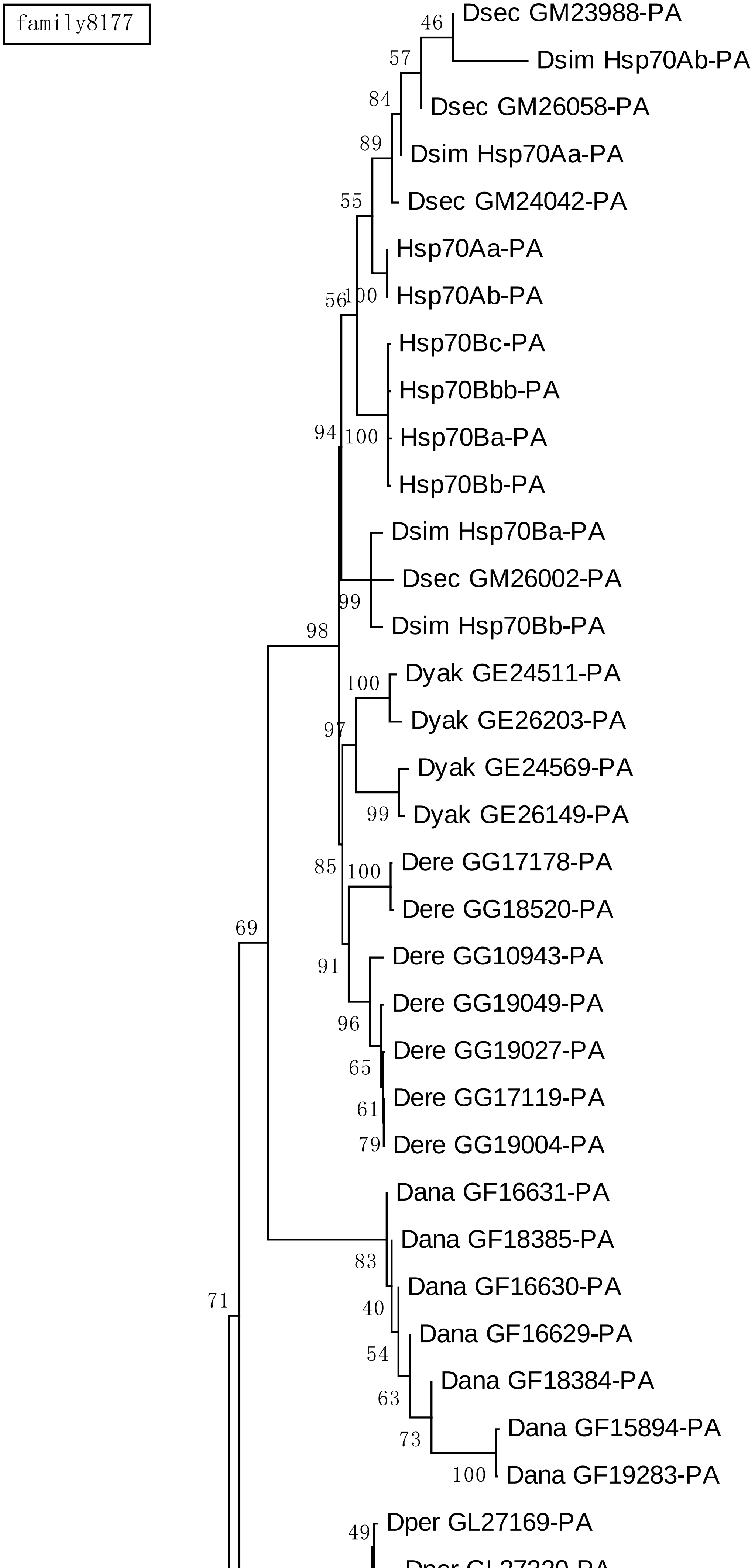
family8177
Dsec GM23988-PA
Dsim Hsp70Ab-PA
Dsec GM26058-PA
Dsim Hsp70Aa-PA
Dsec GM24042-PA
Hsp70Aa-PA
Hsp70Ab-PA
Hsp70Bc-PA
Hsp70Bbb-PA
Hsp70Ba-PA
Hsp70Bb-PA
Dsim Hsp70Ba-PA
Dsec GM26002-PA
Dsim Hsp70Bb-PA
Dyak GE24511-PA
Dyak GE26203-PA
Dyak GE24569-PA
Dyak GE26149-PA
Dere GG17178-PA
Dere GG18520-PA
Dere GG10943-PA
Dere GG19049-PA
Dere GG19027-PA
Dere GG17119-PA
Dere GG19004-PA
Dana GF16631-PA
Dana GF18385-PA
Dana GF16630-PA
Dana GF16629-PA
Dana GF18384-PA
Dana GF15894-PA
Dana GF19283-PA
Dper GL27169-PA
46
57
84
89
55
56
100
94
100
99
98
100
97
99
85
100
69
91
96
65
61
79
83
71
40
54
63
73
100
49

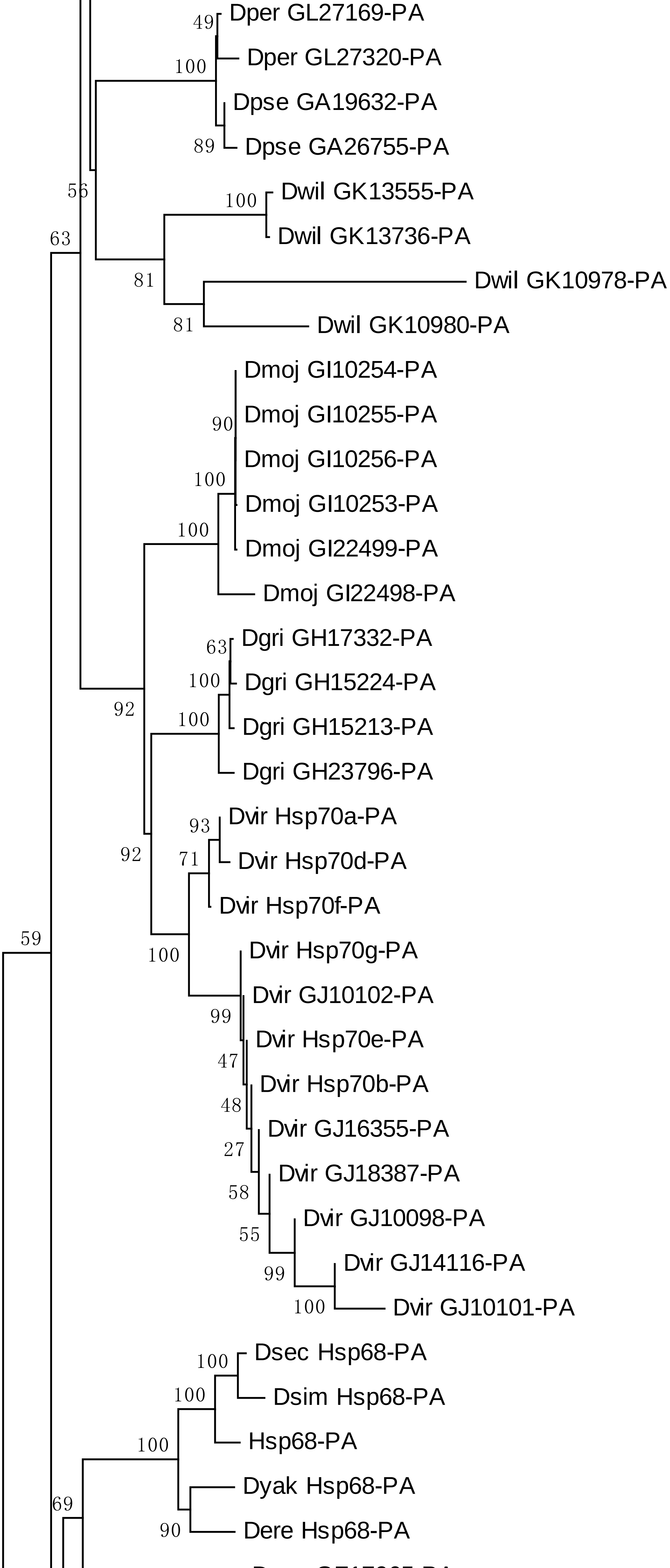
Dper GL27169-PA
Dper GL27320-PA
Dpse GA19632-PA
Dpse GA26755-PA
Dwil GK13555-PA
Dwil GK13736-PA
Dwil GK10978-PA
Dwil GK10980-PA
Dmoj GI10254-PA
Dmoj GI10255-PA
Dmoj GI10256-PA
Dmoj GI10253-PA
Dmoj GI22499-PA
Dmoj GI22498-PA
Dgri GH17332-PA
Dgri GH15224-PA
Dgri GH15213-PA
Dgri GH23796-PA
Dvir Hsp70a-PA
Dvir Hsp70d-PA
Dvir Hsp70f-PA
Dvir Hsp70g-PA
Dvir GJ10102-PA
Dvir Hsp70e-PA
Dvir Hsp70b-PA
Dvir GJ16355-PA
Dvir GJ18387-PA
Dvir GJ10098-PA
Dvir GJ14116-PA
Dvir GJ10101-PA
Dsec Hsp68-PA
Dsim Hsp68-PA
Hsp68-PA
Dyak Hsp68-PA
Dere Hsp68-PA

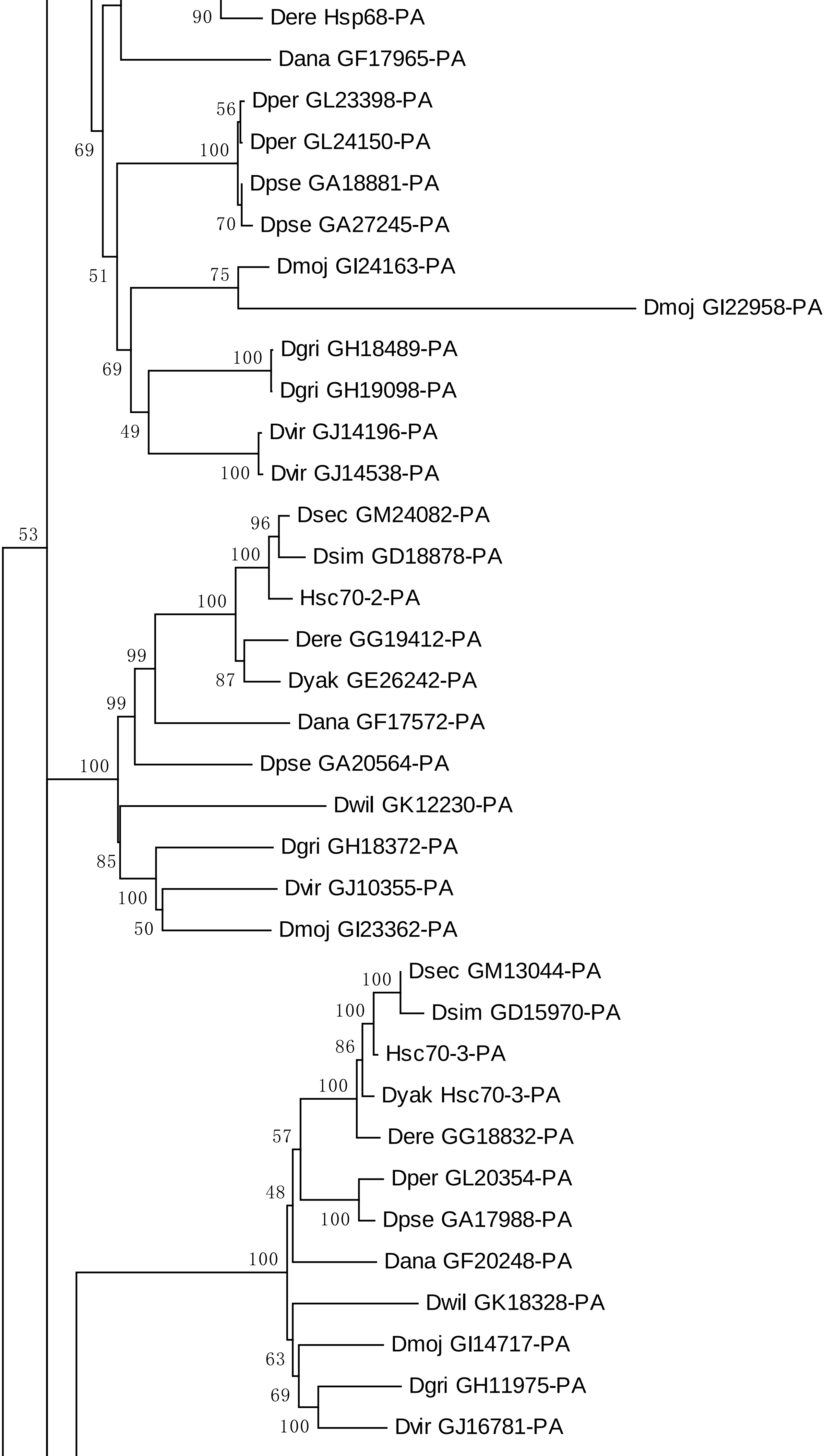

90
Dere Hsp68-PA
Dana GF17965-PA
56
Dper GL23398-PA
69
100
Dper GL24150-PA
Dpse GA18881-PA
70
Dpse GA27245-PA
51
75
Dmoj GI24163-PA
Dmoj GI22958-PA
100
Dgri GH18489-PA
69
Dgri GH19098-PA
49
Dvir GJ14196-PA
100
Dvir GJ14538-PA
96
Dsec GM24082-PA
53
100
Dsim GD18878-PA
100
Hsc70-2-PA
Dere GG19412-PA
99
87
Dyak GE26242-PA
99
Dana GF17572-PA
100
Dpse GA20564-PA
Dwil GK12230-PA
Dgri GH18372-PA
85
100
Dvir GJ10355-PA
50
Dmoj GI23362-PA
100
Dsec GM13044-PA
100
Dsim GD15970-PA
86
Hsc70-3-PA
100
Dyak Hsc70-3-PA
57
Dere GG18832-PA
Dper GL20354-PA
48
100
Dpse GA17988-PA
100
Dana GF20248-PA
Dwil GK18328-PA
Dmoj GI14717-PA
63
69
Dgri GH11975-PA
100
Dvir GJ16781-PA

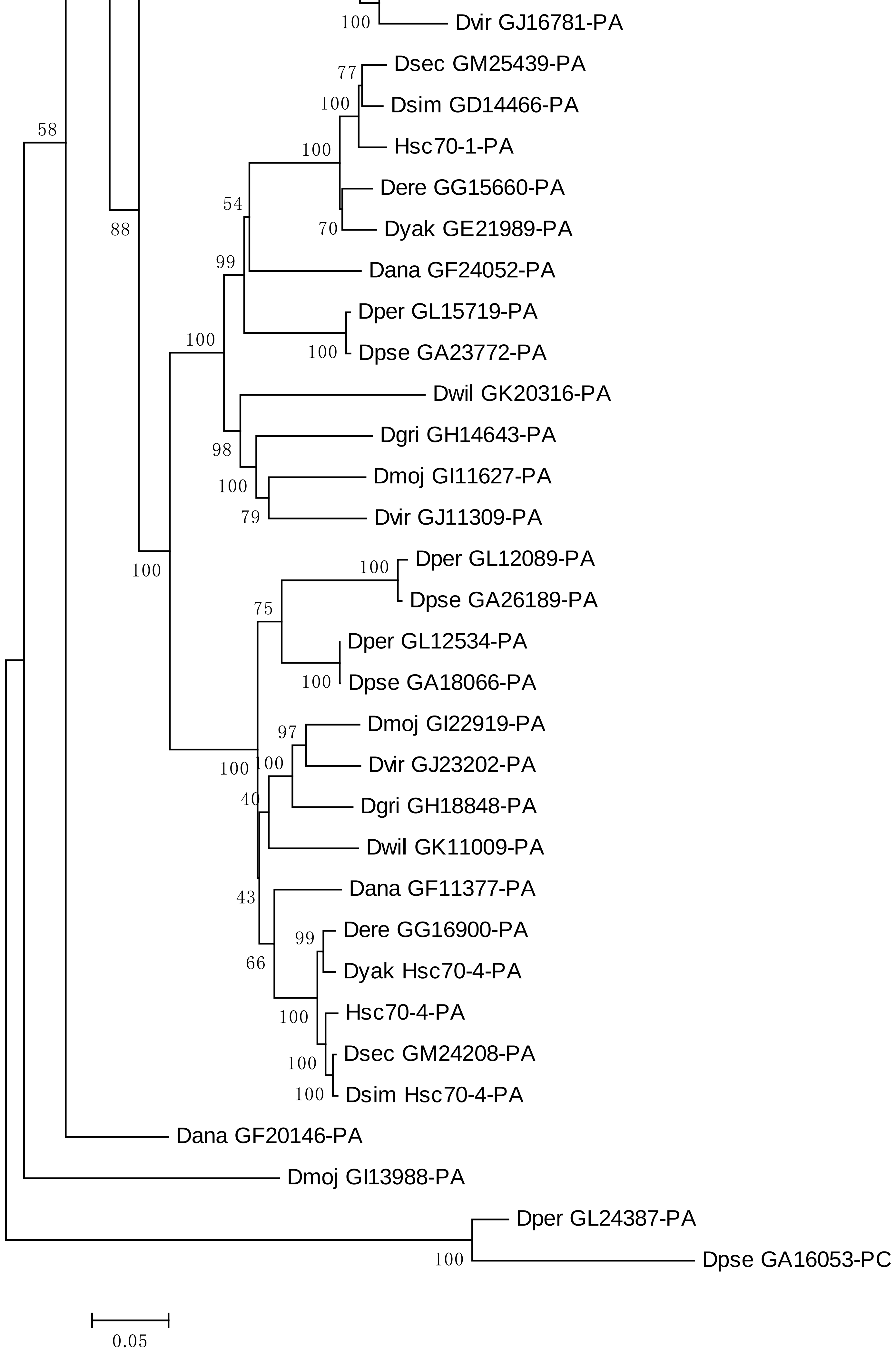
Dvir GJ16781-PA
Dsec GM25439-PA
Dsim GD14466-PA
Hsc70-1-PA
Dere GG15660-PA
Dyak GE21989-PA
Dana GF24052-PA
Dper GL15719-PA
Dpse GA23772-PA
Dwil GK20316-PA
Dgri GH14643-PA
Dmoj GI11627-PA
Dvir GJ11309-PA
Dper GL12089-PA
Dpse GA26189-PA
Dper GL12534-PA
Dpse GA18066-PA
Dmoj GI22919-PA
Dvir GJ23202-PA
Dgri GH18848-PA
Dwil GK11009-PA
Dana GF11377-PA
Dere GG16900-PA
Dyak Hsc70-4-PA
Hsc70-4-PA
Dsec GM24208-PA
Dsim Hsc70-4-PA
Dana GF20146-PA
Dmoj GI13988-PA
Dper GL24387-PA
Dpse GA16053-PC
0.05
