# Supplementary figures and images for: Functional requirements driving the gene duplication in 12 Drosophila species
Source: BMC Genomics. 2013 Aug 15;14:555. doi: 10.1186/1471-2164-14-555 (PMC3751352; doi:10.1186/1471-2164-14-555)

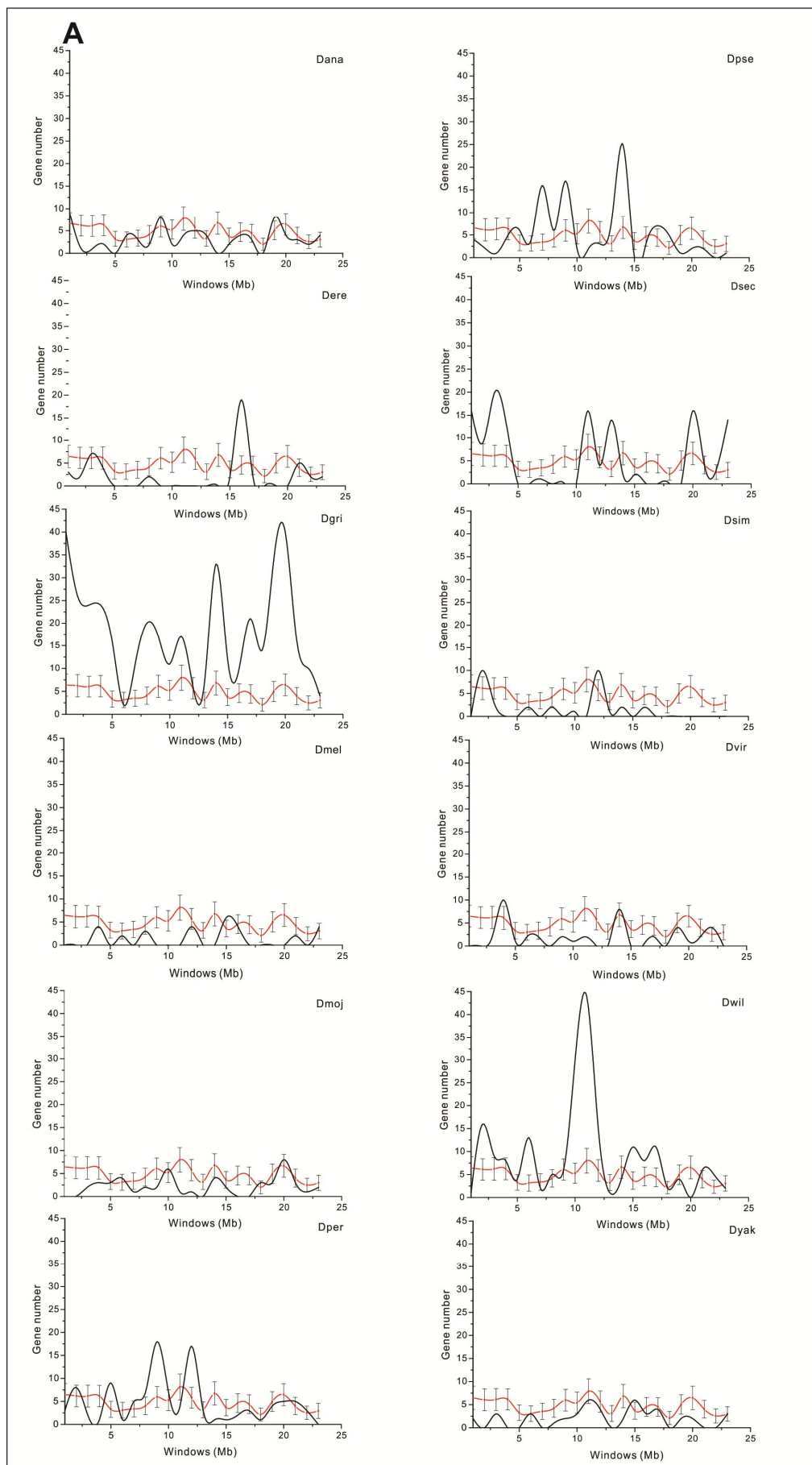

**B**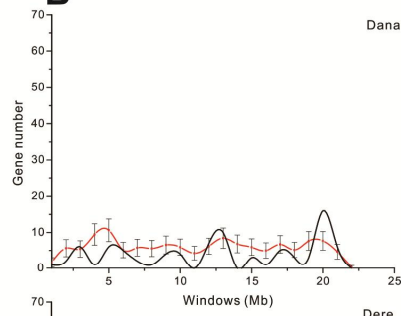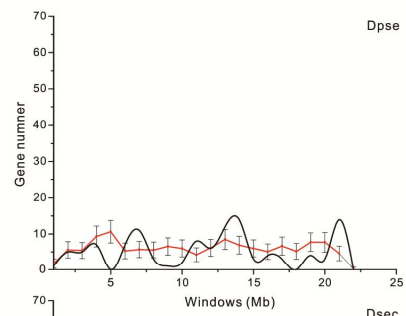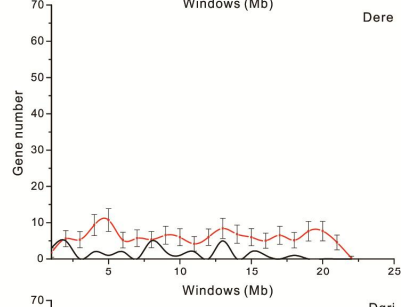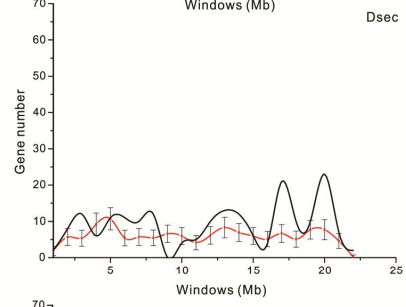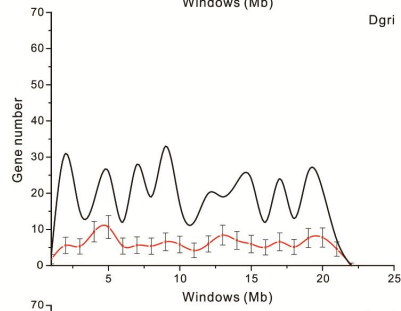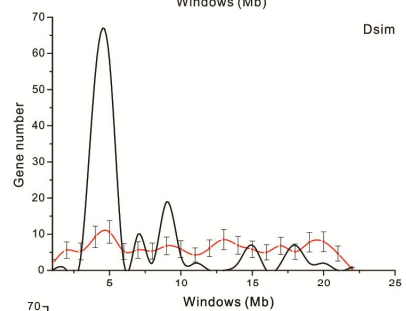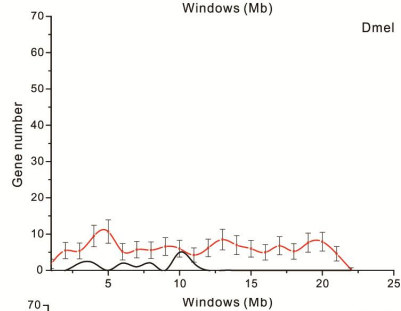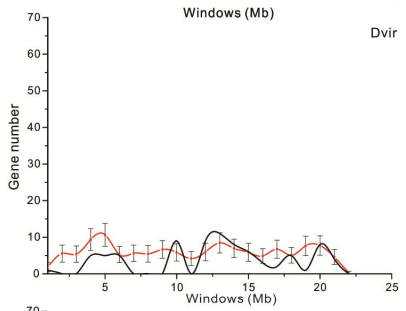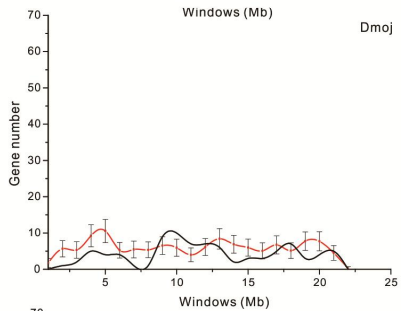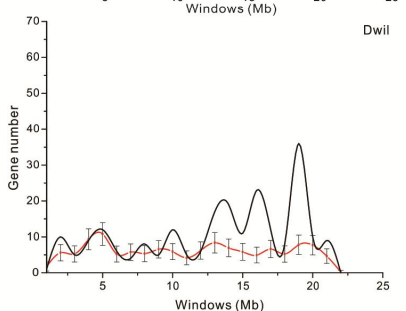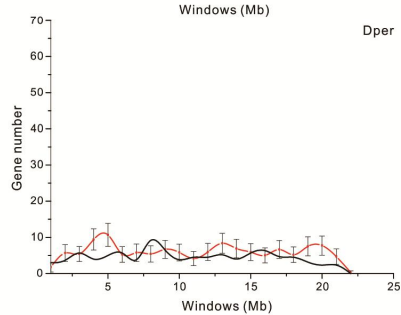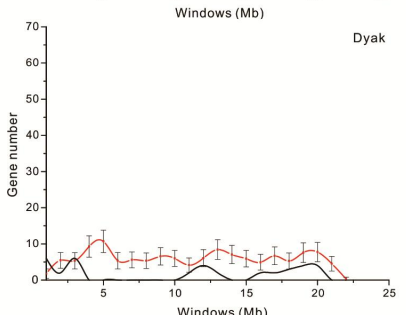

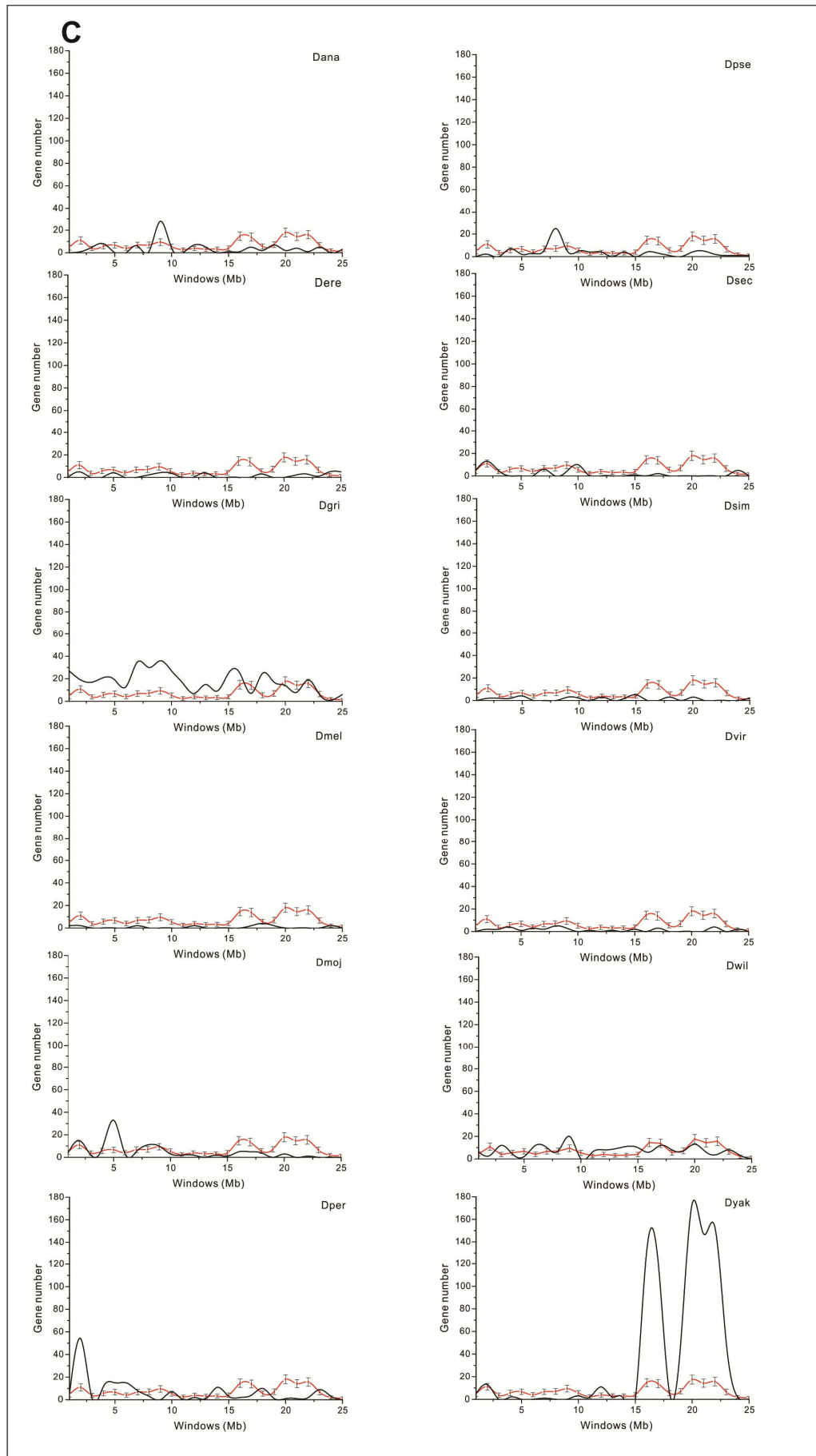

**D**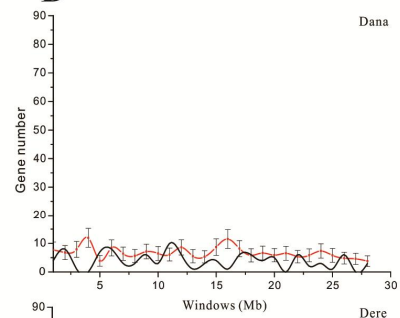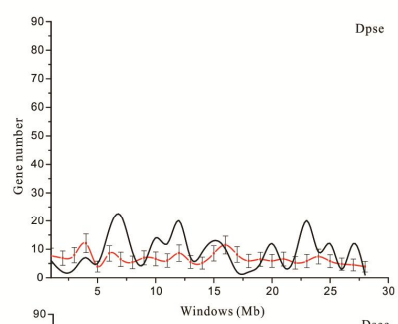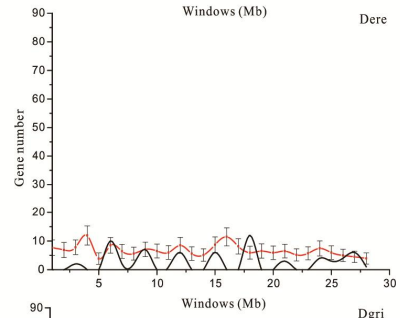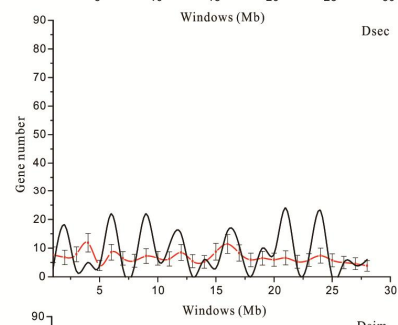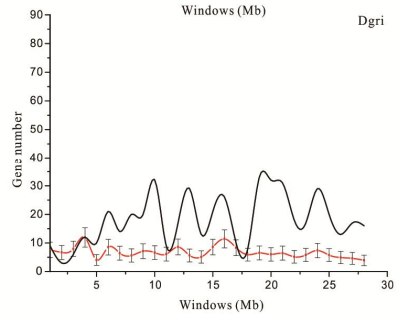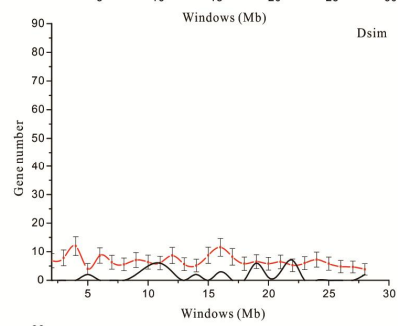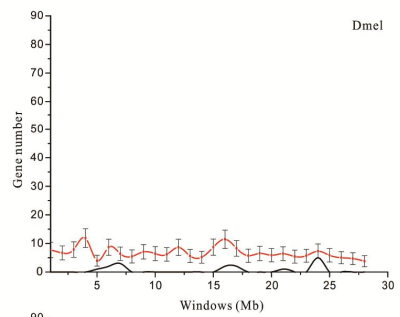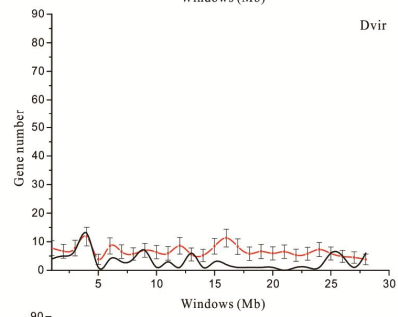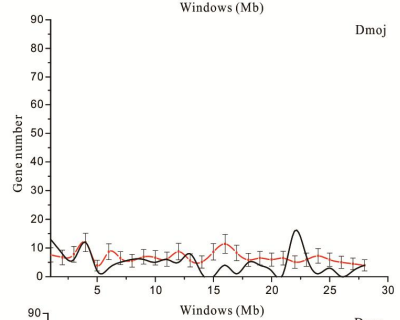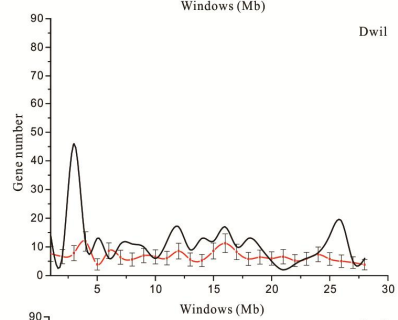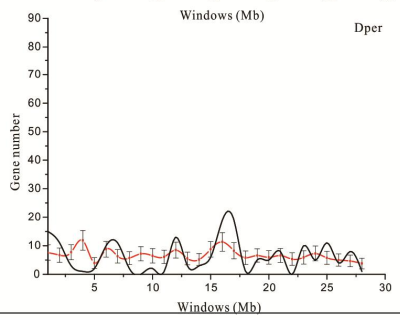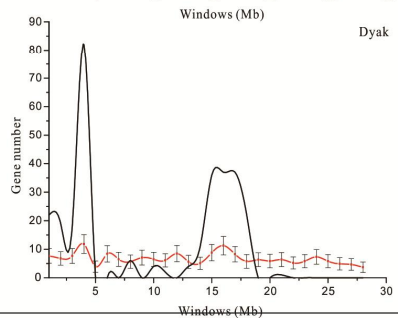

**E**

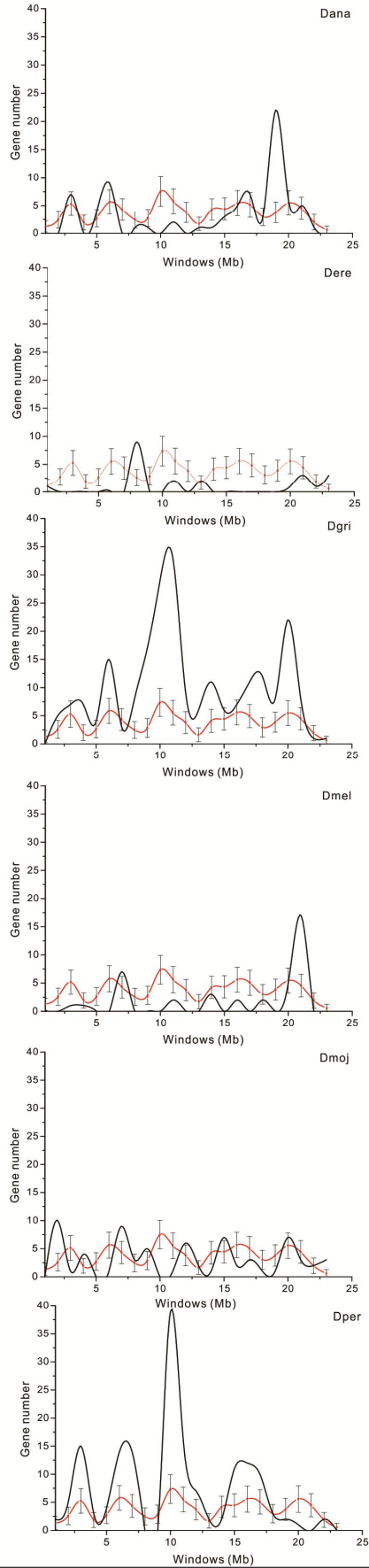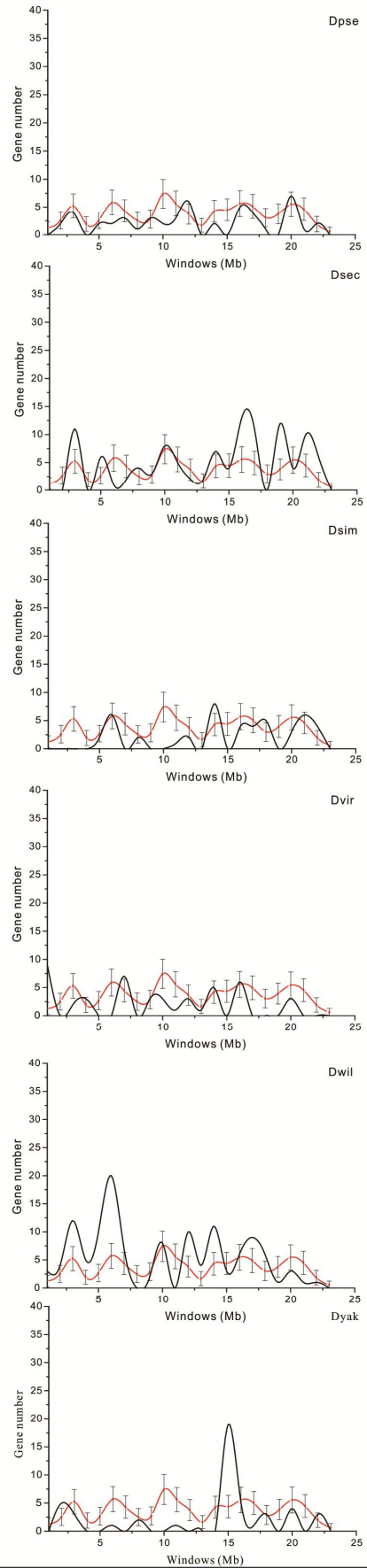

**F**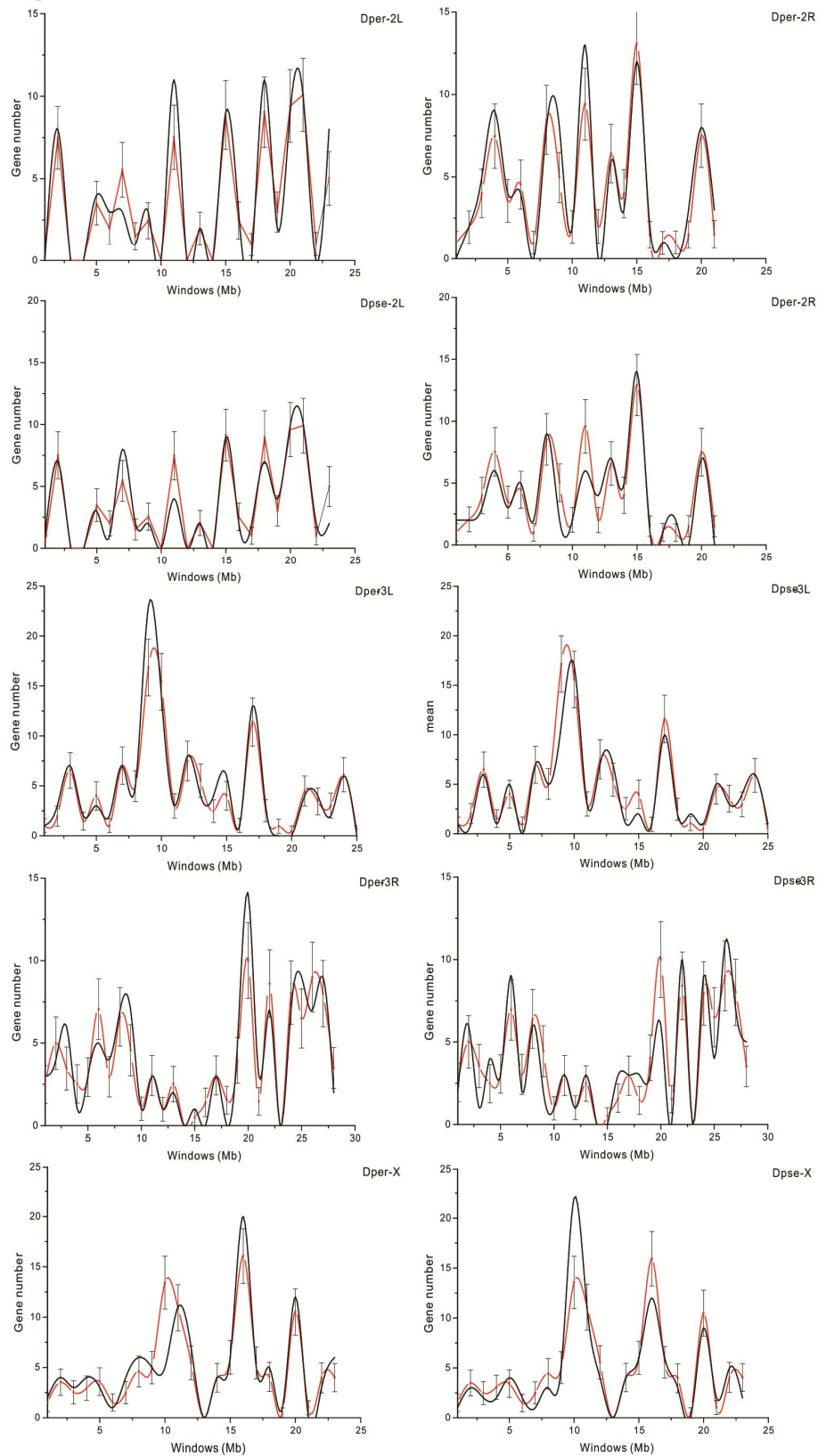

**G**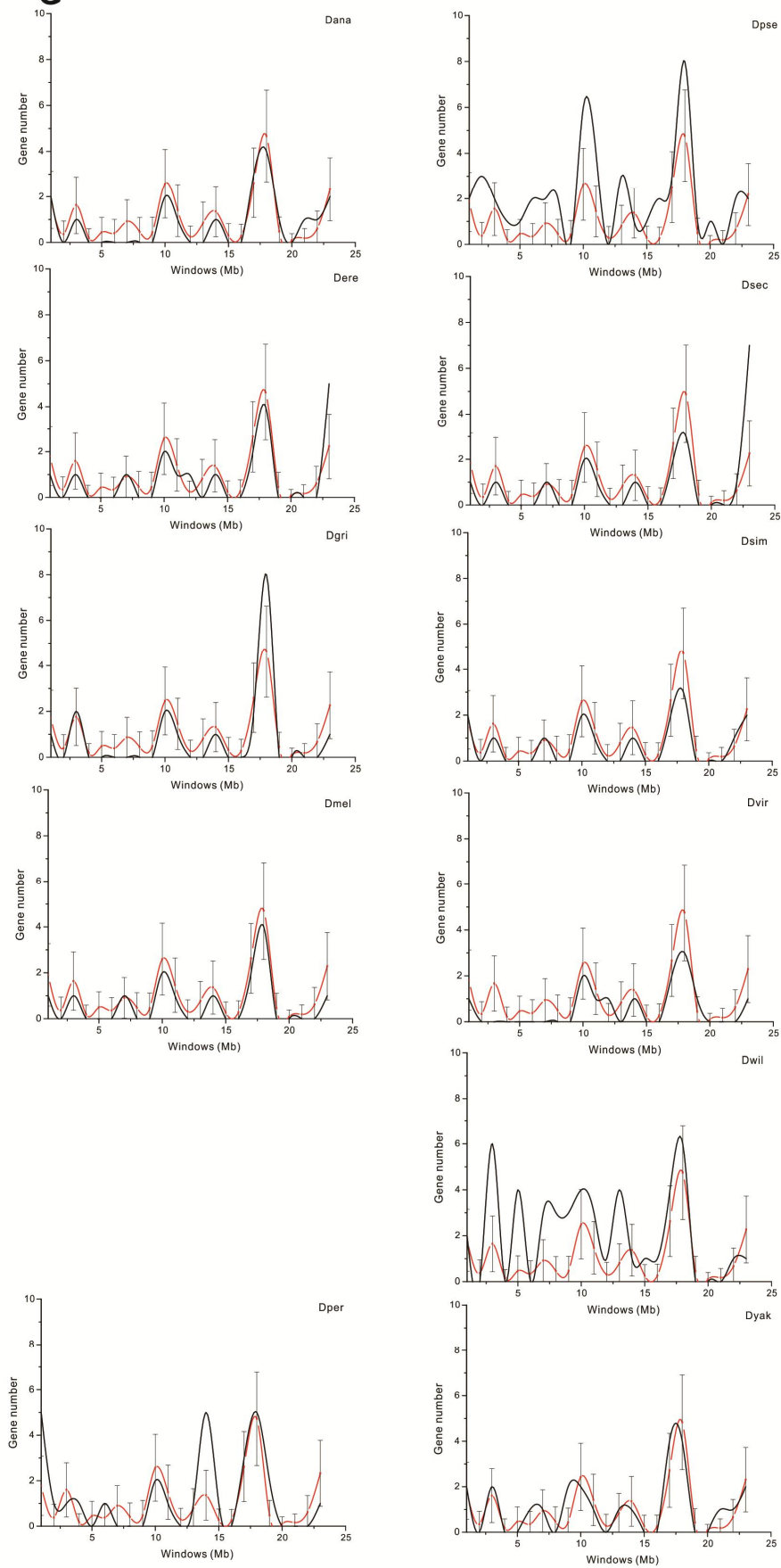

**H**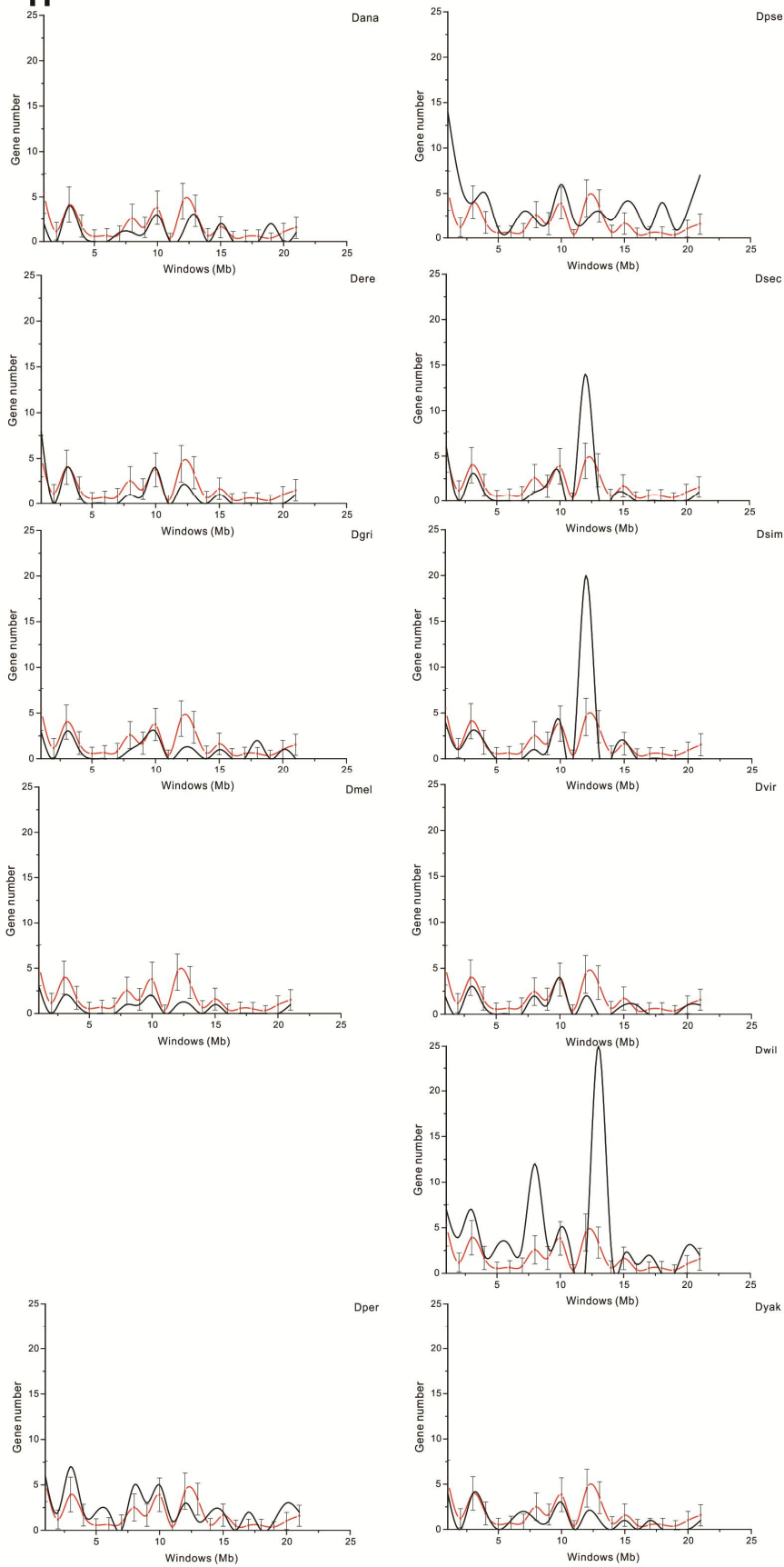

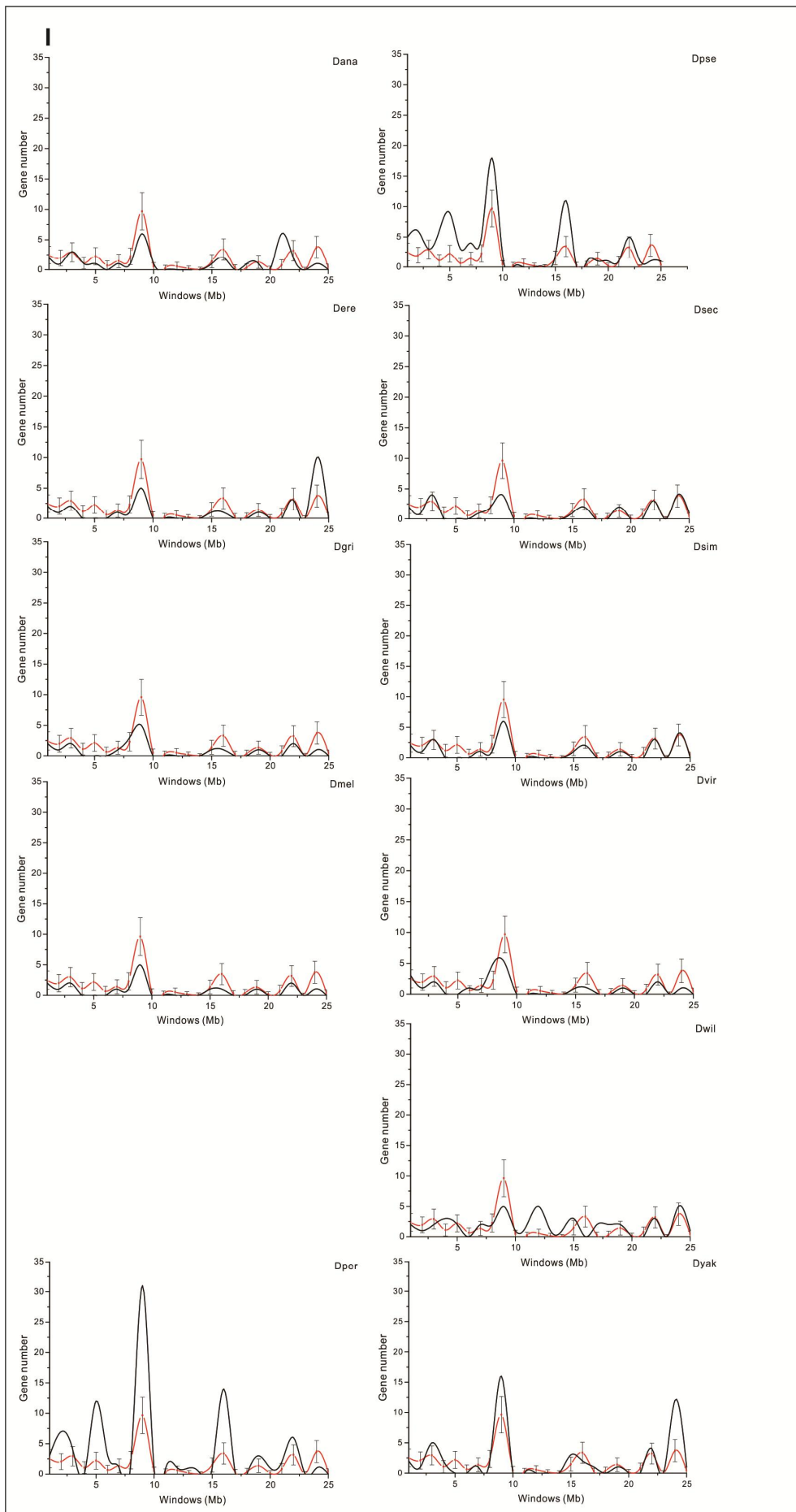

**J**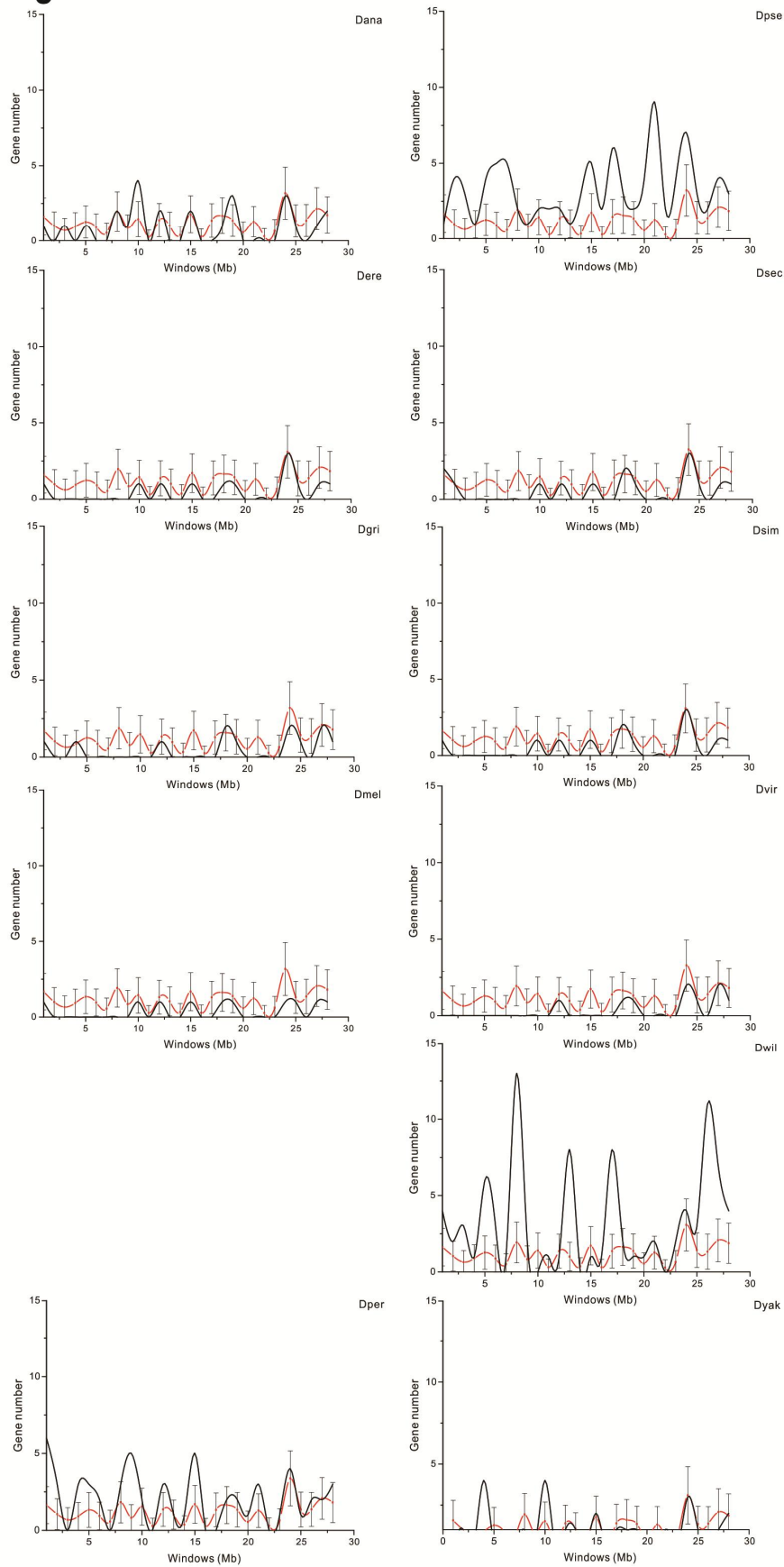

**K**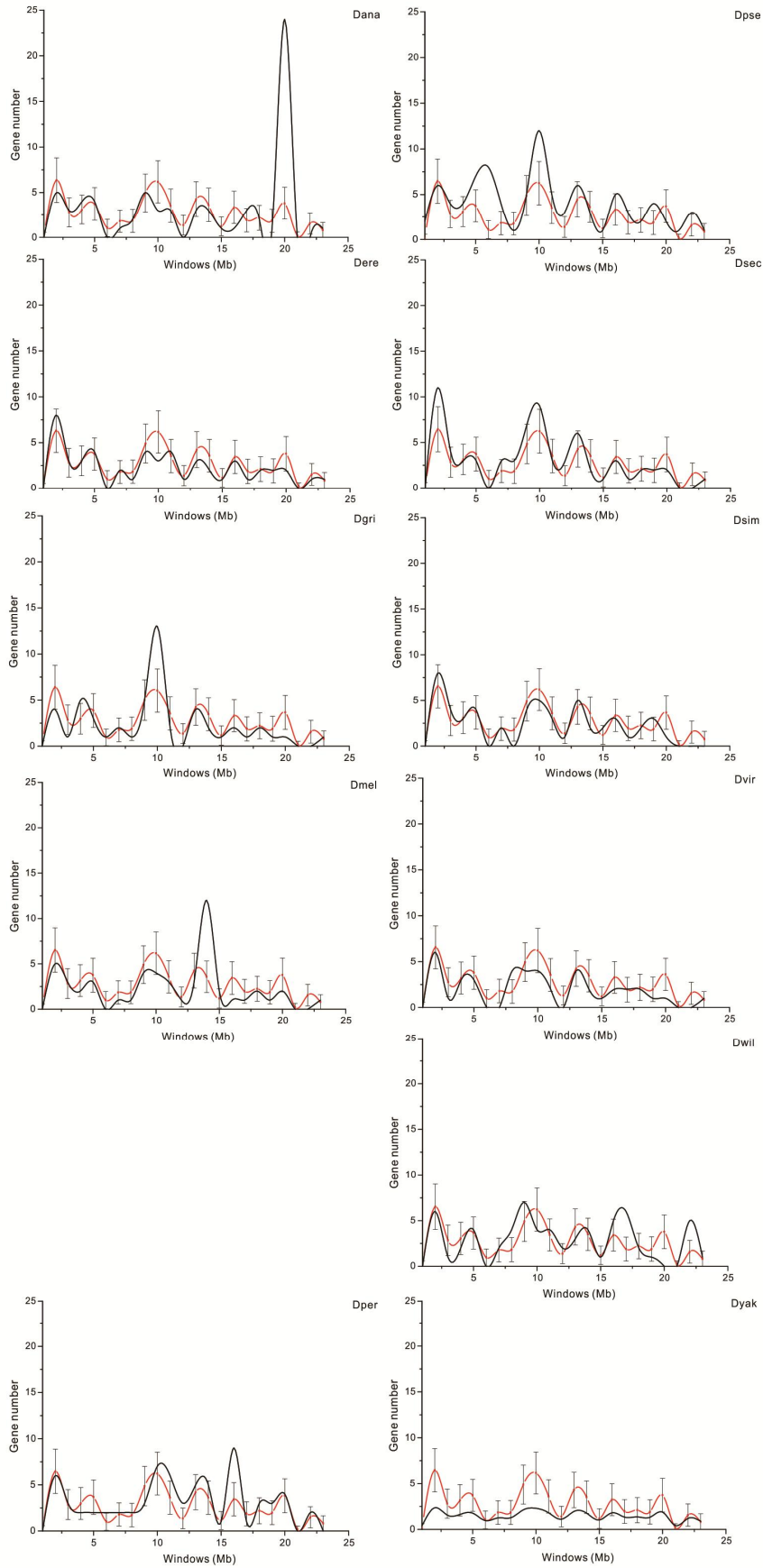

Supplement: Additional file 2: Figure S2 — Distribution of young duplicate genes on chromosomes. (A)-(E) Species-specific duplicates on chromosome 2L, 2R, 3L, 3R and X, respectively. (F) Lineage-specific duplicates of D. pseudoobscura-D. persimilis pair. (G)-(K) Duplicates of complex expansions occurring in 11 species on chromosome 2L, 2R, 3L, 3R and X, respectively. Black lines represent observations, while black bands and red lines (red dots) show confidence intervals and average numbers of genes in corresponding windows. Dmel: D. melanogaster. 2L: chromosome 2L, 2R: chromosome 2R, 3L: chromosome 3L, 3R: chromosome 3R. [file 1471-2164-14-555-S2.pdf]

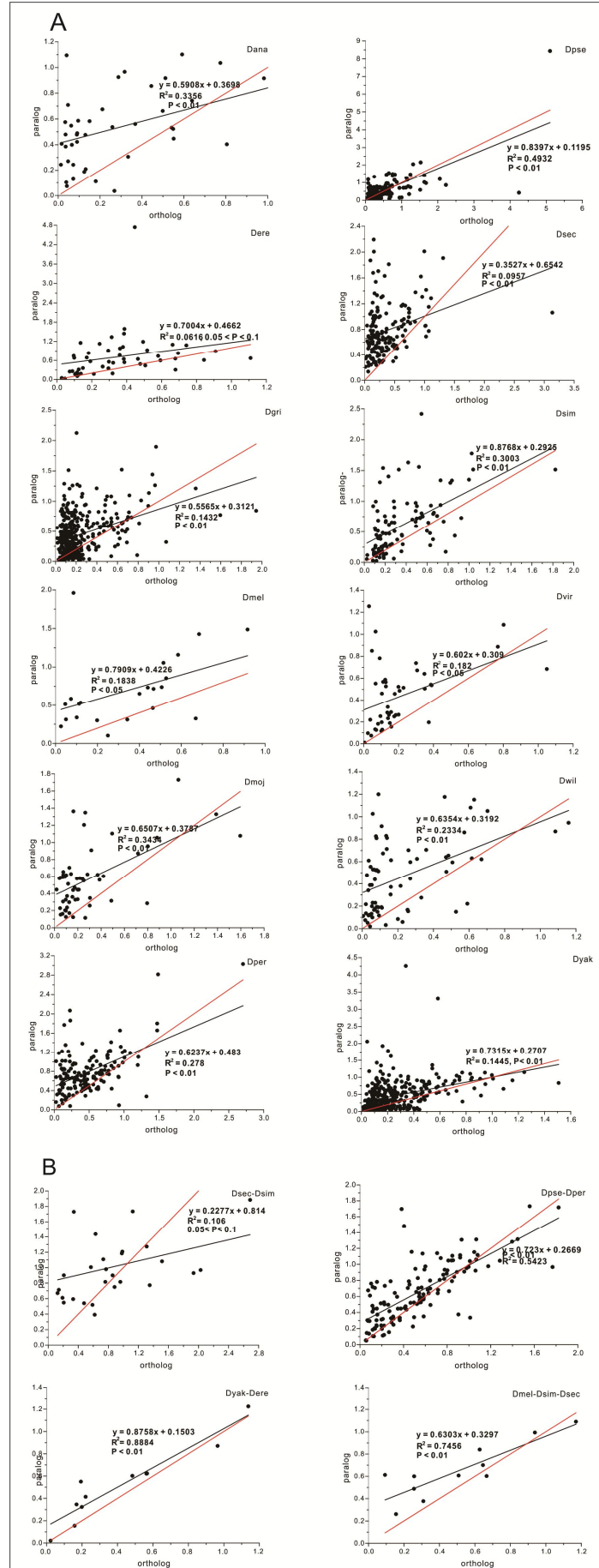

Supplement: Additional file 5: Figure S3 — Mean Ka/Ks ratios of paralog gene pairs vs. ortholog gene pairs. (A) Ka/Ks ratios of species-specific duplicates. (B) Ka/Ks ratios of lineage-specific duplicates. Black lines means the trend line of black dots and red lines represents trend lines with slope = 1. [file 1471-2164-14-555-S5.pdf]
