# Supplementary material for: Functional requirements driving the gene duplication in 12 Drosophila species
Source: BMC Genomics. 2013 Aug 15;14:555. doi: 10.1186/1471-2164-14-555 (PMC3751352; doi:10.1186/1471-2164-14-555)
Supplement: Additional file 4: Table S2 — Structural domains detected in families corresponding to the dots excessively deviating from trend lines. Table S3. Versions of genome sequences and annotations for each species. [file 1471-2164-14-555-S4.doc]

Table S2. Structural domains detected in families corresponding to the dots excessively deviating from trend lines.

| Expansion types | Species | Domains | | P values (T-test) |
| --- | --- | --- | --- | --- |
|  |  | Upper dots | Lower dots |  |
| Species-specific expansions | *D. ananassae* | adh_short | - | P <0.05 * |
|  | *D. erecta* | - | - | P <0.01 ** |
|  | *D. grimshawi* | CBM_14 | - | P <0.01 ** |
|  | *D. melanogaster* | G-alpha | - | P <0.01 ** |
|  | *D. mojavensis* | Kunitz_BPTI | - | P <0.01 ** |
|  | *D. persimilis* | Homeobox, OAR | - | P <0.01 ** |
|  | *D. pseudoobscura* | MAP1_LC3 | - | P =0.535 |
|  | *D. sechellia* | PDZ, MOSC_N, MOSC, REJ | - | P <0.01 ** |
|  | *D. simulans* | Ribosomal_S17 | - | P <0.01 ** |
|  | *D. virilis* | Sec7, PH, Peptidase_M48 | - | P <0.01 ** |
|  | *D. willistoni* | MIP, Fz, Frizzled,CUB, EGF | - | P <0.01 ** |
|  | *D. yakuba* | Coesterase,Turandot | - | P <0.01 ** |
| Lineage-specific expansions | *D. sechellia*-*D. simulans* | - | - | P =0.261 |
|  | *D. yakuba-D. erecta* | - | - | P =0.517 |
|  | *D. pseudoobscura-D. persimilis* | Pam16, BTB, BACK | - | P <0.01 ** |
|  | *D. melanogaster-D. sechellia*-*D. simulans* | - | - | P =0.279 |

Table S3. Versions of genome sequences and annotations for each species.

| Species | Version |
| --- | --- |
| *D.* *ananassae* | r1.3 |
| *D. erecta* | r1.3 |
| *D.* *grimshawi* | r1.3 |
| *D. melanogaster* | r5.30 |
| *D.* *mojavensis* | r1.3 |
| *D. persimilis* | r1.3 |
| *D. pseudoobscura* | r2.13 |
| *D.* *sechellia* | r1.3 |
| *D. simulans* | r1.3 |
| *D.* *virilis* | r1.2 |
| *D.* *willistoni* | r1.3 |
| *D. yakuba* | r1.3 |
